# Identification of miRNAs and their targets in two *Taraxacum* species with contrasting rubber-producing ability

**DOI:** 10.3389/fpls.2023.1287318

**Published:** 2023-11-08

**Authors:** Cuili Liang, Yitong Yan, Yingchao Tan, Xue Yang, Jie Cao, Chaorong Tang, Kaiye Liu

**Affiliations:** ^1^ National Key Laboratory for Biological Breeding of Tropical Crops, Hainan University, Haikou, China; ^2^ College of Tropical Crops, Sanya Nanfan Research Institute of Hainan University, Hainan Yazhou Bay Seed Laboratory, Hainan University, Haikou, China; ^3^ Natural Rubber Cooperative Innovation Center of Hainan Province and Ministry of Education of PR China, Hainan University, Haikou, China; ^4^ Yunnan Institute of Tropical Crops, Xishuangbanna, China

**Keywords:** miRNA, miRNA targets, regulatory modules, natural rubber production, *Taraxacum*

## Abstract

MicroRNAs (miRNAs) are widely involved in various aspects of plant growth and development. However, how miRNAs and their targets regulate natural rubber metabolism remains unclear in the rubber-producing dandelions, which are being developed as alternative commercial sources of natural rubber. Here, we combined small RNA sequencing, degradome sequencing, target gene prediction, and mRNA sequencing to identify miRNAs and their targets in two dandelion species, the high rubber-yielding *Taraxacum kok-saghyz* (Tk) and the low rubber-yielding *T. spadiceum* (Ts). A total of 142 miRNAs, including 108 known and 34 novel ones, were discovered, with 53 identified as differentially expressed (DE) between the latex of Tk and Ts. Degradome sequencing identified 145 targets corresponding to 74 miRNAs. TAPIR and psRNATarget, respectively, predicted 165 and 164 non-redundant targets for the 53 aforementioned DE miRNAs. Gene ontology (GO) enrichment analysis indicated the DE miRNAs and their targets might affect natural rubber production via regulating macromolecular biosynthesis and metabolism in latex. Four critical types of regulatory modules, including miR172-AP2/ERF, miR164-NAC, miR160-ARF, and miRN19-protein kinase, were identified and their interaction networks were constructed, indicating a potential involvement in natural rubber production. The findings and the large miRNA dataset presented here are beneficial to further deciphering the roles of miRNAs in the biosynthesis of natural rubber and medicinal metabolites in dandelion.

## 1 Introduction

Natural rubber (*cis*-1,4-polyisoprene, NR) is a strategically important raw material used in the manufacturing of medical, agricultural and industrial products (Cornish, 2001). It is a high molecular polymer with collective elite physical and mechanical properties, such as elasticity, abrasion and impact resistance, heat dispersion and malleability at cold temperature, making it irreplaceable by synthetic alternatives in most applications (Puskas et al., 2014). According to International Rubber Study Group’s (IRSG) report, the global NR demand and production surged by 9.4% and 5.7% in 2021, respectively. Total NR production is forecasted to further grow by 3.7%, reaching 14.80 million tons in 2023. Presently, NR is commercially obtained exclusively from a single tropical tree species: the Para rubber tree (*Hevea brasiliensis*). However, NR supplies from *Hevea* tree are not sustainable due to its restriction to specific tropical regions, susceptibility to fungal infections (especially the South American Leaf Blight, SALB), and laborious and skilled harvest work (Mooibroek and Cornish, 2000; van Beilen and Poirier, 2007a; Ahrends et al., 2015; Men et al., 2019). Therefore, it is urgent to develop alternative rubber crops. So far, more than 1800 plant species have been identified as containing rubber in their latex (Metcalfe, 1967). Nevertheless, only a few of these species, including *Parthenium argentatum* Gray, two dandelion species of *Taraxacum kok-saghyz* (Tk) and *T. brevicorniculatum* (Tb), *Lactuca sativa*, and *Ficus bengalensis*, produce NR with an average molecular weight over 1000 kg/mol, an essential determinant of high rubber quality (Rousset et al., 2021; Salehi et al., 2021).

The genus *Taraxacum*, commonly known as the dandelion plants, is a member of the family Asteraceae, subfamily Cichorioideae, and is widely distributed in the temperate zones (Schütz et al., 2006). *Taraxacum* has long been used as medicinal herbs in traditional Chinese medicine to treat hepatitis and the immune response to upper respiratory infections, bronchitis, and pneumonia (Sweeney et al., 2005; Qadir et al., 2022). In Germany, the application of *Taraxacum* is recorded in medicating gout, diarrhea, blister, spleen, and liver complaints (Faber, 1958). In North America, the infusions and decoctions of the *Taraxacum* are applied to treat kidney disease and heartburn (Sweeney et al., 2005). In Mexico and Turkey, the dandelion is used as a laxative and potent anti-diabetic medicine (Schütz et al., 2006). The therapeutic actions of *Taraxacum* species have been partially ascribed to some sesquiterpenes in their roots and leaves. Recently, the extracts of potential pharmaceutical importance, including a number of sesquiterpenes, triterpenes, phytosterols, and phenolic compounds from dandelion roots, were identified in dandelion plants (Leu et al., 2003; Kisiel and Michalska, 2006). About seventy *Taraxacum* microspecies are identified in China, of which Tk and Tb are known to produce high-quality NR in their latex, the milky cytoplasm of specialized cells known as laticifers (Post et al., 2012). Tk, also called Russian dandelion or Rubber dandelion, was exploited as the rubber-producing crop in the USSR due to it significant accumulation of high-quality rubber in the root (5% to 24% on a dry weight basis) (van Beilen and Poirier, 2007b). In its roots, the laticifer cells, appearing as long tubular vessels produce and store latex, similar to the laticifers in the rubber tree’s bark (Ramirez-Cadavid et al., 2017). Meanwhile, its known pathway of rubber biosynthesis also corresponds roughly with that of the rubber tree (Tang et al., 2016; Lin et al., 2017). Proteins identified as important for rubber biosynthesis in the rubber tree, including CPT (cis-prenyltransferase), CPTL (cis-prenyltransferase-like), and REF/SRPP (rubber elongation factor/small rubber particle protein), are also recognized as crucial components of the rubber-producing machinery in dandelion species (Salehi et al., 2021). The advantages of Tk as perennial herb, including a relatively simple genome, wide planting area, small plant architecture, ease to transform and harvest, and a relatively short life cycle, make it an ideal model plant for studying natural rubber biosynthesis and production (Lin et al., 2017; Lin et al., 2021). Hitherto, we know little about the regulatory mechanisms of the biosynthesis of secondary metabolites including the high molecular rubber and the component of therapeutic agents in *Taraxacum*.

miRNAs are endogenous noncoding RNAs with 20-24 nucleotides (nt) that play important roles in regulating plant growth and development as well as biotic and abiotic stress (Song et al., 2019). They are produced through a multistep process including transcription of miRNA genes, precursor processing, and assembly of miRNA-induced silencing complex (miRISC). The mechanisms of miRNA in regulating target gene expression are conserved across different plant species, including cleavage of target genes by base pairing, translational repression, and miRNA-dependent DNA methylation (Jones-Rhoades et al., 2006). The crucial roles of miRNAs have been reported in the regulation of plant growth and development (Chen, 2009). For instance, in *Arabidopsis*, miR156, miR172, and miR159 are involved in the regulation of vegetative phase change, floral transition, and flowering time (Aukerman and Sakai, 2003; Millar and Gubler, 2005; Schwab et al., 2005; Wu and Poethig, 2006). Additionally, miR160 and miR164 play a role in regulating root development and the emergence of lateral roots (Guo et al., 2005; Mallory et al., 2005). miRNAs are also involved in the responses of plants to environmental stimulation as well as biotic and abiotic stresses (Song et al., 2019). For example, miR396 inhibits leaf growth by targeting *GRFs* (Growth Regulating Factor) under UV-B radiation damage in *Arabidopsis* (Casadevall et al., 2013). miR393 targets the auxin receptor genes *HvTIR1* and *HvAFB*, contributing to the inhibition of root elongation under aluminum stress in barley (Bai et al., 2017). Overexpression of miR319 enhances cold tolerance of rice and sugarcane by down-regulating the *TCP* transcription factors (Thiebaut et al., 2012; Yang et al., 2013). Down-regulation of miR165/166 confers enhanced drought resistance via the elevation of ABA levels in *Arabidopsis* (Yan et al., 2016). Moreover, miRNAs act as a key regulator in the production of secondary metabolites, which are predicted to protect plants from a series of environmental conditions (Zhang et al., 2022). miR5021 and miR414 regulate the biosynthesis of terpenoid backbone, sesquiterpenoid, and triterpenoid in *Xanthium strumarium* (Fan et al., 2015). miR2161 regulates the biosynthesis of benzylisoquinoline alkaloids in opium poppy plants by targeting mRNA encoding enzymes such as *S-adenosyl-L-methionine*, *30-hydroxy-N-methylcoclaurine*, and *40-O-methyltransferase* (Gupta et al., 2017). Recently, a total of 574 and 396 miRNAs were predicted, respectively, in Tk and *T. officinale* by homologous retrieval using the publicly available RNA-seq and EST data of these two *Taraxacum* species (Karimi et al., 2022). Of these miRNAs, miR5021, miR838 and miR1533 were speculated by the KEGG analysis to participate in the terpene biosynthesis (Karimi et al., 2022). So far, the miRNAs and their targets have never been investigated by targeted sequencing analysis in *Taraxacum* and the roles of miRNA-target modules in the regulation of rubber biosynthesis are not known.

Here, to identify the key miRNA-target modules in the natural rubber biosynthesis pathway of *Taraxacum* plants, we performed small RNA sequencing, degradome sequencing, target gene prediction, and mRNA sequencing in a pair of *Taraxacum* species with contrasting ability of rubber production, the elite Tk and the inferior Ts (*T. spadiceum*). A vast number of miRNAs were identified in the two dandelions species, and most importantly 53 ones revealed to be differentially expressed between the latex of the two dandelion species. The targets of these differentially expressed miRNAs were significantly enriched in the processes of macromolecule biosynthesis and metabolism in latex. The miRNA-target regulatory modules that implicate in the biosynthesis of natural rubber were further investigated. Together, the results we obtained provide the first-hand information on the role of miRNAs in natural rubber biosynthesis and a valuable basis for exploring the detailed functions of miRNAs in rubber-producing dandelions.

## 2 Materials and methods

### 2.1 Plant material and growth conditions

Tk and Ts germplasm populations were collected in Xinjiang, China, and the accessions of Ts-01 and Tk-20 from the two microspecies were selected for the study. Tk and Ts plants were propagated by tissue culture following the procedures: the leaves were cut into small pieces of 0.5 cm^2^ in liquid Murashige & Skoog (MS) medium (4.4 g/L MS, 20 g/L sucrose, pH 5.8) and put on the propagation medium (4.4 g/L MS, 20 g/L sucrose, 0.7 mg/L kinetin, 0.2 mg/L IAA, 9 g/L Agar, pH 5.8) for 3 weeks; the regenerated buds were transferred to the rooting medium (2.2 g/L MS, 30 g/L sucrose, 9 g/L Agar, pH 5.8) for 4 weeks. The resulting plantlets were transplanted to soil and grown in a growth chamber at 16 h light/8 h dark, 24°C, and 60% humidity for 8 weeks. Afterwards, these plants were transferred to a 6°C growth chamber for 4 weeks of vernalization, and then cultivated in the growth chamber at 16 h light/8 h dark, 24°C, and 60% humidity until flowering.

### 2.2 Histochemical staining and rubber content determination

The roots from five-month-old Tk and Ts plants were fixed in FAA solution for 24 hours, and then sectioned into 6 μm slices. The slices were stained with saturated oil red O solution for 10 minutes, differentiated in 60% isopropanol for 10 seconds, washed with double distilled water and then sealed with glycerin gelatin. The bright field observations were performed using a microscope (SZX16, Olympus, Japan). One gram of dried roots from the Tk and Ts plants was ground into a fine powder and boiled in 20 ml of double-distilled water for an hour. The supernatant was discarded, and 20 ml of 3% KOH was added and boiled for another 2 hours. The coagulated rubber was taken out and dried to a constant weight in a hot air oven at 60°C, and then weighted.

### 2.3 RNA extraction, library construction and sequencing

During the full flowering stage, total RNA was extracted from the flower, leaf, root and latex of Tk and Ts using the TRNzol Universal Reagent (Catalog Number 4992730) from TIANGEN Biotech Co., Ltd. (Beijing, China) according to the manufacturer’s instructions. The quality and integrity of RNA were evaluated using the INFINTE 200 PRO (TECAN, Switzerland) and Agilent 2100 Bioanalyzer (Agilent Technologies, CA, USA). In total, twelve small RNA libraries were constructed for the both *Taraxacum* species, including one for each of the tissues of flower, leaf and root, and three for the tissue of latex, and subjected to small-RNA sequencing. Briefly, the libraries were constructed with approximately 2.5 µg total RNA per sample by using the NEBNext^®^ Multiplex Small RNA Library Prep Kit for Illumina (NEB, MA, USA). The small RNA was ligated with 3′ and 5′ end adapters, respectively, followed by reverse transcription to synthesize cDNA and sequenced on the Illumina HiSeq 2500 platform. Six mRNA libraries from latex were constructed by using the NEBNext Ultra RNA Library Prep Kit following the manufacturer’s instructions. mRNA libraries were sequenced by the Illumina NovaSeq™ 6000 platform to generate 150-bp paired-end reads. About 100 µg total RNA from the tissues of flower, leaf, root and latex of each dandelion species were equally pooled for degradome library construction. Degradome cDNA library was also processed on the Illumina HiSeq 2500 platform.

### 2.4 Identification of miRNA and differentially expressed miRNA

We processed the raw reads of miRNA sequencing using the following steps: the data quality was evaluated by fastp v0.210 (Chen et al., 2018); the adapter sequences, low-quality reads, and inserted reads of less than 18 nt were removed by Cutadapt v2.10 (Martin, 2011); the structural and organelle RNAs, including tRNA, rRNA, snoRNA, and snRNA, were removed by aligning the reads to the Rfam database (https://rfam.org/) and organelle RNA database. To identify known and novel miRNAs, the retained reads were aligned to the miRBase V22 (https://www.mirbase.org) and the reference genome (Unpublished data), respectively. The reads were aligned to the reference genome by using bowtie v1.3.1 with the following parameters: –all -m 20 –best –strata -v 1 (Langmead et al., 2009). The novel miRNAs were further identified by sRNAminer v1.1.1 (https://github.com/kli28/sRNAminer) using the merged data of all the small RNA sequencing libraries (Axtell and Meyers, 2018; Feng et al., 2019). The miRNA expression level was calculated and normalized to transcripts per 10 million (TP10M), and the differentially expressed miRNAs were identified and screened by DESeq2 with log_2_|FC| > 1, padj < 0.01 and total read counts >100 of six latex libraries (Love et al., 2014).

### 2.5 Target prediction, degradome, and mRNA sequencing analysis

The targets of differentially expressed miRNA were predicted by TAPIR (score <= 4; free energy ratio >= 0.7) and psRNATarget (expectation <= 2; other parameters were set to default), and only the target genes that were jointly identified by two software programs were used for further analysis (Bonnet et al., 2010; Dai et al., 2018). Degradome sequencing reads were analyzed by the CleaveLand v4.5 pipeline using the parameters: mode 1 and a p-value of less than 0.05 (Addo-Quaye et al., 2009). miRNA targets of different categories were represented in target-plots (T-plots). The mRNA sequencing data were analyzed following the HISAT2 v2.2.1/StringTie v2.1.5 pipeline (Pertea et al., 2016). The gene expression level was normalized by Fragments Per Kilobase of exon model per Million mapped fragments (FPKM) and the differentially expressed genes were determined by DESeq2, with the padj < 0.01.

### 2.6 GO enrichment analysis

The GO analysis of miRNA targets was performed using the OmicShare tools, a free online platform for data analysis (https://www.omicshare.com/tools).

### 2.7 miRNA-mRNA regulatory networks construction

The regulatory networks of miRN19, miR160a, miR164a and miR172a, and their targets were constructed and visualized by Cytoscape V3.9.1 (Shannon et al., 2003).

## 3 Results

### 3.1 Comparison of rubber production between Tk and Ts

**Figure 1:**
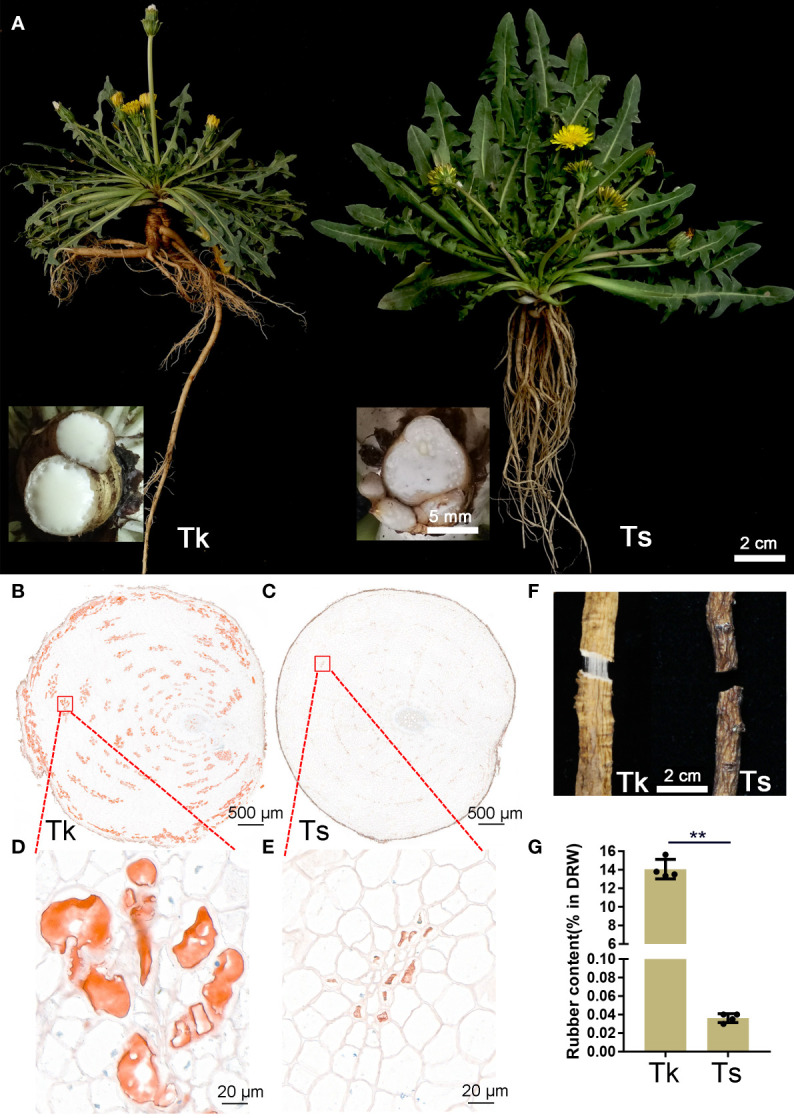
Phenotypic comparison between Tk and Ts in the flowering period. The representative plants of Tk-20 and Ts-01 in the flowering period **(A)**. In the insets, the outflow of latex in the Tk and Ts was shown after cutting the roots. Oil red O staining was conducted on root cross section of Tk **(B, D)** and Ts **(C, E)**. Rubber filaments were compared between the dried roots of Tk and Ts **(F)**. Natural rubber contents were measured between Tk and Ts **(G)**. ** indicates *p* < 0.01.

Tk and Ts plants were propagated from leaves by tissue culture, vernalized and grown in pots in the growth chamber. The rubber production phenotypes were measured at the peak flowering time, which was about two months after vernalization (**Figure 1A**). Compared to Ts, much more latex outflowed in Tk after cutting the main root. Oil-red O staining of transverse root sections showed both Tk and Ts contain ring-shaped and well-dispersed young and mature laticifer cells (**Figures 1B, C**). Nevertheless, the intensity of oil-red staining revealed that the number of rubber particles in the laticifer cells of Tk was significantly higher than that of Ts (**Figures 1D, E**). When we broke and pulled apart the dried roots, apparent rubber filaments were observed in Tk but not in Ts, indicating a much higher rubber content of Tk than that of Ts (**Figure 1F**). Moreover, the rubber content of Tk was quantitatively determined by dry root weight to be 14.07%, whereas that of Ts was barely 0.03%, confirming a striking difference in rubber content between Tk and Ts (**Figure 1G**).

### 3.2 Small RNA sequencing in Tk and Ts

**Figure 2:**
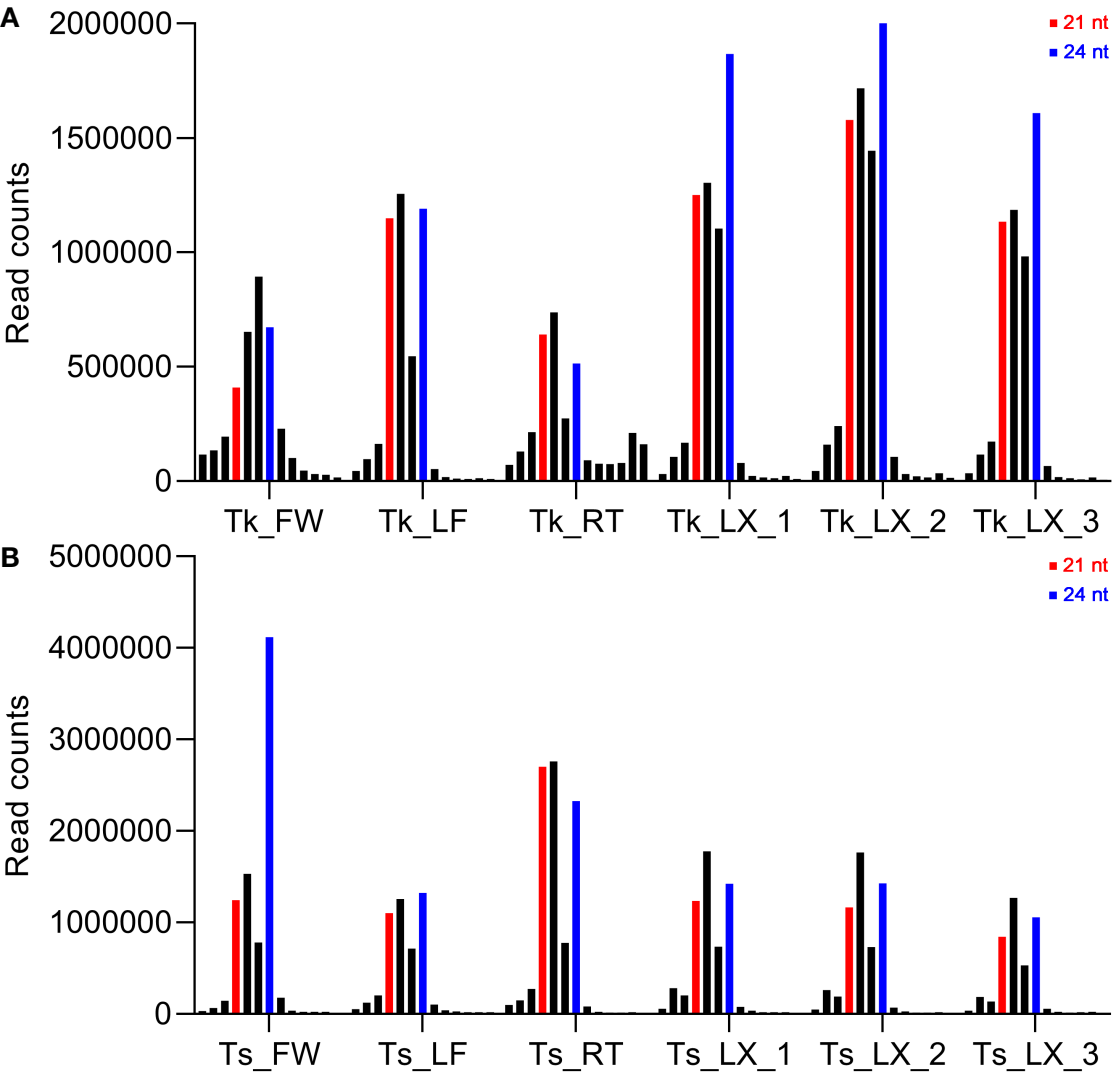
Length distribution and abundance of small RNAs in flower (FW), leaf (LF), root (RT) and latex (LX) of Tk and Ts. The sequential length distributions of clean reads ranging from 18 to 30 nt of Tk **(A)** and Ts **(B)** are shown. The red and blue bars indicate the lengths of 21 and 24 nt, respectively.

In order to achieve more integrated miRNA information of the genus *Taraxacum*, a total of 12 small RNA (sRNA) libraries were constructed and sequenced from the tissues of flower (FW), leaf (LF), root (RT) and latex (LX) in Tk and Ts. Small RNA-Seq generated raw reads ranging from 9,907,779 to 18,806,129 per library. The raw sequences were computationally analyzed to remove low quality sequences, adaptors, and reads shorter than 18 nt. After filtering, 3,520,292 to 8,209,888 clean reads per library, representing 28%-77% of the initial raw reads, were retained. The sRNA lengths varied extensively from 18 to 30 nt, with the majority ranging from 21 to 24 nt (**Figure 2**).

### 3.3 Identification of miRNAs and differentially expressed miRNAs

**Figure 3:**
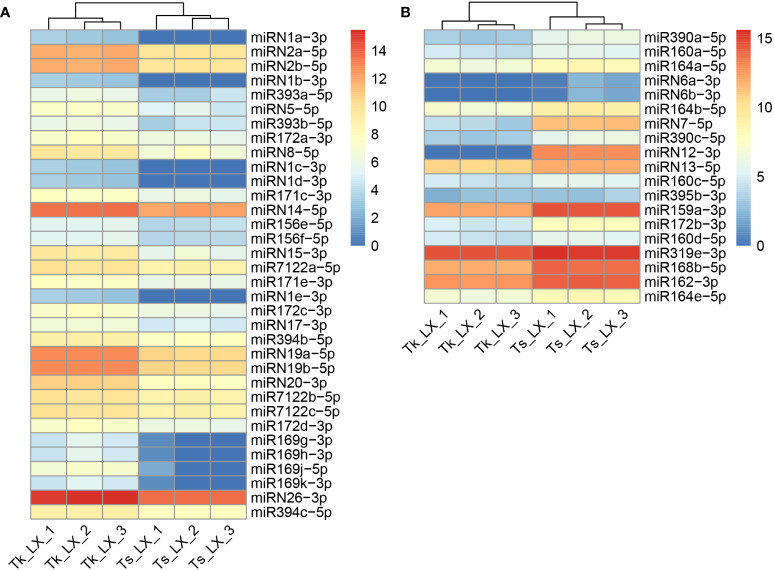
Heatmap of differentially expressed miRNAs. The up-regulated **(A)** and down-regulated **(B)** miRNAs are identified in the latex of Tk vs. Ts. The color is based on the log2(TP10M) value.

The clean reads of these miRNA libraries were subjected to mapping against the miRNABase V22. Furthermore, sRNAmer was used to identify new miRNA by merging all the miRNA sequencing libraries. Totally, we detected 108 known and 34 novel miRNAs in these two dandelion species, with the sequences and genomic locations of miRNA, miRNA star, and miRNA precursor summarized in **Supplementary Table 1**. To identify the differentially expressed miRNA in the latex between Tk and Ts, we quantified the expression levels of the 142 identified miRNAs (**Supplementary Table 2**). A total of 53 miRNAs, including 32 known and 21 newly identified ones, were differentially expressed (DE) between the two dandelion accessions (**Supplementary Table 2**). Among these DE miRNAs, 34 were up-regulated and 19 were down-regulated in the latex of Tk when compared to that of Ts (**Figure 3**; **Supplementary Table 2**).

### 3.4 miRNA targets analysis

**Figure 4:**
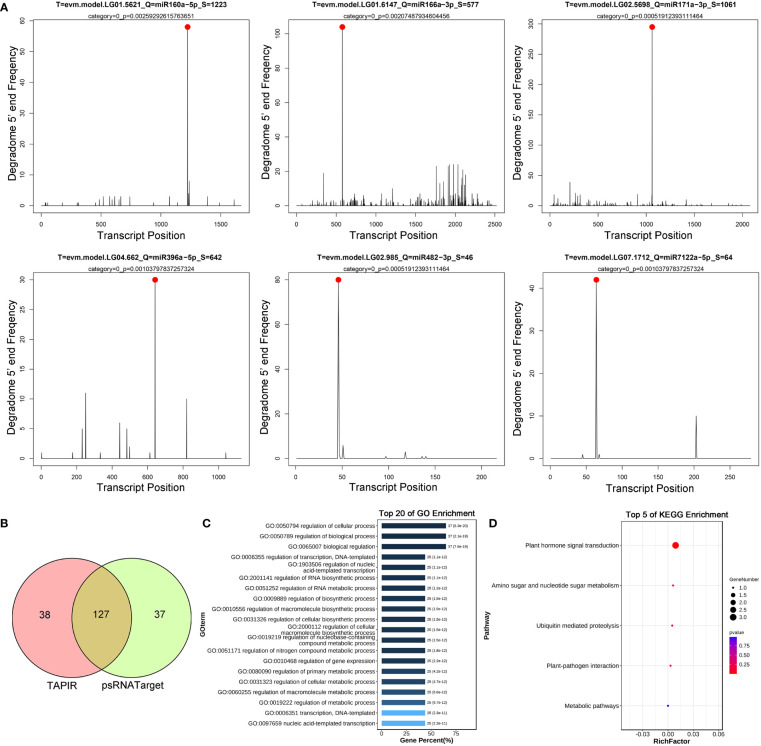
miRNA target identification and functional enrichment analysis. The six representative miRNA targets are identified by degradome sequencing **(A)**. Venn diagram for targets of differentially expressed miRNAs are predicted by TAPIR and psRNATarget **(B)**. The top 20 GO enrichments are shown for the targets of differentially expressed miRNA **(C)**. The top 5 KEGG enrichments are displayed for the targets of differentially expressed miRNA **(D)**.

We combined the degradome sequencing and target prediction to identify the miRNA targets. Using the degradome sequencing data, 145 targets corresponding to 74 miRNAs were identified by the CleaveLand4 pipeline. The target plots (T-plots) revealed the comprehensive categories and cleavage information of all these miRNAs and their targets (**Supplementary Data 1**). The T-plots for six miRNAs and their representative targets are shown in **Figure 4A**. In order to obtain more complete miRNA-target information, 53 DE miRNAs were also used to predict targets by the software programs TAPIR and psRNATarget. A total of 298 and 306 targets, corresponding to 165 and 164 non-redundant genes, were identified by TAPIR and psRNATarget, respectively (**Supplementary Table 3**). Among these non-redundant target genes, 127 ones were identified by both types of software (**Figure 4B**), and subjected further gene ontology (GO) functional annotation. The GO results revealed six statistically significant enrichment for the macromolecule-related pathways, i.e. “regulation of macromolecule biosynthetic process, GO:0010556”, “regulation of macromolecule metabolic process, GO:0060255”, “regulation of cellular macromolecule biosynthetic process, GO:2000112”, “cellular macromolecule metabolic process, GO:0044260”, “macromolecule biosynthetic process, GO:0009059”, and “macromolecule metabolic process, GO:0043170” (**Figure 4C**; **Supplementary Table 4**). The root ontology analysis of the enriched GO terms revealed that these pathways related to macromolecules are interconnected and fall within the subcategory of GO:0043170 (**Supplementary Figure 1**). KEGG enrichment analysis identified the involvement of these target genes in five pathways, particularly the significant enrichment in plant hormone signal transduction (**Figure 4D**). These observations suggest the DE miRNAs and their targets might participate in the biosynthesis and metabolism of natural rubber, a type of macromolecule.

### 3.5 miRNA-target modules implicated in natural rubber metabolism

**Figure 5:**
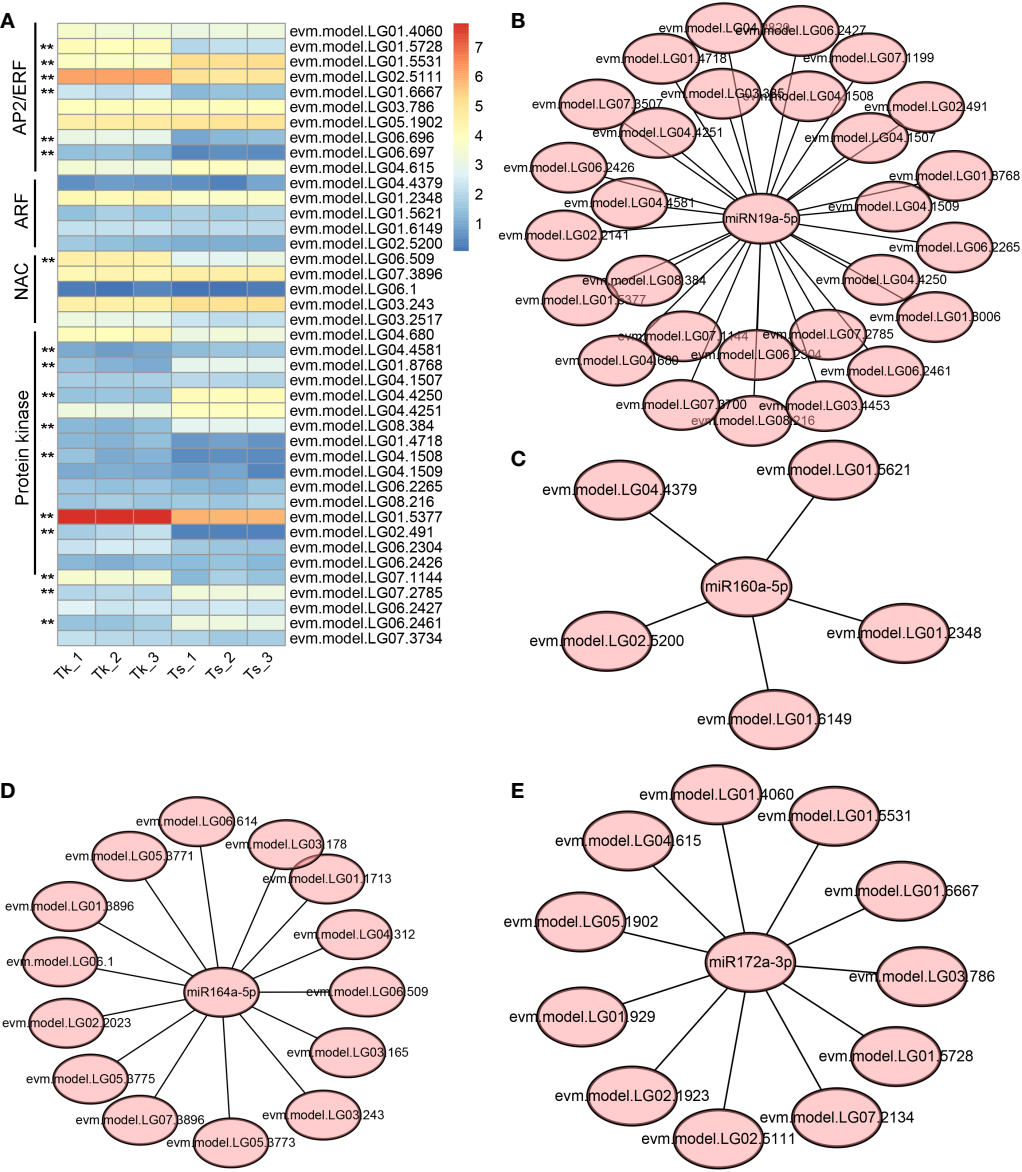
Regulatory networks of miRNA-target involved in the biosynthesis and metabolism of natural rubber macromolecules. Gene families involved in macromolecule biosynthesis and metabolism and their expression levels in Tk and Ts latex **(A)**, the color is based on the log2(FPKM+1) value. ** indicates a significant difference in gene expression between Tk and Ts at the padj-value < 0.01 level. The regulatory networks are shown between miRN19a **(B)**, miR160a **(C)**, miR164a **(D)** and miR172a **(E)**, and their target genes.

We compiled the list of miRNA targets involved in macromolecule-related metabolism based on the GO categories and compared their expression levels in the latex of Tk and Ts. Four kinds of target gene families stood out, including *AP2/ERF* (APETALA2/Ethylene Responsive element binding Factor), *ARF* (Auxin Response Factor), *NAC* (NAM, ATAF1/2 and CUC2 domain containing protein), and *protein kinase*. Of them, six *AP2/ERF* genes, one *NAC* gene, and eight *protein kinase* genes were expressed significantly different between the latex of Tk and Ts (**Figure 5A**). The DE target genes of *AP2/ERF*, *NAC*, *ARF*, and *protein kinase* were mainly targeted by miR172, miR164, miR160, and miRN19 families, respectively. To further understand the regulatory networks between these miRNAs and their targets, the miRNA-mRNA interaction network maps were constructed, including the association of miR164 with 15 targets, miRN19a with 28 targets, miR160a with 5 targets, and miR172a with 11 targets. (**Figures 5B–E**). These DE miRNAs and their target genes might play an important role in the metabolism of natural rubber.

## 4 Discussion

Plants of the genus *Taraxacum*, are widespread throughout the world and about 2800 species within 60 sections have been identified (Kirschner et al., 2013). In addition to its long history of use in traditional medicine, the development of rubber-producing dandelions, especially the Russian dandelion *T. kok-saghyz* (Tk), into alternative rubber crops has regained much attention since the early 2000s (Martinez et al., 2015; Salehi et al., 2021). However, the disadvantages of Tk, such as poor growth vigor and self-incompatibility, make essential the incorporation of other *Taraxacum* species in Tk breeding and scientific projects (Zeisek et al., 2019; Salehi et al., 2021). In this study, *T. spadiceum* (Ts), an apomictic dandelion species with strong growth vigor and low root rubber-content, was investigated together with Tk (**Figure 1**). The findings of miRNA-targets and the vast amount of small RNA datasets obtained here from the two dandelion species will benefit not only the research in rubber production but that in the other important agronomic traits for Tk domestication.

The read distribution of the sRNA sequencing data generally showed that the abundances of 21 and 24 nt sRNAs are the highest in different tissues (Chen, 2009). However, sRNAs of 22 and 23 nt revealed to be highly expressed in our sequencing data (**Figure 2**). Notably, the abundances of the 22-nt sRNA were the highest in all the three latex samples of Ts when compared to the other length-types of sRNAs (**Figure 2B**). In *Arabidopsis*, DCL2 is thought to regulate the biogenesis of viral 22 nt siRNAs, which repress the translation of their cognate mRNAs as well as global translation (Deleris et al., 2006). In addition, the 22 nt sRNA have also been shown play an important role in fine-tuning plant growth and stress responses (Wu et al., 2020). Latex produced by rubber-producing dandelions consists of multiple secondary metabolites and is thought to defend against biotic and abiotic stresses (Ramos et al., 2019). Therefore, the highly expressed 22 nt sRNA in latex might participate in both latex metabolism and plant defense. The mechanisms of generation and physiological functions have never been reported for the type of 23 nt sRNAs in plants. Hence, the exact roles of 22 and 23 nt sRNAs in latex needs to be further investigated, especially their possible involvement in the latex metabolism of the rubber-producing dandelion species. miRNAs and their targets are highly conserved in plants. In this study, 108 conserved miRNAs and 34 dandelion specific miRNAs were identified. Of which, a total of 53 were differentially expressed (DE) between the latex of Tk and Ts, including 32 conserved and 21 newly identified miRNAs (**Supplementary Table 2**). Among the DE miRNAs, miRN26 and miR319e were, respectively, the most abundant newly identified and conserved miRNAs (**Figure 3**). These results imply that species-specific miRNAs may play an important role in latex formation and rubber production. We used degradome sequencing to identify the miRNA target genes, and only 145 targets for 74 miRNAs were obtained (**Supplementary Data 1**). Target genes could be detected for some of the conserved miRNAs, including miR156, miR160, miR166, miR167, miR171, miR319, miR393, and miR396, but not for nearly half of miRNAs identified by degradome sequencing. Four possible reasons might account for this result: the expression abundance of some miRNA targets is too low to be detected; the expression of some target genes exhibits strict spatio-temporal characteristics; some novel and specific miRNAs could have arisen recently and have not necessarily acquired a target; and the sequencing depth of the degradome library needs to be increased. To obtain comprehensive miRNA targets implicated in rubber production, we further used target prediction to identify miRNA target genes for the DE miRNAs between the latex of Tk and Ts. TAPIR and psRNATarget predicted 298 and 307 miRNA targets, respectively, including 165 and 164 non-redundant genes (**Supplementary Table 3**). Notably, a total of 127 non-redundant targets were detected by both programs. Together, we obtained comprehensive and credible miRNA target information of these DE miRNAs.Terpenoids, including monoterpenes, sesquiterpenes, diterpenes, triterpenes, tetraterpenes and polyterpenes, are types of natural hydrocarbons that are widely distributed in plants. The C5 phosphates, isopentenyl diphosphate (IPP) and dimethylallyl diphosphate (DMAPP), are the common initial molecules for synthesizing of both terpenoid and natural rubber in plants (Hossain et al., 2022). Currently, miRNAs have been widely reported in the regulation of terpenoids biosynthesis. For instance, miR4995 plays a promoter function in the biosynthesis of terpenoids that ultimately affects the production of picroside-I in *Picrorhiza kurroa Royle* (Vashisht et al., 2015). The miR5021 was predicted to target the enzymes that affect the synthesis of terpenoid indole alkaloids in *Catharanthus roseus* (L.) G. Don (Pani and Mahapatra, 2013). Overexpression of miRStv_11 and anti-miR319 enhances the biosynthesis of steviol glycosides by suppressing the expression levels of *KO*, *KS*, *UGT86C2* and *KAH* in *Stevia rebaudiana* (Saifi et al., 2019). In this work, GO analysis on the targets of the differentially expressed miRNAs between the latex of Tk and Ts revealed significant enrichment for six pathways involved in the regulation of macromolecule biosynthesis and metabolism (**Figure 4**; **Supplementary Table 4**). These target genes might play an important role in the regulation of natural rubber biosynthesis in these two dandelion species with distinct rubber content in their roots (**Figure 1**). KEGG analysis revealed that the target genes were enriched in five pathways, with plant hormone signal transduction being the most significant pathway. Production practices have demonstrated that extrinsically applying ethylene on rubber trees can enhance latex metabolism and rubber flow time, ultimately leading to a significant increase in rubber yield (Domiciano et al., 2018). JA (jasmonic acid) signaling involves the nature rubber production by regulating the laticifer differentiation in rubber tree (Chao et al., 2023). These results indicate that plant hormones play an important role in the biosynthesis of natural rubber. Four types of miRNA-target modules are predicted to be widely involved in macromolecule-related processes (**Figure 5**). Notably, the miRN19-protein kinase module is a module specific in the rubber-producing dandelions (**Supplementary Table 3**). The expression levels of many protein kinase genes in latex were significantly different between Tk and Ts, reinforcing the regulatory roles of the DE miRNAs identified to target protein kinases. Whether the species-specific miRNA-target regulatory modules in *Taraxacum* participate directly or indirectly in natural rubber production warrants further investigation.

## 5 Conclusion

In summary, 142 miRNAs were discovered by miRNA sequencing in the two dandelion species, Tk and Ts, with striking differences in rubber production, and 53 are differentially expressed in their latex. The targets of these differentially expressed miRNAs were significantly enriched in the processes of macromolecular biosynthesis and metabolism. Four types of miRNA-target modules, including miR172-AP2/ERF, miR164-ARF, miR160-NAC and miRN19-protein kinase, might play an important role in rubber production for their differential expressions in the latex of the two dandelions. Taken together, the findings and extensive datasets presented here provide a basis for the deeper understanding of the regulatory roles of miRNA in dandelion rubber production.

## Data availability statement

The datasets presented in this study can be found in online repositories. The names of the repository/repositories and accession number(s) can be found in the article/**Supplementary Material**.

## Author contributions

CL: Investigation, Resources, Software, Validation, Writing – original draft. YY: Data curation, Formal Analysis, Investigation, Methodology, Writing – original draft. YT: Data curation, Formal Analysis, Methodology, Writing – original draft. XY: Resources, Visualization, Writing – original draft. JC: Methodology, Visualization, Writing – original draft. CT: Funding acquisition, Supervision, Writing – review & editing. KL: Conceptualization, Funding acquisition, Supervision, Writing – original draft.

## Supplementary material

Supplementary Figure 1 Root ontology analysis of the enriched GO terms. The GO terms involving macromolecule-related pathways are labeled in red and displayed in an enlarged font.
