# Supplementary figures and images for: Identification of miRNAs and their targets in two Taraxacum species with contrasting rubber-producing ability (part 2 of 2)
Source: Front Plant Sci. 2023 Nov 8;14:1287318. doi: 10.3389/fpls.2023.1287318 (PMC10663287; doi:10.3389/fpls.2023.1287318)

**T=evm.model.LG01.5436\_Q=miR6275-3p\_S=116**

category=0\_p=0.00569556437049679

Degradsome 5' end Frequency

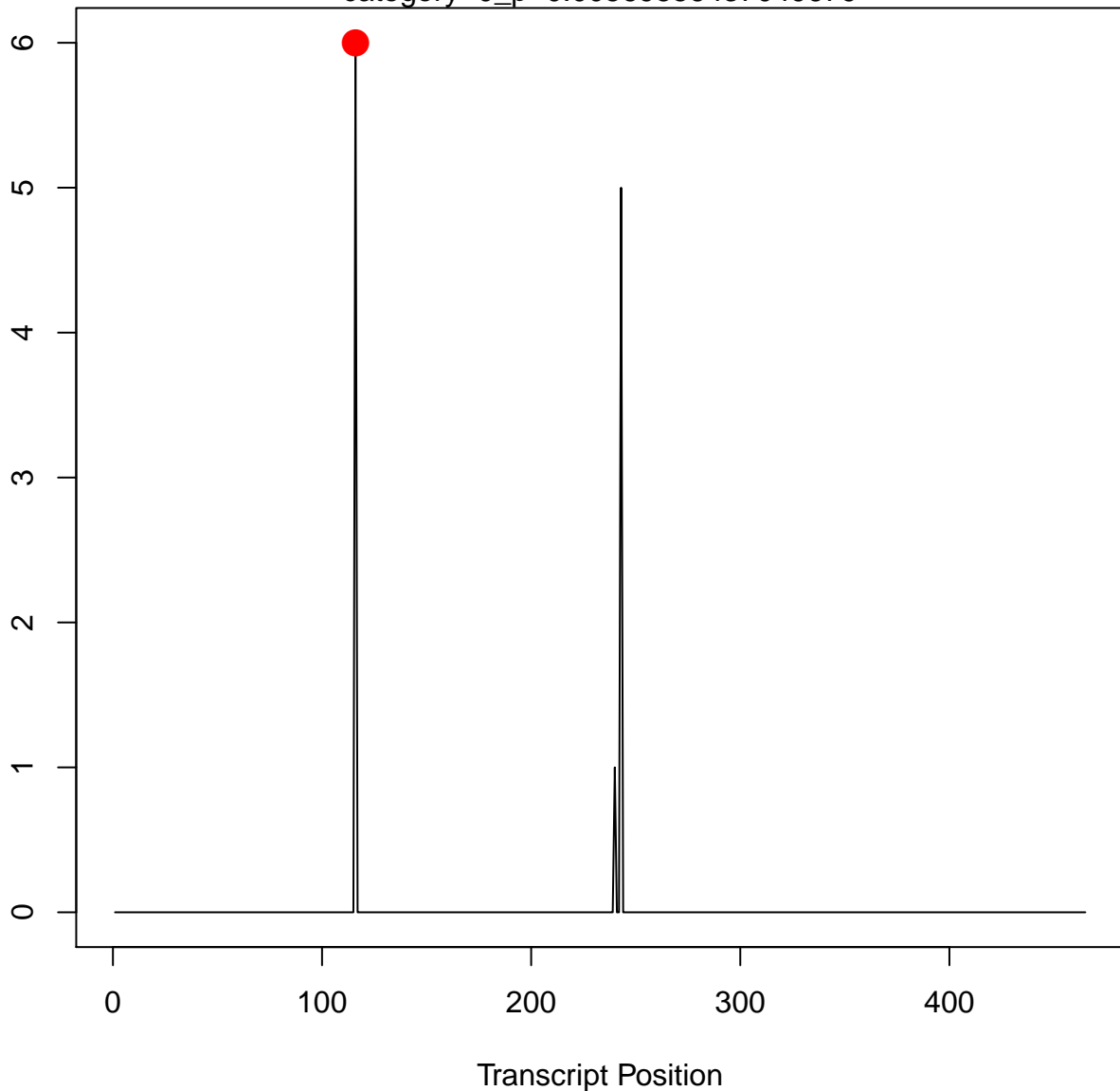

Supplement: Supplementary file 1 [file DataSheet_1.zip › The miRNA-target modules identified by the CleaveLand4/miR6275-3p_evm.model.LG01.5436_116_TPlot.pdf]

**T=evm.model.LG05.3668\_Q=miR6275-5p\_S=209**

category=1\_p=0.0474175120011409

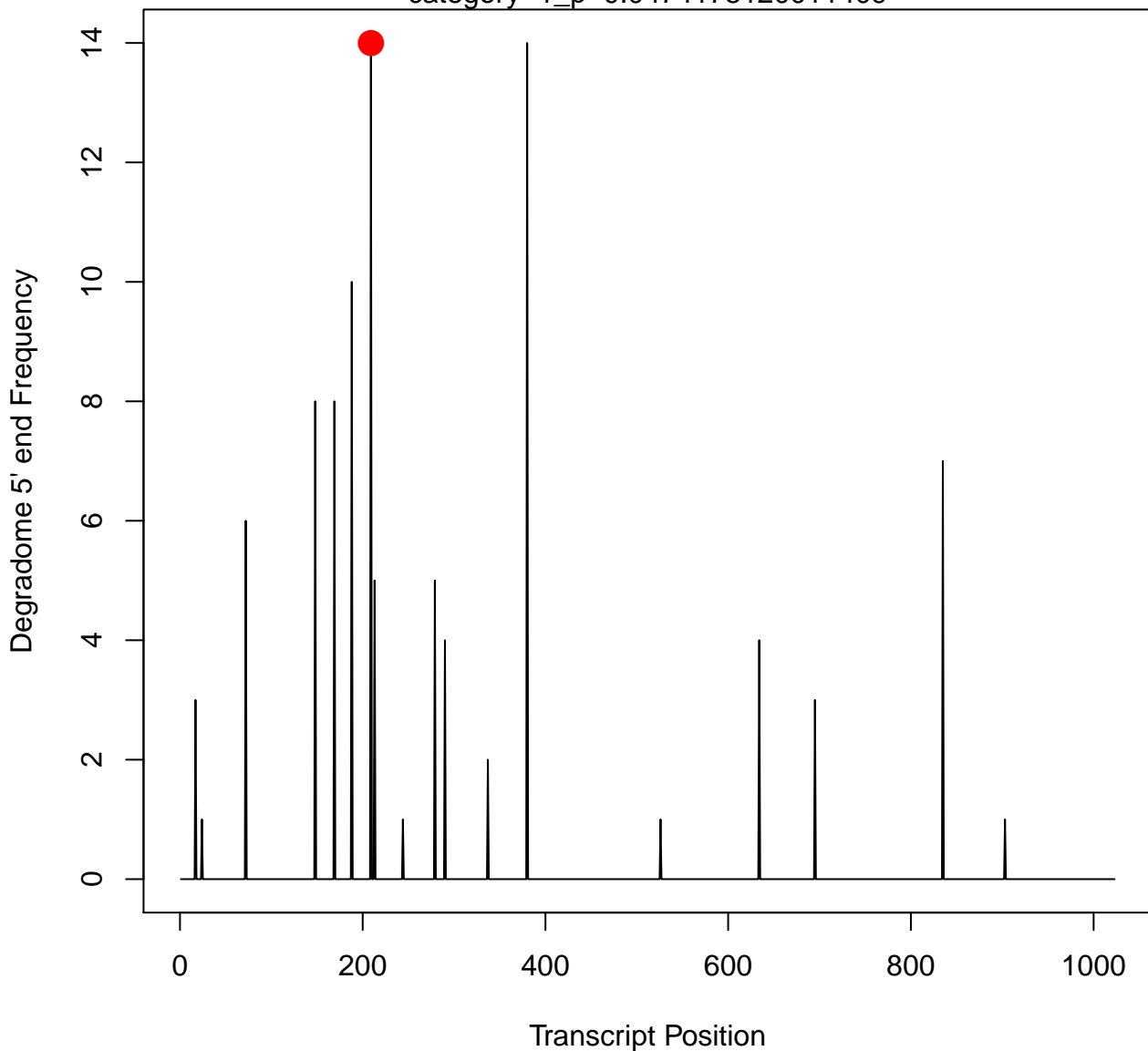

Supplement: Supplementary file 1 [file DataSheet_1.zip › The miRNA-target modules identified by the CleaveLand4/miR6275-5p_evm.model.LG05.3668_209_TPlot.pdf]

**T=evm.model.LG07.1712\_Q=miR7122a-5p\_S=64**

category=0\_p=0.00103797837257324

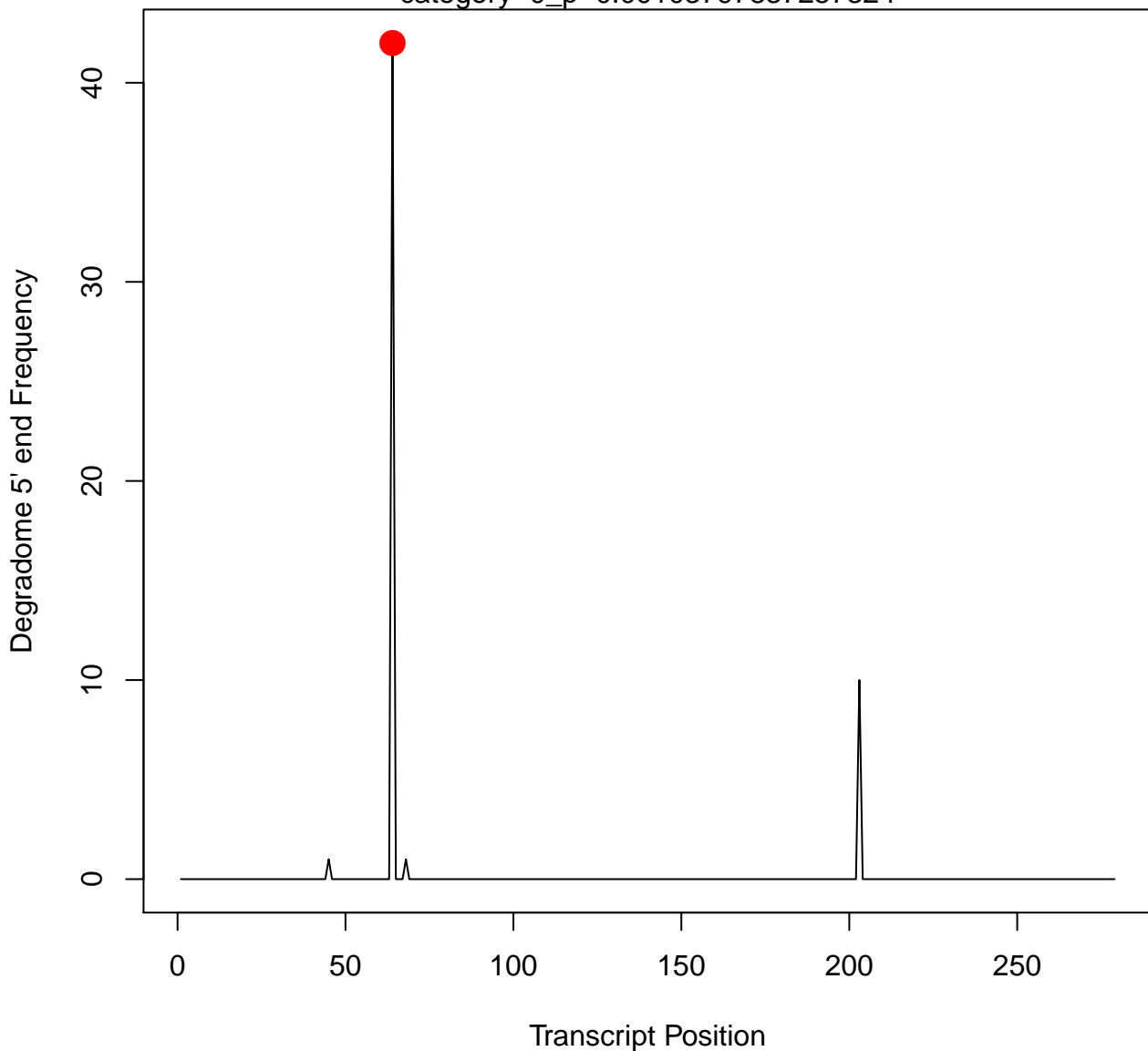

Supplement: Supplementary file 1 [file DataSheet_1.zip › The miRNA-target modules identified by the CleaveLand4/miR7122a-5p_evm.model.LG07.1712_64_TPlot.pdf]

**T=evm.model.LG04.3334\_Q=miRN11-5p\_S=880**

category=2\_p=0.0345432623396535

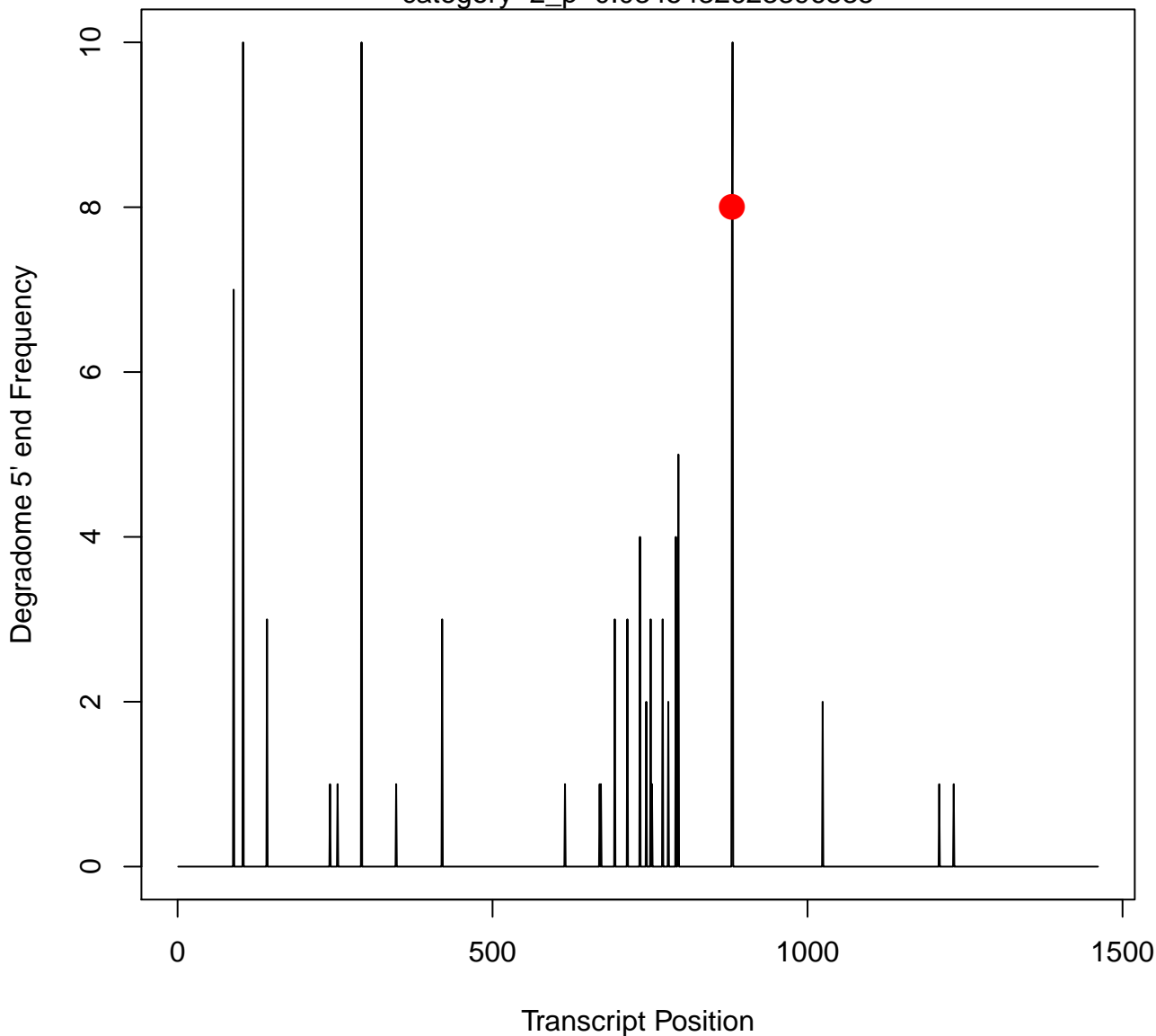

Supplement: Supplementary file 1 [file DataSheet_1.zip › The miRNA-target modules identified by the CleaveLand4/miRN11-5p_evm.model.LG04.3334_880_TPlot.pdf]

**T=evm.model.LG04.3338\_Q=miRN11-5p\_S=994**

category=2\_p=0.0260209548157995

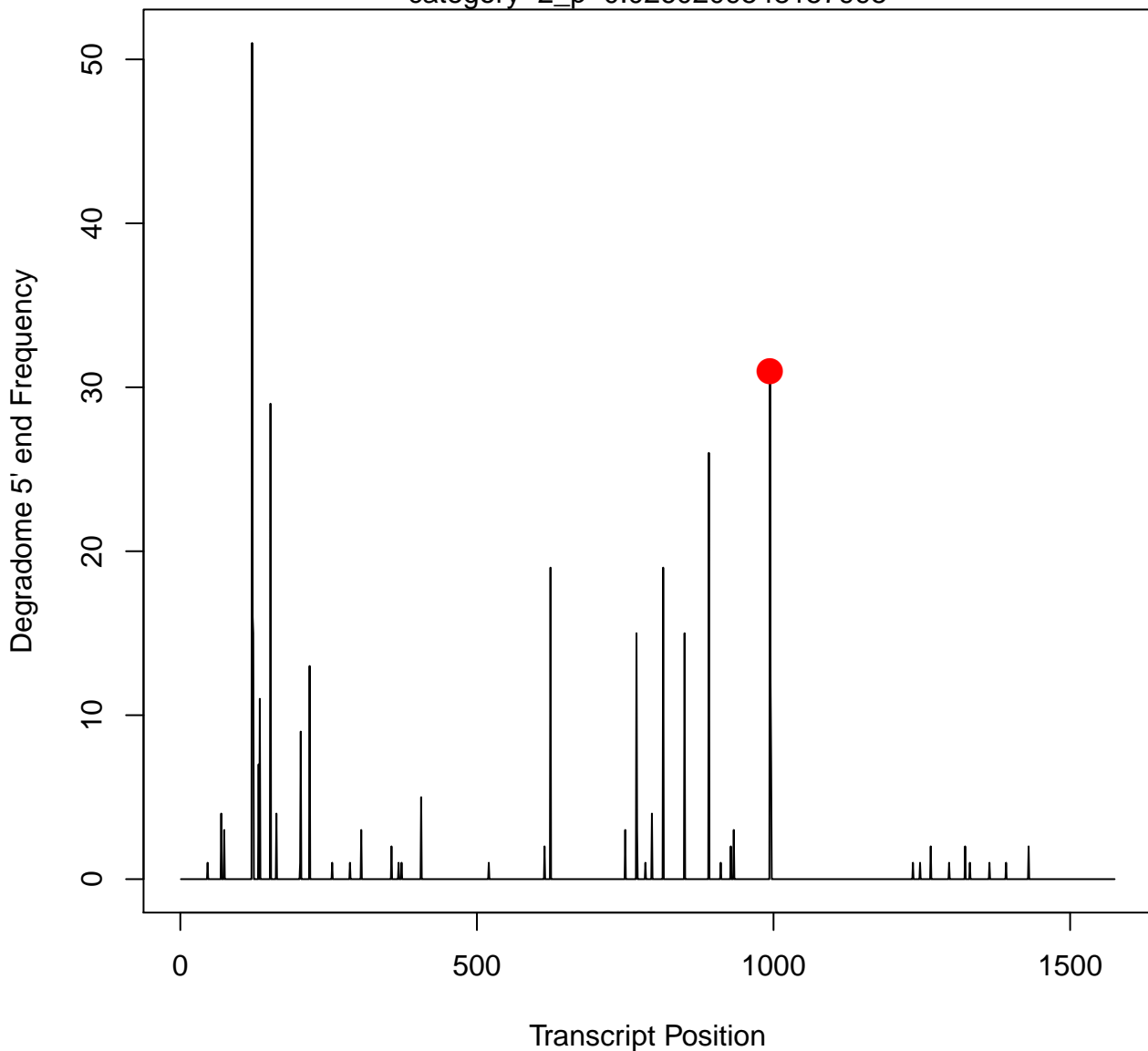

Supplement: Supplementary file 1 [file DataSheet_1.zip › The miRNA-target modules identified by the CleaveLand4/miRN11-5p_evm.model.LG04.3338_994_TPlot.pdf]

**T=evm.model.LG04.3339\_Q=miRN11-5p\_S=1084**

category=2\_p=0.042990999752487

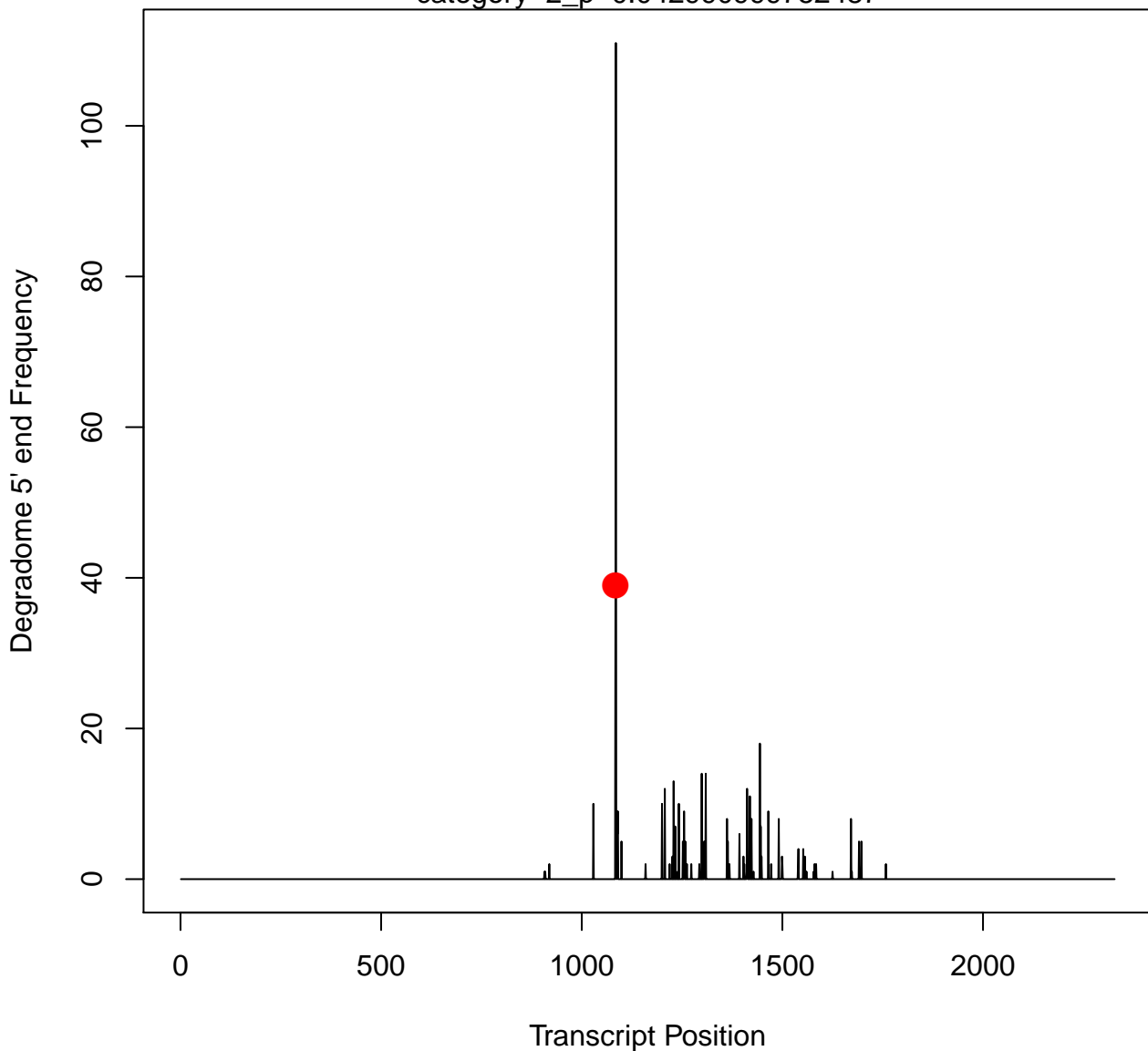

Supplement: Supplementary file 1 [file DataSheet_1.zip › The miRNA-target modules identified by the CleaveLand4/miRN11-5p_evm.model.LG04.3339_1084_TPlot.pdf]

**T=evm.model.LG05.3240\_Q=miRN11-5p\_S=976**

category=2\_p=0.0174234189334928

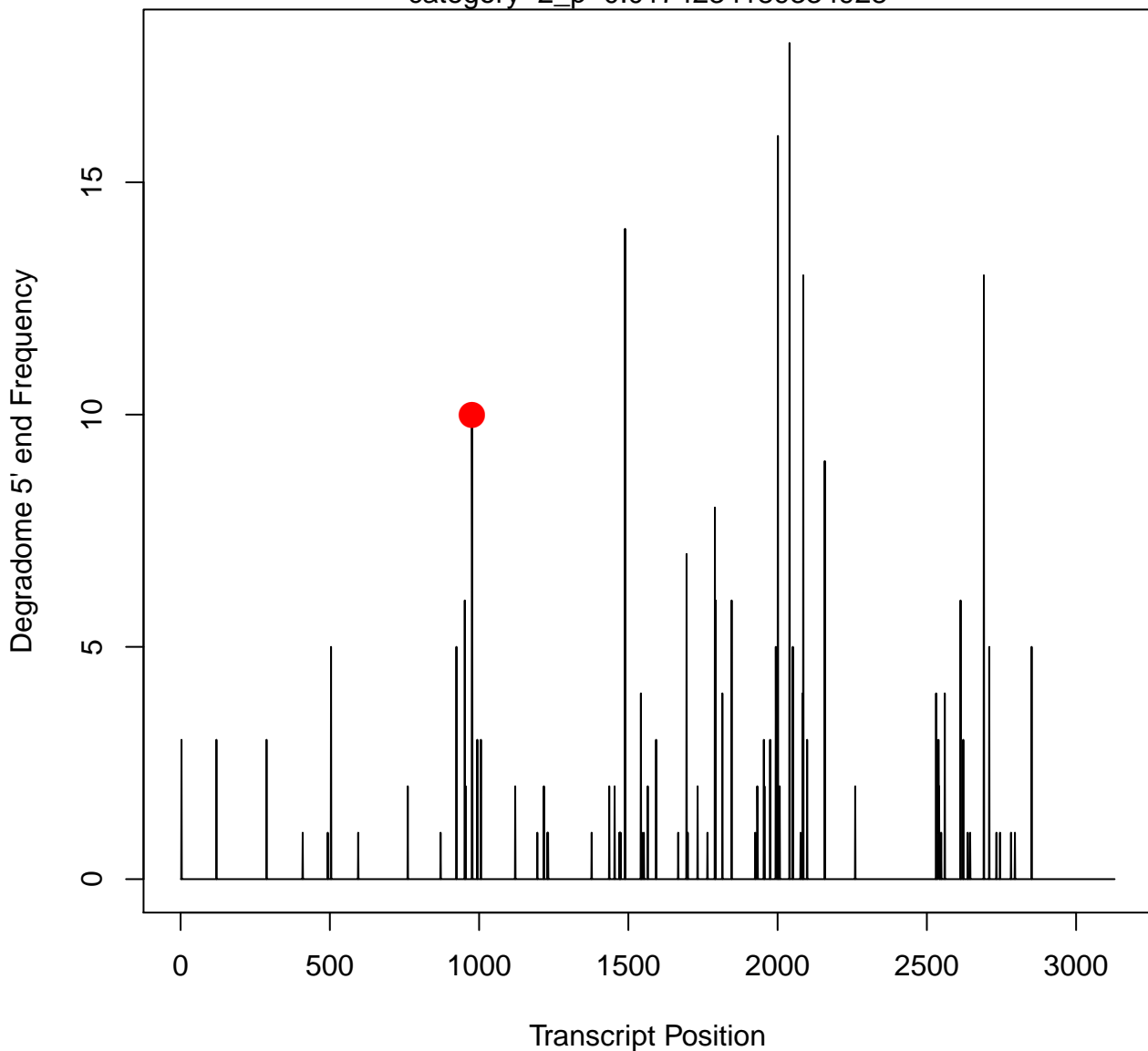

Supplement: Supplementary file 1 [file DataSheet_1.zip › The miRNA-target modules identified by the CleaveLand4/miRN11-5p_evm.model.LG05.3240_976_TPlot.pdf]

**T=evm.model.LG06.1993\_Q=miRN11-5p\_S=776**

category=1\_p=0.0429698226235549

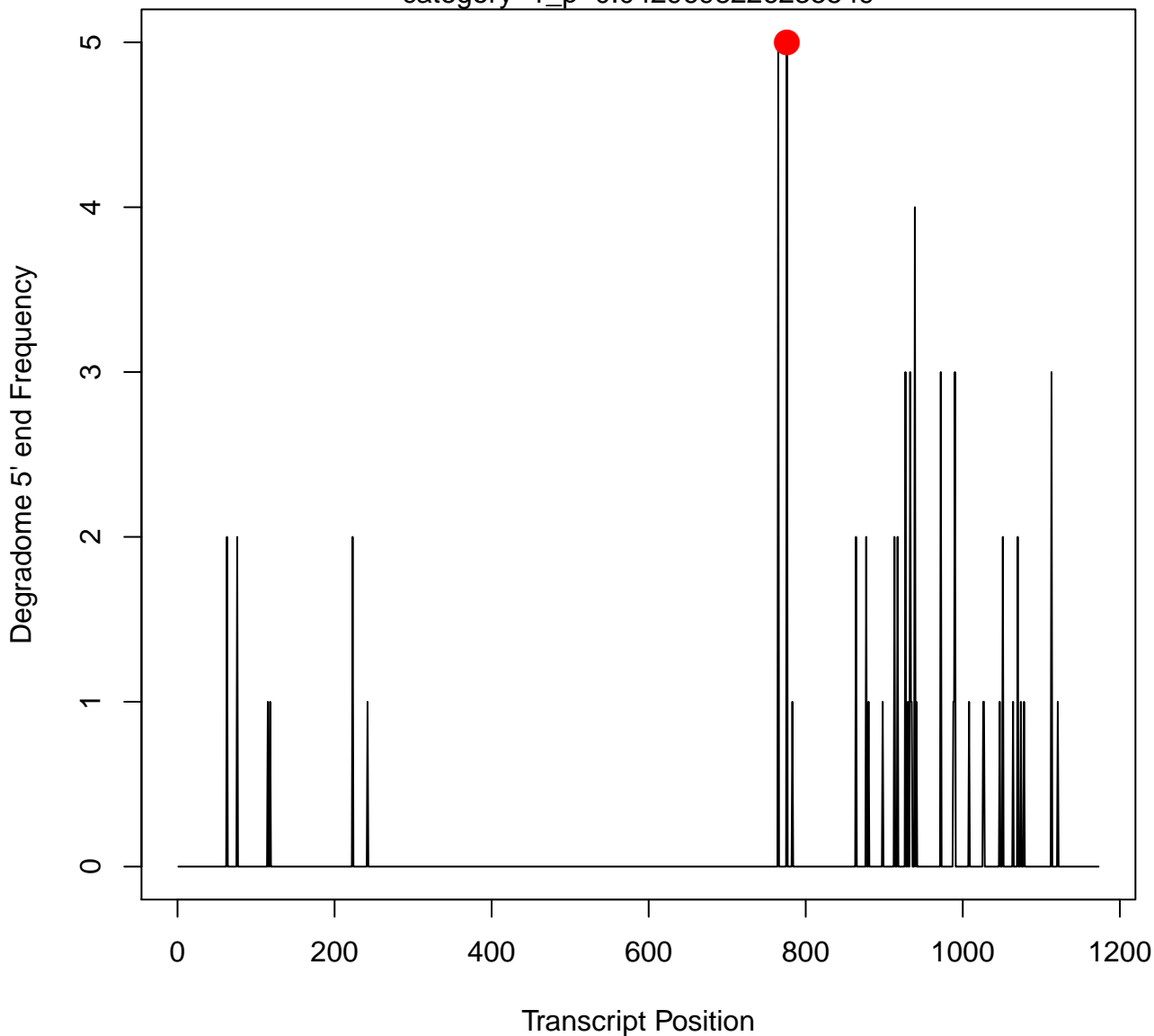

Supplement: Supplementary file 1 [file DataSheet_1.zip › The miRNA-target modules identified by the CleaveLand4/miRN11-5p_evm.model.LG06.1993_776_TPlot.pdf]

**T=evm.model.Contig1.87\_Q=miRN14-5p\_S=446**

category=3\_p=0.0477069564524738

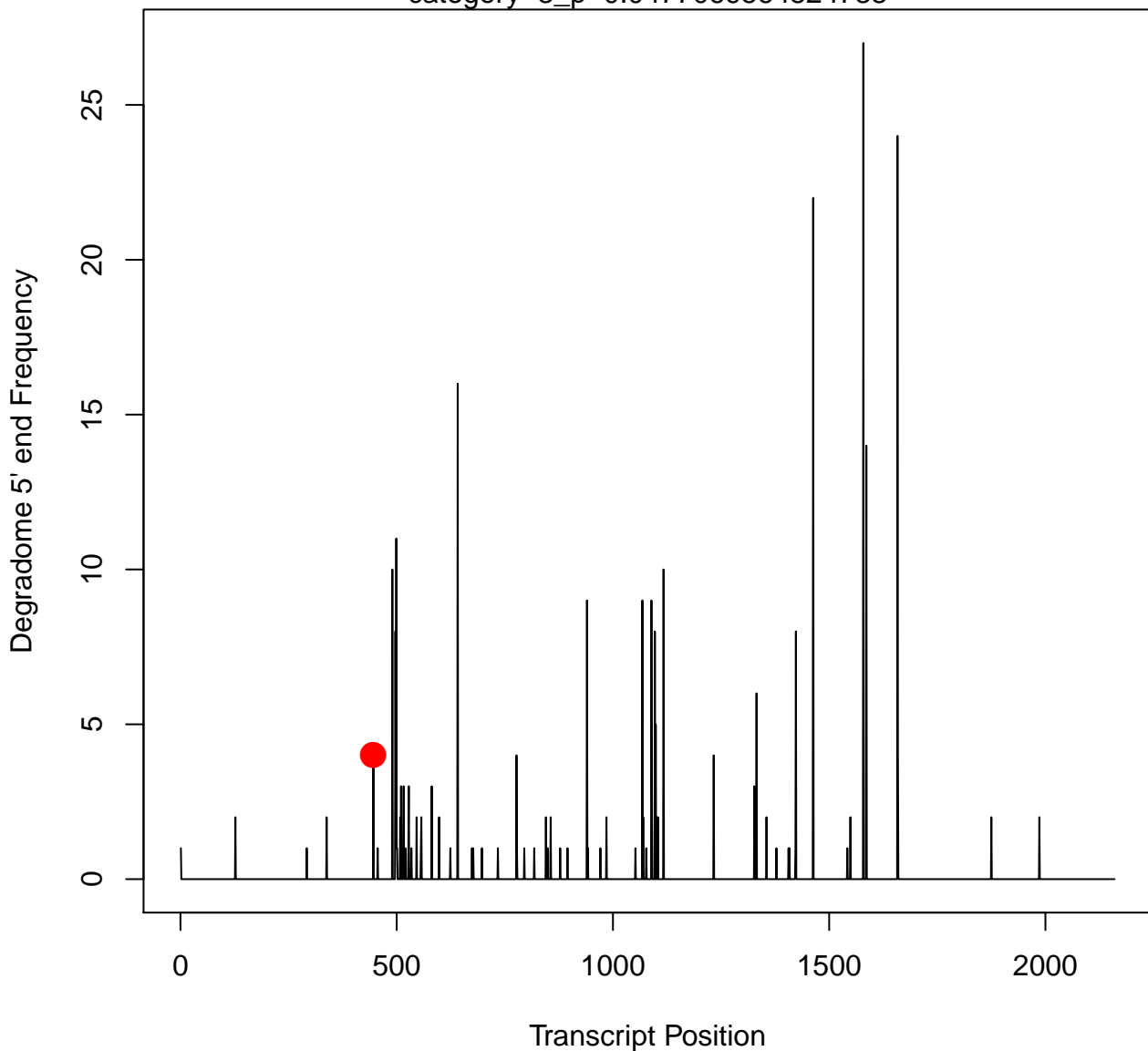

Supplement: Supplementary file 1 [file DataSheet_1.zip › The miRNA-target modules identified by the CleaveLand4/miRN14-5p_evm.model.Contig1.87_446_TPlot.pdf]

**T=evm.model.LG01.1279\_Q=miRN14-5p\_S=344**

category=0\_p=0.0241097620029824

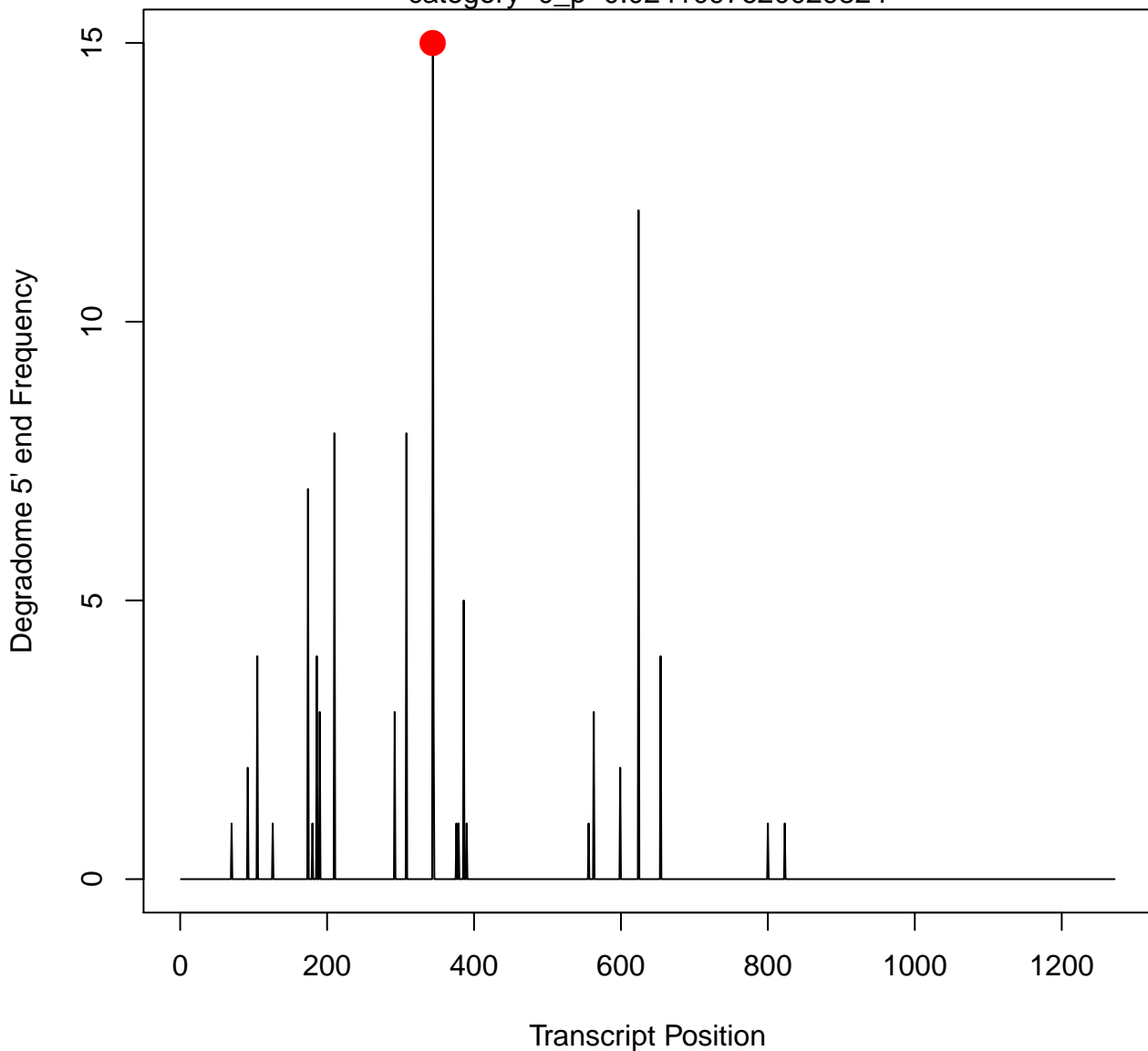

Supplement: Supplementary file 1 [file DataSheet_1.zip › The miRNA-target modules identified by the CleaveLand4/miRN14-5p_evm.model.LG01.1279_344_TPlot.pdf]

**T=evm.model.LG04.3773\_Q=miRN14-5p\_S=419**

category=0\_p=0.00051912393111464

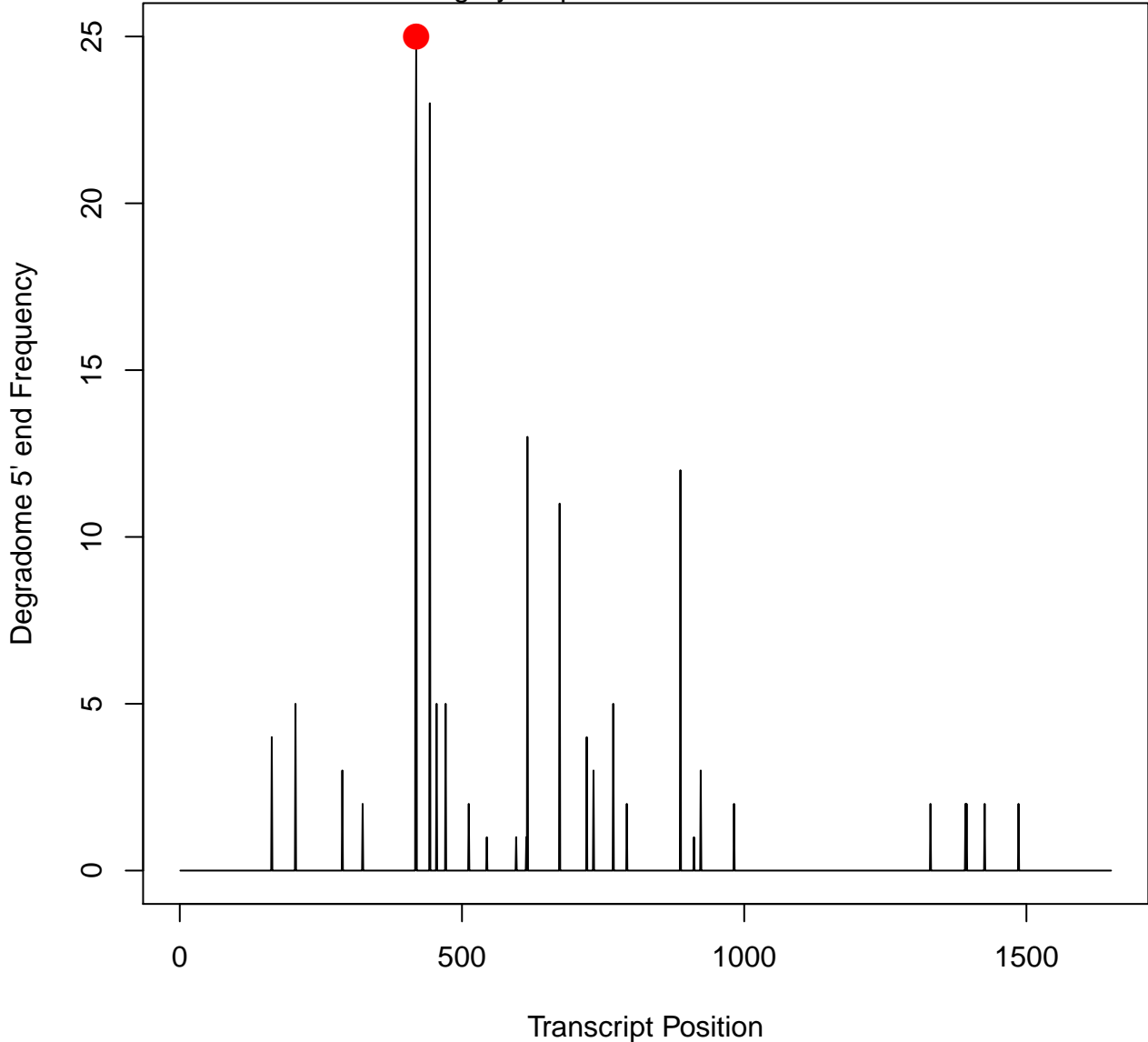

Supplement: Supplementary file 1 [file DataSheet_1.zip › The miRNA-target modules identified by the CleaveLand4/miRN14-5p_evm.model.LG04.3773_419_TPlot.pdf]

**T=evm.model.LG05.3563\_Q=miRN14-5p\_S=104**

category=4\_p=0.0423765939260693

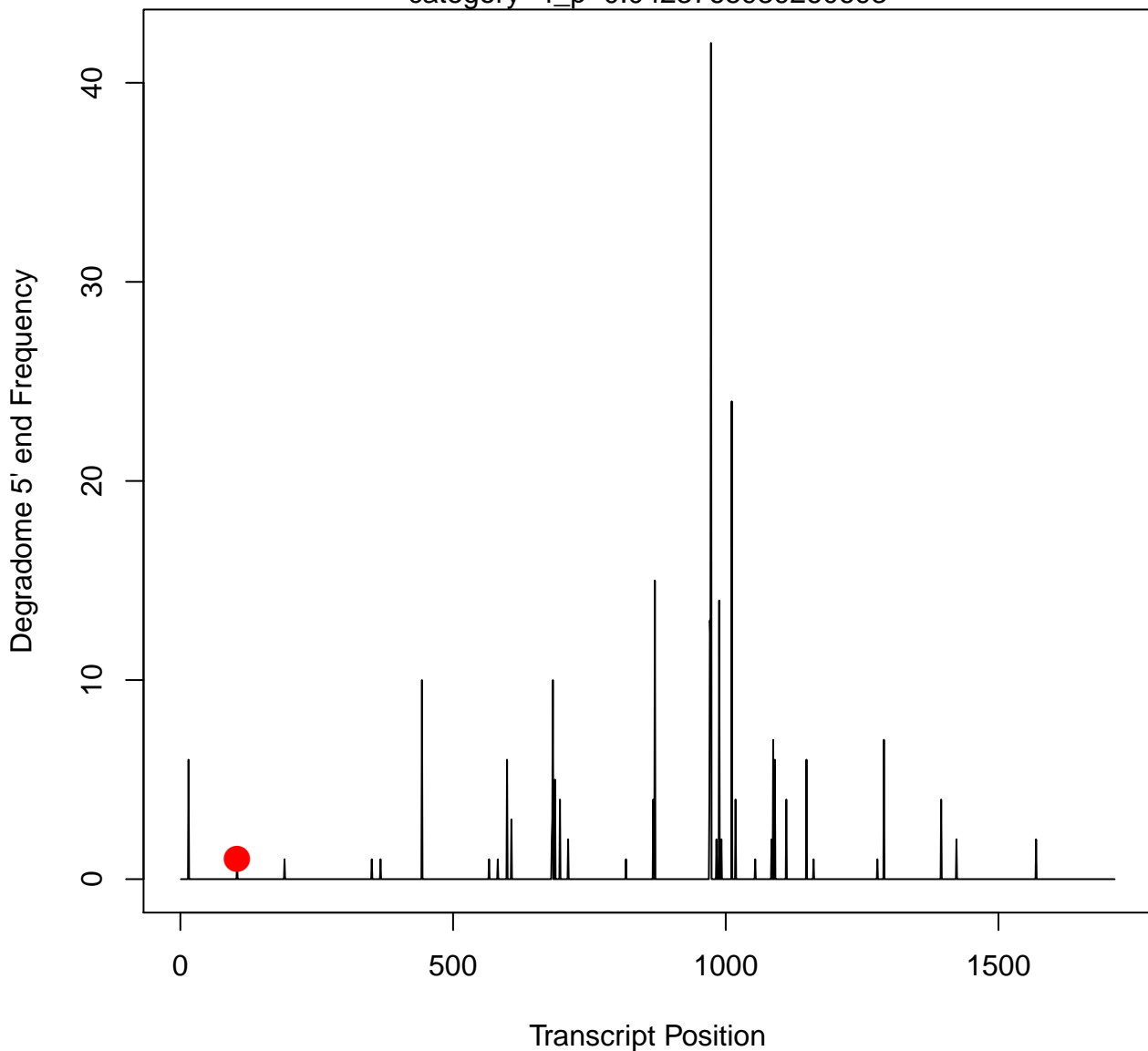

Supplement: Supplementary file 1 [file DataSheet_1.zip › The miRNA-target modules identified by the CleaveLand4/miRN14-5p_evm.model.LG05.3563_104_TPlot.pdf]

**T=evm.model.LG05.368\_Q=miRN14-5p\_S=356**

category=0\_p=0.00827372235029444

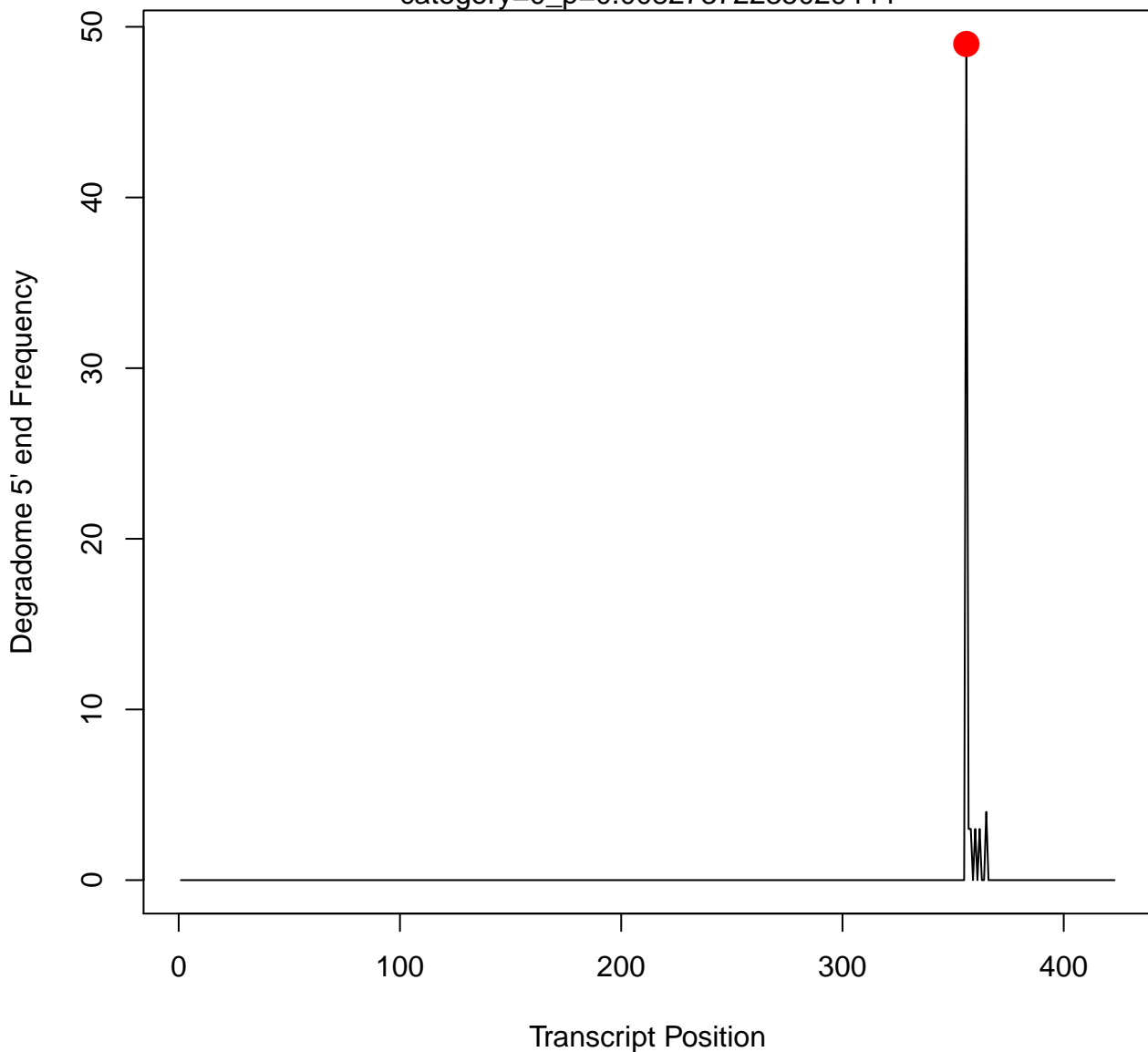

Supplement: Supplementary file 1 [file DataSheet_1.zip › The miRNA-target modules identified by the CleaveLand4/miRN14-5p_evm.model.LG05.368_356_TPlot.pdf]

**T=evm.model.LG06.3352\_Q=miRN14-5p\_S=443**

category=3\_p=0.0241449679652586

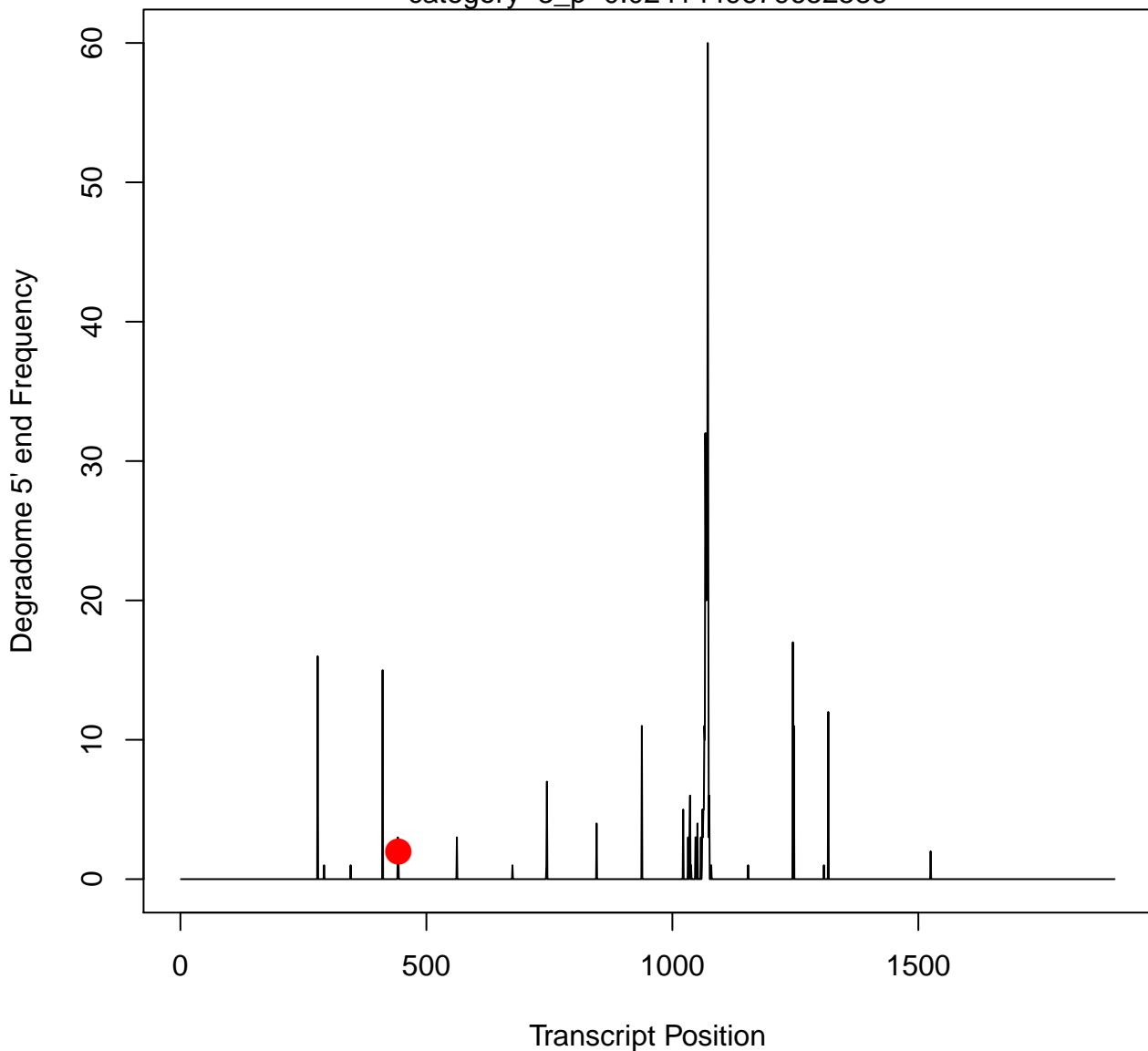

Supplement: Supplementary file 1 [file DataSheet_1.zip › The miRNA-target modules identified by the CleaveLand4/miRN14-5p_evm.model.LG06.3352_443_TPlot.pdf]

**T=evm.model.LG08.1784\_Q=miRN14-5p\_S=458**

category=4\_p=0.0423765939260693

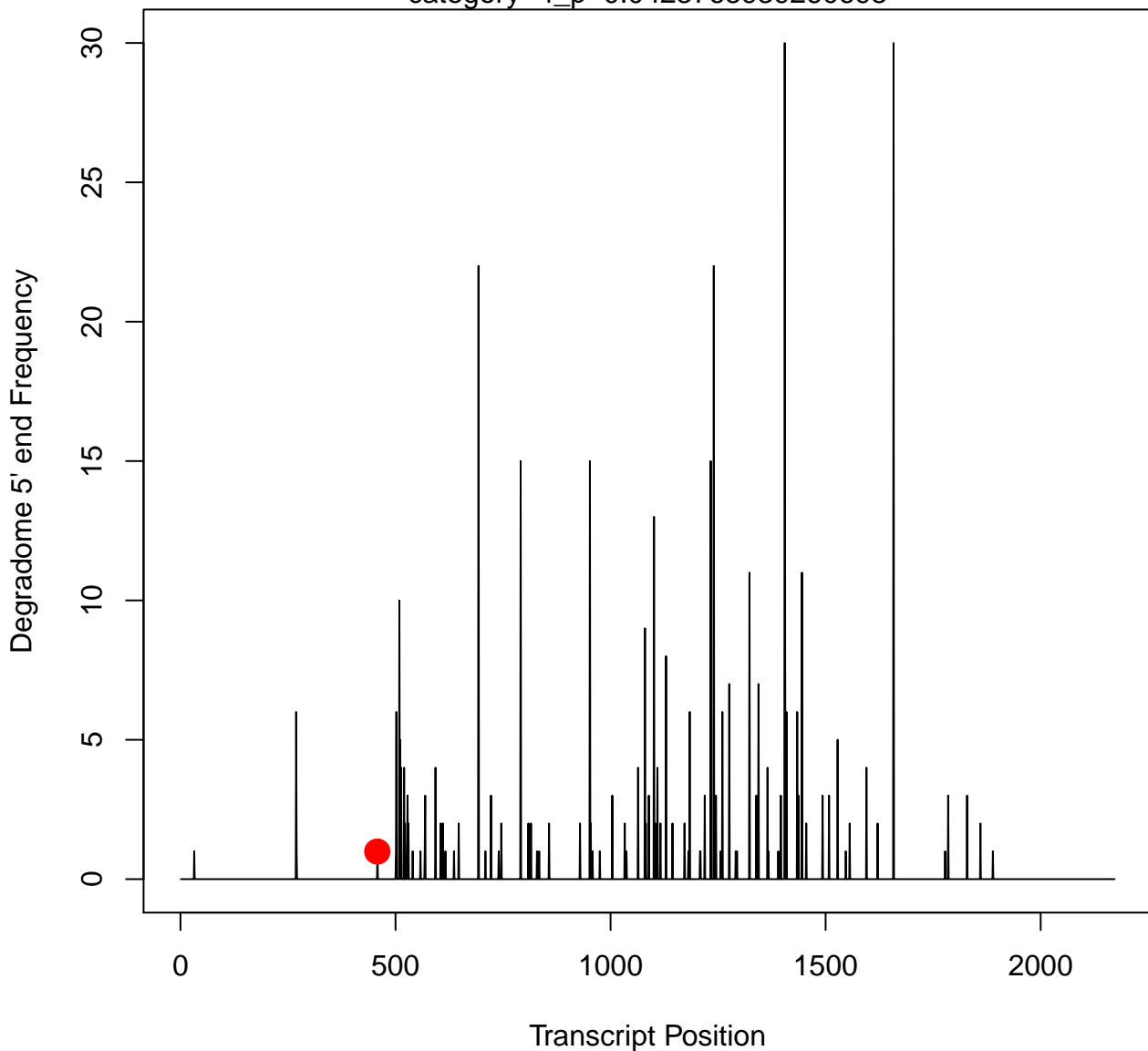

Supplement: Supplementary file 1 [file DataSheet_1.zip › The miRNA-target modules identified by the CleaveLand4/miRN14-5p_evm.model.LG08.1784_458_TPlot.pdf]

**T=evm.model.LG02.5093\_Q=miRN15-3p\_S=1615**

category=0\_p=0.00930311277800744

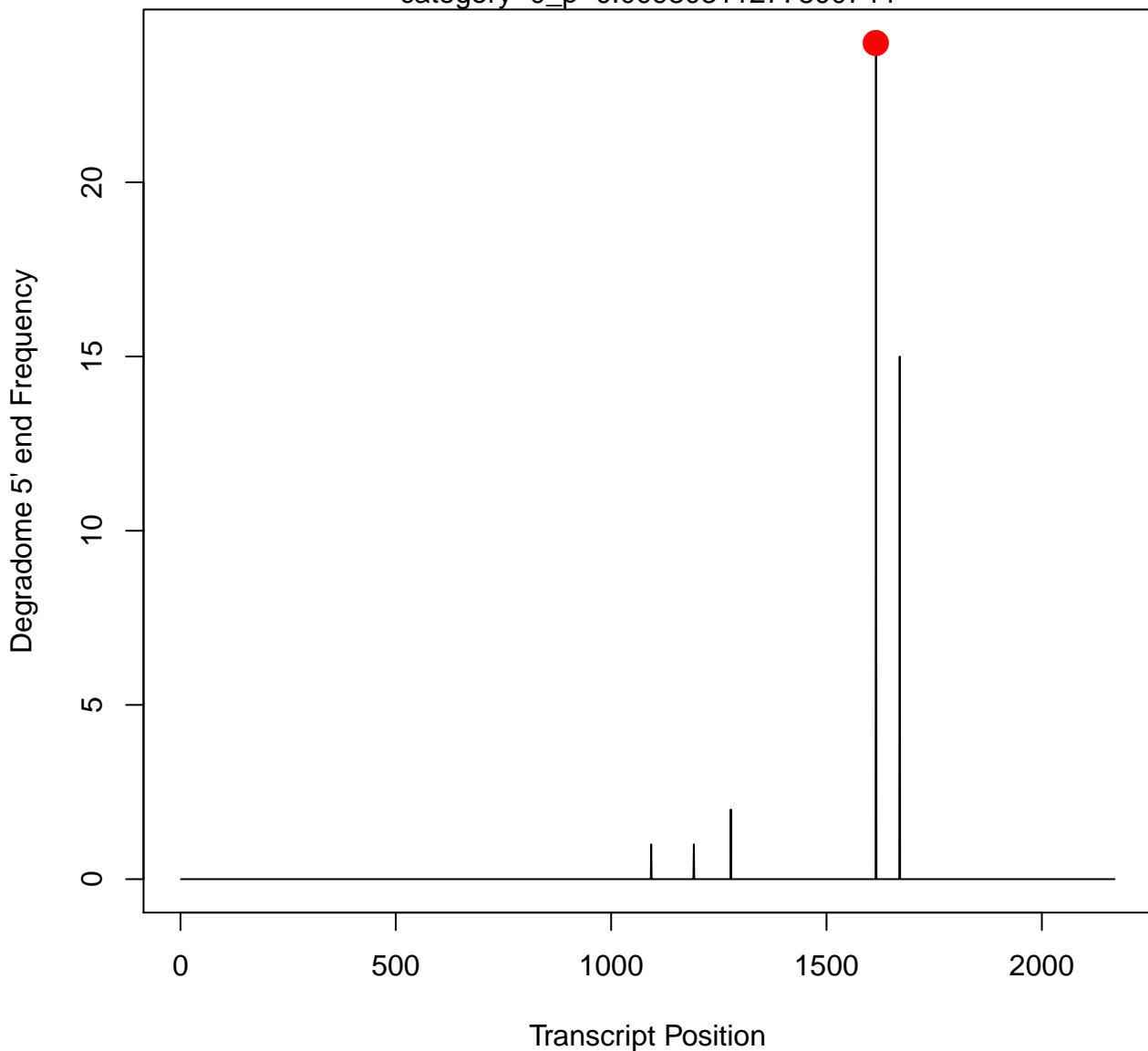

Supplement: Supplementary file 1 [file DataSheet_1.zip › The miRNA-target modules identified by the CleaveLand4/miRN15-3p_evm.model.LG02.5093_1615_TPlot.pdf]

**T=evm.model.LG04.3535\_Q=miRN17-3p\_S=4908**

category=2\_p=0.0345432623396535

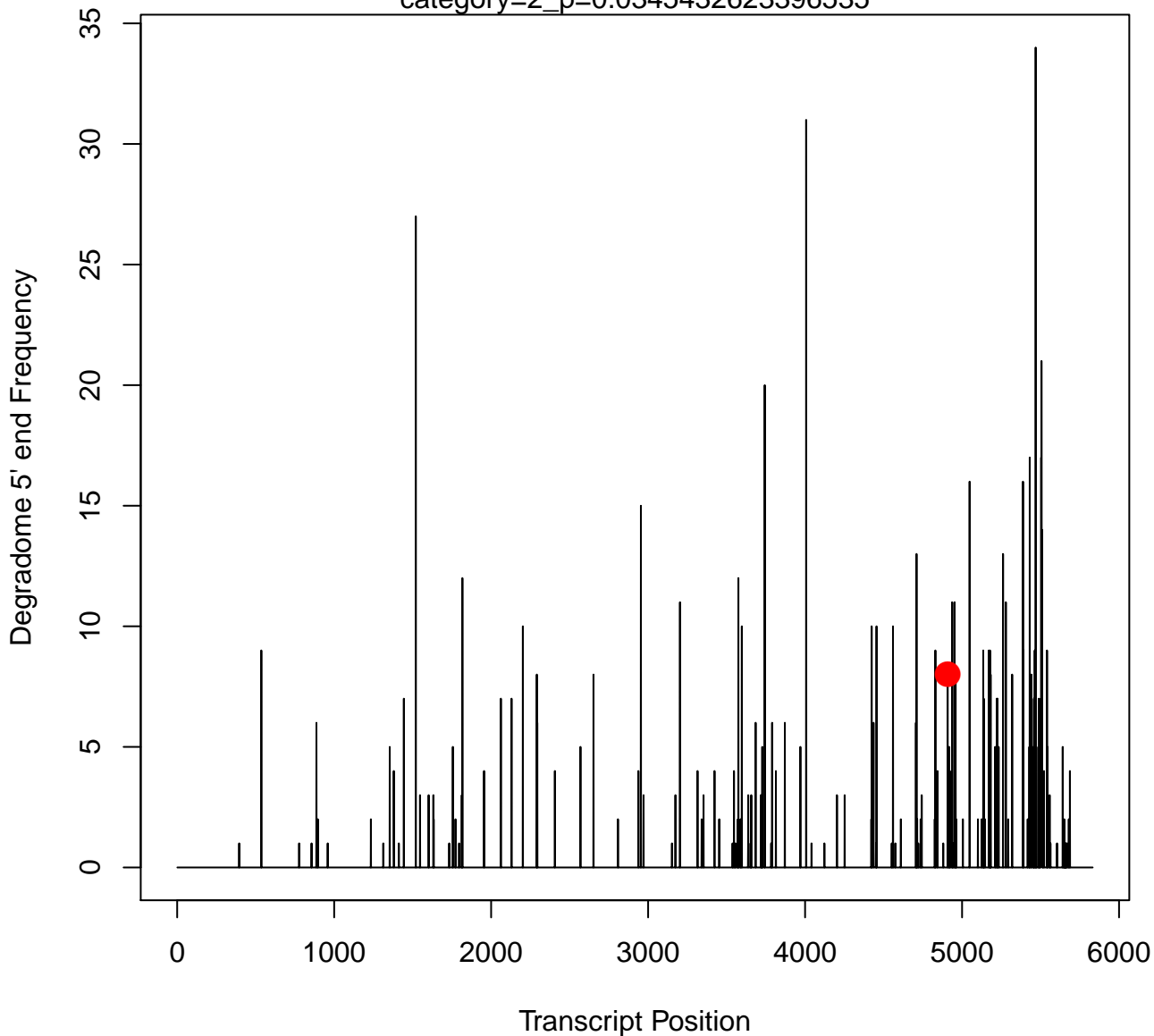

Supplement: Supplementary file 1 [file DataSheet_1.zip › The miRNA-target modules identified by the CleaveLand4/miRN17-3p_evm.model.LG04.3535_4908_TPlot.pdf]

**T=evm.model.LG08.2016\_Q=miRN18-5p\_S=709**

category=3\_p=0.0241449679652586

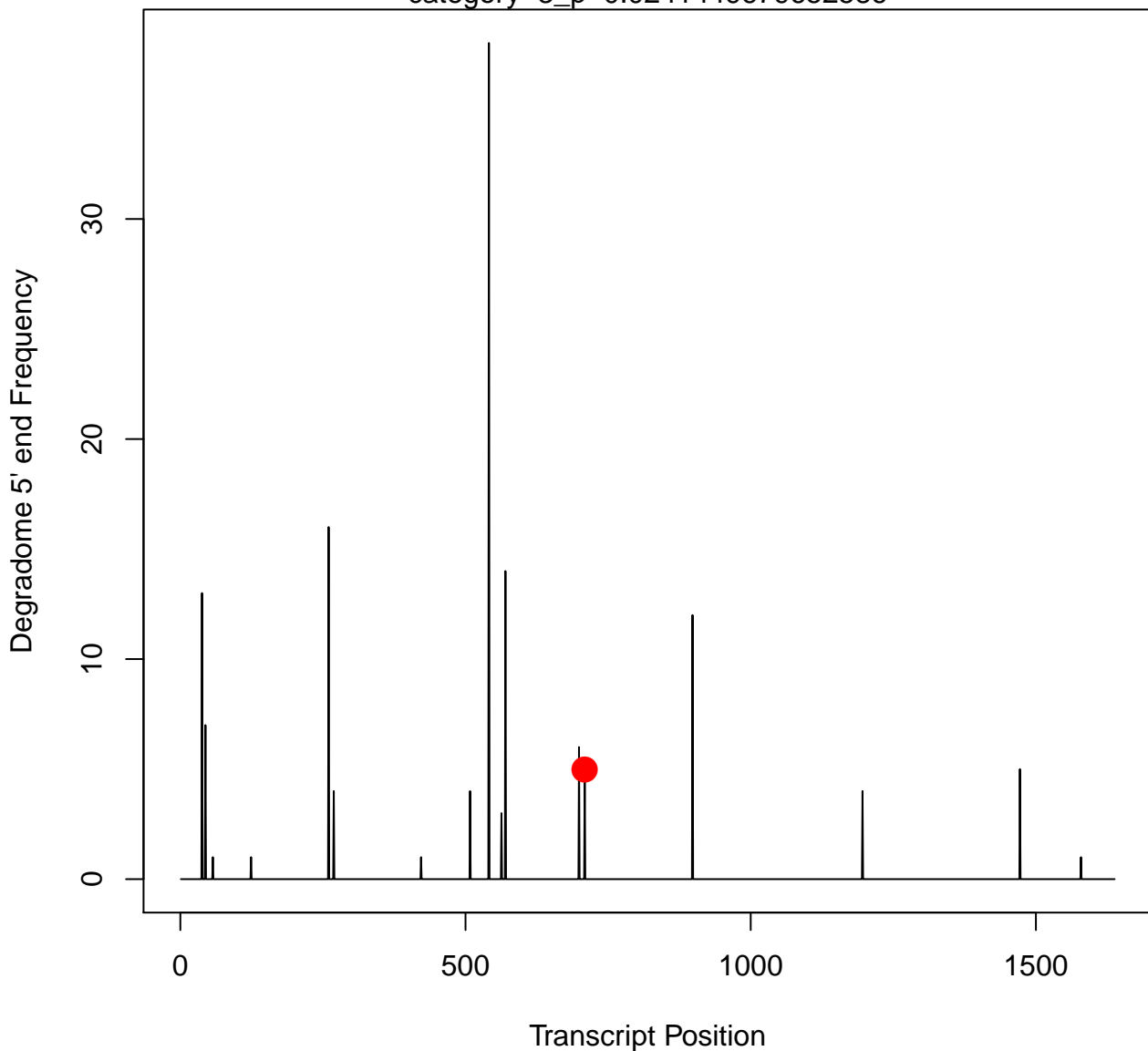

Supplement: Supplementary file 1 [file DataSheet_1.zip › The miRNA-target modules identified by the CleaveLand4/miRN18-5p_evm.model.LG08.2016_709_TPlot.pdf]

**T=evm.model.LG01.4718\_Q=miRN19a-5p\_S=2113**

category=3\_p=0.0477069564524738

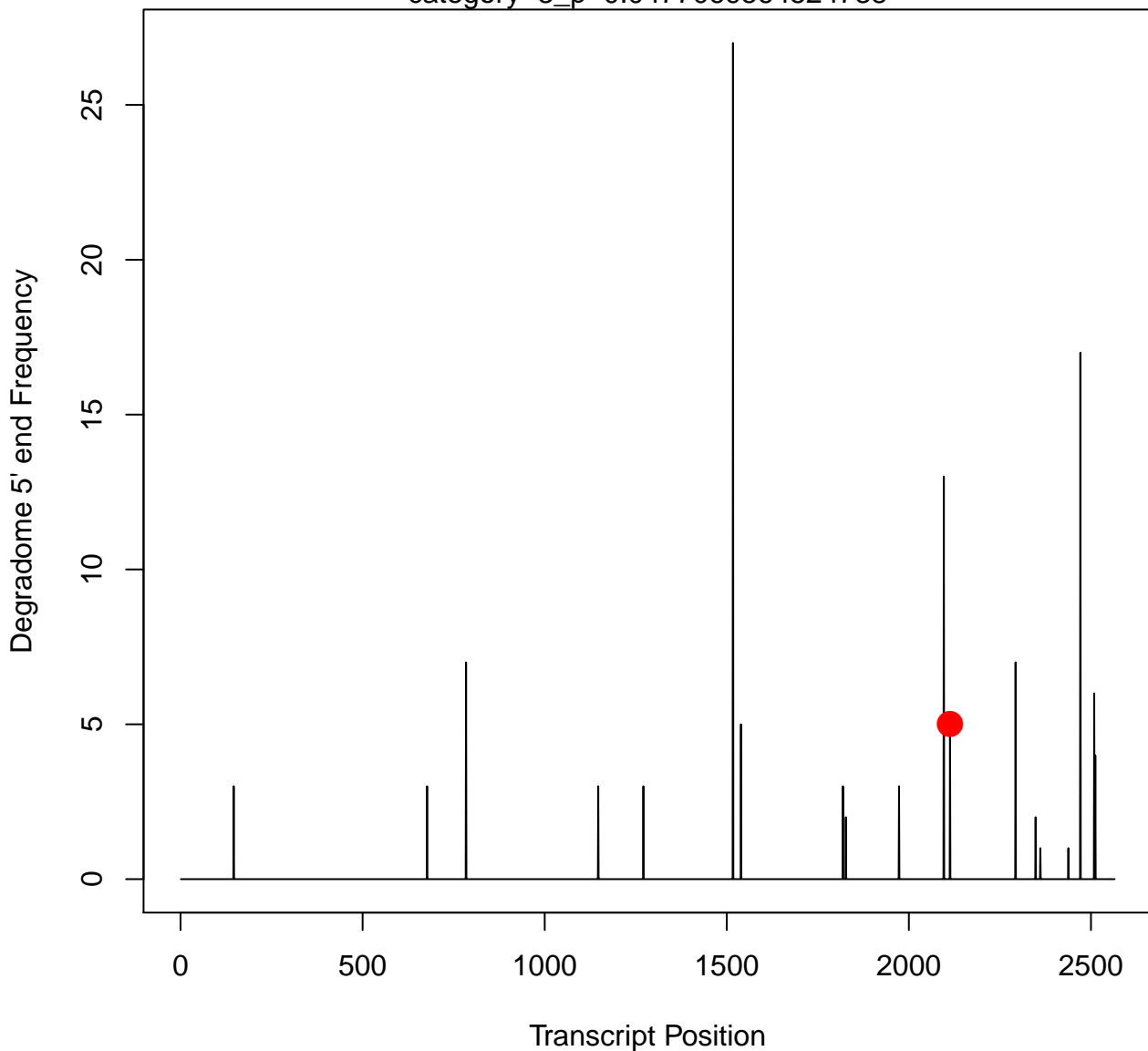

Supplement: Supplementary file 1 [file DataSheet_1.zip › The miRNA-target modules identified by the CleaveLand4/miRN19a-5p_evm.model.LG01.4718_2113_TPlot.pdf]

**T=evm.model.LG01.3352\_Q=miRN19b-3p\_S=728**

category=4\_p=0.0423765939260693

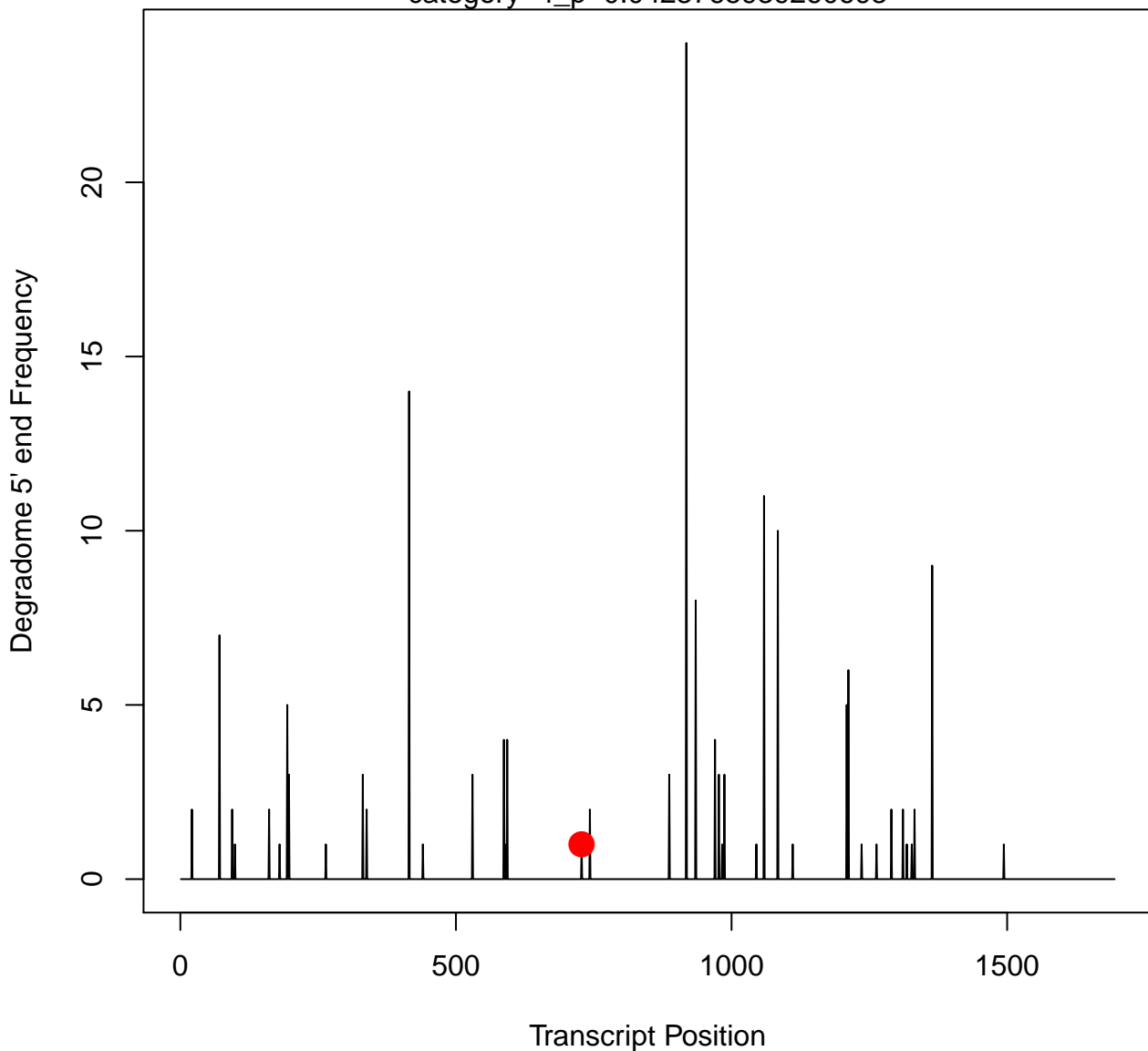

Supplement: Supplementary file 1 [file DataSheet_1.zip › The miRNA-target modules identified by the CleaveLand4/miRN19b-3p_evm.model.LG01.3352_728_TPlot.pdf]

**T=evm.model.LG06.2304\_Q=miRN19b-5p\_S=640**

category=2\_p=0.0174234189334928

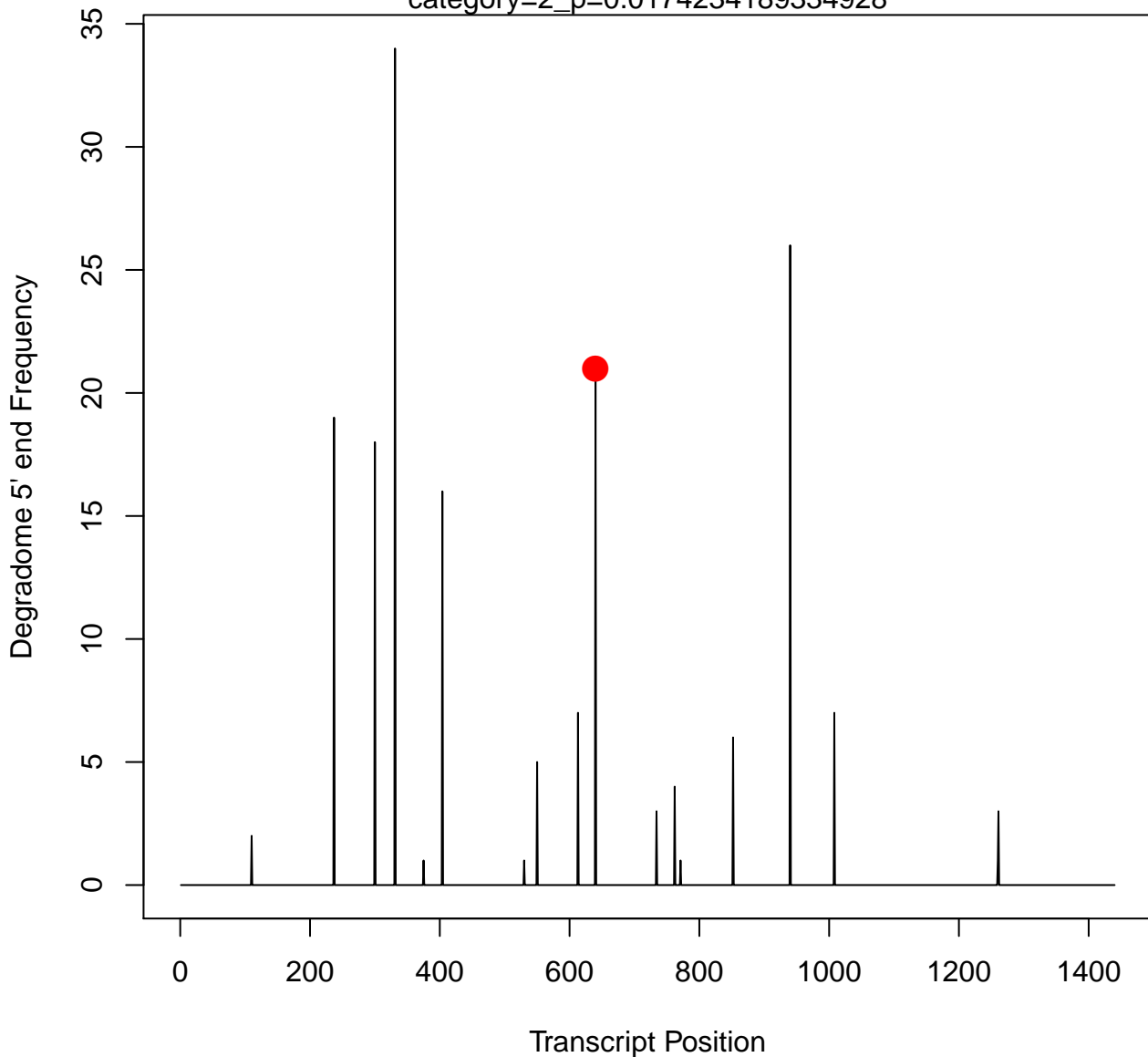

Supplement: Supplementary file 1 [file DataSheet_1.zip › The miRNA-target modules identified by the CleaveLand4/miRN19b-5p_evm.model.LG06.2304_640_TPlot.pdf]

**T=evm.model.LG08.846\_Q=miRN1a-3p\_S=203**

category=0\_p=0.0159681557397247

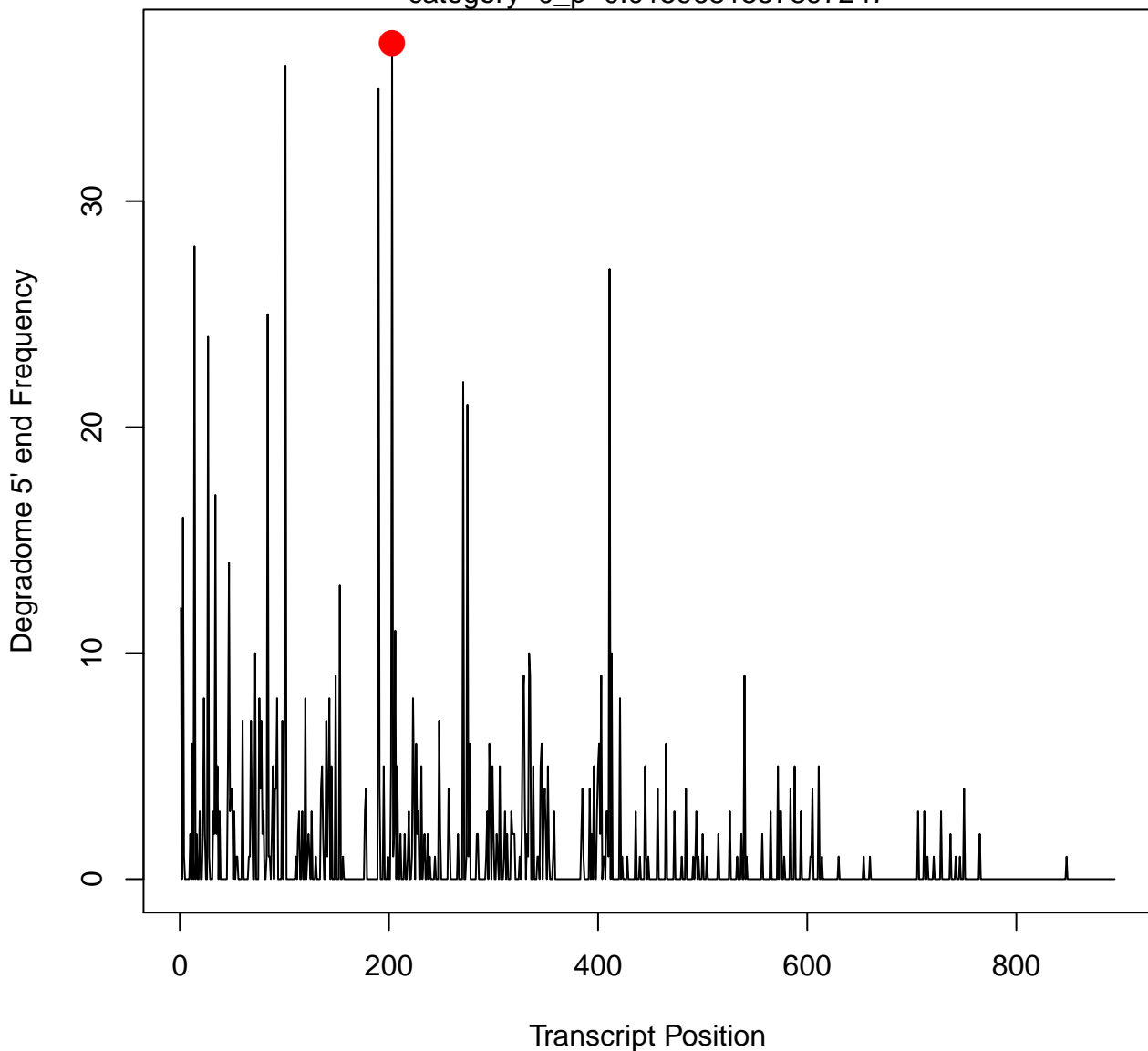

Supplement: Supplementary file 1 [file DataSheet_1.zip › The miRNA-target modules identified by the CleaveLand4/miRN1a-3p_evm.model.LG08.846_203_TPlot.pdf]

**T=evm.model.LG04.4552\_Q=miRN21-5p\_S=400**

category=2\_p=0.042990999752487

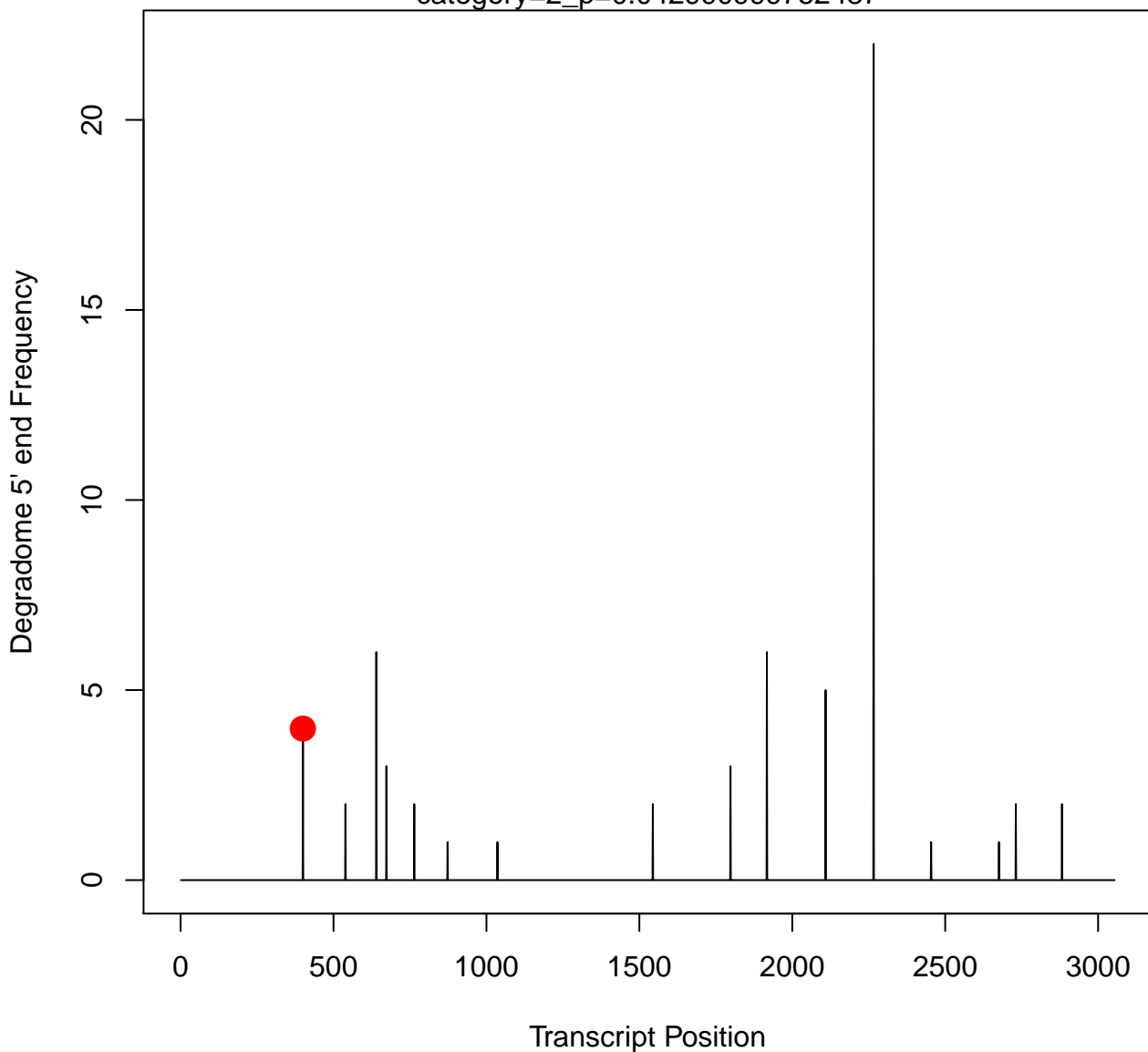

Supplement: Supplementary file 1 [file DataSheet_1.zip › The miRNA-target modules identified by the CleaveLand4/miRN21-5p_evm.model.LG04.4552_400_TPlot.pdf]

**T=evm.model.LG06.2301\_Q=miRN22-3p\_S=333**

category=4\_p=0.00862274555333054

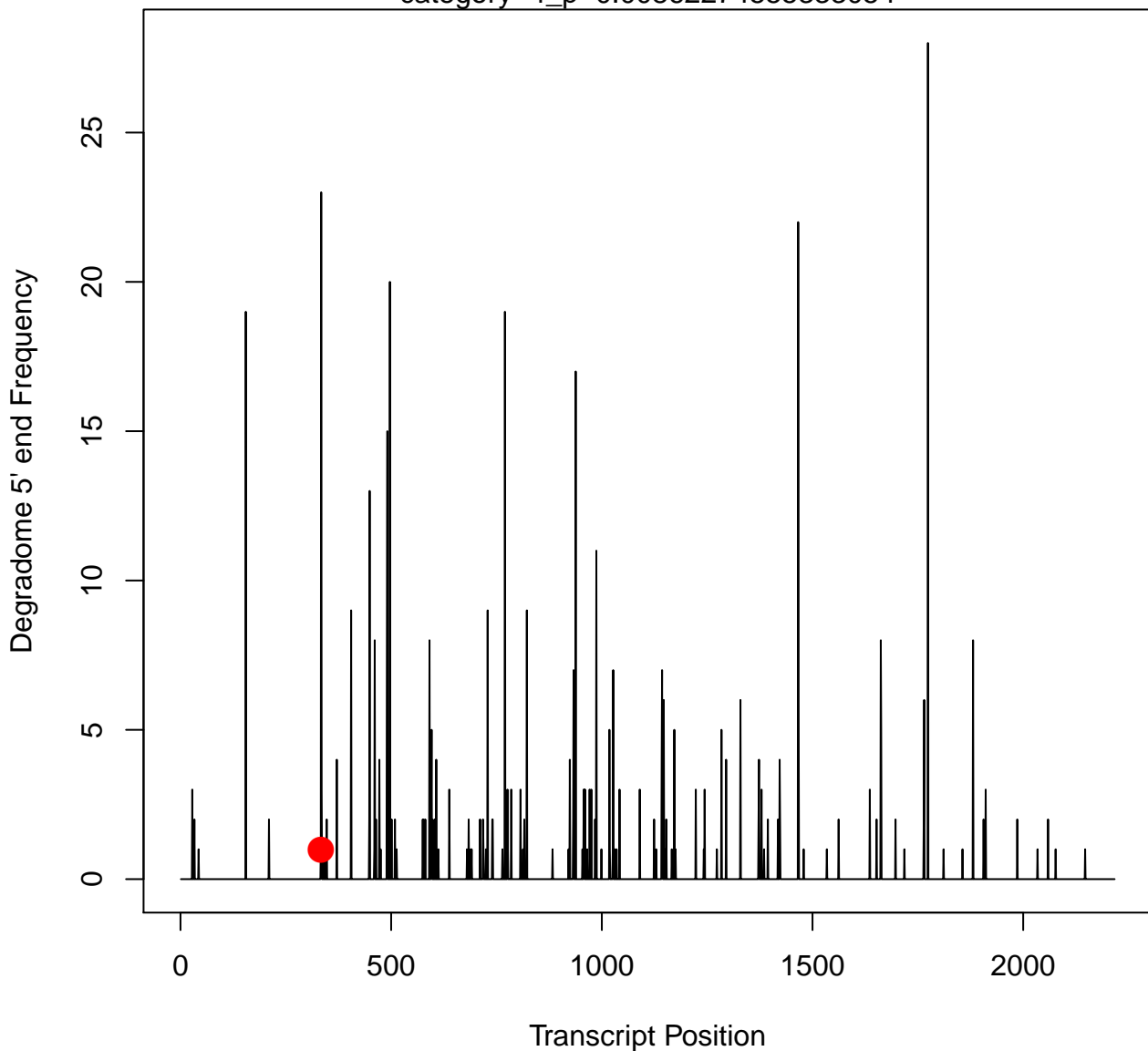

Supplement: Supplementary file 1 [file DataSheet_1.zip › The miRNA-target modules identified by the CleaveLand4/miRN22-3p_evm.model.LG06.2301_333_TPlot.pdf]

**T=evm.model.LG07.1189\_Q=miRN22-3p\_S=318**

category=2\_p=0.0174234189334928

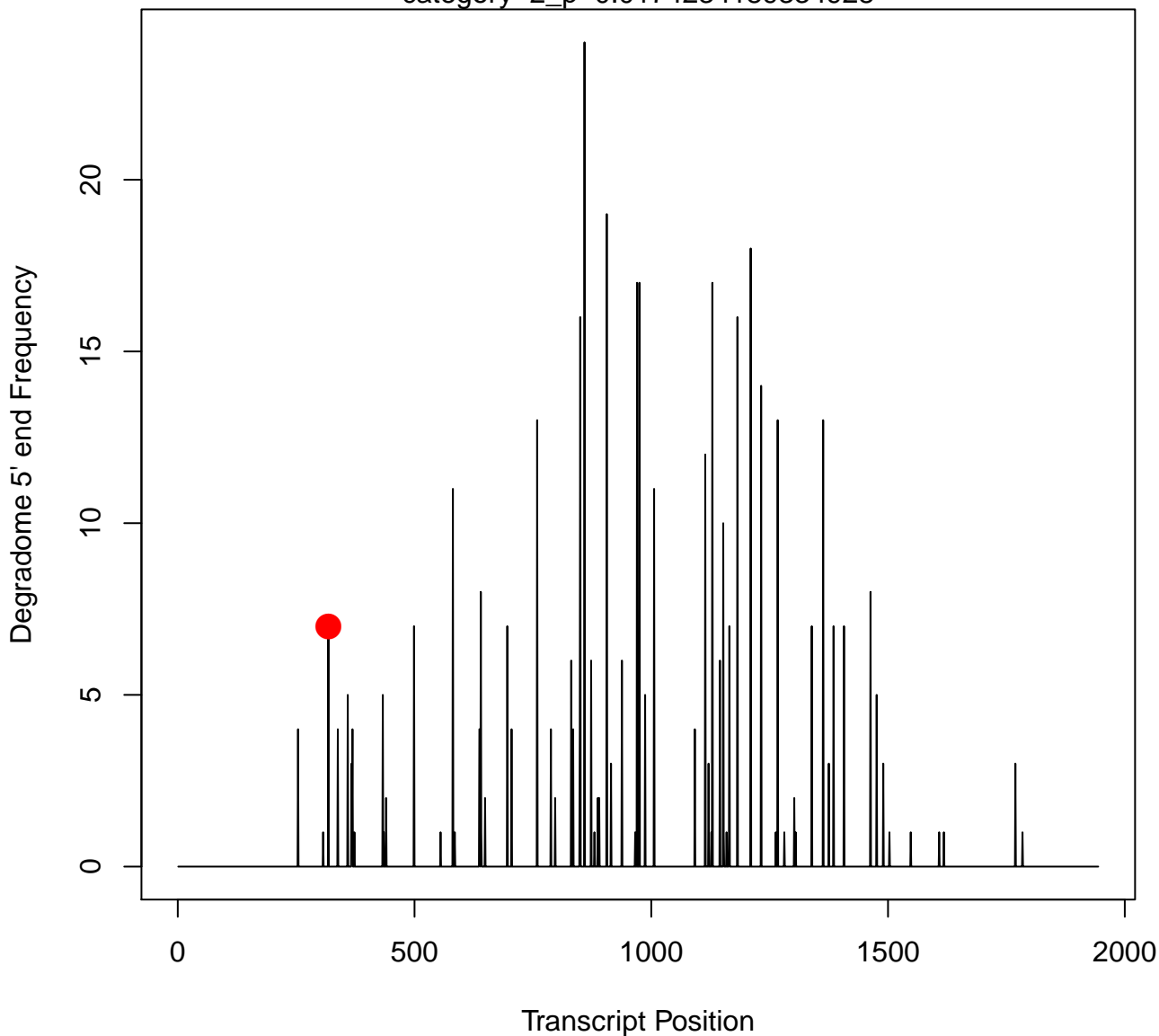

Supplement: Supplementary file 1 [file DataSheet_1.zip › The miRNA-target modules identified by the CleaveLand4/miRN22-3p_evm.model.LG07.1189_318_TPlot.pdf]

**T=evm.model.LG06.176\_Q=miRN24-5p\_S=78**

category=0\_p=0.0311783909995083

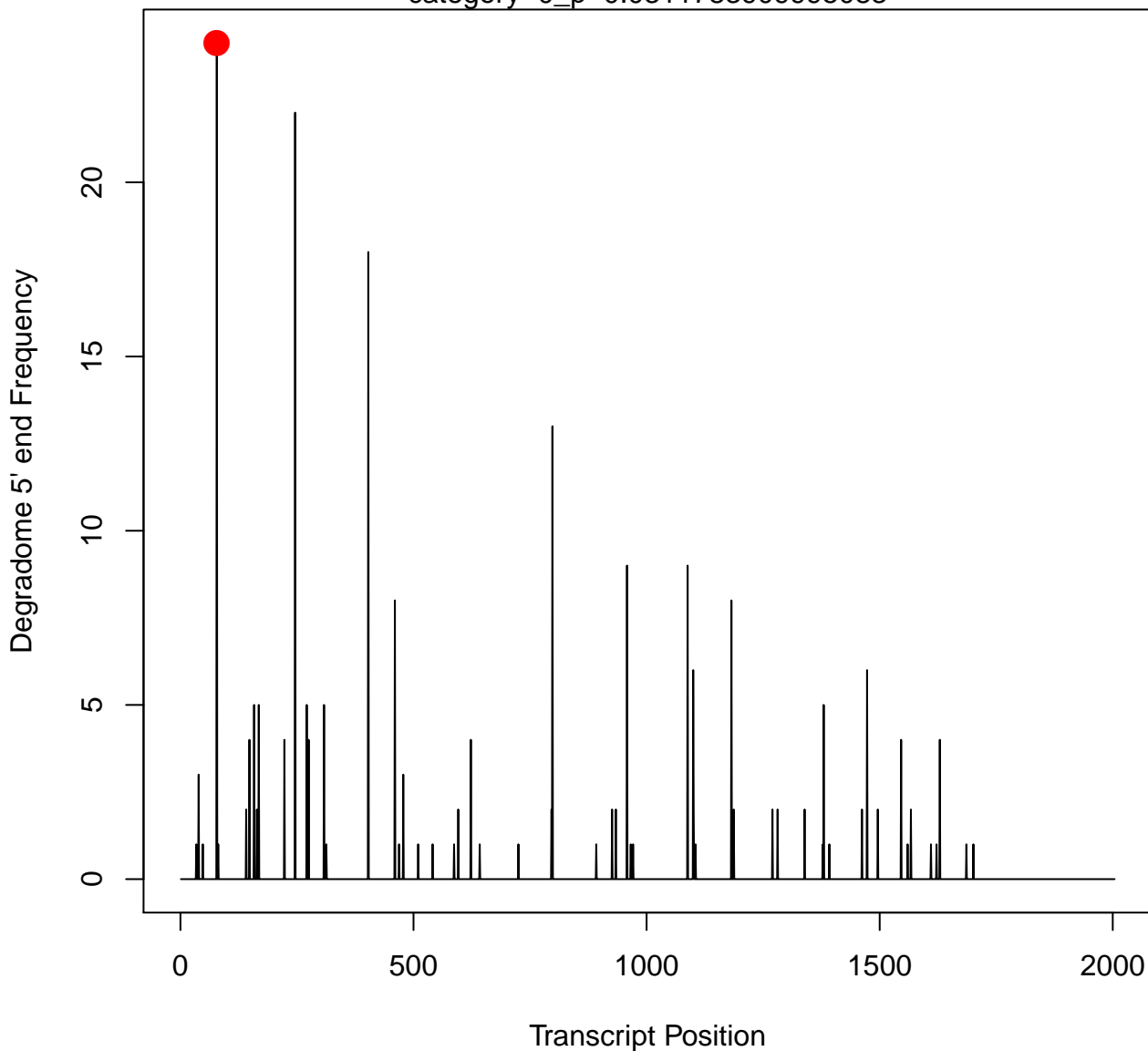

Supplement: Supplementary file 1 [file DataSheet_1.zip › The miRNA-target modules identified by the CleaveLand4/miRN24-5p_evm.model.LG06.176_78_TPlot.pdf]

**T=evm.model.LG06.3489\_Q=miRN27-3p\_S=992**

category=3\_p=0.0241449679652586

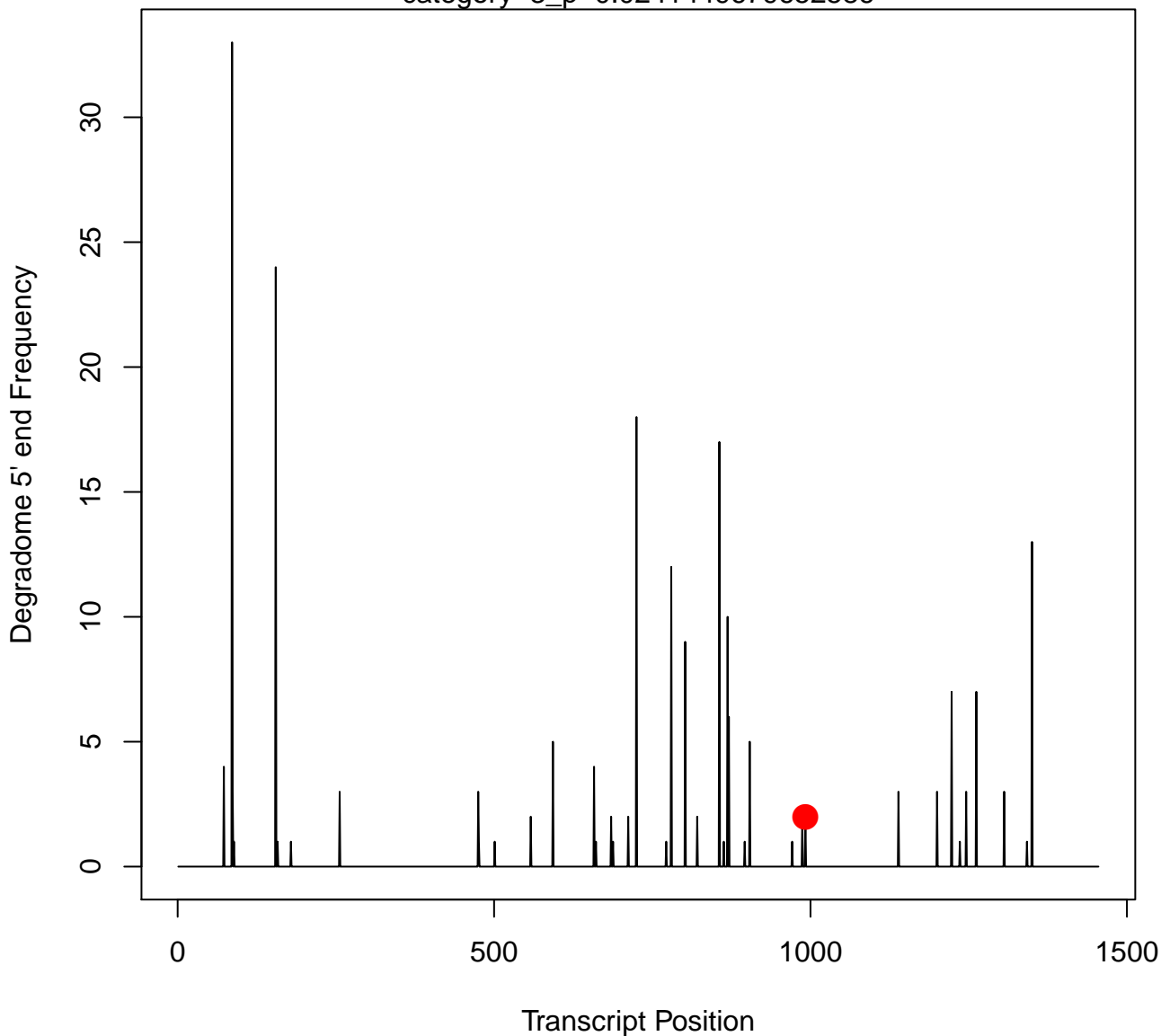

Supplement: Supplementary file 1 [file DataSheet_1.zip › The miRNA-target modules identified by the CleaveLand4/miRN27-3p_evm.model.LG06.3489_992_TPlot.pdf]

**T=evm.model.LG08.1593\_Q=miRN27-3p\_S=701**

category=0\_p=0.00466242549475071

Degradome 5' end Frequency

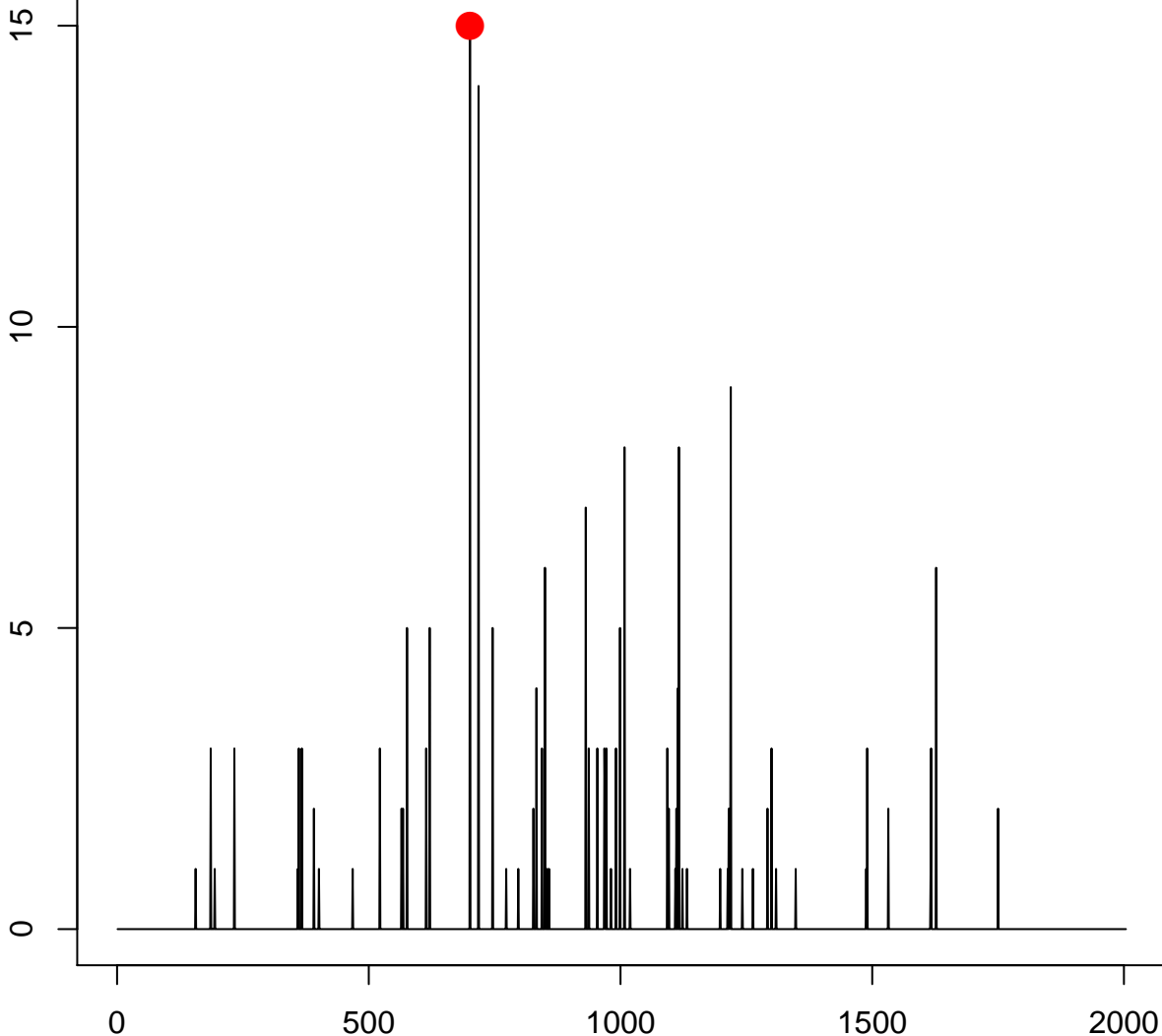

Transcript Position

Supplement: Supplementary file 1 [file DataSheet_1.zip › The miRNA-target modules identified by the CleaveLand4/miRN27-3p_evm.model.LG08.1593_701_TPlot.pdf]

**T=evm.model.LG07.596\_Q=miRN2a-5p\_S=71**

category=0\_p=0.00103797837257324

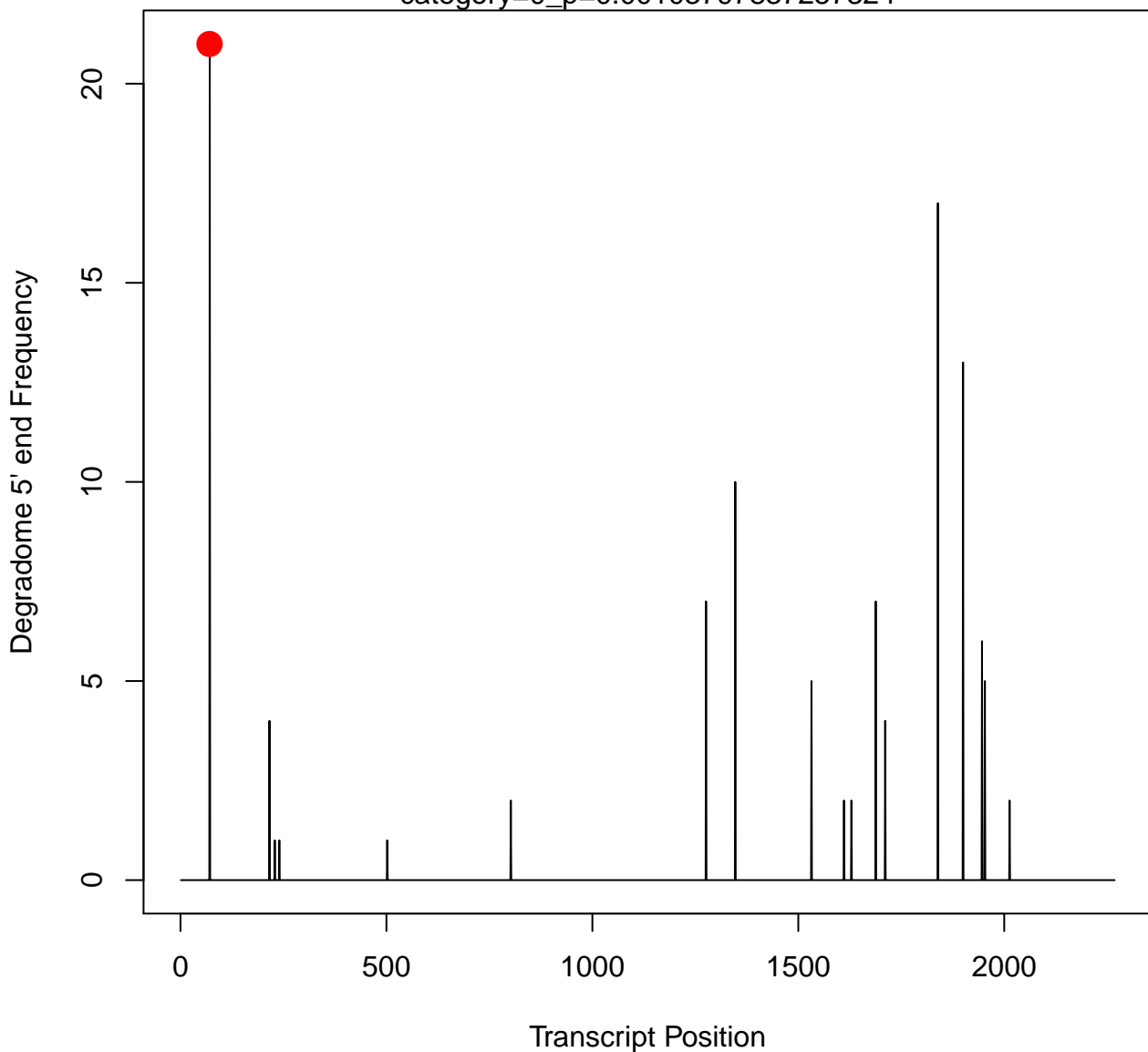

Supplement: Supplementary file 1 [file DataSheet_1.zip › The miRNA-target modules identified by the CleaveLand4/miRN2a-5p_evm.model.LG07.596_71_TPlot.pdf]

**T=evm.model.LG04.3983\_Q=miRN2b-3p\_S=95**

category=2\_p=0.042990999752487

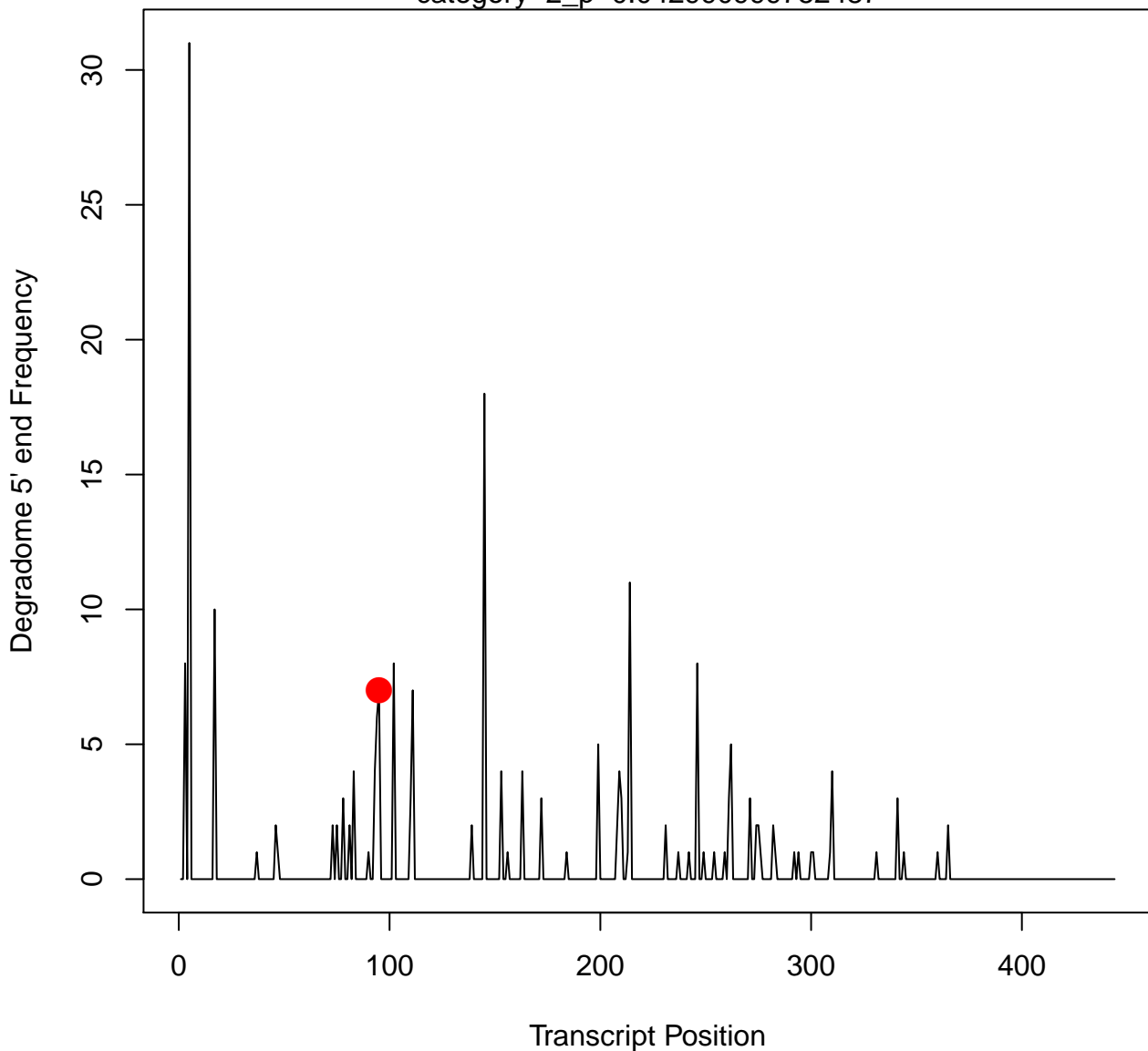

Supplement: Supplementary file 1 [file DataSheet_1.zip › The miRNA-target modules identified by the CleaveLand4/miRN2b-3p_evm.model.LG04.3983_95_TPlot.pdf]

**T=evm.model.LG03.4320\_Q=miRN2b-5p\_S=62**

category=0\_p=0.00155656346427469

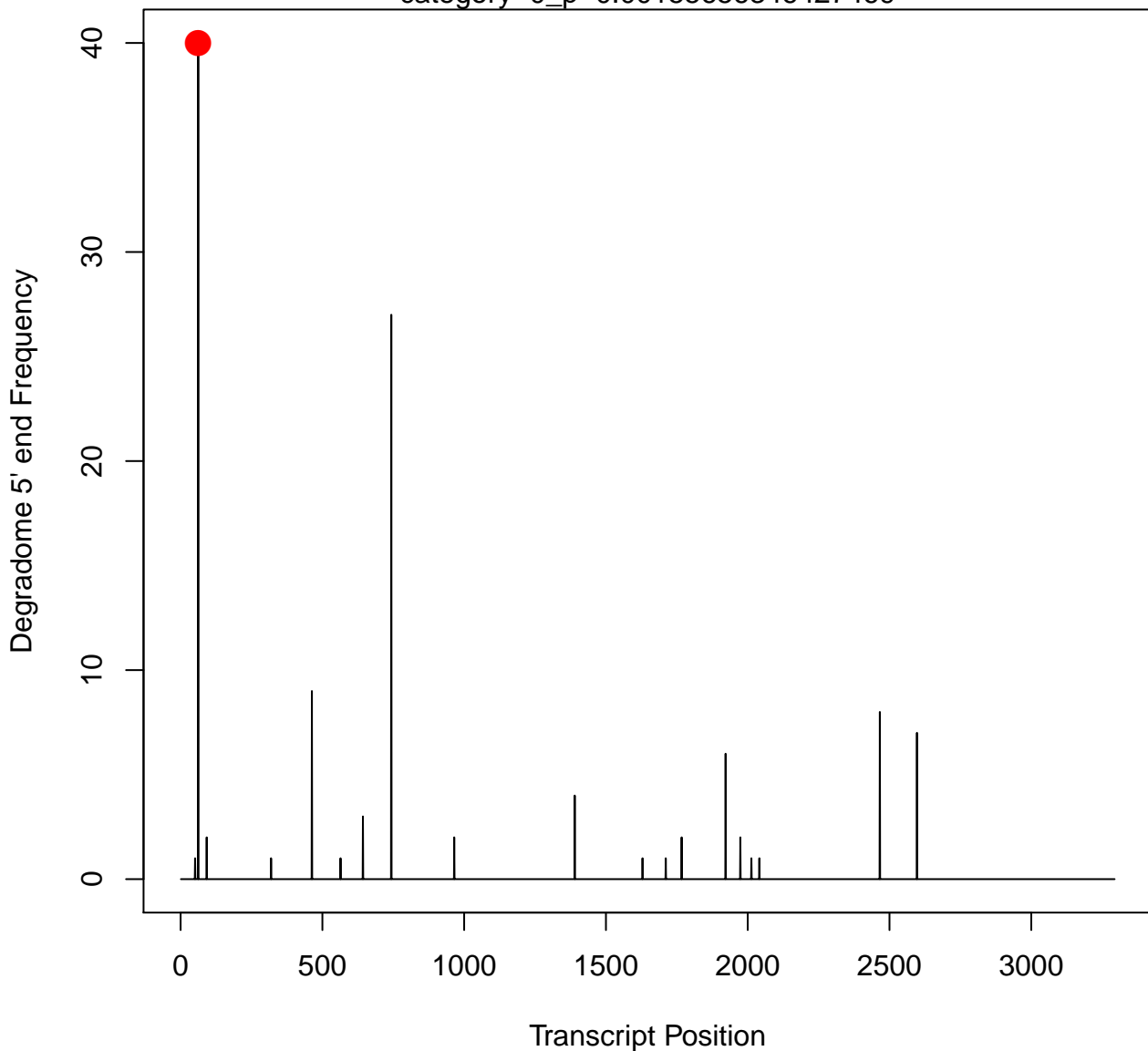

Supplement: Supplementary file 1 [file DataSheet_1.zip › The miRNA-target modules identified by the CleaveLand4/miRN2b-5p_evm.model.LG03.4320_62_TPlot.pdf]

**T=evm.model.LG01.6624\_Q=miRN3-5p\_S=83**

category=2\_p=0.0345432623396535

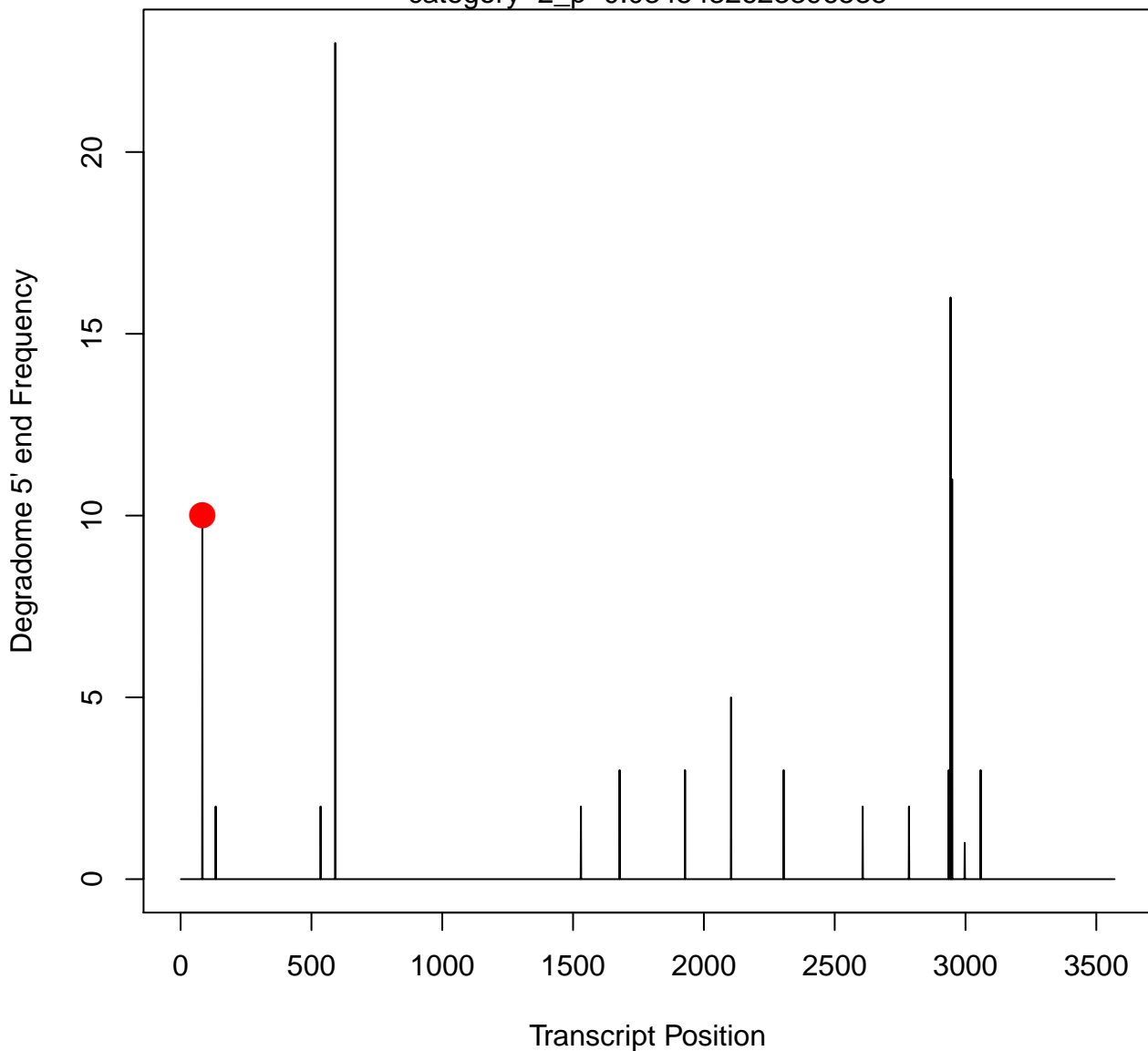

Supplement: Supplementary file 1 [file DataSheet_1.zip › The miRNA-target modules identified by the CleaveLand4/miRN3-5p_evm.model.LG01.6624_83_TPlot.pdf]

**T=evm.model.LG06.3489\_Q=miRN3-5p\_S=86**

category=0\_p=0.00051912393111464

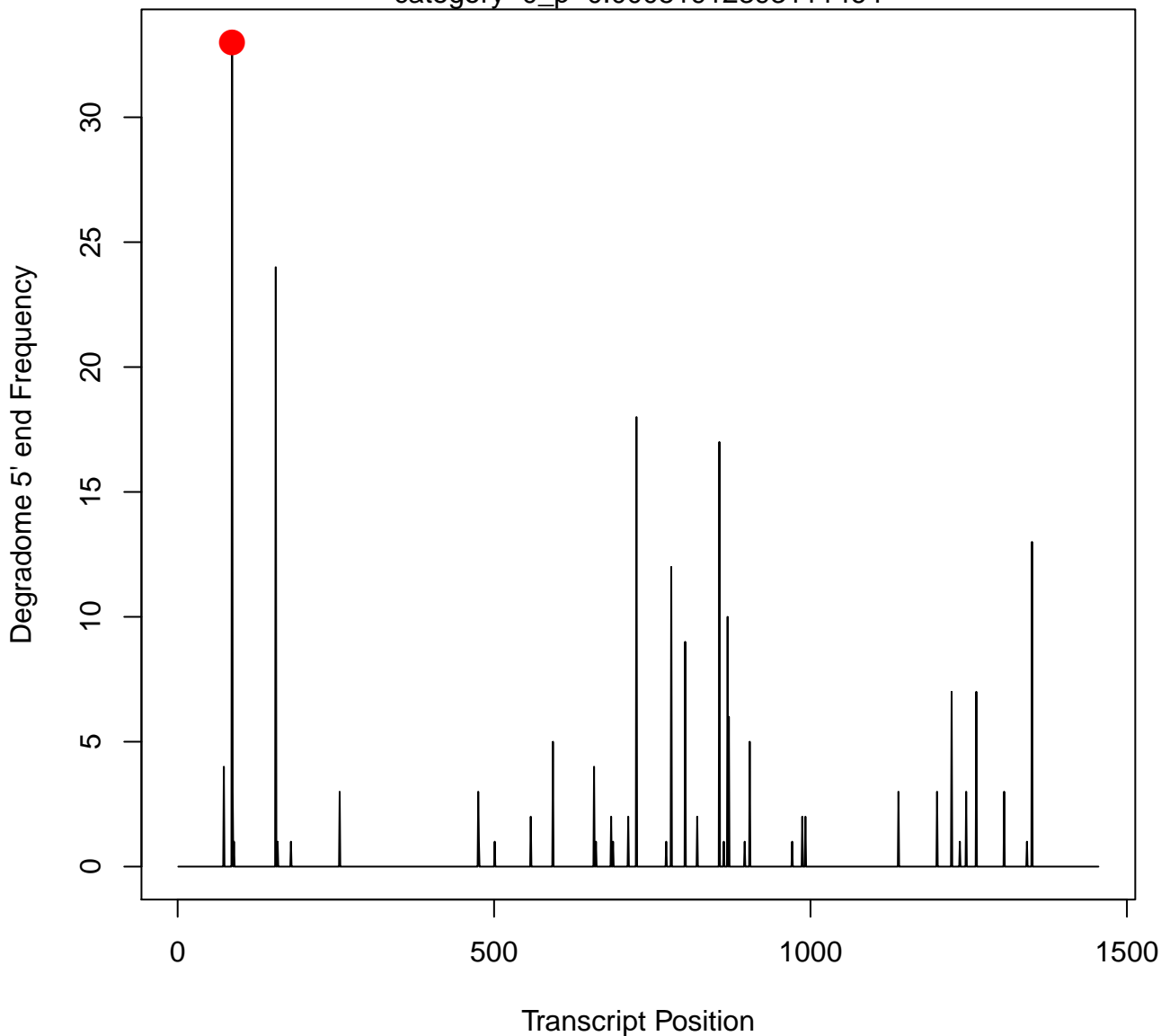

Supplement: Supplementary file 1 [file DataSheet_1.zip › The miRNA-target modules identified by the CleaveLand4/miRN3-5p_evm.model.LG06.3489_86_TPlot.pdf]

**T=evm.model.LG08.1113\_Q=miRN3-5p\_S=62**

category=2\_p=0.0174234189334928

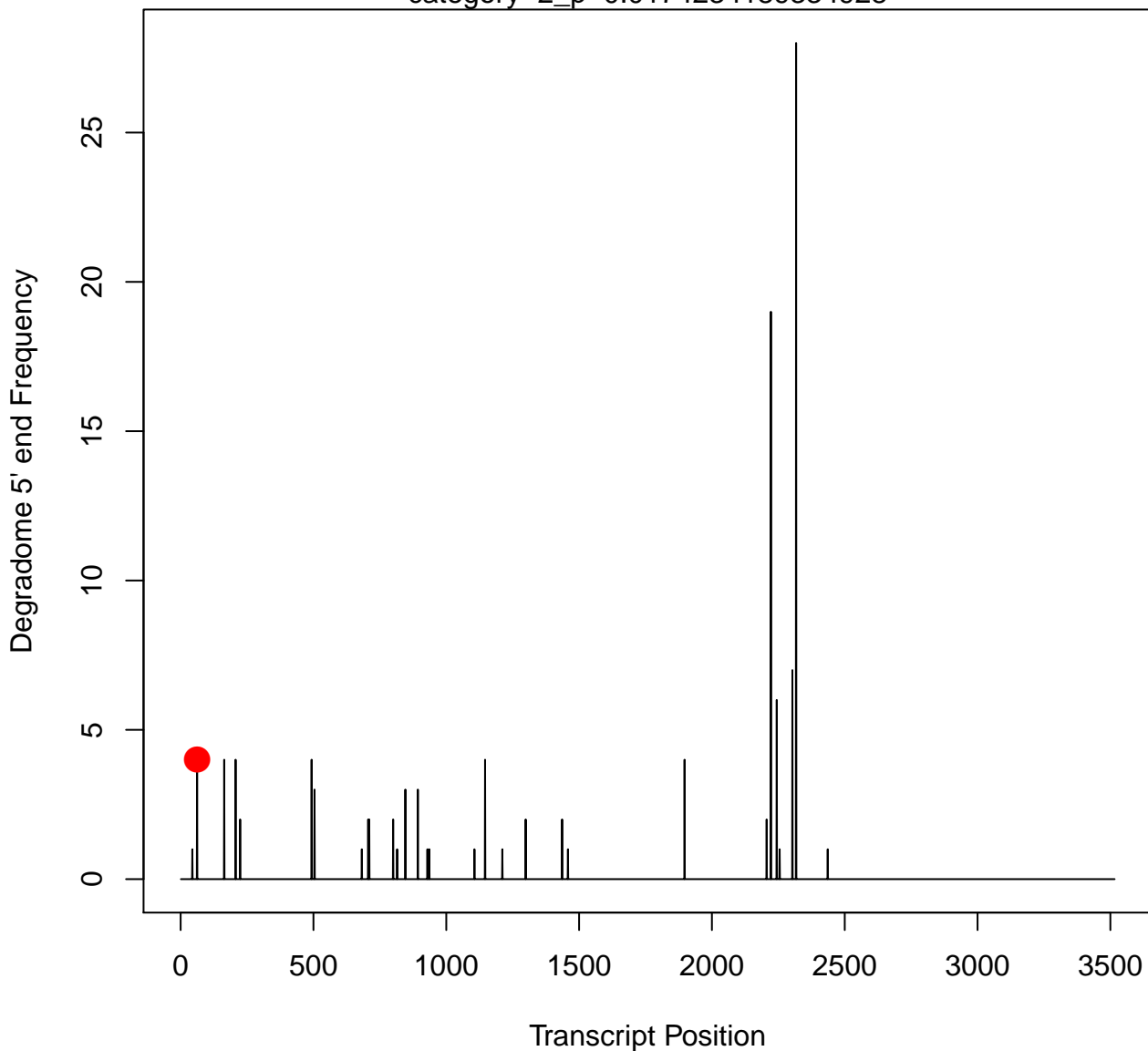

Supplement: Supplementary file 1 [file DataSheet_1.zip › The miRNA-target modules identified by the CleaveLand4/miRN3-5p_evm.model.LG08.1113_62_TPlot.pdf]

**T=evm.model.LG02.6165\_Q=miRN4-5p\_S=70**

category=0\_p=0.00672763087043382

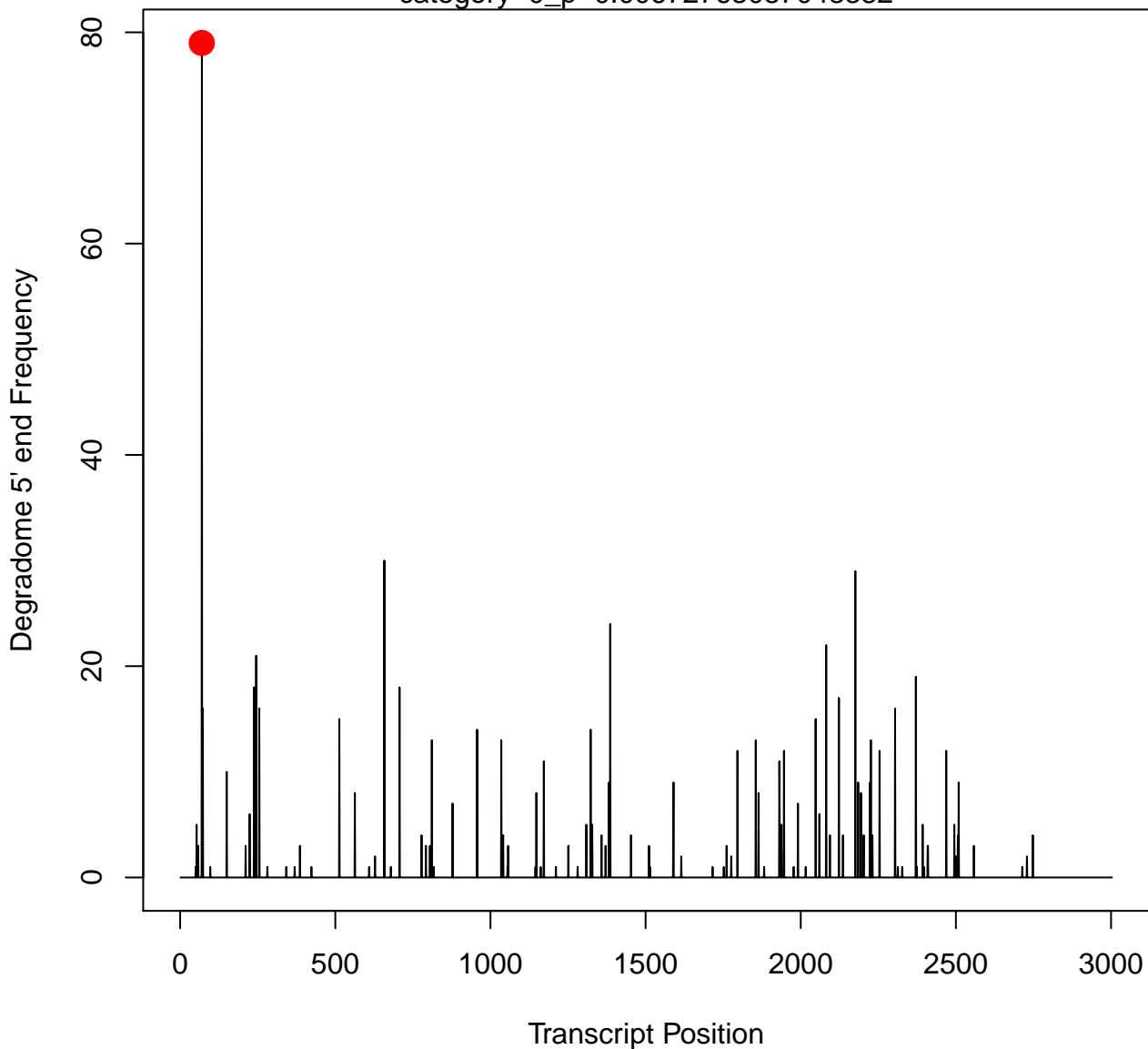

Supplement: Supplementary file 1 [file DataSheet_1.zip › The miRNA-target modules identified by the CleaveLand4/miRN4-5p_evm.model.LG02.6165_70_TPlot.pdf]

**T=evm.model.LG01.7680\_Q=miRN5-3p\_S=1218**

category=2\_p=0.042990999752487

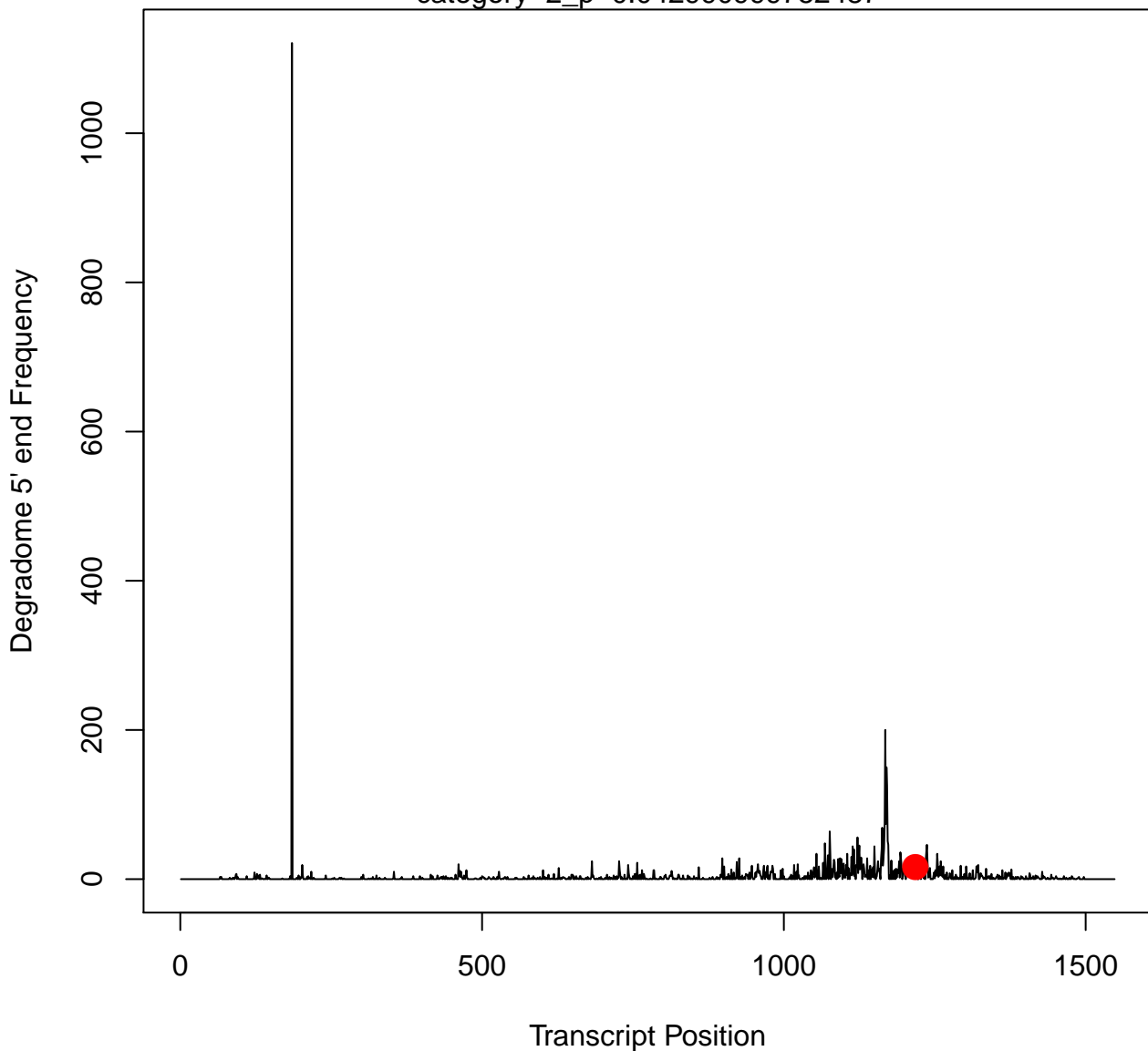

Supplement: Supplementary file 1 [file DataSheet_1.zip › The miRNA-target modules identified by the CleaveLand4/miRN5-3p_evm.model.LG01.7680_1218_TPlot.pdf]

**T=evm.model.LG03.51\_Q=miRN5-3p\_S=656**

category=3\_p=0.0477069564524738

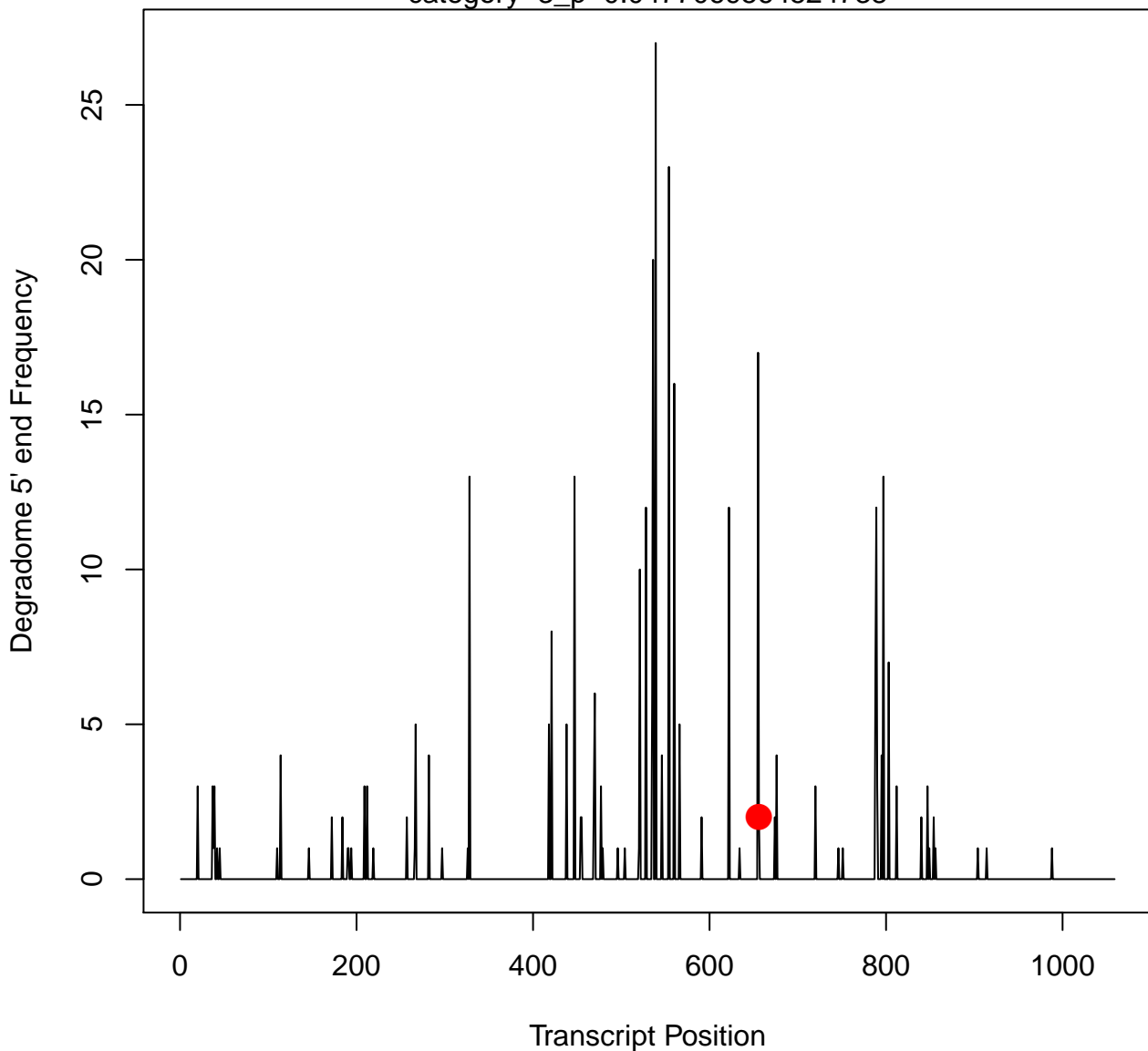

Supplement: Supplementary file 1 [file DataSheet_1.zip › The miRNA-target modules identified by the CleaveLand4/miRN5-3p_evm.model.LG03.51_656_TPlot.pdf]

**T=evm.model.LG02.692\_Q=miRN7-5p\_S=755**

category=4\_p=0.00862274555333054

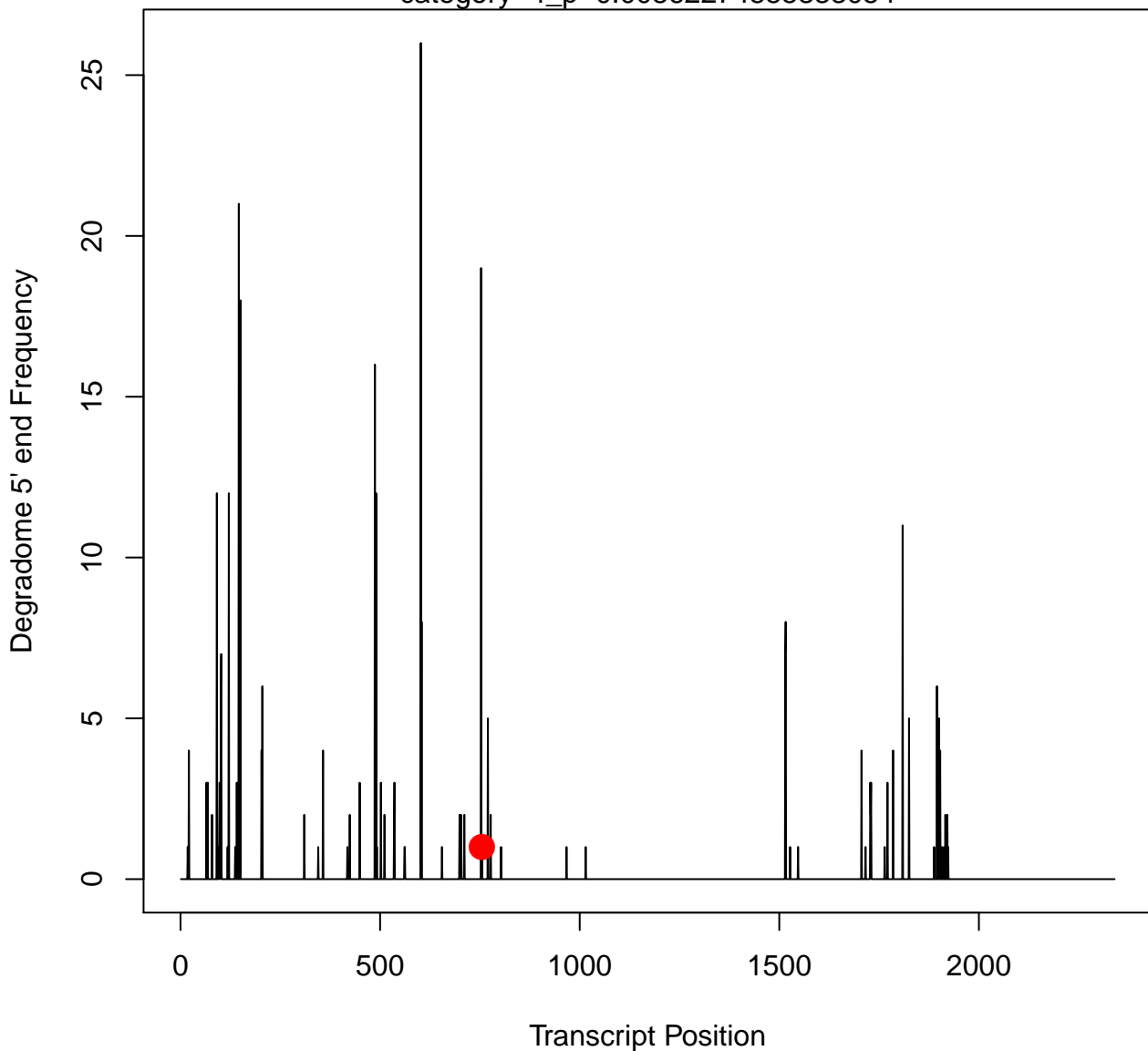

Supplement: Supplementary file 1 [file DataSheet_1.zip › The miRNA-target modules identified by the CleaveLand4/miRN7-5p_evm.model.LG02.692_755_TPlot.pdf]

**T=evm.model.LG02.4722\_Q=miRN8-5p\_S=316**

category=2\_p=0.0260209548157995

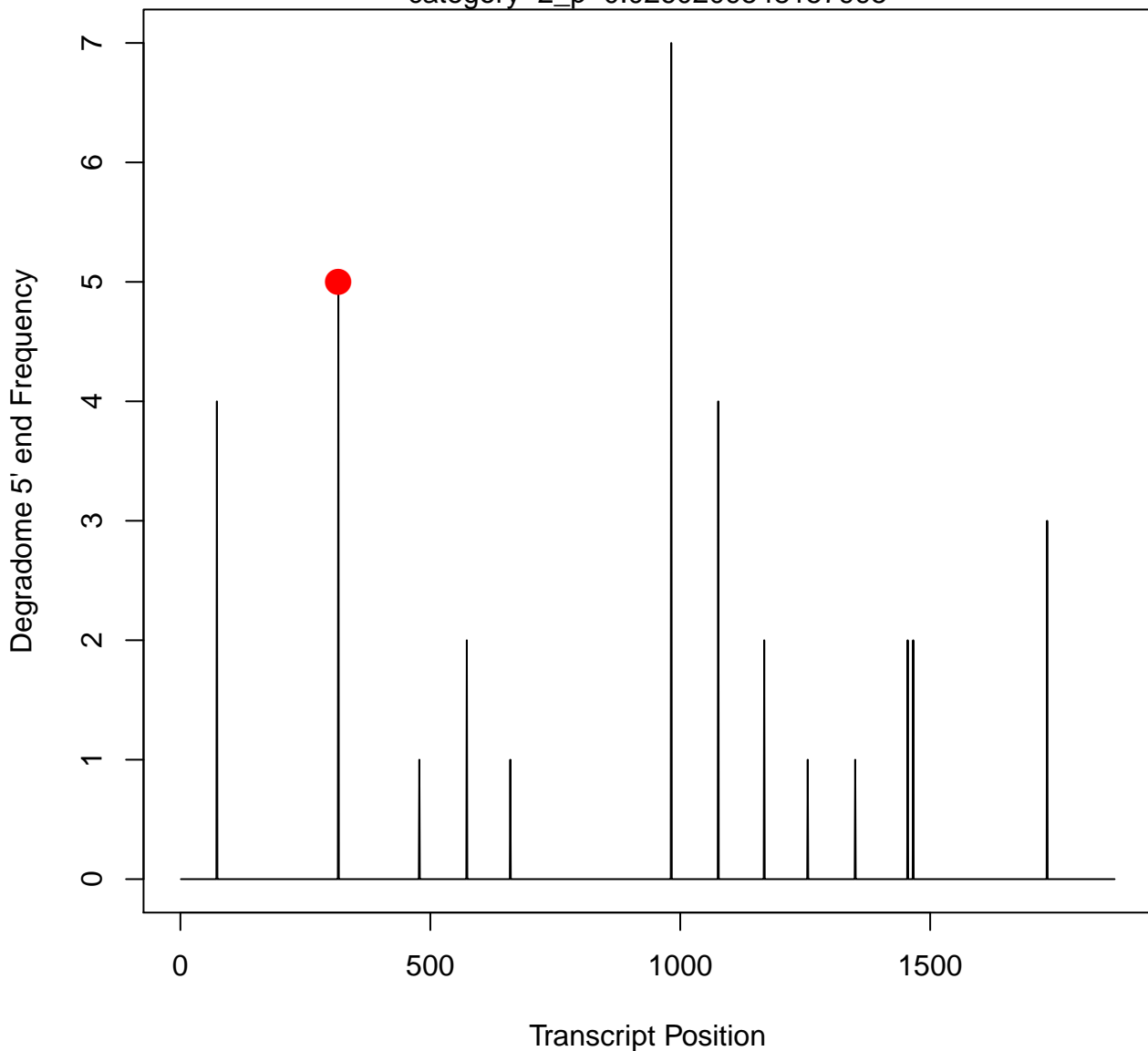

Supplement: Supplementary file 1 [file DataSheet_1.zip › The miRNA-target modules identified by the CleaveLand4/miRN8-5p_evm.model.LG02.4722_316_TPlot.pdf]

**T=evm.model.LG02.4727\_Q=miRN8-5p\_S=1756**

category=0\_p=0.00775862610766487

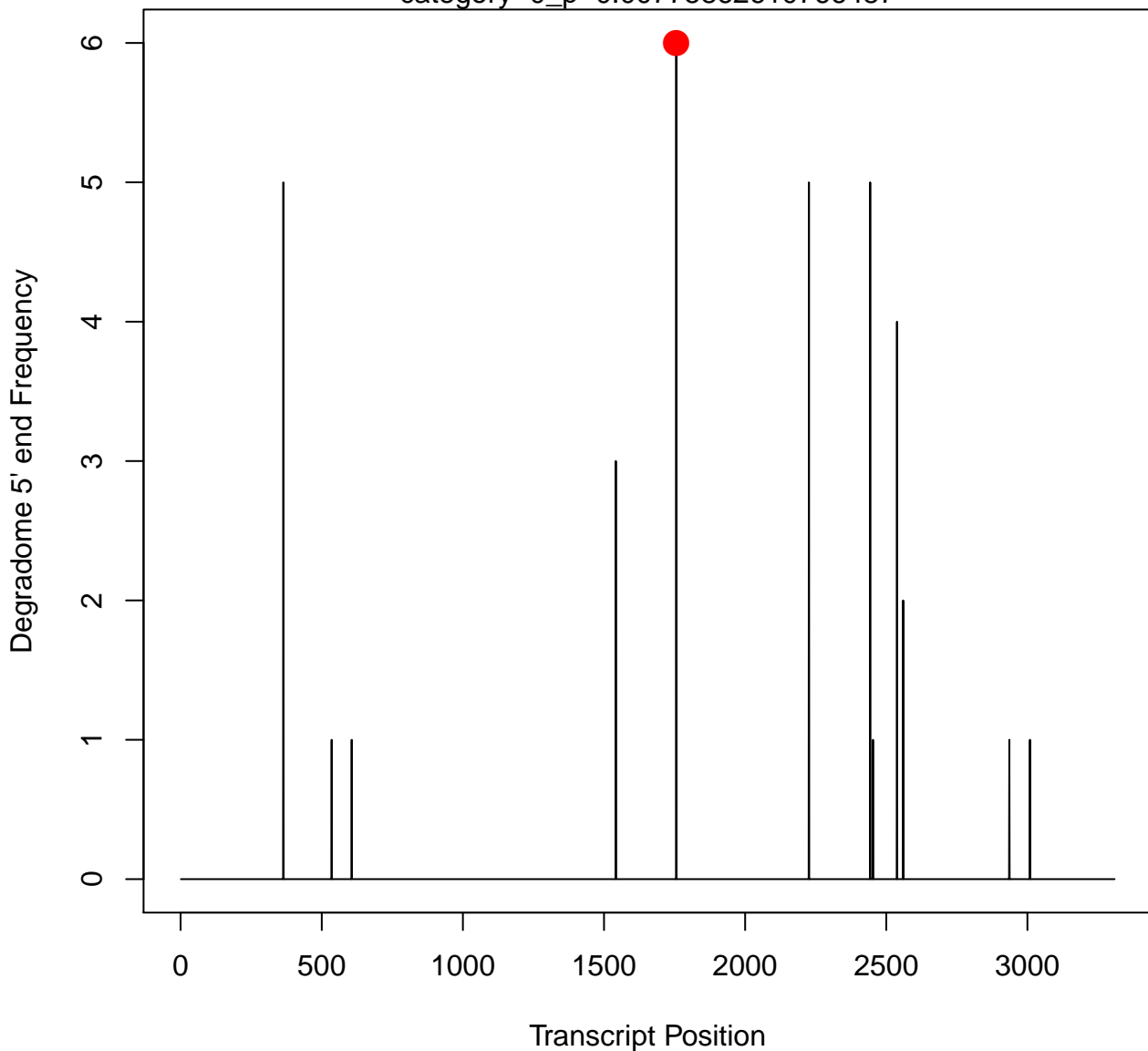

Supplement: Supplementary file 1 [file DataSheet_1.zip › The miRNA-target modules identified by the CleaveLand4/miRN8-5p_evm.model.LG02.4727_1756_TPlot.pdf]

**T=evm.model.LG02.4741\_Q=miRN8-5p\_S=316**

category=2\_p=0.0345432623396535

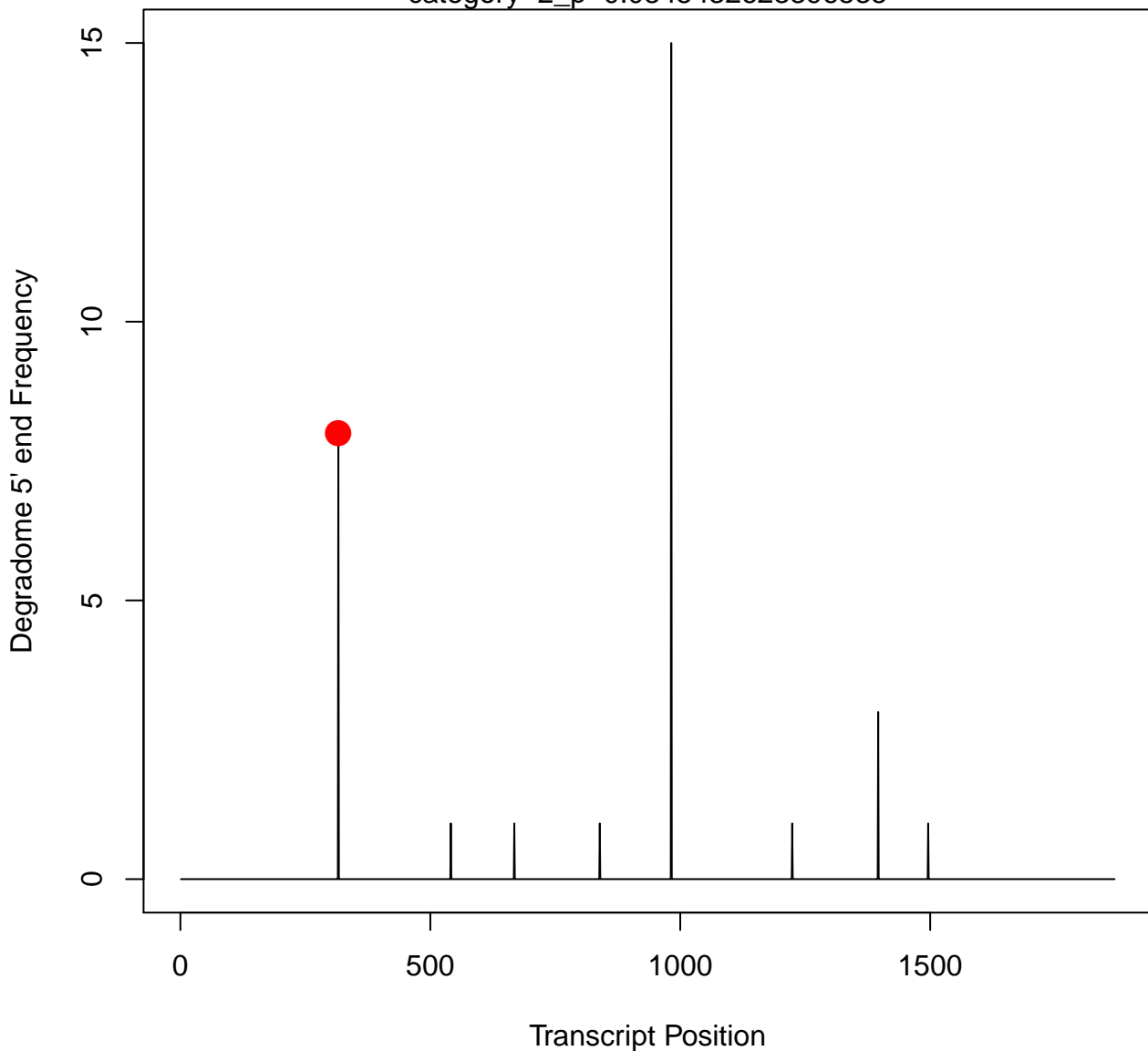

Supplement: Supplementary file 1 [file DataSheet_1.zip › The miRNA-target modules identified by the CleaveLand4/miRN8-5p_evm.model.LG02.4741_316_TPlot.pdf]

**T=evm.model.LG02.4761\_Q=miRN8-5p\_S=316**

category=3\_p=0.0241449679652586

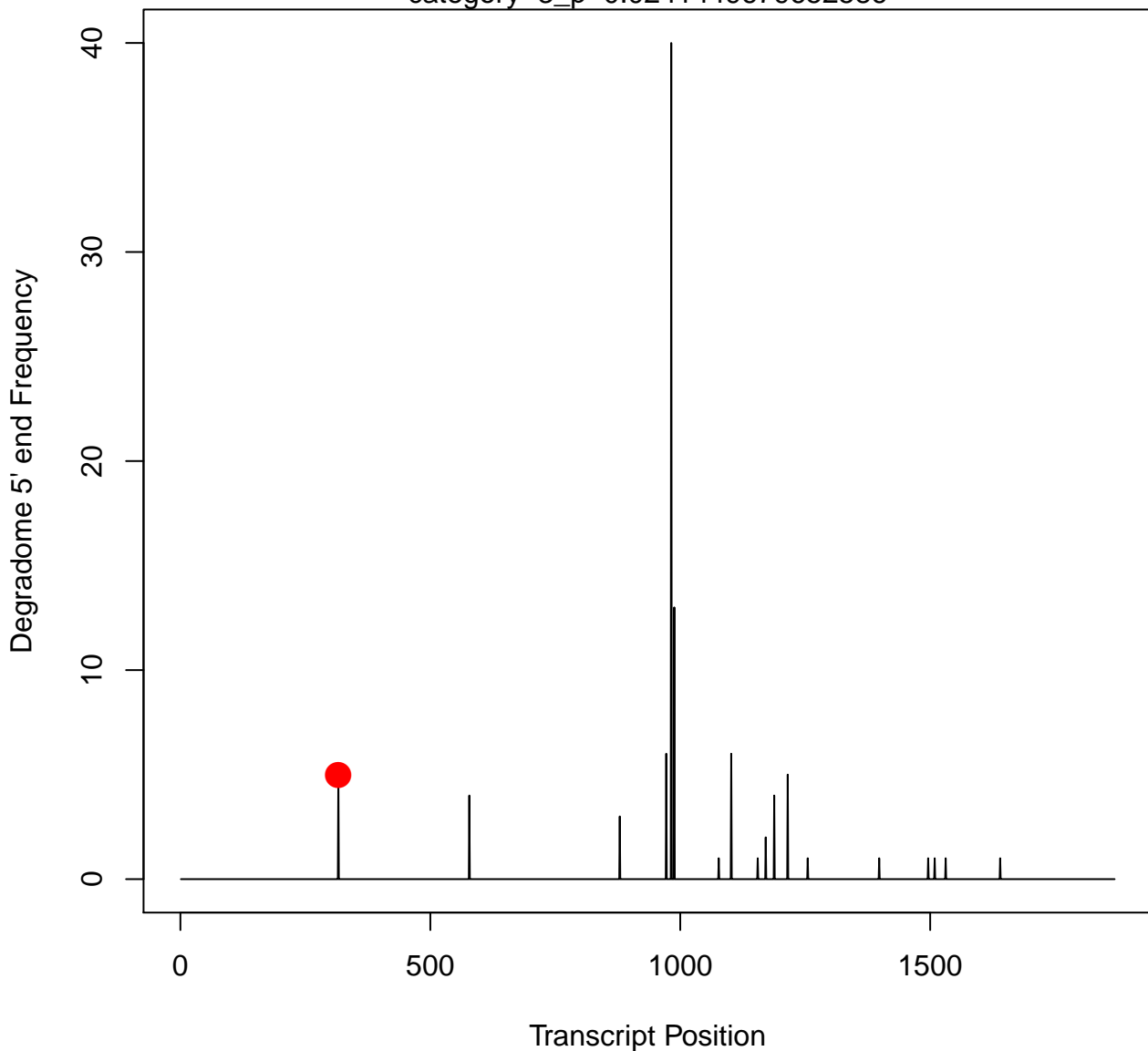

Supplement: Supplementary file 1 [file DataSheet_1.zip › The miRNA-target modules identified by the CleaveLand4/miRN8-5p_evm.model.LG02.4761_316_TPlot.pdf]

**T=evm.model.LG02.2248\_Q=miRN9-5p\_S=752**

category=4\_p=0.0256458225535021

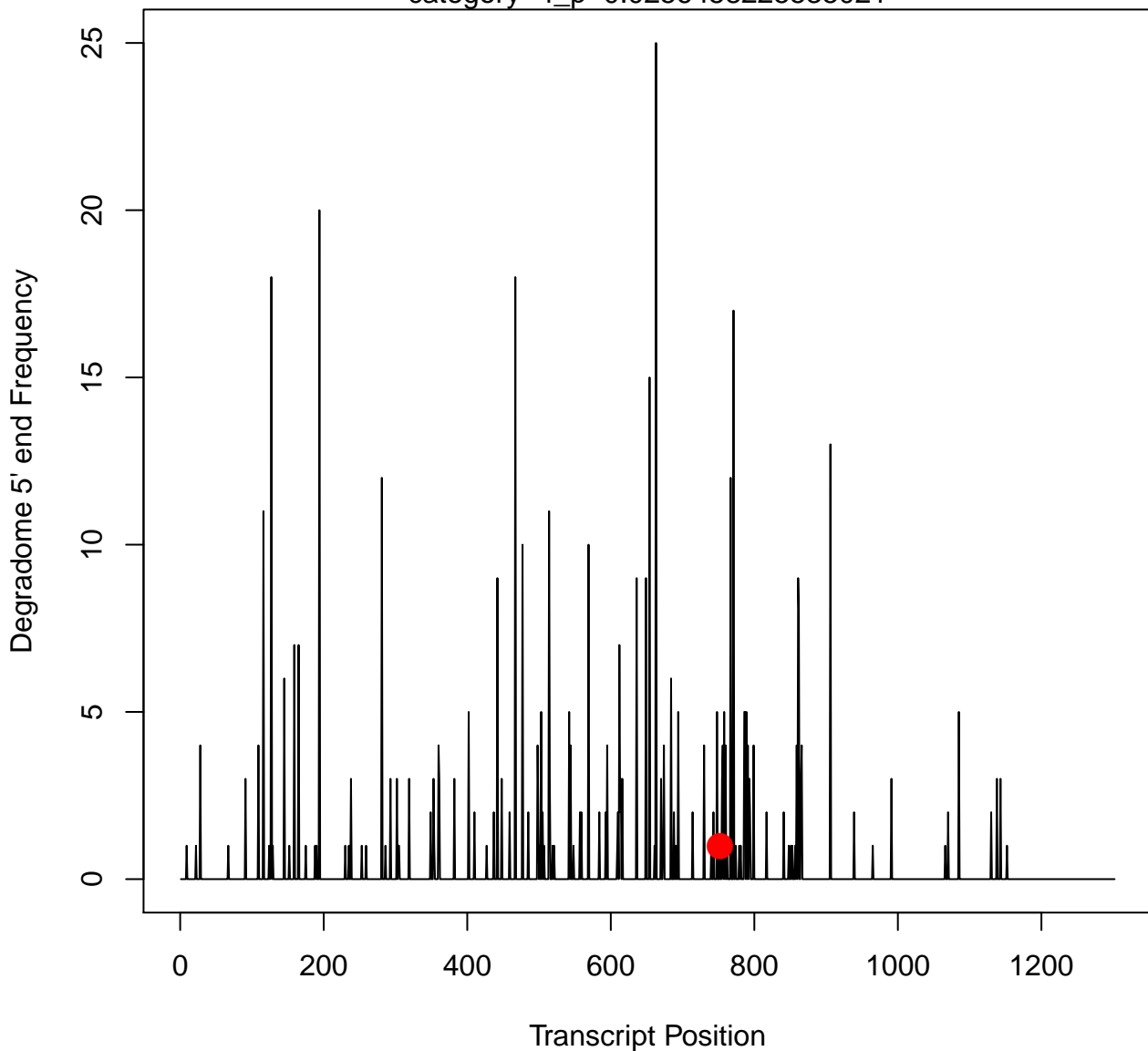

Supplement: Supplementary file 1 [file DataSheet_1.zip › The miRNA-target modules identified by the CleaveLand4/miRN9-5p_evm.model.LG02.2248_752_TPlot.pdf]

**T=evm.model.LG04.1330\_Q=miRN9-5p\_S=752**

category=3\_p=0.012146249673191

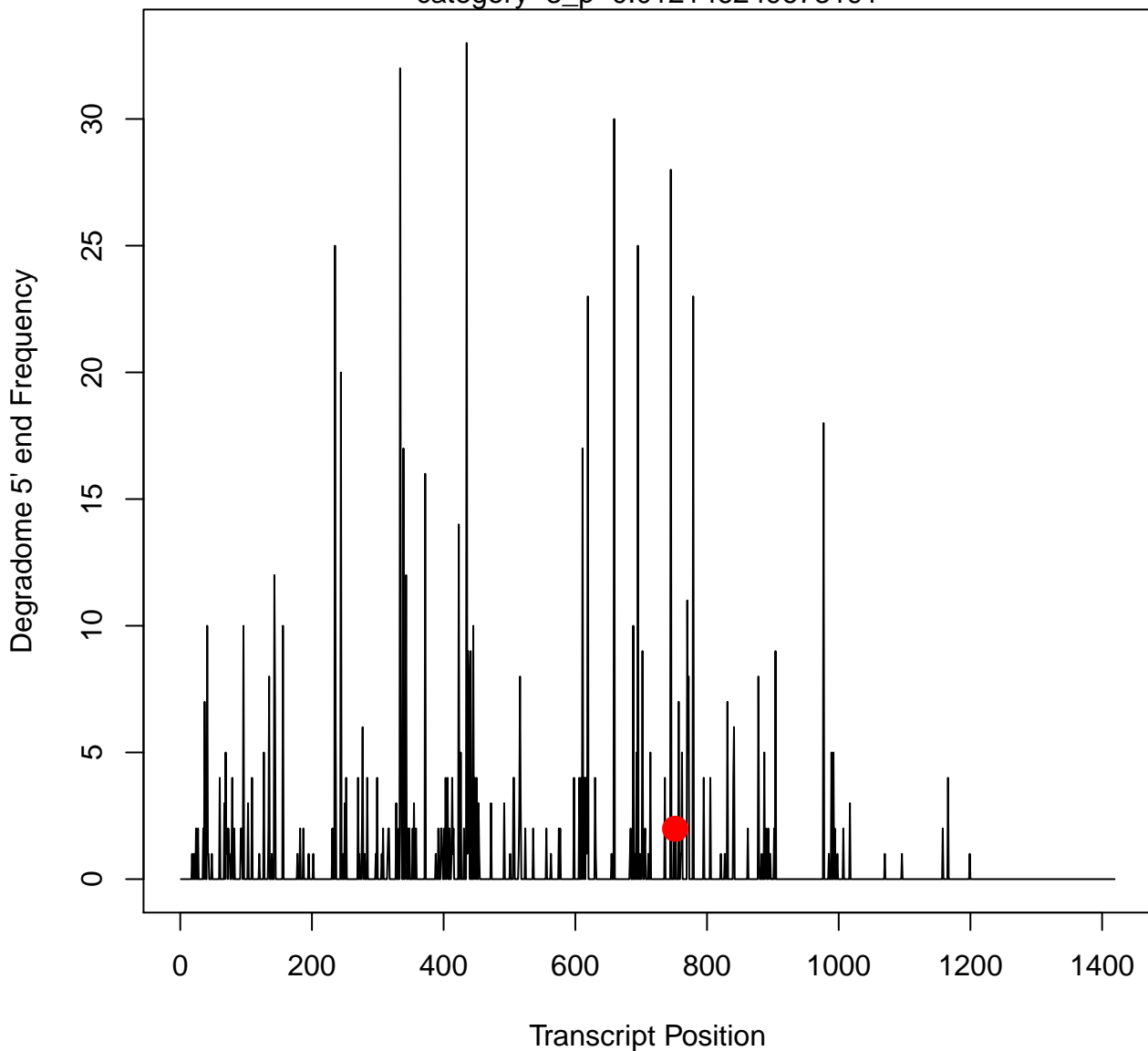

Supplement: Supplementary file 1 [file DataSheet_1.zip › The miRNA-target modules identified by the CleaveLand4/miRN9-5p_evm.model.LG04.1330_752_TPlot.pdf]

**T=evm.model.LG04.903\_Q=miRN9-5p\_S=752**

category=2\_p=0.0174234189334928

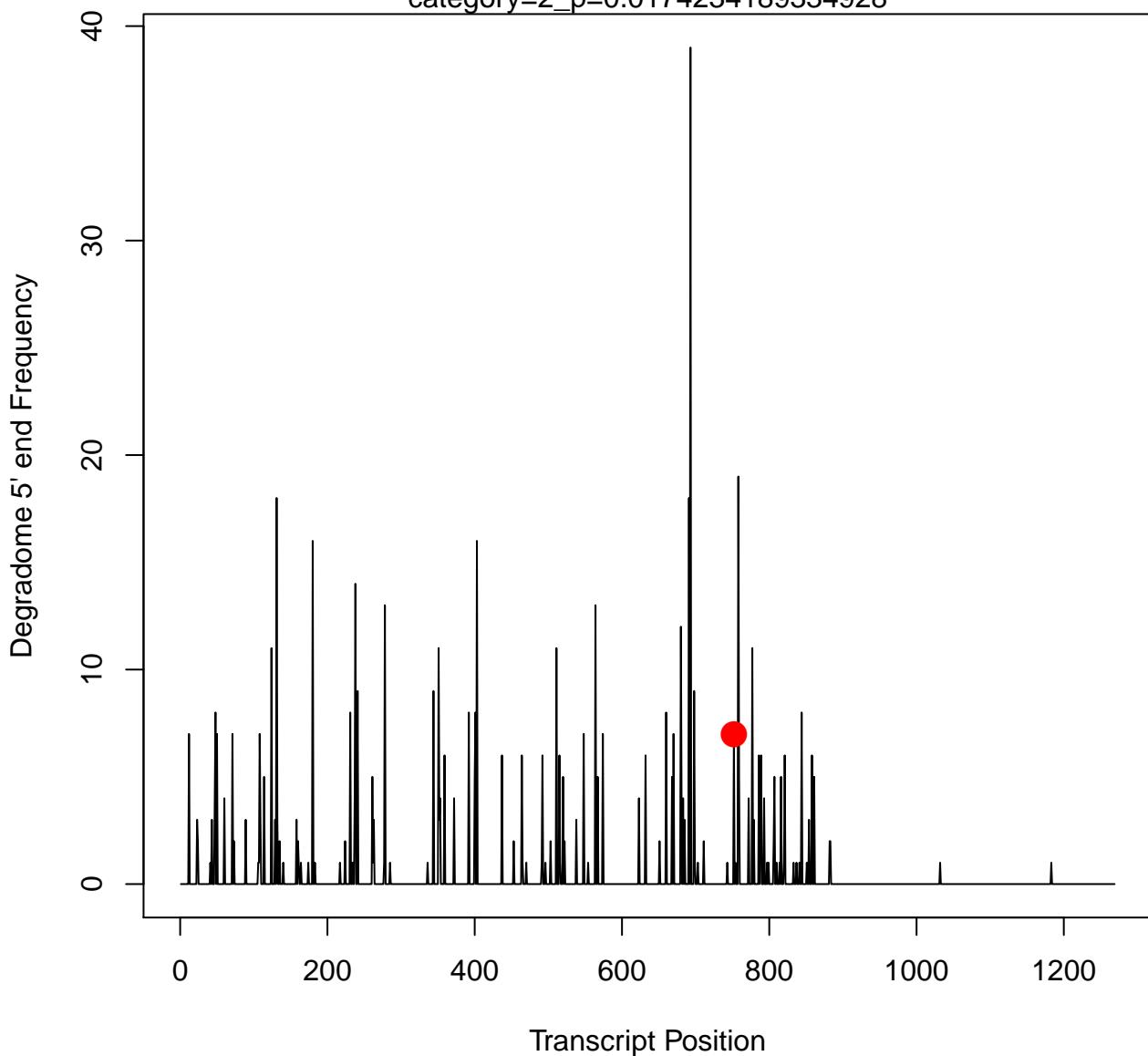

Supplement: Supplementary file 1 [file DataSheet_1.zip › The miRNA-target modules identified by the CleaveLand4/miRN9-5p_evm.model.LG04.903_752_TPlot.pdf]

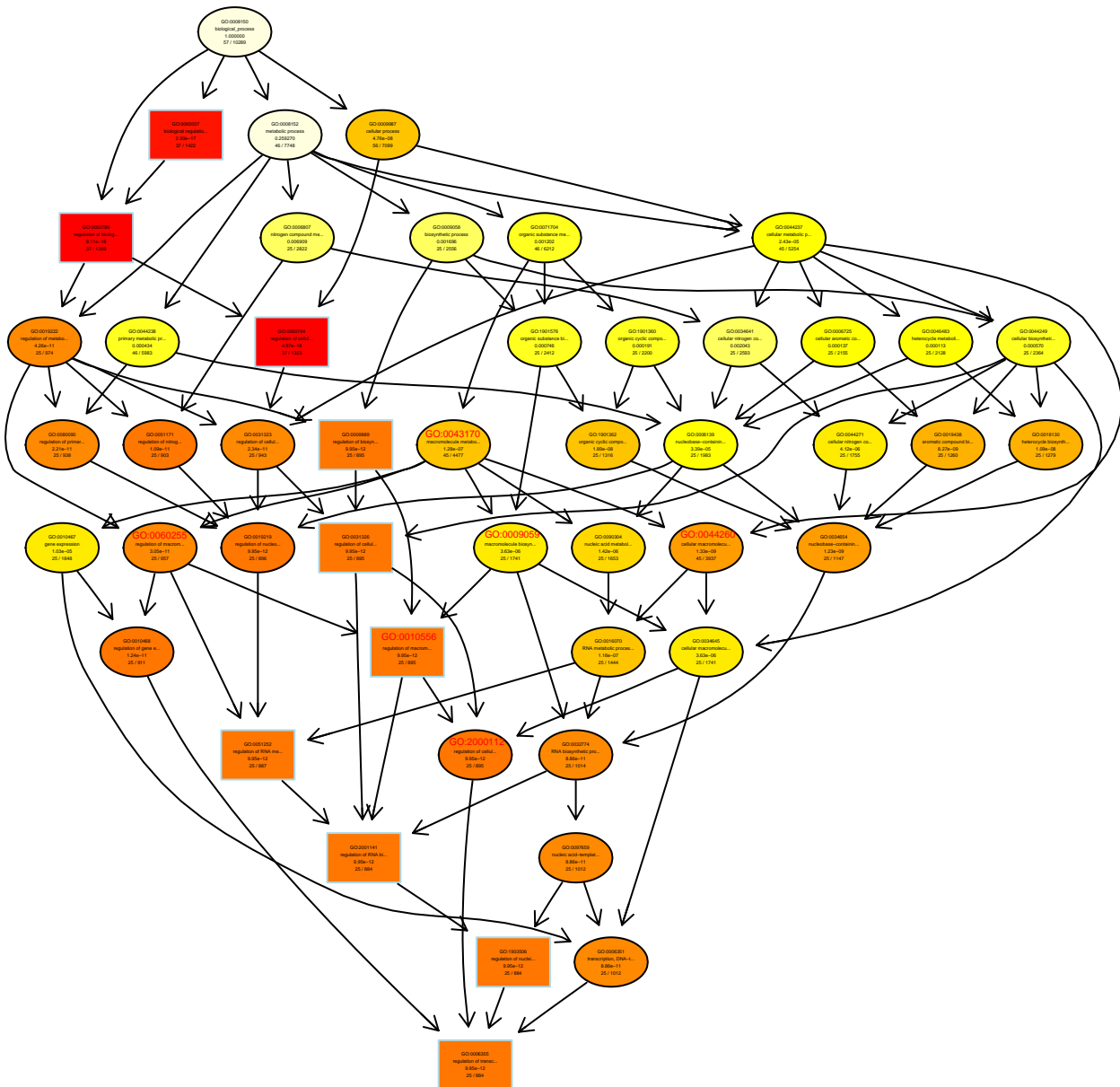

Supplement: Supplementary Figure 1 — Root ontology analysis of the enriched GO terms. The GO terms involving macromolecule-related pathways are labeled in red and displayed in an enlarged font. [file Image_1.pdf]
