# Supplementary figures and images for: Identification of miRNAs and their targets in two Taraxacum species with contrasting rubber-producing ability (part 1 of 2)
Source: Front Plant Sci. 2023 Nov 8;14:1287318. doi: 10.3389/fpls.2023.1287318 (PMC10663287; doi:10.3389/fpls.2023.1287318)

**T=evm.model.LG01.3873\_Q=miR156c-5p\_S=747**

category=4\_p=0.00862274555333054

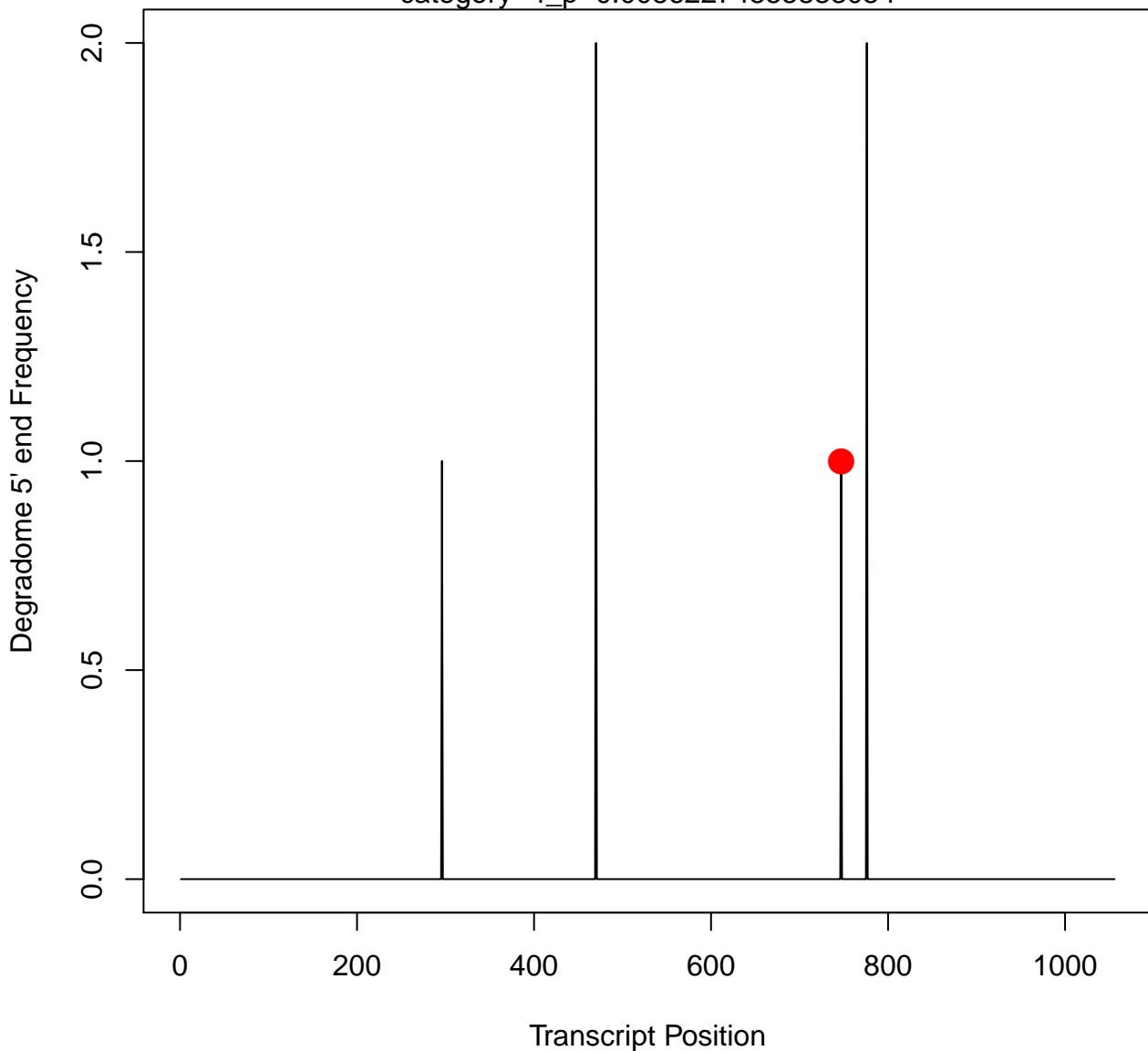

Supplement: Supplementary file 1 [file DataSheet_1.zip › The miRNA-target modules identified by the CleaveLand4/miR156c-5p_evm.model.LG01.3873_747_TPlot.pdf]

**T=evm.model.LG06.290\_Q=miR156c-5p\_S=933**

category=3\_p=0.0241449679652586

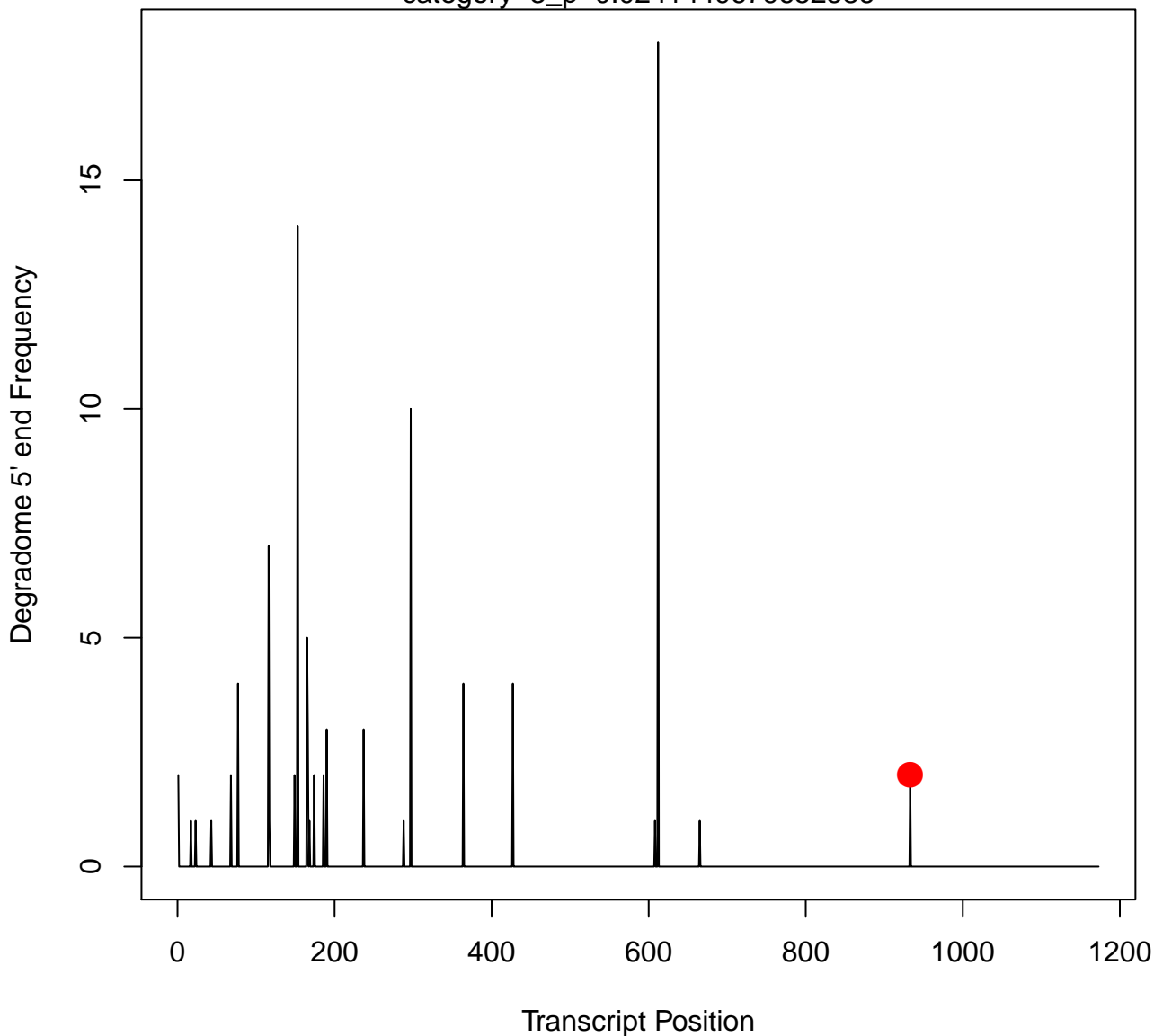

Supplement: Supplementary file 1 [file DataSheet_1.zip › The miRNA-target modules identified by the CleaveLand4/miR156c-5p_evm.model.LG06.290_933_TPlot.pdf]

**T=evm.model.LG02.1997\_Q=miR159a-3p\_S=739**

category=0\_p=0.0113586892874949

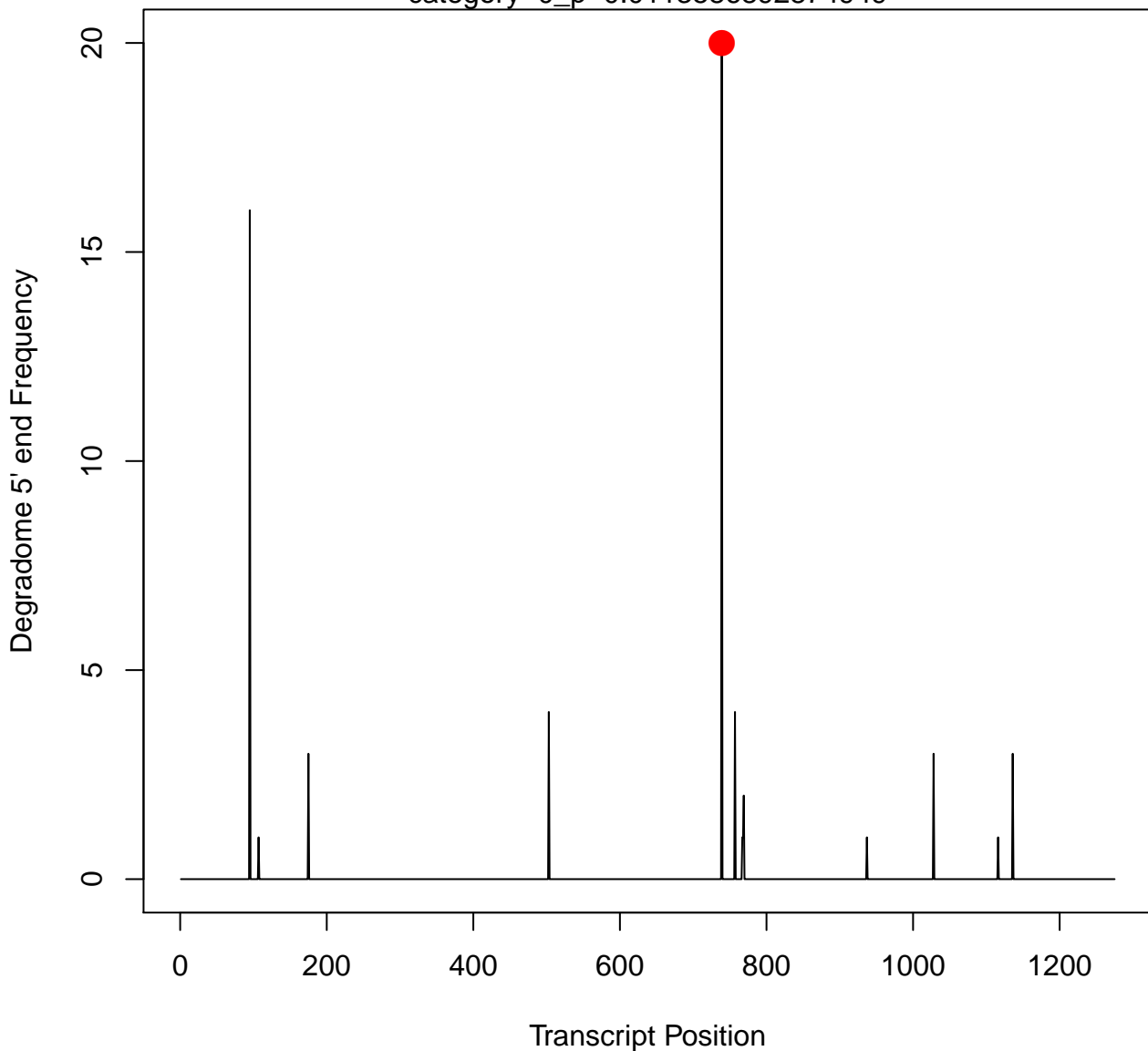

Supplement: Supplementary file 1 [file DataSheet_1.zip › The miRNA-target modules identified by the CleaveLand4/miR159a-3p_evm.model.LG02.1997_739_TPlot.pdf]

**T=evm.model.LG02.3374\_Q=miR159b-3p\_S=859**

category=0\_p=0.00103797837257324

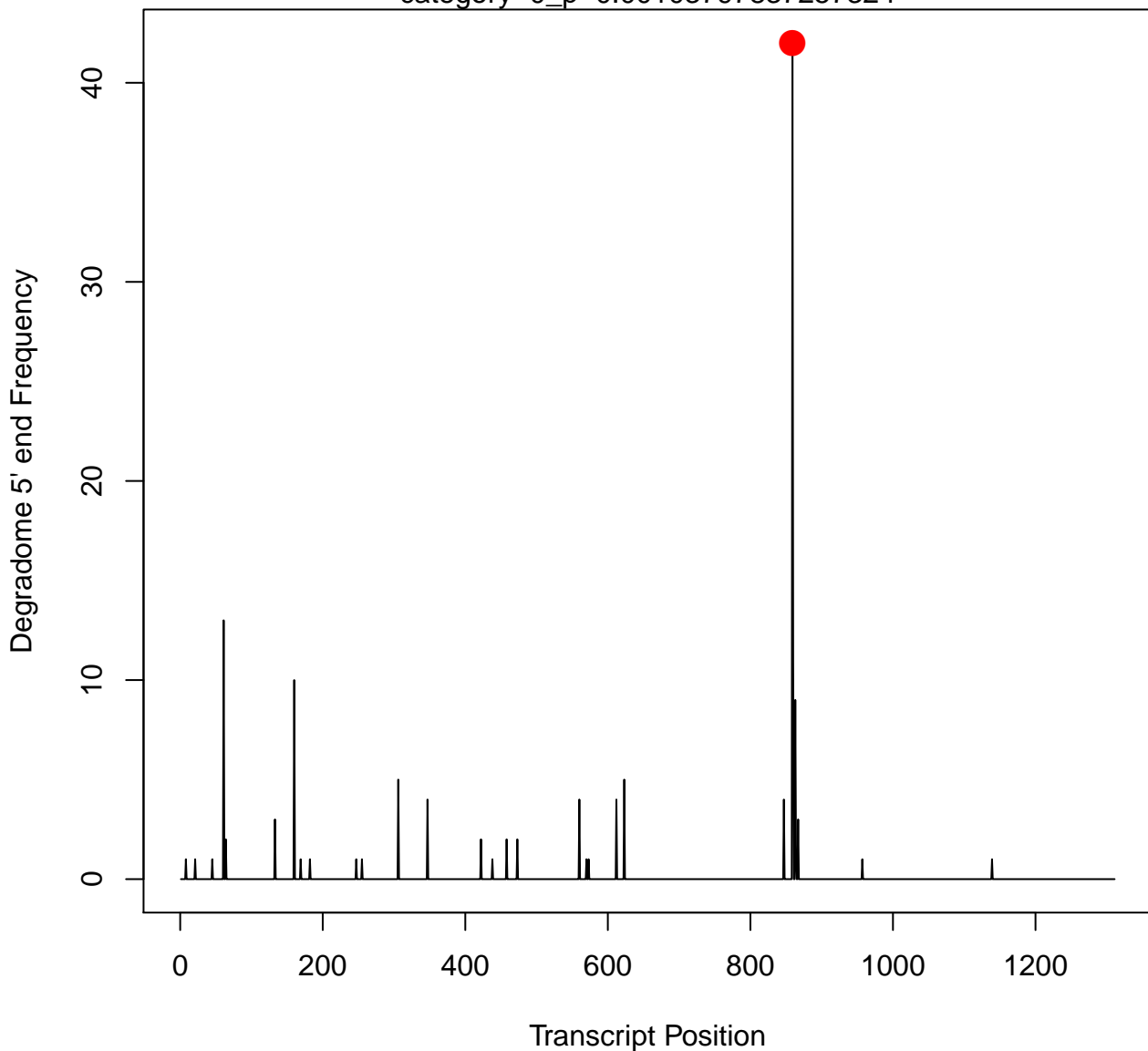

Supplement: Supplementary file 1 [file DataSheet_1.zip › The miRNA-target modules identified by the CleaveLand4/miR159b-3p_evm.model.LG02.3374_859_TPlot.pdf]

**T=evm.model.LG01.5621\_Q=miR160a-5p\_S=1223**

category=0\_p=0.00259292615763651

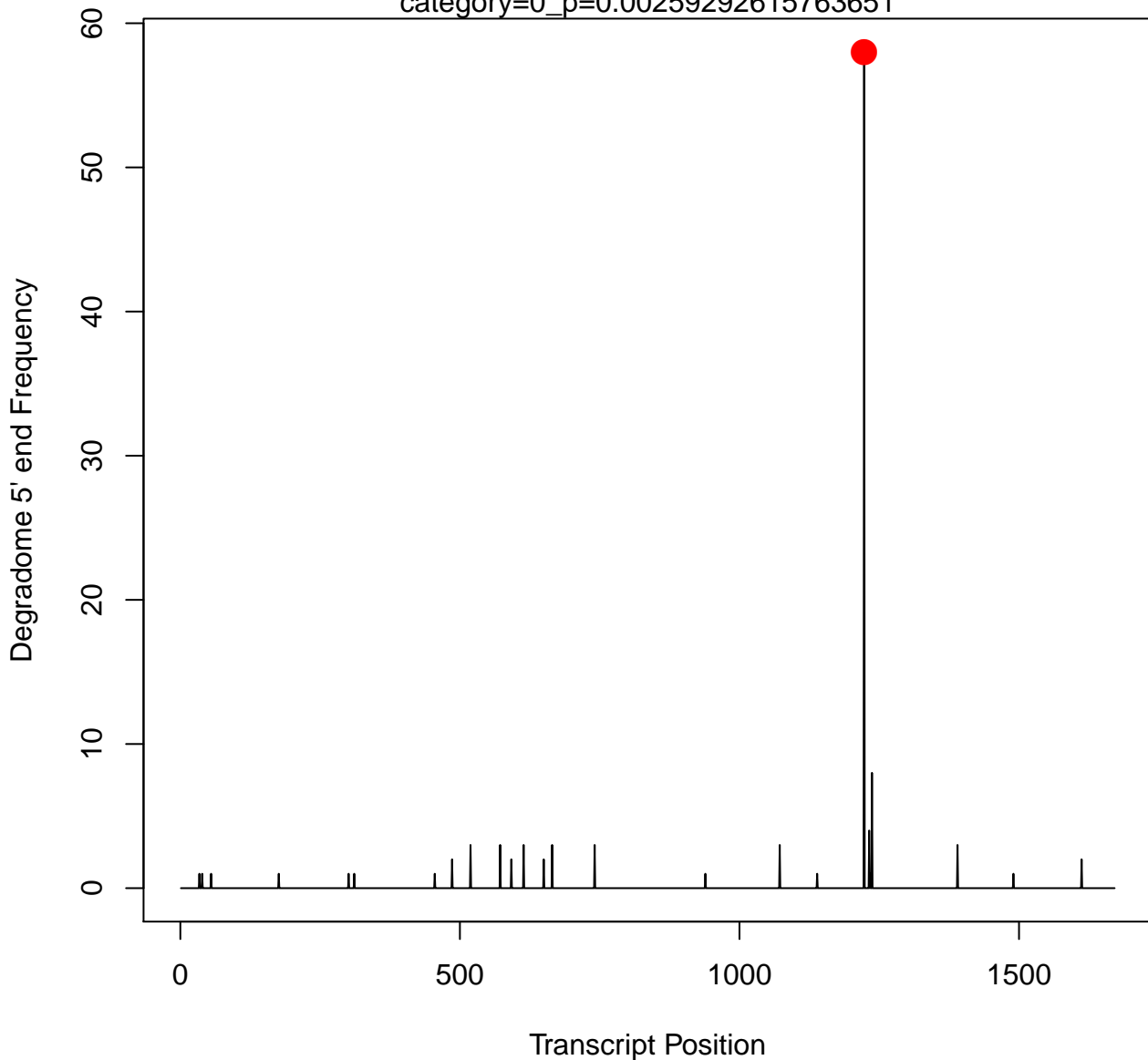

Supplement: Supplementary file 1 [file DataSheet_1.zip › The miRNA-target modules identified by the CleaveLand4/miR160a-5p_evm.model.LG01.5621_1223_TPlot.pdf]

**T=evm.model.LG02.5200\_Q=miR160a-5p\_S=1376**

category=0\_p=0.00051912393111464

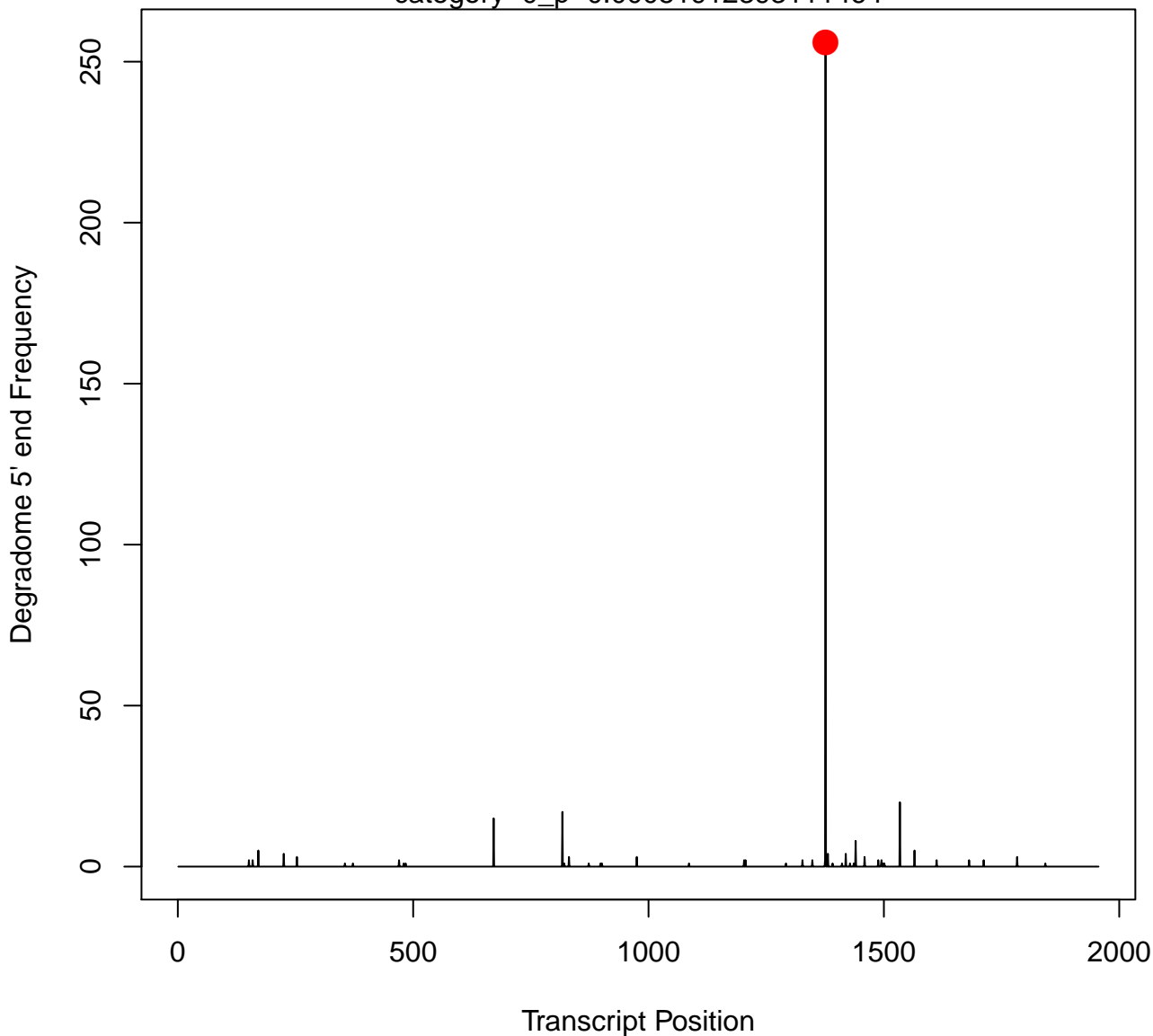

Supplement: Supplementary file 1 [file DataSheet_1.zip › The miRNA-target modules identified by the CleaveLand4/miR160a-5p_evm.model.LG02.5200_1376_TPlot.pdf]

**T=evm.model.LG04.4379\_Q=miR160a-5p\_S=1265**

category=0\_p=0.00103797837257324

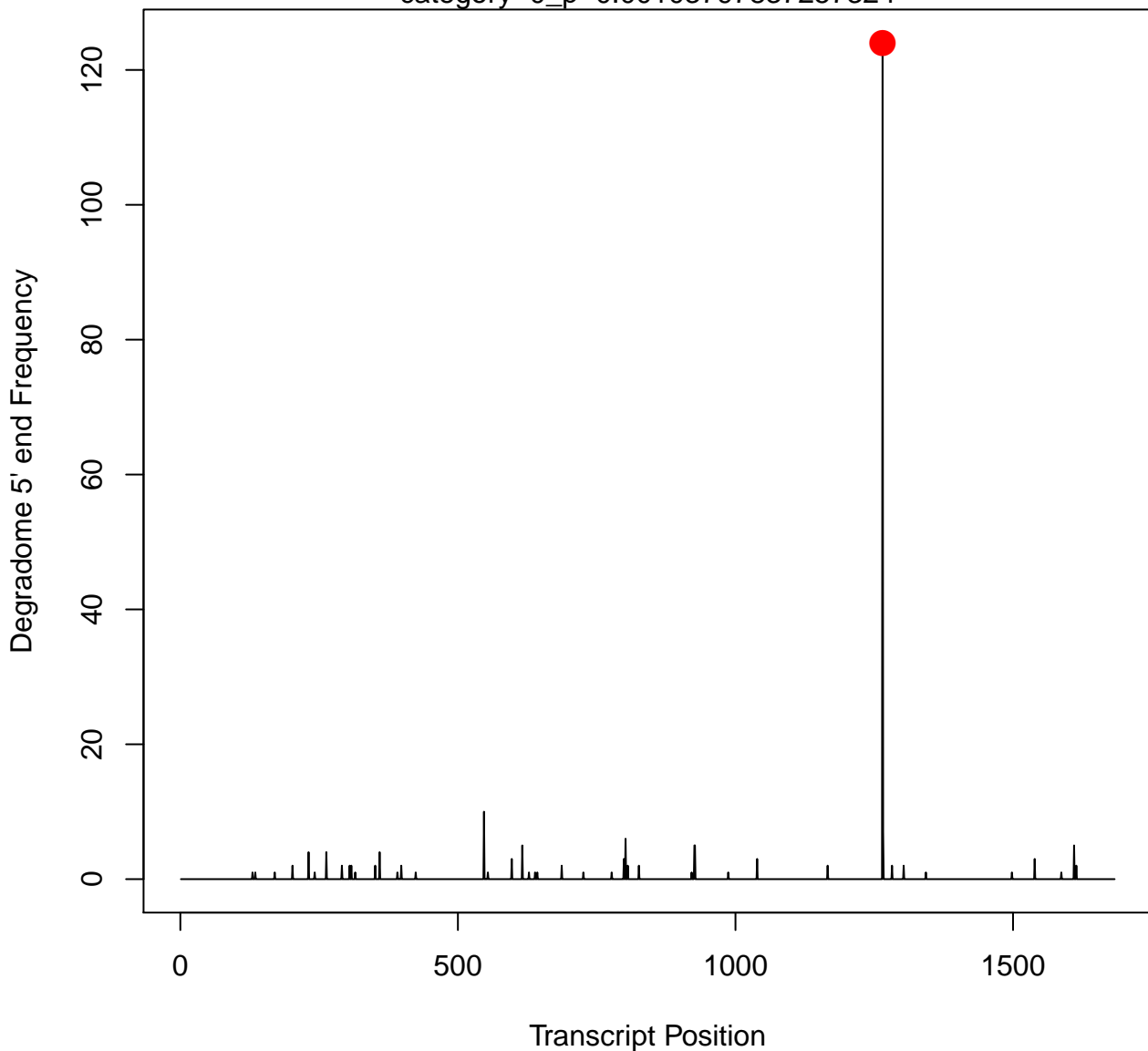

Supplement: Supplementary file 1 [file DataSheet_1.zip › The miRNA-target modules identified by the CleaveLand4/miR160a-5p_evm.model.LG04.4379_1265_TPlot.pdf]

**T=evm.model.LG03.4889\_Q=miR160c-3p\_S=326**

category=3\_p=0.0477069564524738

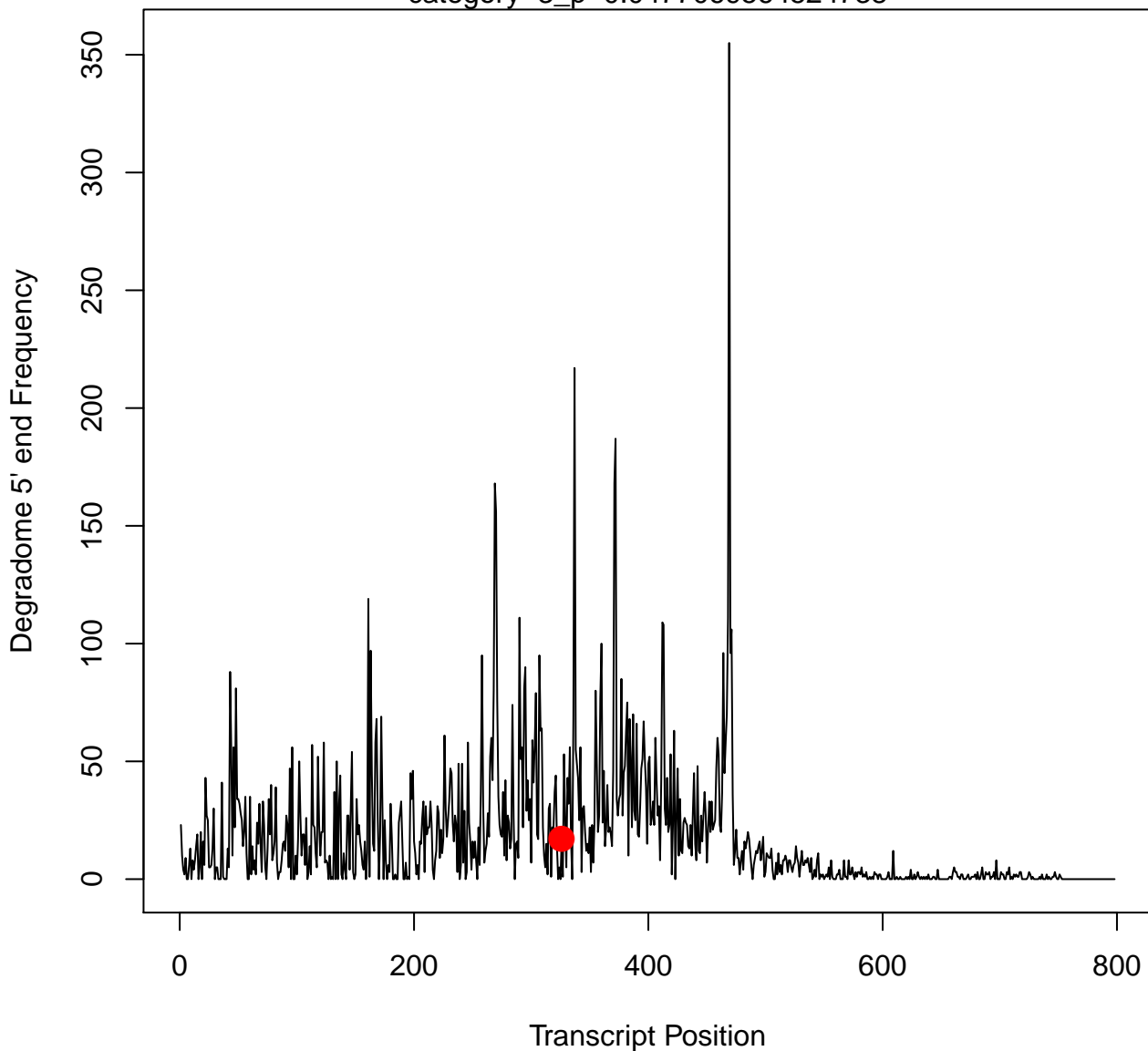

Supplement: Supplementary file 1 [file DataSheet_1.zip › The miRNA-target modules identified by the CleaveLand4/miR160c-3p_evm.model.LG03.4889_326_TPlot.pdf]

**T=evm.model.LG01.2348\_Q=miR160c-5p\_S=1178**

category=0\_p=0.00155656346427469

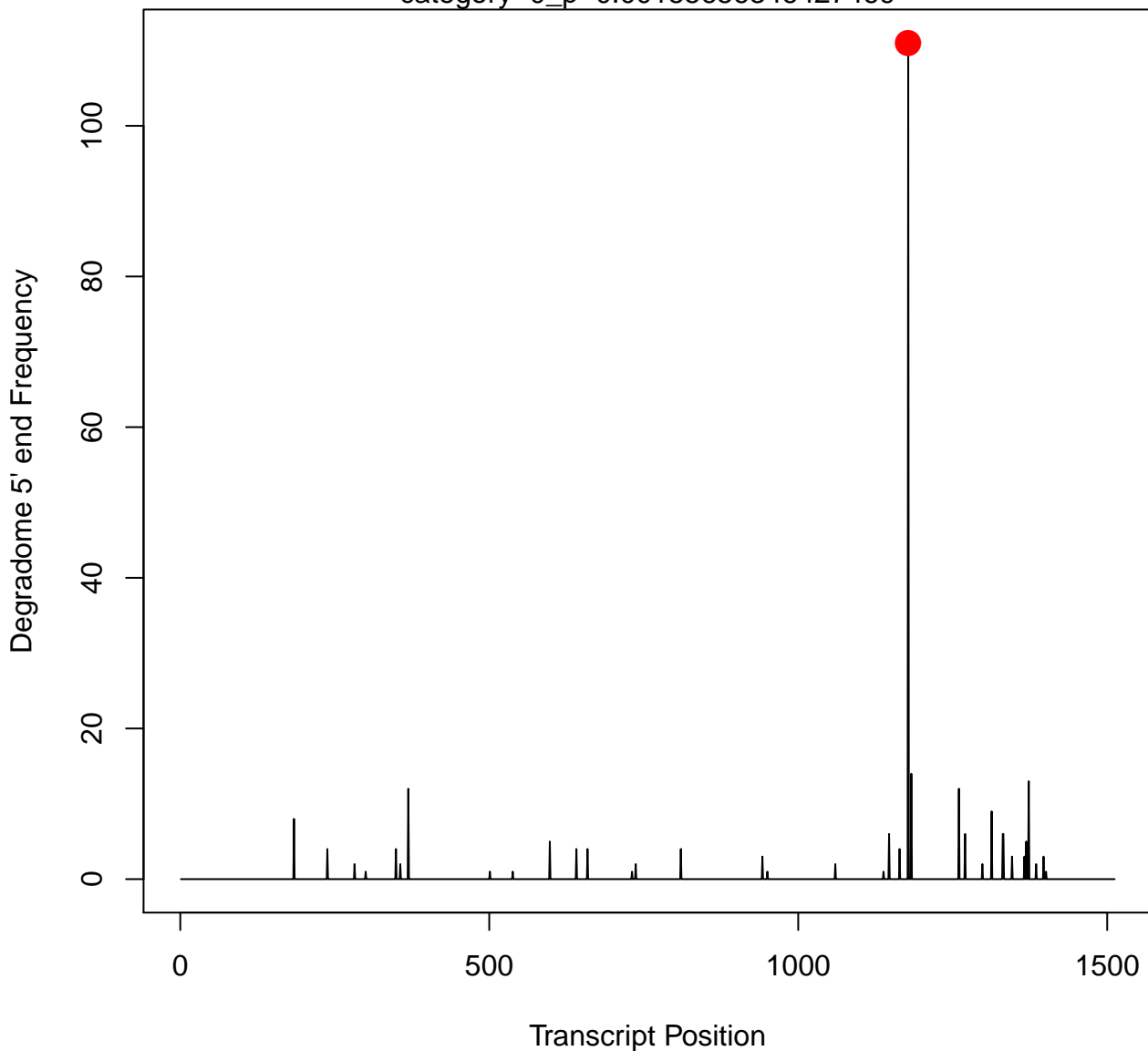

Supplement: Supplementary file 1 [file DataSheet_1.zip › The miRNA-target modules identified by the CleaveLand4/miR160c-5p_evm.model.LG01.2348_1178_TPlot.pdf]

**T=evm.model.LG01.6149\_Q=miR160c-5p\_S=1325**

category=0\_p=0.00207487934604456

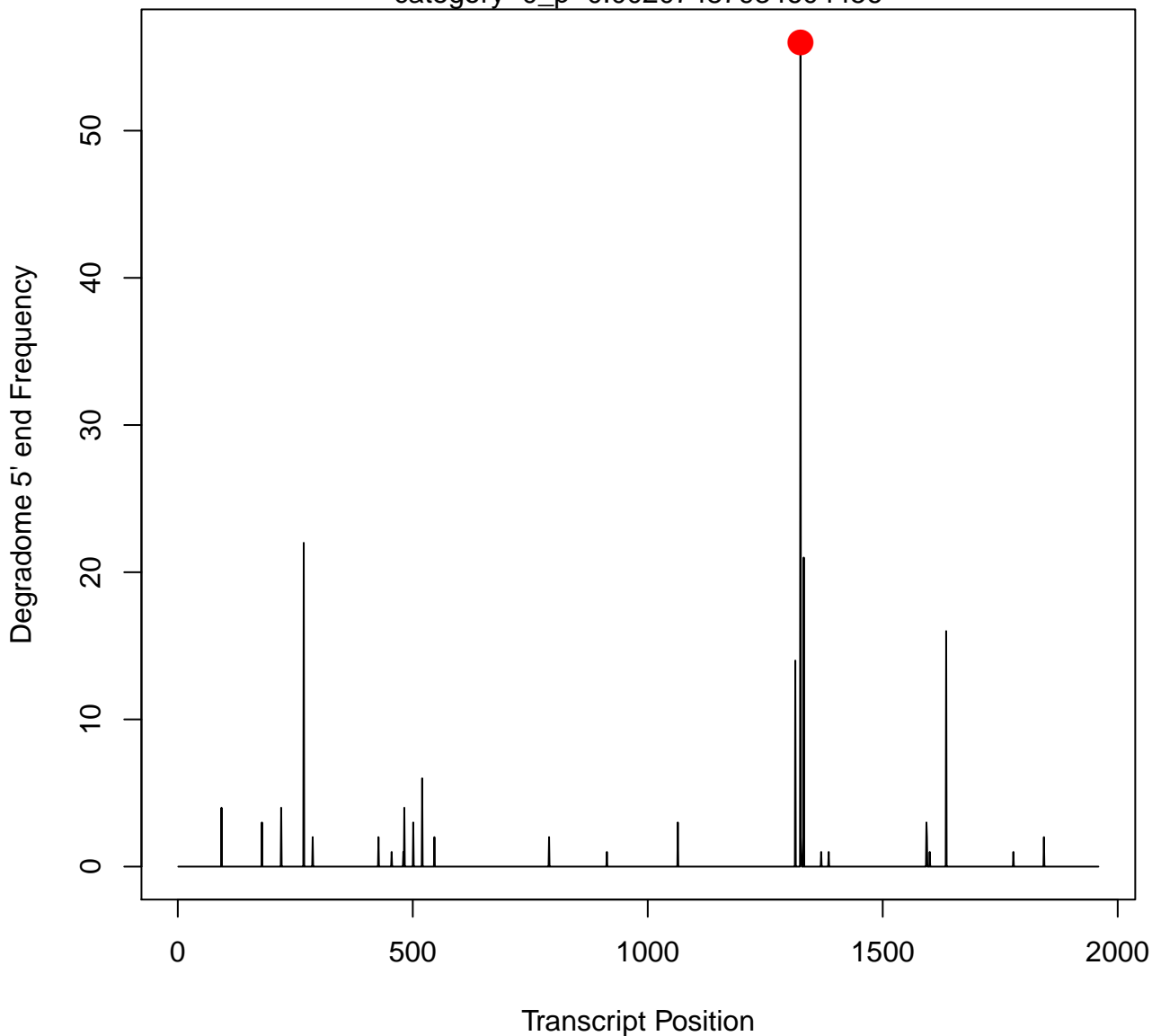

Supplement: Supplementary file 1 [file DataSheet_1.zip › The miRNA-target modules identified by the CleaveLand4/miR160c-5p_evm.model.LG01.6149_1325_TPlot.pdf]

**T=evm.model.LG01.2348\_Q=miR160d-5p\_S=1178**

category=0\_p=0.00155656346427469

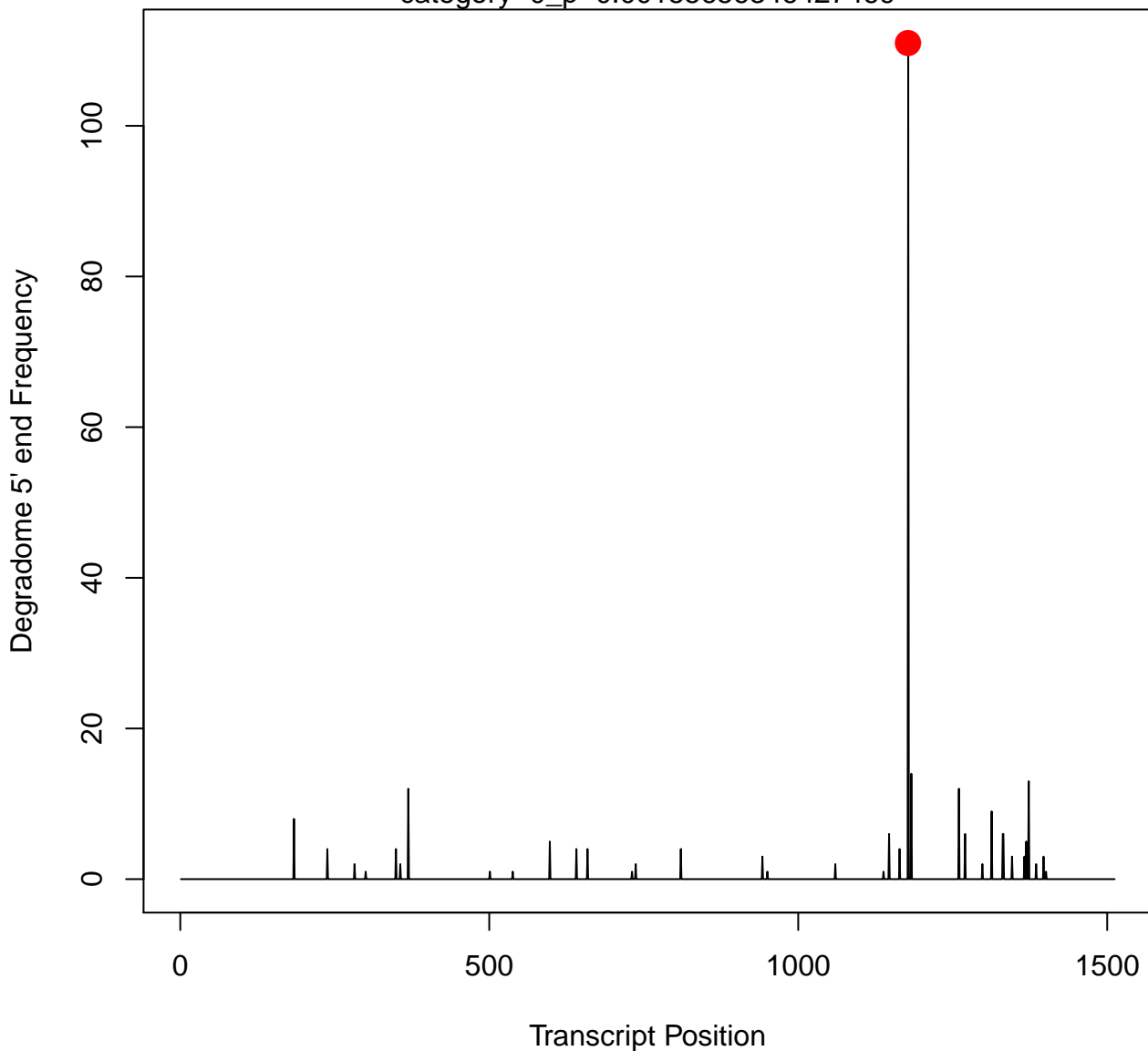

Supplement: Supplementary file 1 [file DataSheet_1.zip › The miRNA-target modules identified by the CleaveLand4/miR160d-5p_evm.model.LG01.2348_1178_TPlot.pdf]

**T=evm.model.LG01.6149\_Q=miR160d-5p\_S=1325**

category=0\_p=0.00207487934604456

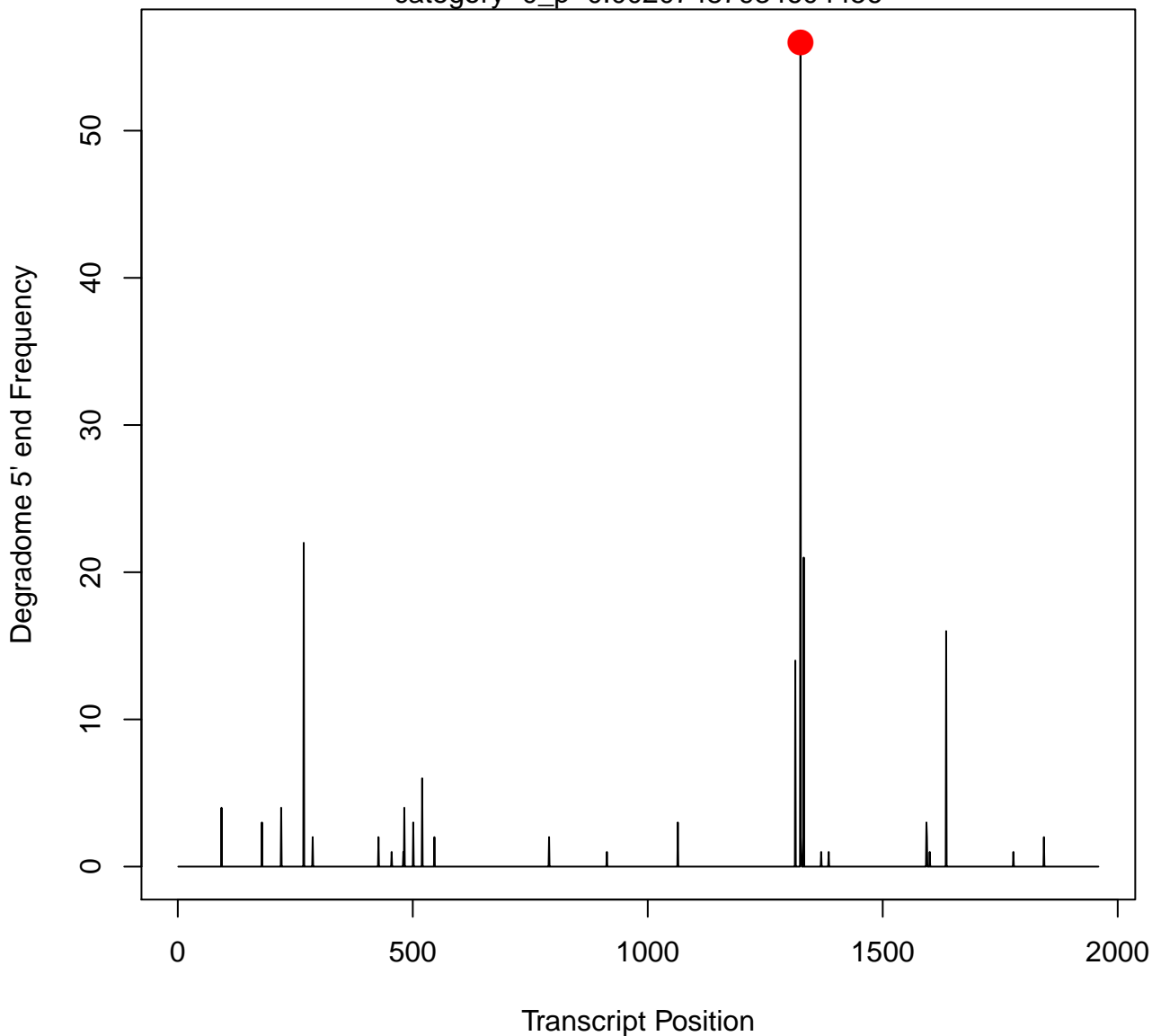

Supplement: Supplementary file 1 [file DataSheet_1.zip › The miRNA-target modules identified by the CleaveLand4/miR160d-5p_evm.model.LG01.6149_1325_TPlot.pdf]

**T=evm.model.LG05.25\_Q=miR162-3p\_S=2829**

category=3\_p=0.012146249673191

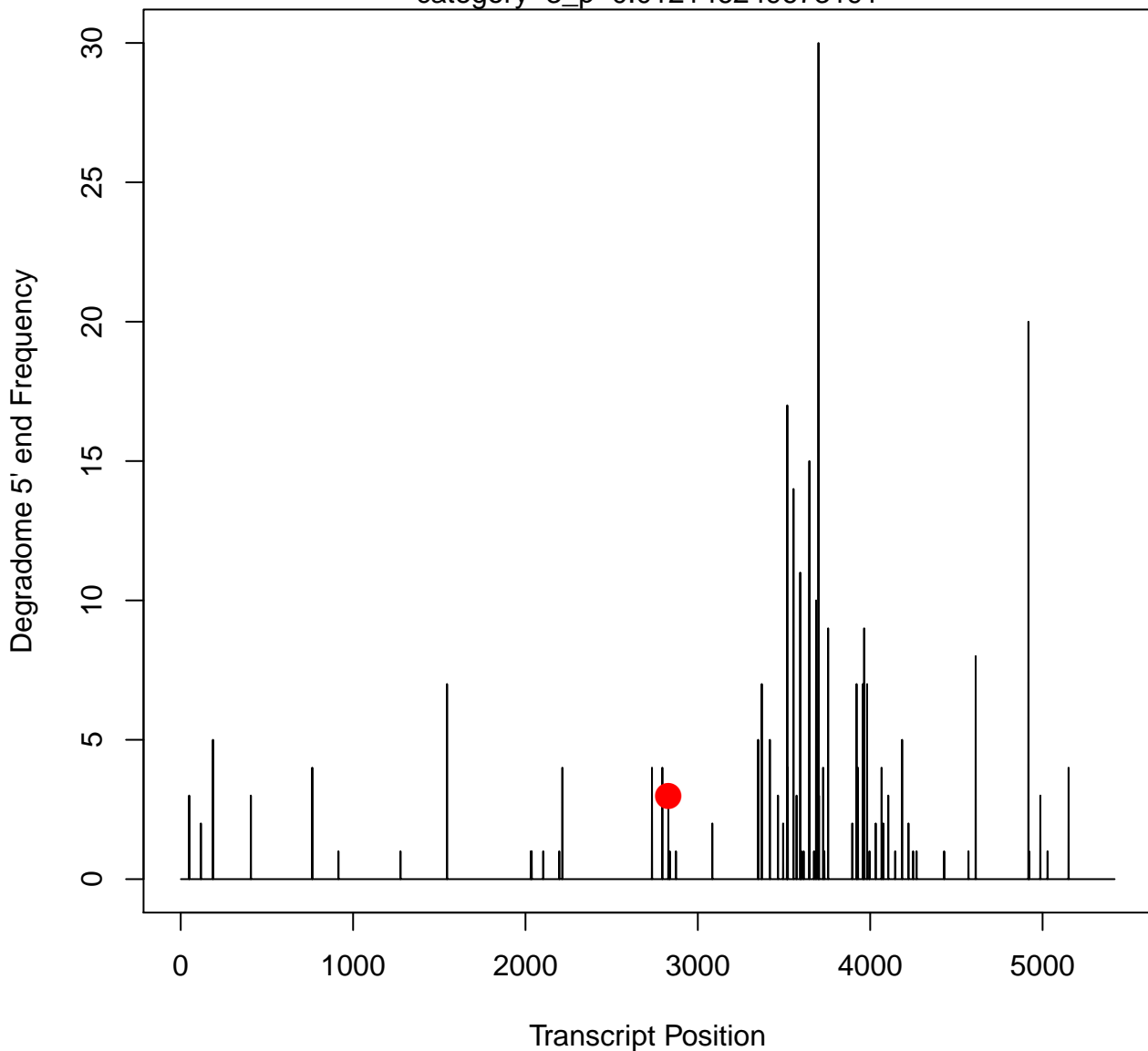

Supplement: Supplementary file 1 [file DataSheet_1.zip › The miRNA-target modules identified by the CleaveLand4/miR162-3p_evm.model.LG05.25_2829_TPlot.pdf]

**T=evm.model.LG03.2517\_Q=miR164a-5p\_S=649**

category=0\_p=0.00155656346427469

Degradome 5' end Frequency

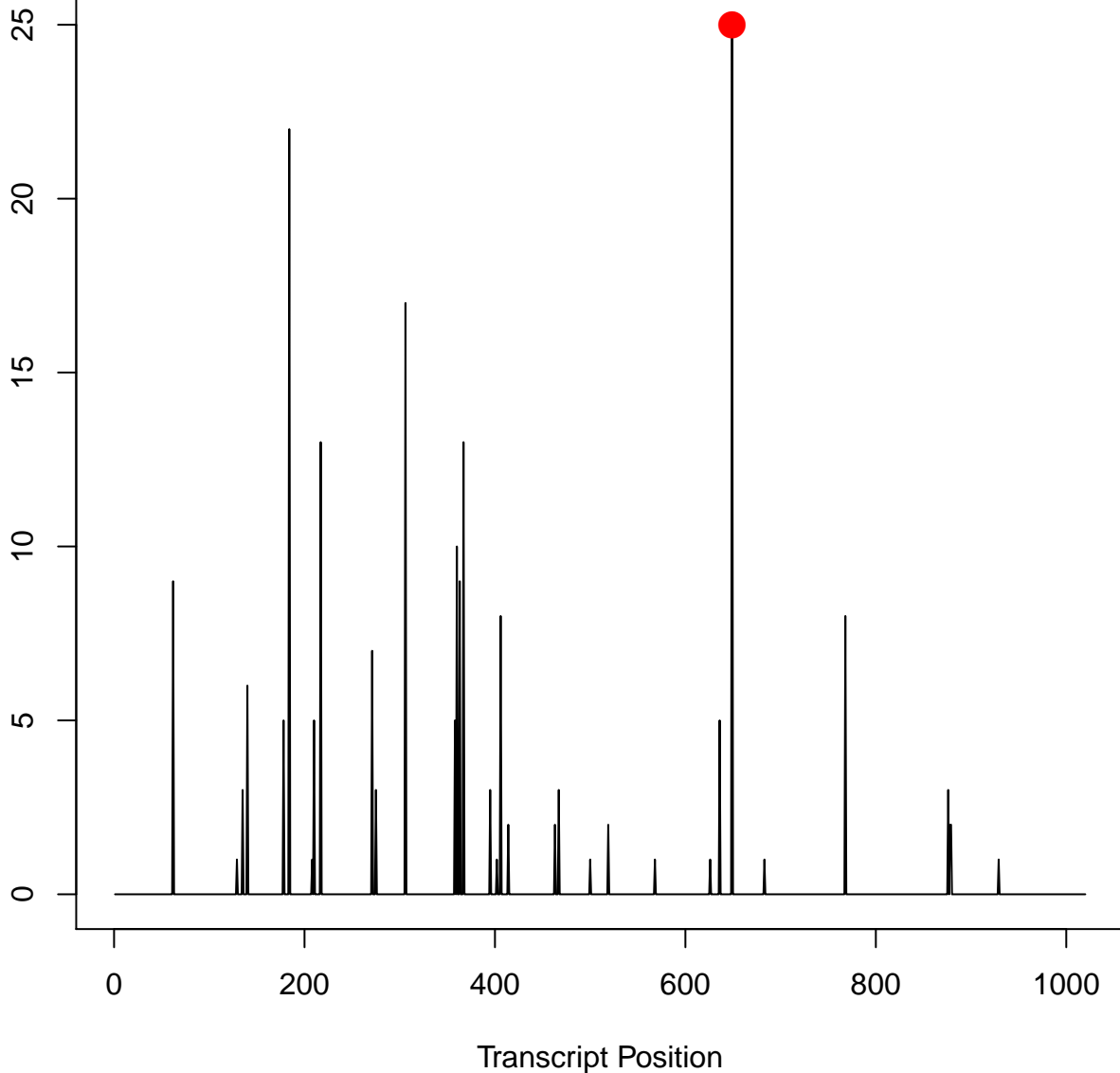

Supplement: Supplementary file 1 [file DataSheet_1.zip › The miRNA-target modules identified by the CleaveLand4/miR164a-5p_evm.model.LG03.2517_649_TPlot.pdf]

**T=evm.model.LG06.509\_Q=miR164b-5p\_S=655**

category=2\_p=0.0345432623396535

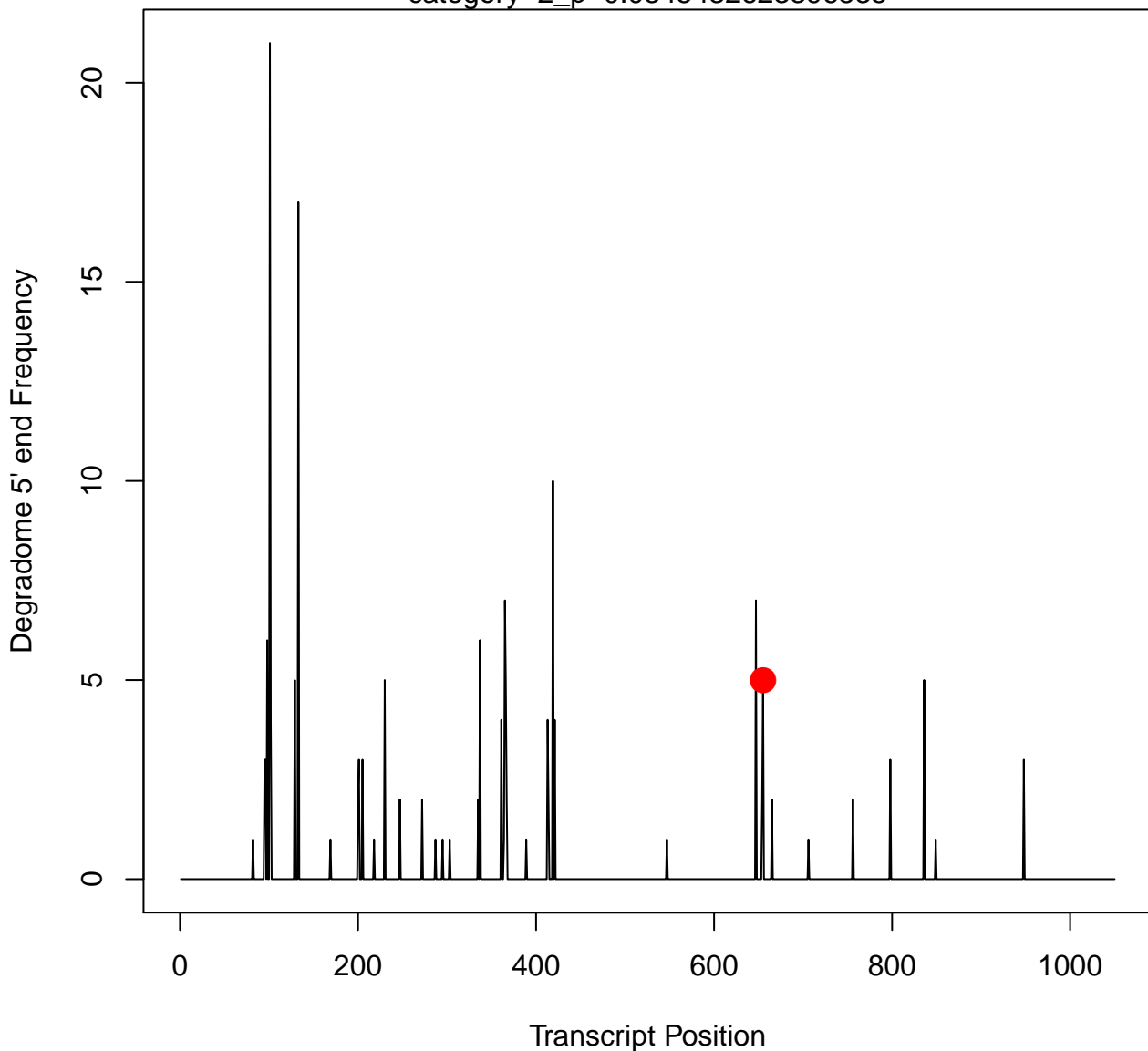

Supplement: Supplementary file 1 [file DataSheet_1.zip › The miRNA-target modules identified by the CleaveLand4/miR164b-5p_evm.model.LG06.509_655_TPlot.pdf]

**T=evm.model.LG07.3896\_Q=miR164b-5p\_S=664**

category=0\_p=0.00103797837257324

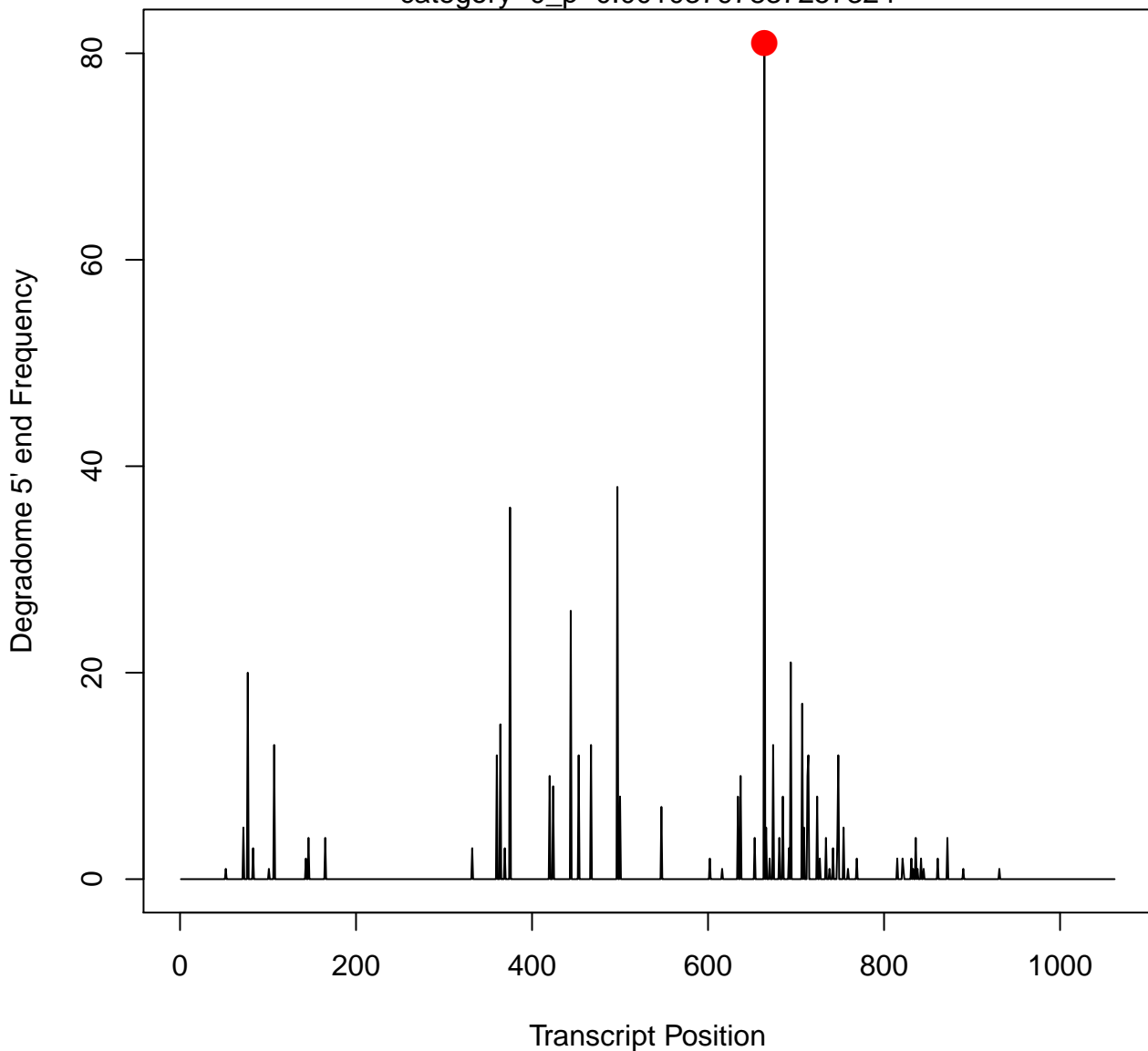

Supplement: Supplementary file 1 [file DataSheet_1.zip › The miRNA-target modules identified by the CleaveLand4/miR164b-5p_evm.model.LG07.3896_664_TPlot.pdf]

**T=evm.model.LG06.509\_Q=miR164c-5p\_S=655**

category=2\_p=0.0260209548157995

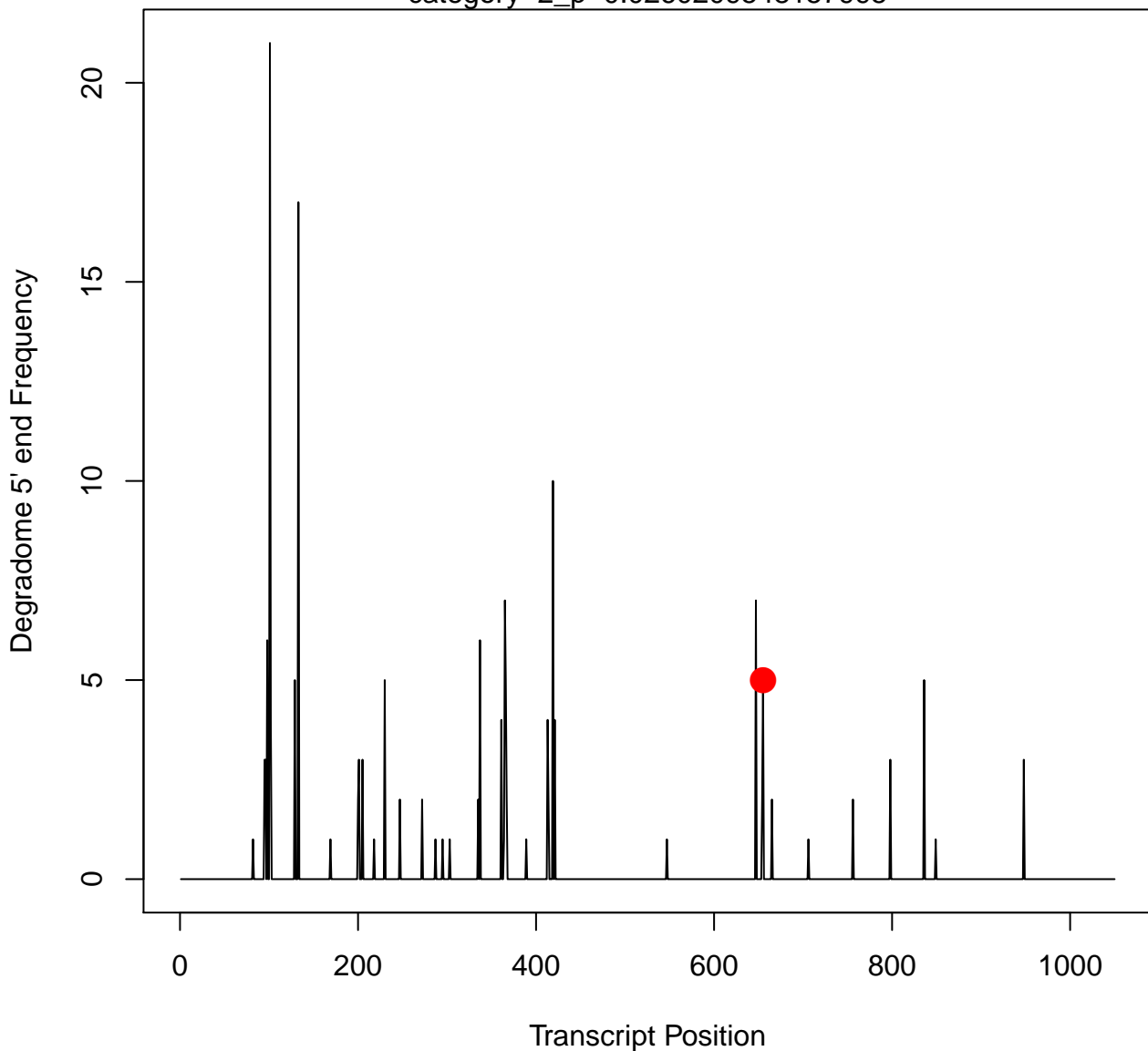

Supplement: Supplementary file 1 [file DataSheet_1.zip › The miRNA-target modules identified by the CleaveLand4/miR164c-5p_evm.model.LG06.509_655_TPlot.pdf]

**T=evm.model.LG07.3896\_Q=miR164c-5p\_S=664**

category=0\_p=0.00051912393111464

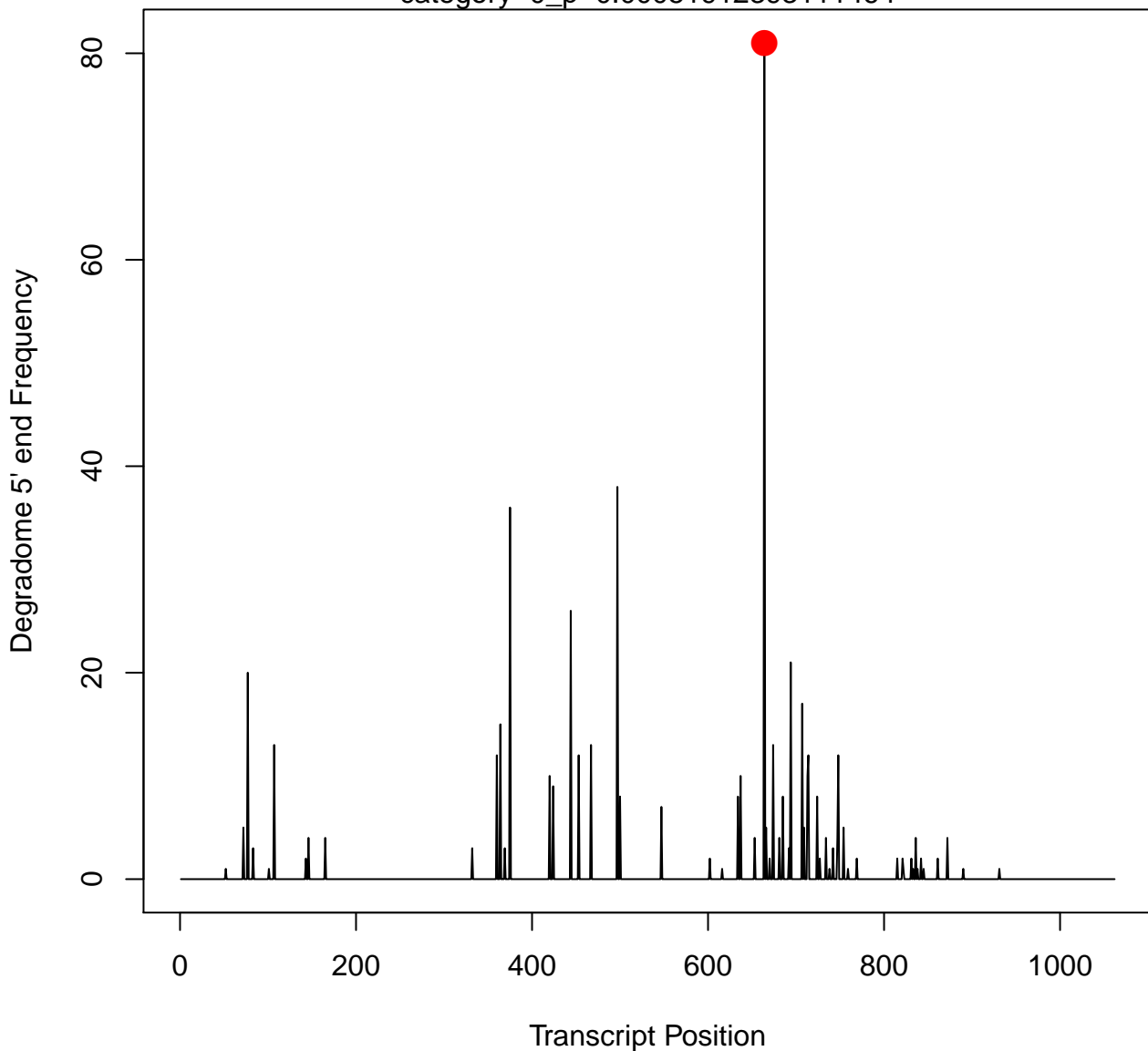

Supplement: Supplementary file 1 [file DataSheet_1.zip › The miRNA-target modules identified by the CleaveLand4/miR164c-5p_evm.model.LG07.3896_664_TPlot.pdf]

**T=evm.model.LG03.2517\_Q=miR164e-5p\_S=649**

category=0\_p=0.00155656346427469

Degradsome 5' end Frequency

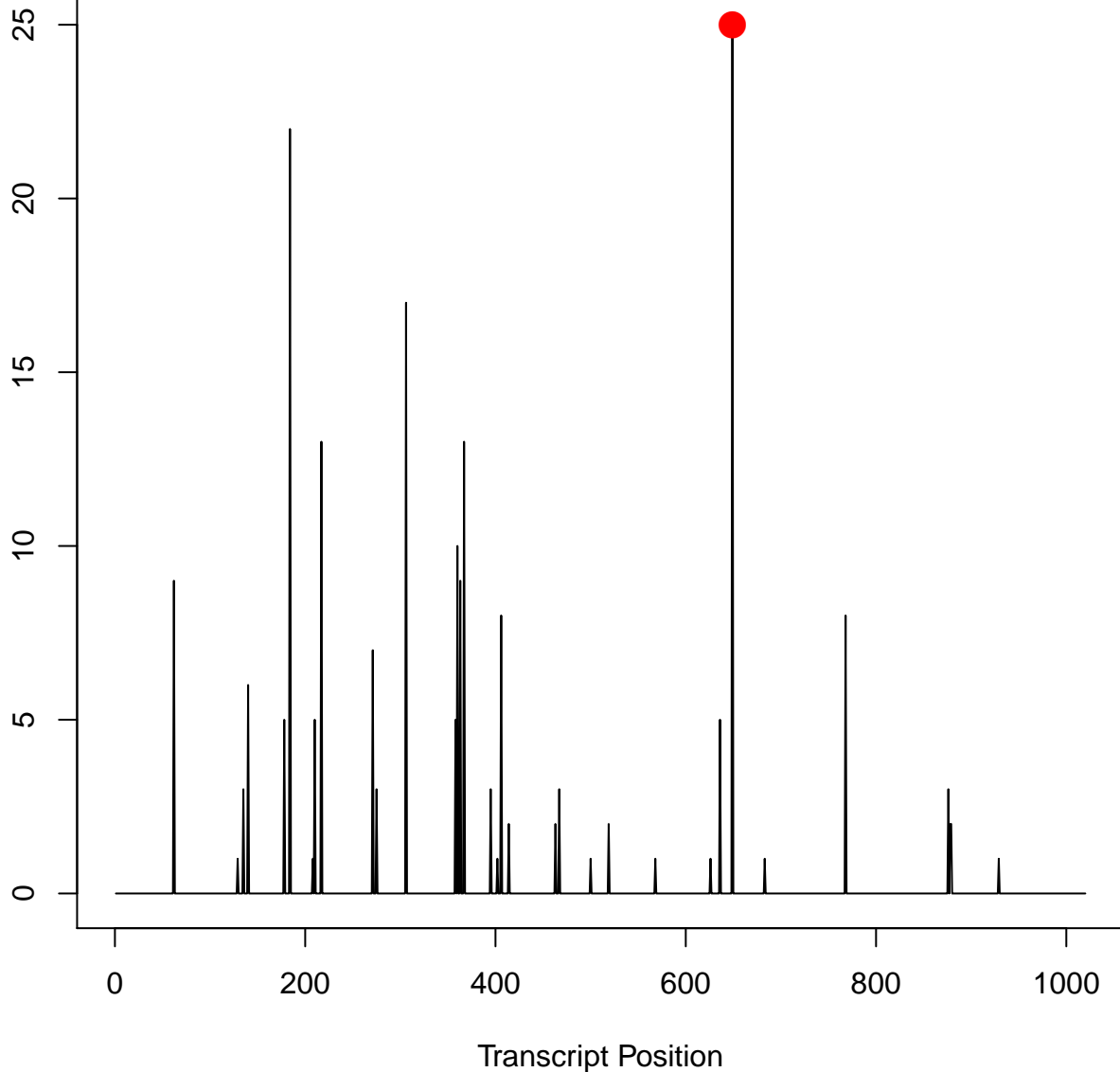

Supplement: Supplementary file 1 [file DataSheet_1.zip › The miRNA-target modules identified by the CleaveLand4/miR164e-5p_evm.model.LG03.2517_649_TPlot.pdf]

**T=evm.model.LG01.6147\_Q=miR166a-3p\_S=577**

category=0\_p=0.00207487934604456

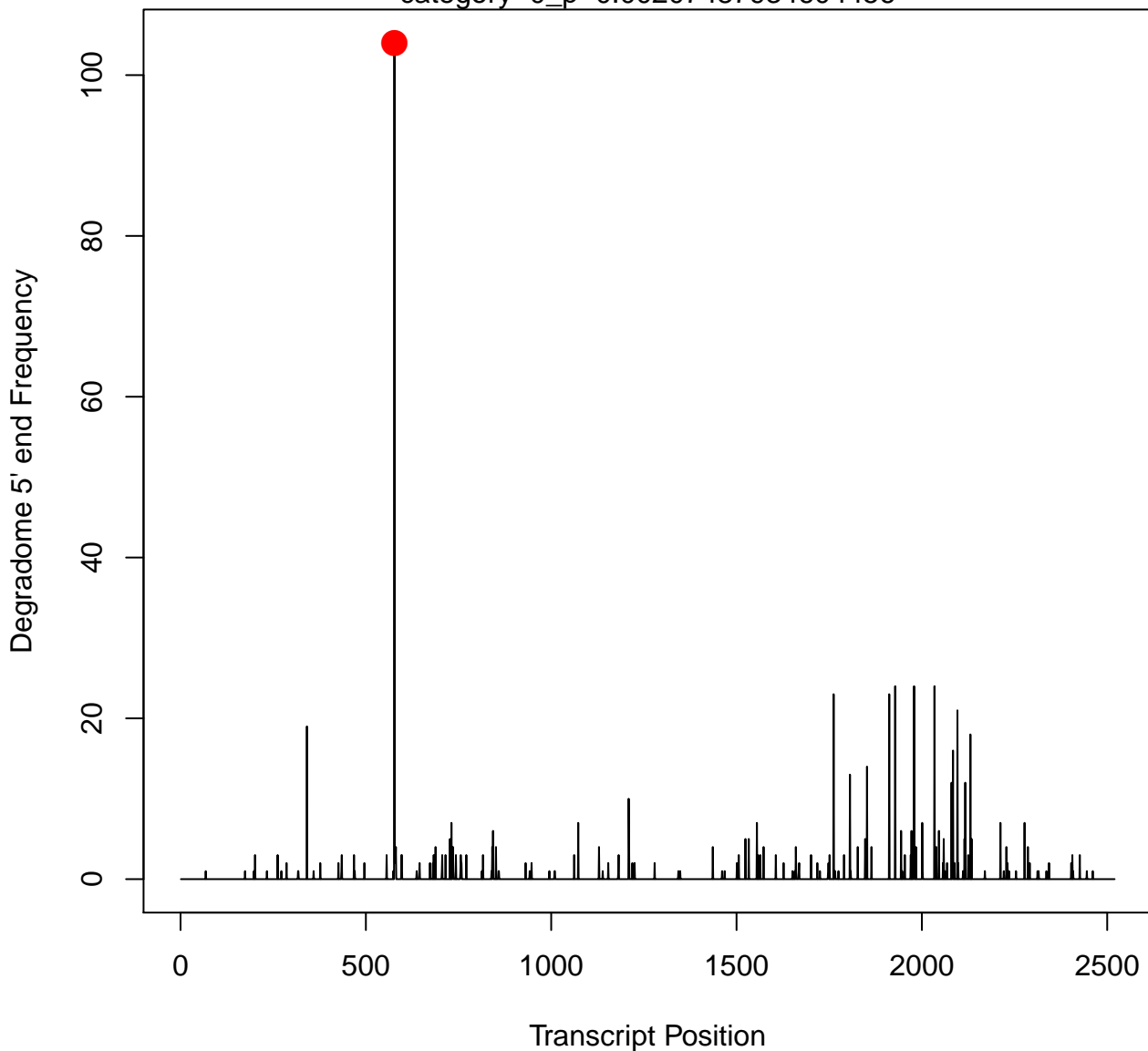

Supplement: Supplementary file 1 [file DataSheet_1.zip › The miRNA-target modules identified by the CleaveLand4/miR166a-3p_evm.model.LG01.6147_577_TPlot.pdf]

**T=evm.model.LG02.5446\_Q=miR166a-3p\_S=565**

category=2\_p=0.042990999752487

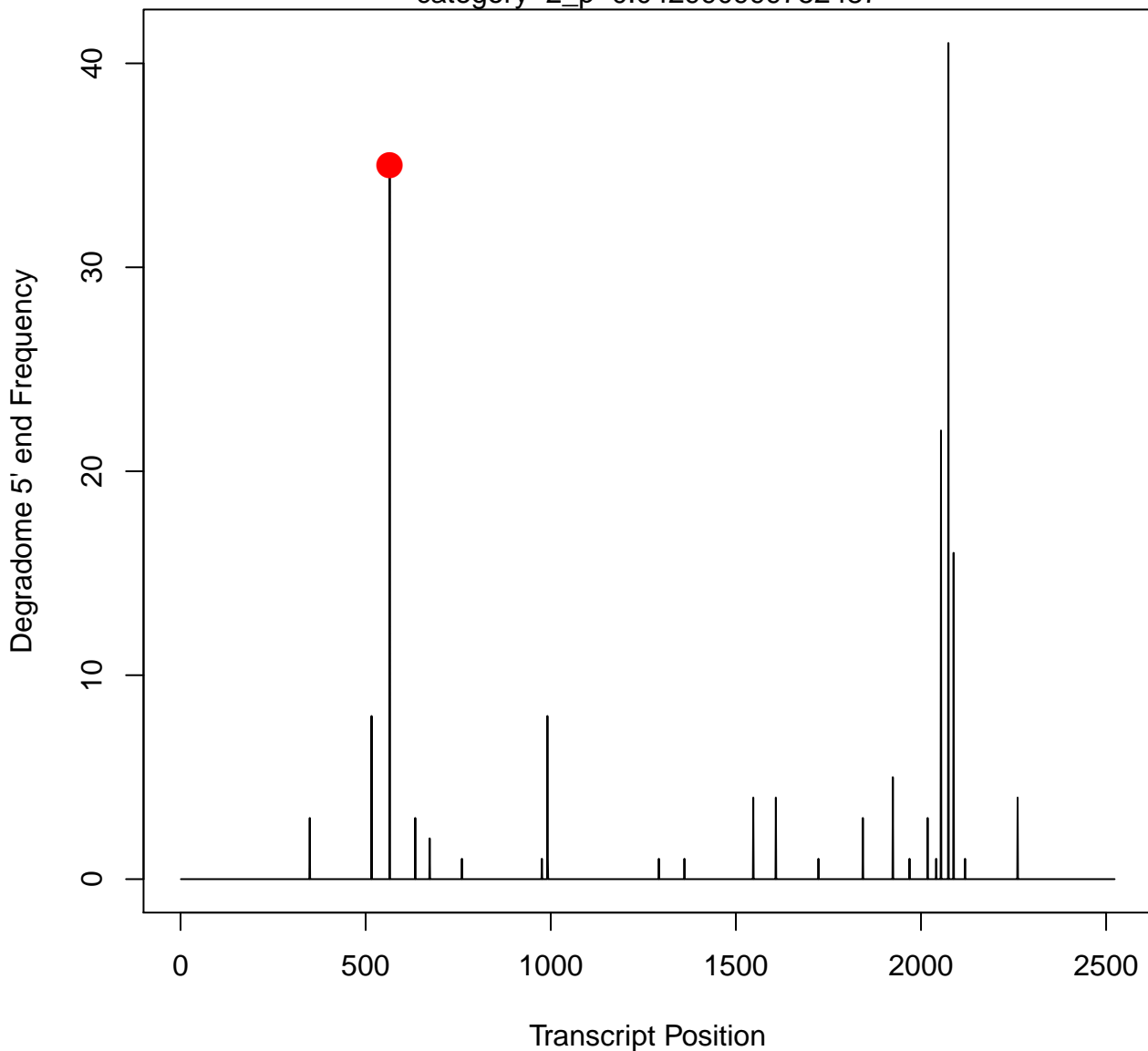

Supplement: Supplementary file 1 [file DataSheet_1.zip › The miRNA-target modules identified by the CleaveLand4/miR166a-3p_evm.model.LG02.5446_565_TPlot.pdf]

**T=evm.model.LG07.3497\_Q=miR166a-3p\_S=547**

category=0\_p=0.00051912393111464

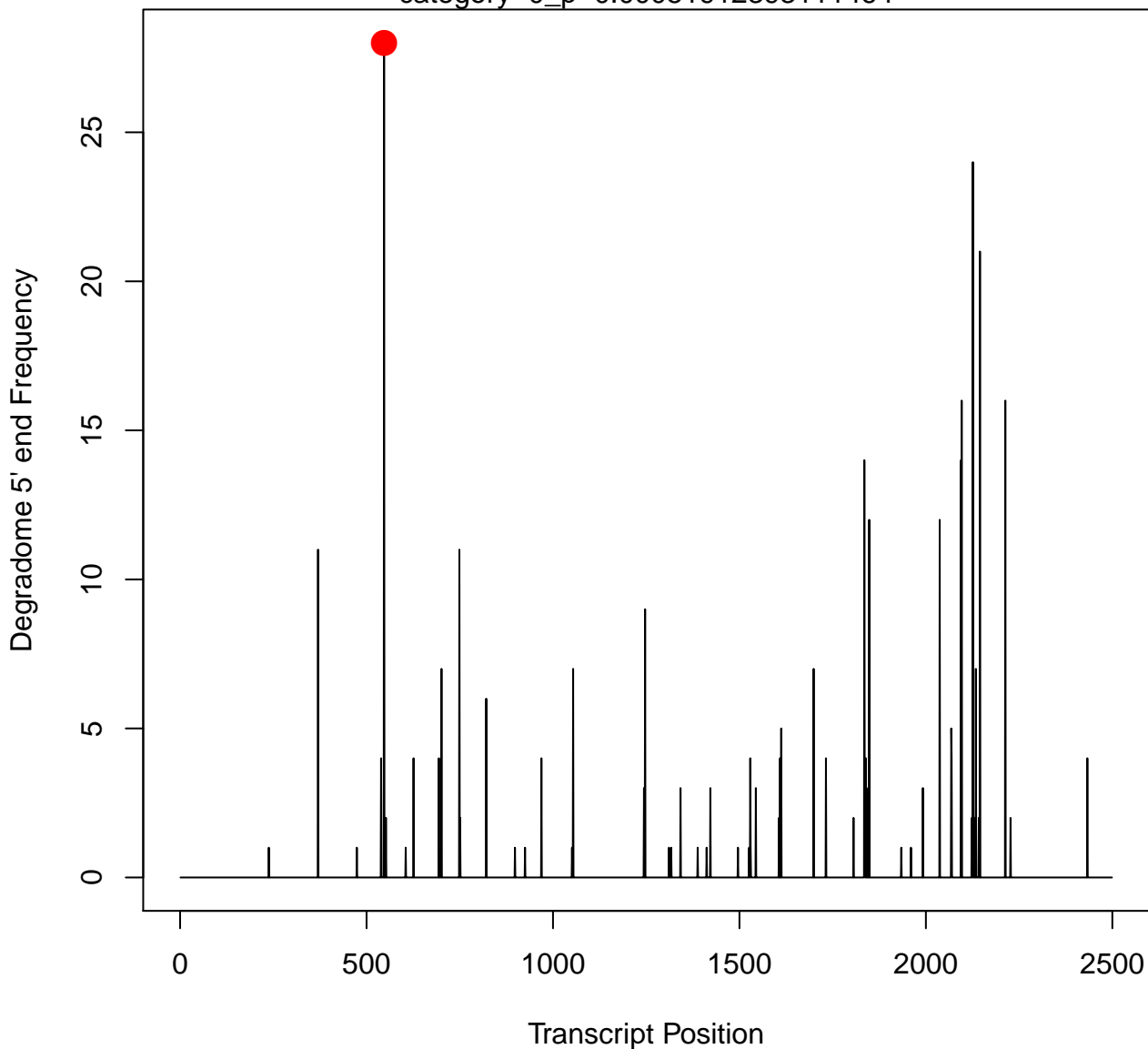

Supplement: Supplementary file 1 [file DataSheet_1.zip › The miRNA-target modules identified by the CleaveLand4/miR166a-3p_evm.model.LG07.3497_547_TPlot.pdf]

**T=evm.model.LG08.2054\_Q=miR166b-5p\_S=436**

category=2\_p=0.0174234189334928

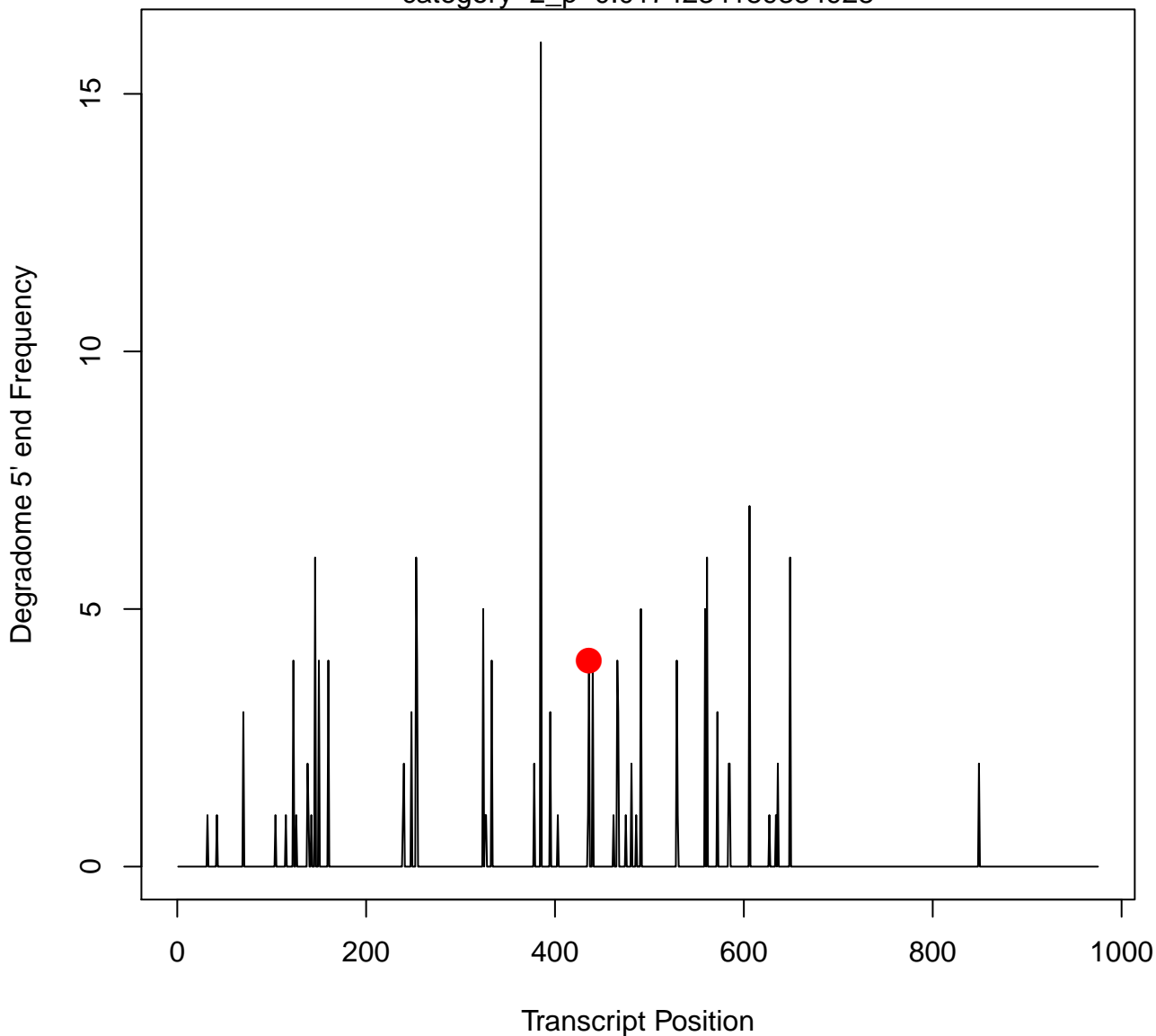

Supplement: Supplementary file 1 [file DataSheet_1.zip › The miRNA-target modules identified by the CleaveLand4/miR166b-5p_evm.model.LG08.2054_436_TPlot.pdf]

**T=evm.model.LG02.2189\_Q=miR166e-3p\_S=562**

category=0\_p=0.00155656346427469

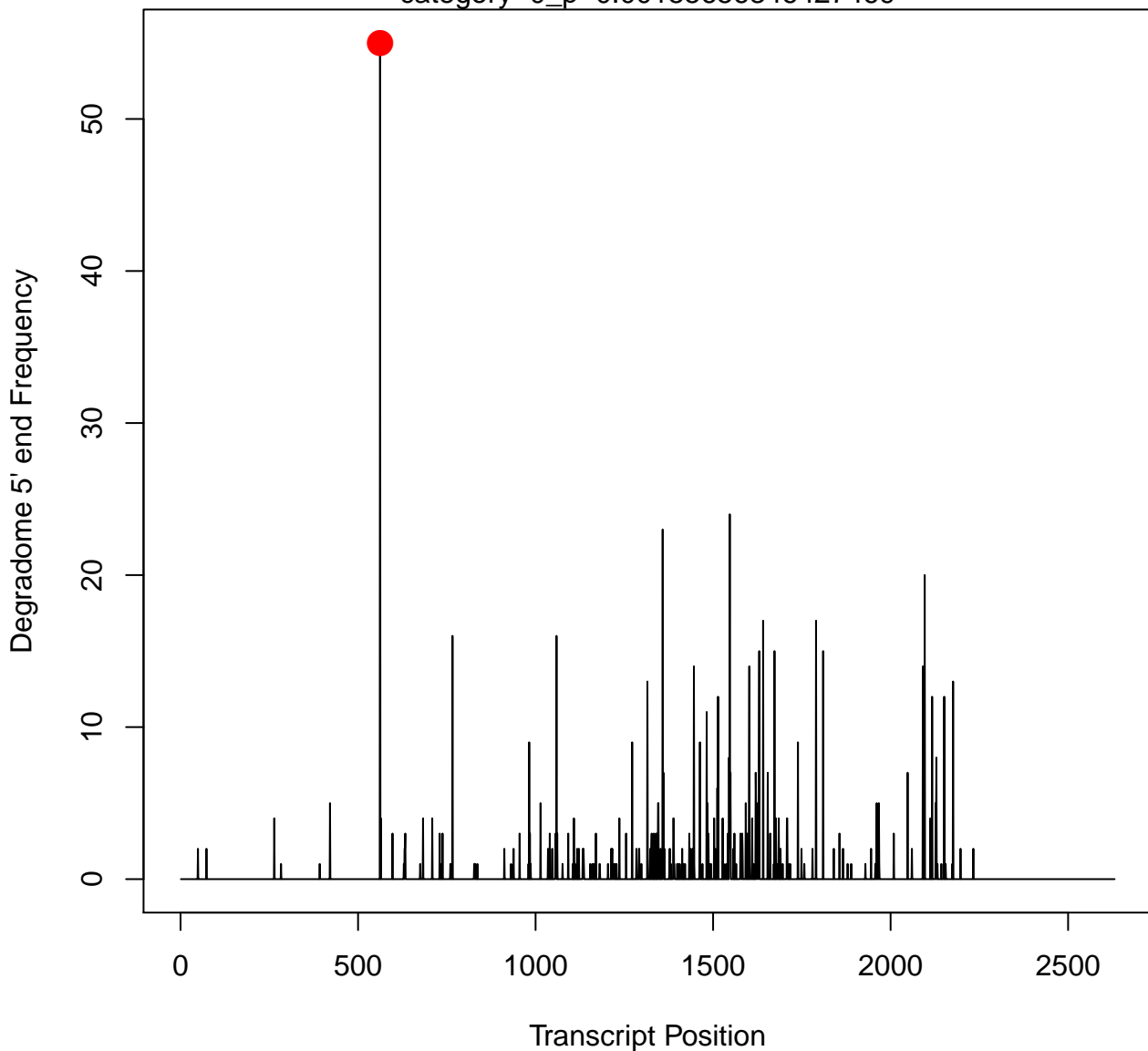

Supplement: Supplementary file 1 [file DataSheet_1.zip › The miRNA-target modules identified by the CleaveLand4/miR166e-3p_evm.model.LG02.2189_562_TPlot.pdf]

**T=evm.model.LG03.2778\_Q=miR166e-3p\_S=550**

category=2\_p=0.0174234189334928

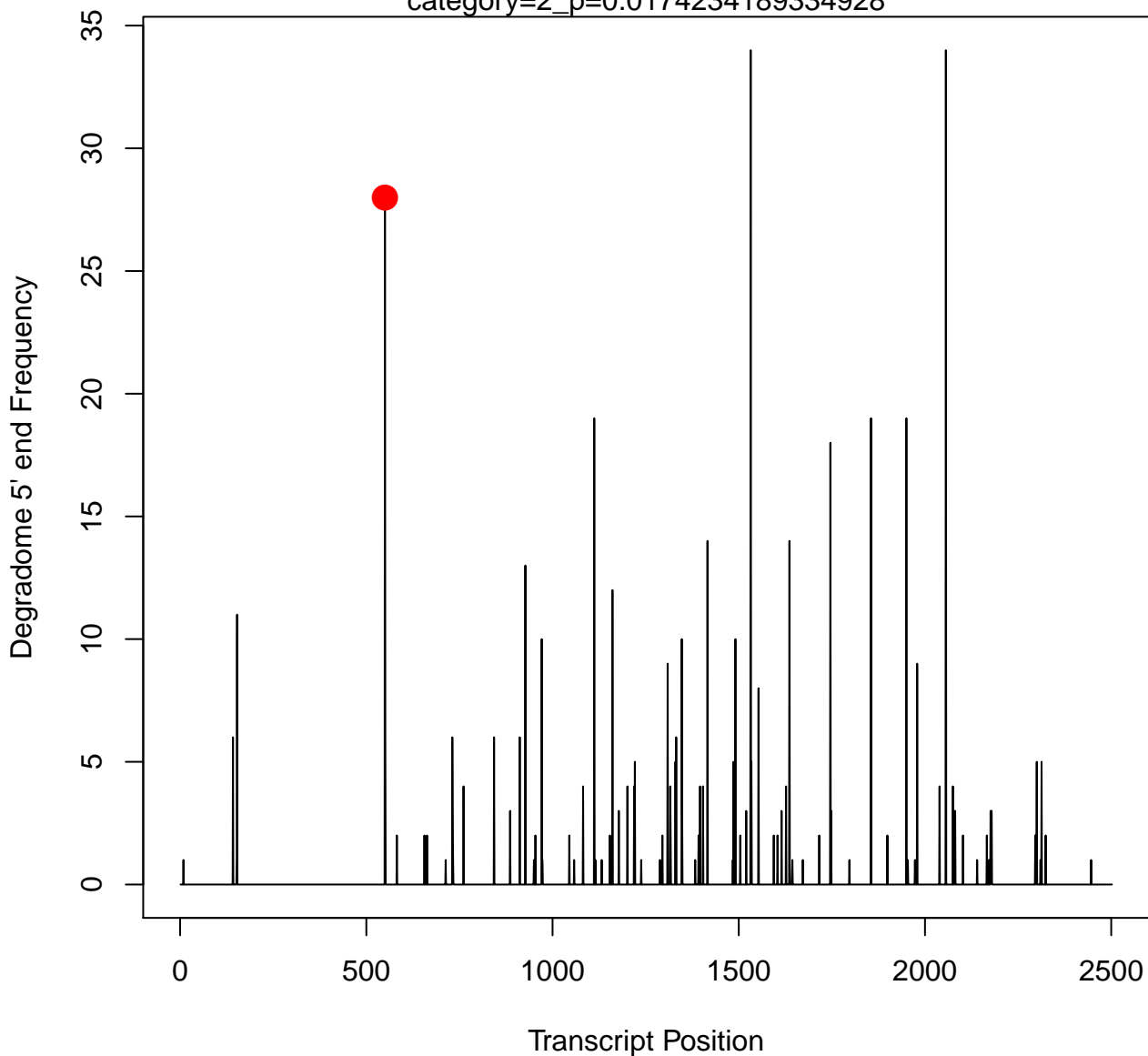

Supplement: Supplementary file 1 [file DataSheet_1.zip › The miRNA-target modules identified by the CleaveLand4/miR166e-3p_evm.model.LG03.2778_550_TPlot.pdf]

**T=evm.model.LG02.2189\_Q=miR166i-3p\_S=564**

category=2\_p=0.0174234189334928

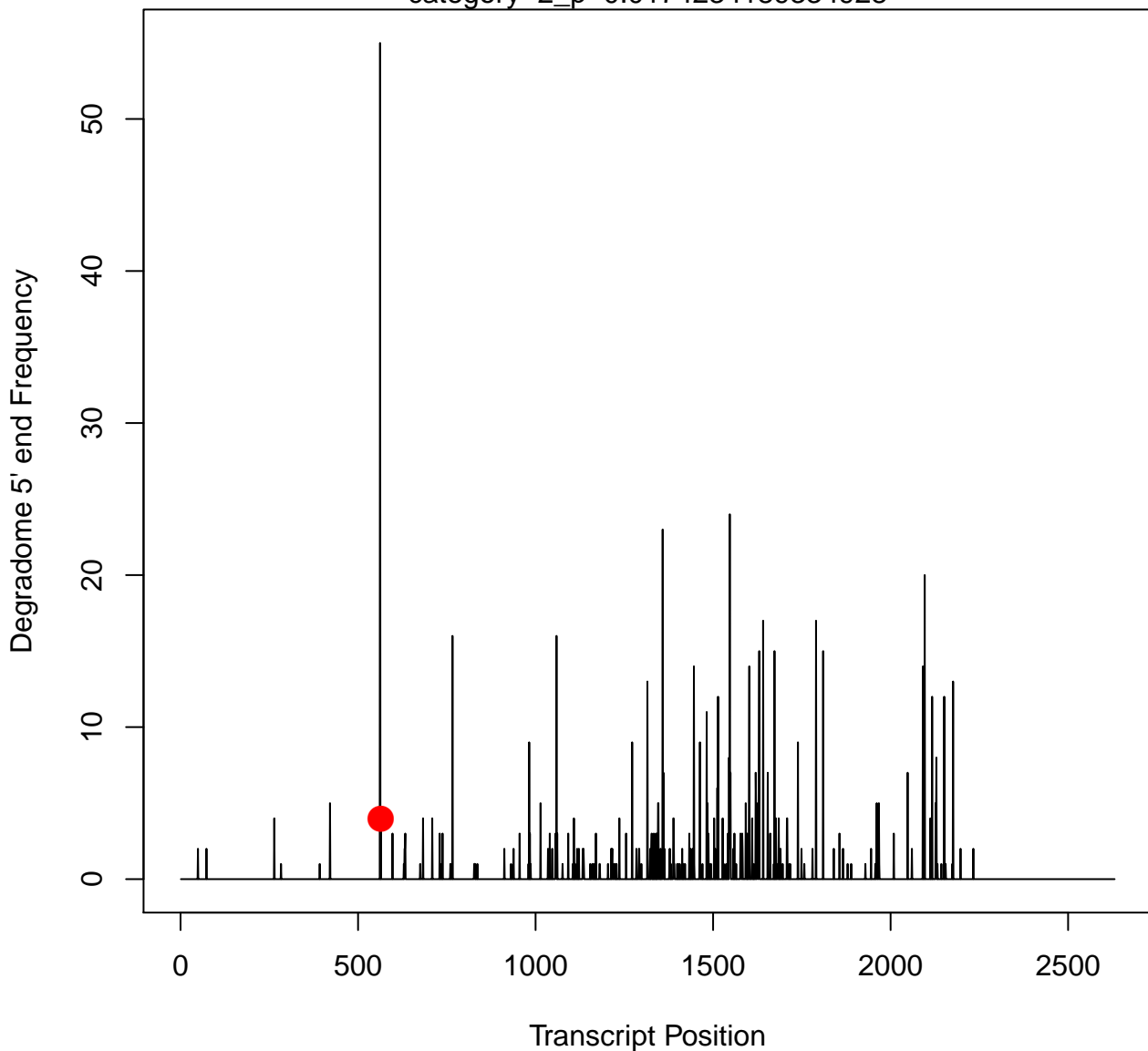

Supplement: Supplementary file 1 [file DataSheet_1.zip › The miRNA-target modules identified by the CleaveLand4/miR166i-3p_evm.model.LG02.2189_564_TPlot.pdf]

**T=evm.model.LG07.3497\_Q=miR166i-3p\_S=549**

category=4\_p=0.00862274555333054

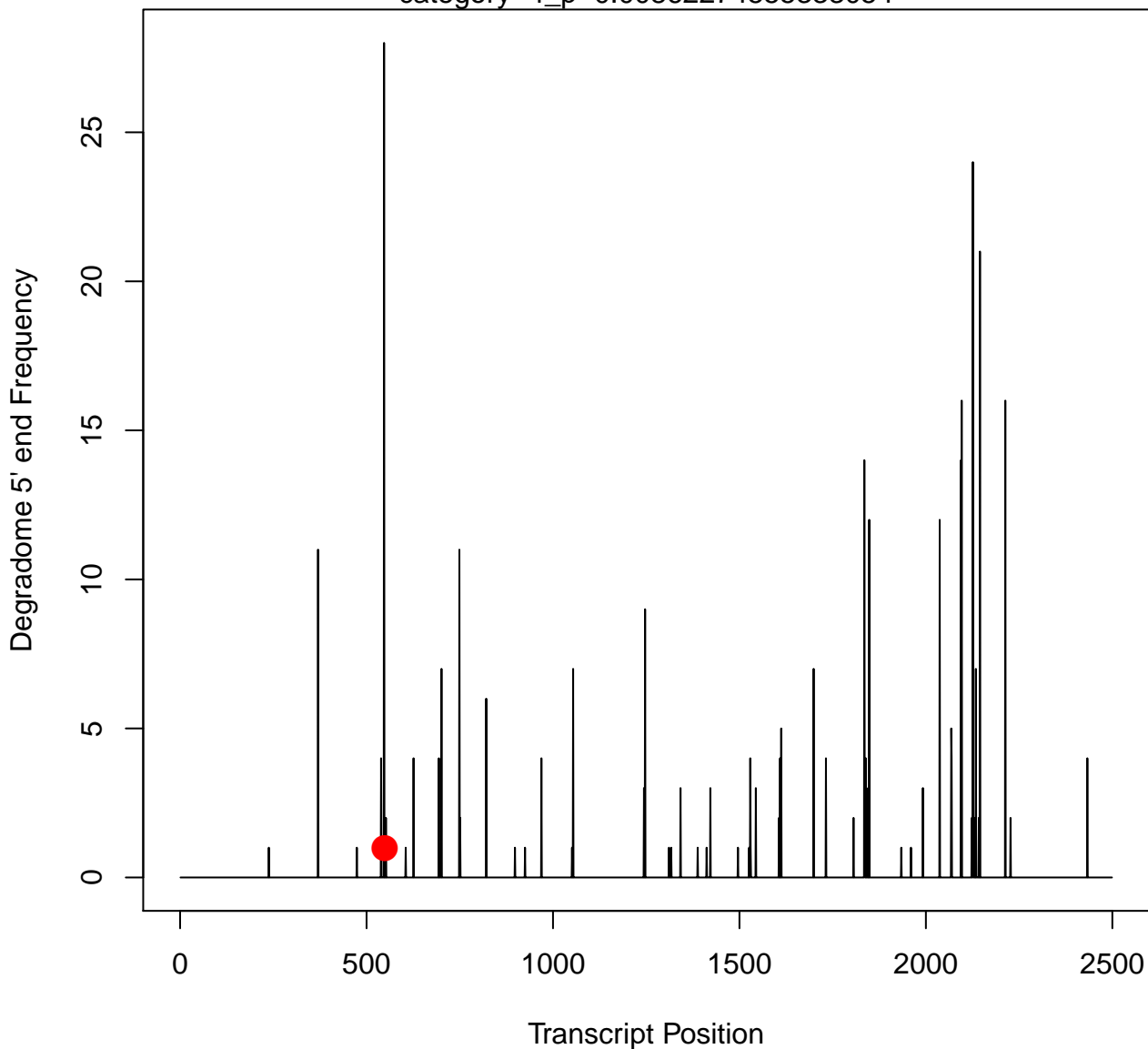

Supplement: Supplementary file 1 [file DataSheet_1.zip › The miRNA-target modules identified by the CleaveLand4/miR166i-3p_evm.model.LG07.3497_549_TPlot.pdf]

**T=evm.model.LG03.3558\_Q=miR166i-5p\_S=1127**

category=4\_p=0.0256458225535021

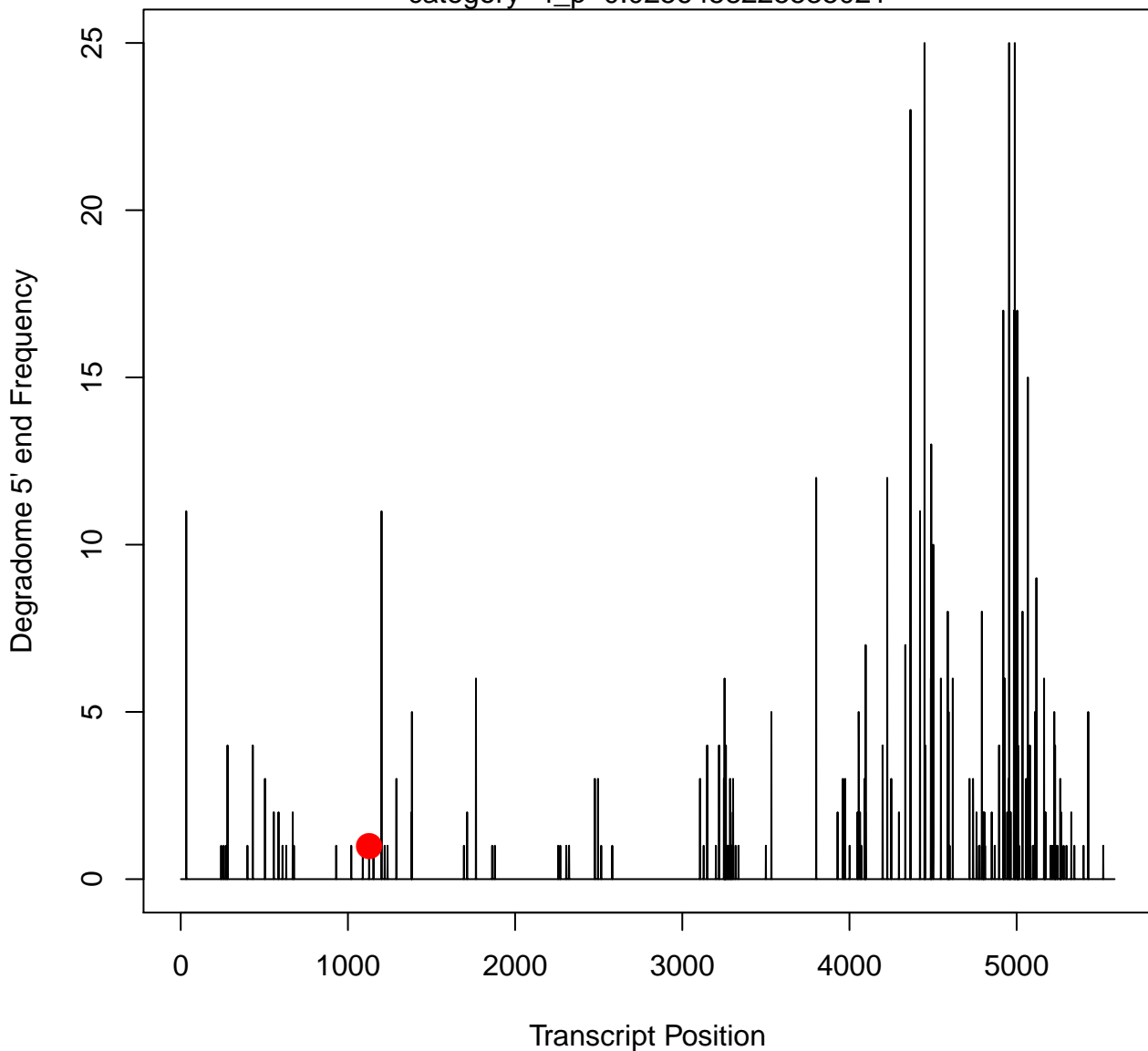

Supplement: Supplementary file 1 [file DataSheet_1.zip › The miRNA-target modules identified by the CleaveLand4/miR166i-5p_evm.model.LG03.3558_1127_TPlot.pdf]

**T=evm.model.LG02.2189\_Q=miR166j-3p\_S=562**

category=0\_p=0.00155656346427469

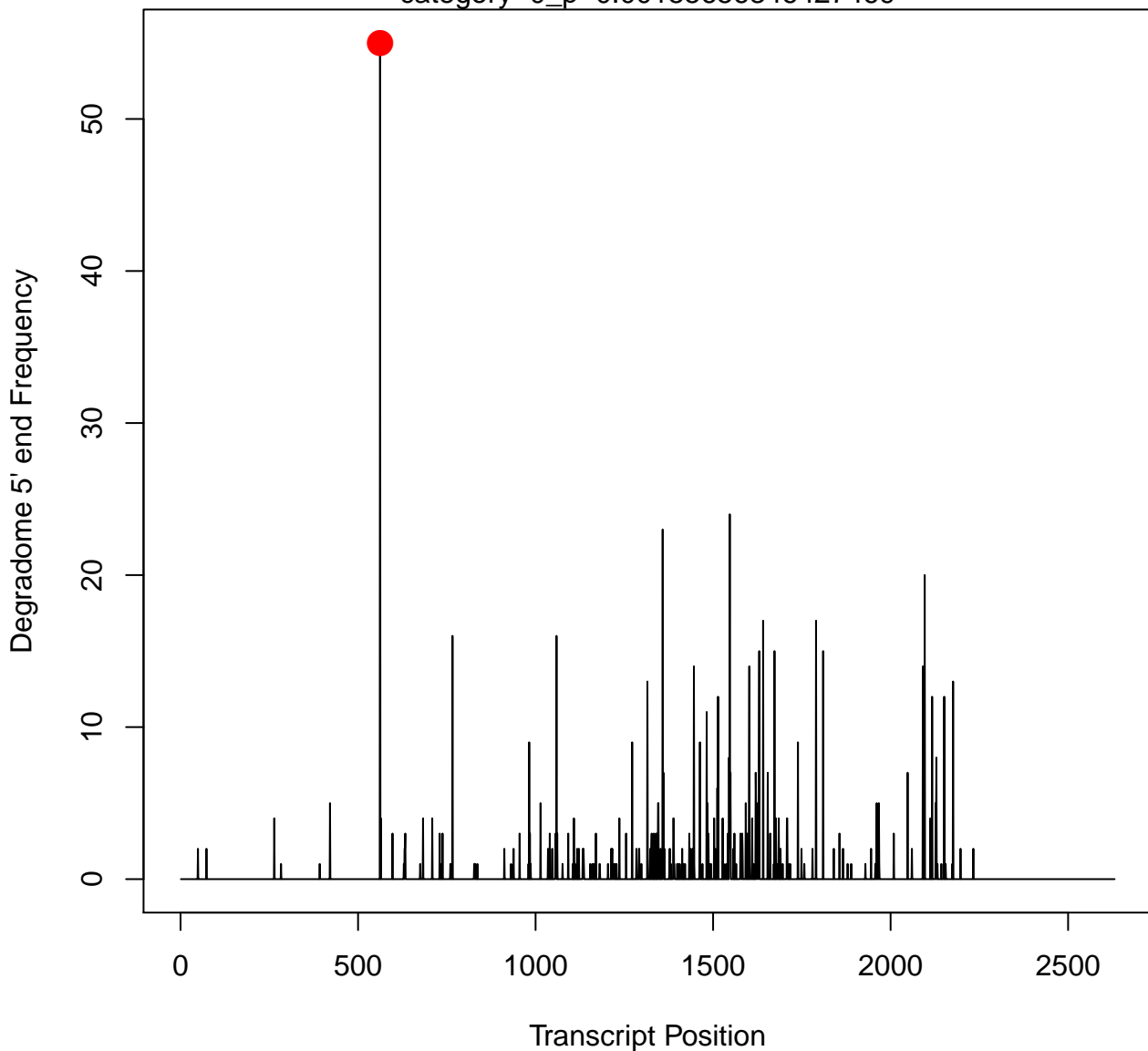

Supplement: Supplementary file 1 [file DataSheet_1.zip › The miRNA-target modules identified by the CleaveLand4/miR166j-3p_evm.model.LG02.2189_562_TPlot.pdf]

**T=evm.model.LG03.2778\_Q=miR166j-3p\_S=550**

category=2\_p=0.0174234189334928

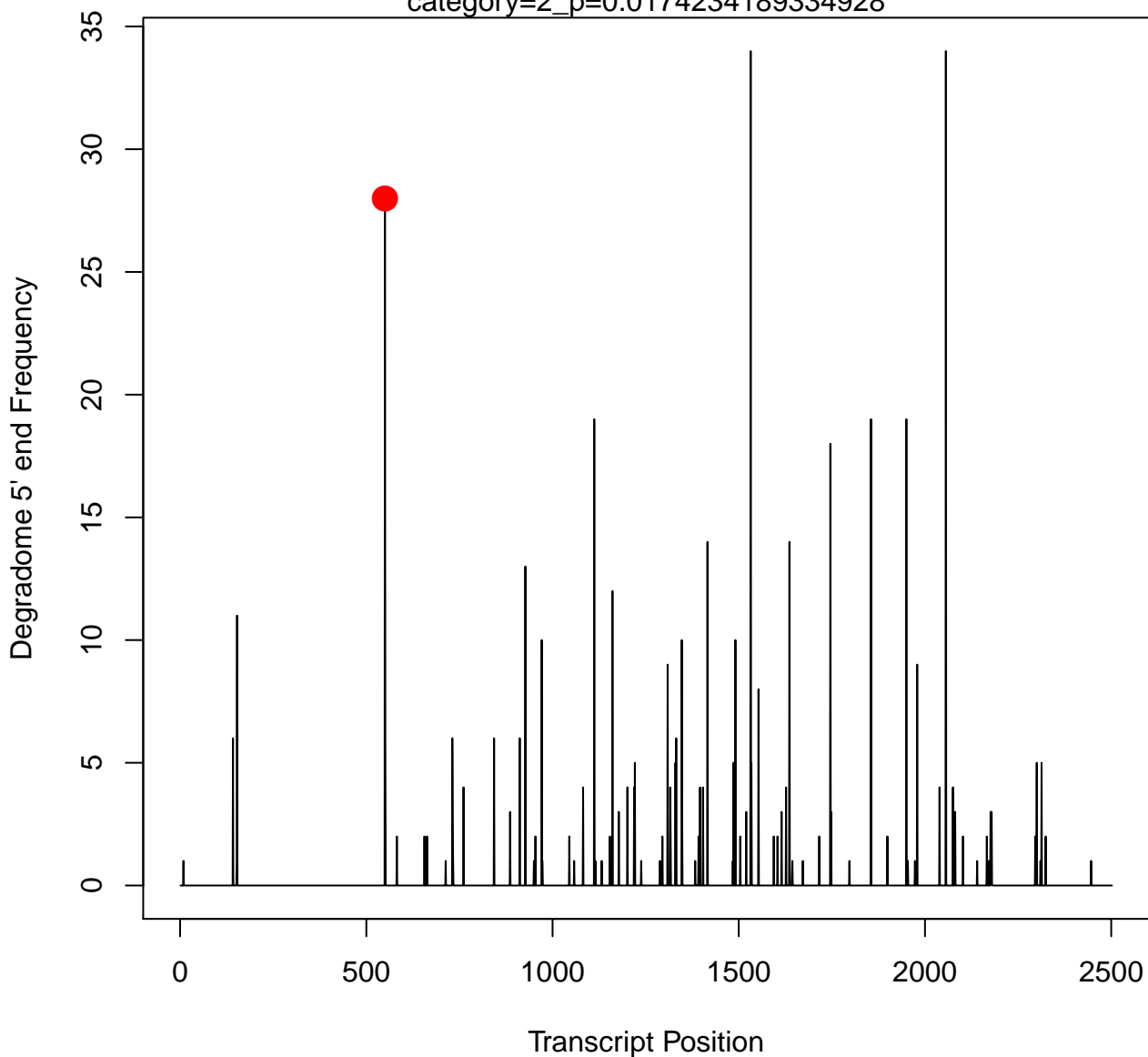

Supplement: Supplementary file 1 [file DataSheet_1.zip › The miRNA-target modules identified by the CleaveLand4/miR166j-3p_evm.model.LG03.2778_550_TPlot.pdf]

**T=evm.model.LG01.7049\_Q=miR167a-5p\_S=2222**

category=3\_p=0.0477069564524738

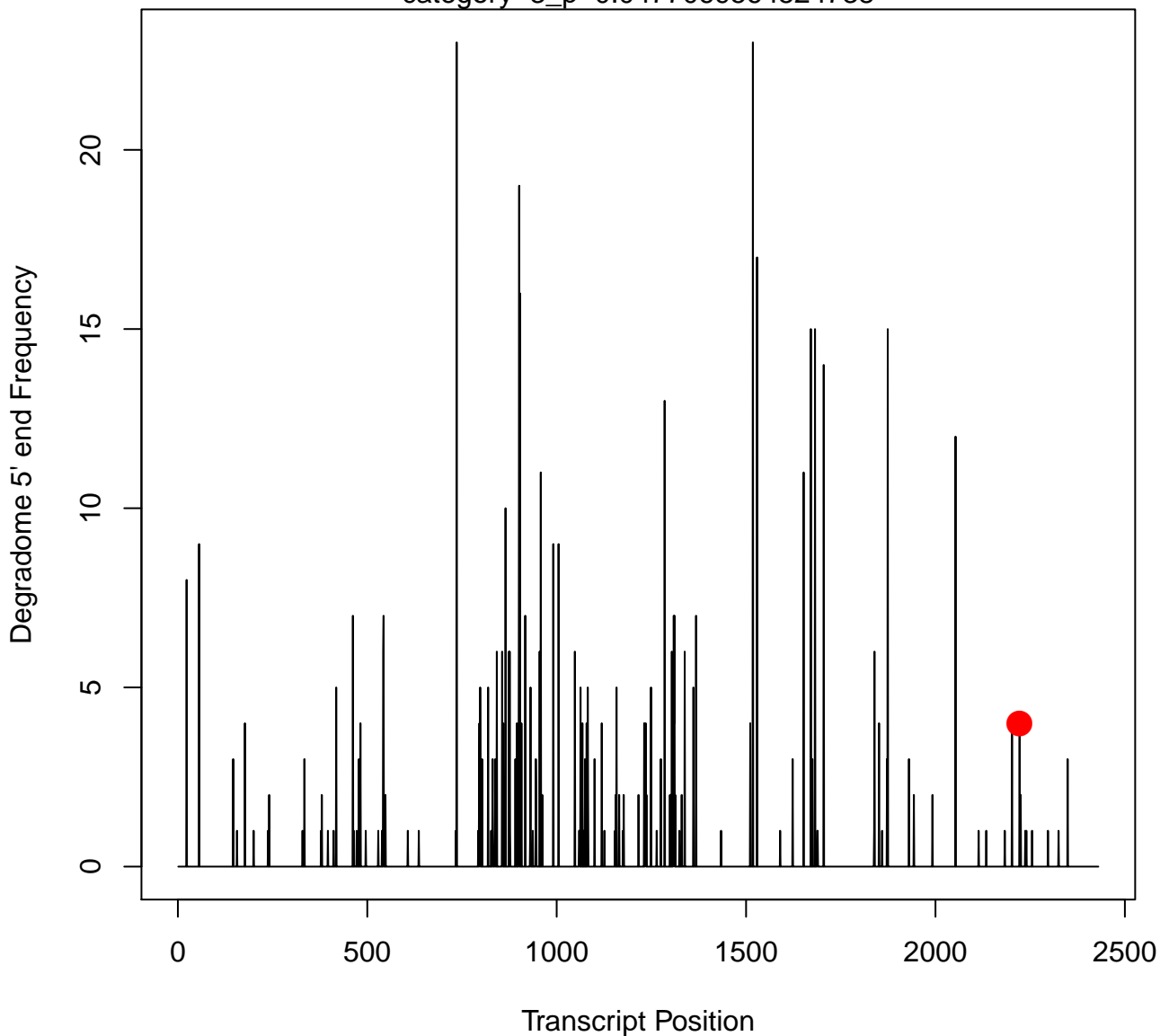

Supplement: Supplementary file 1 [file DataSheet_1.zip › The miRNA-target modules identified by the CleaveLand4/miR167a-5p_evm.model.LG01.7049_2222_TPlot.pdf]

**T=evm.model.LG02.237\_Q=miR167e-5p\_S=102**

category=1\_p=0.0205482959025826

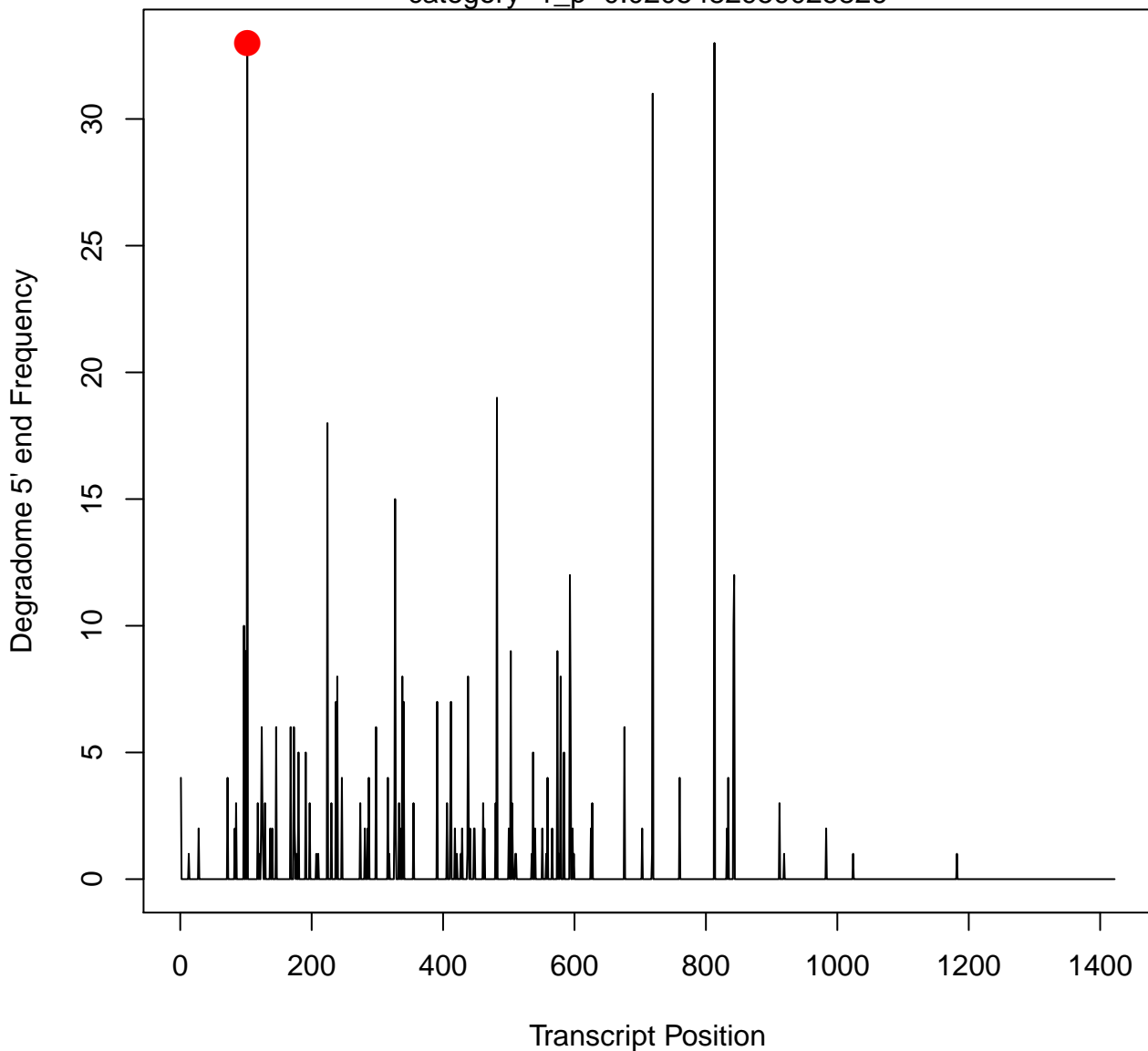

Supplement: Supplementary file 1 [file DataSheet_1.zip › The miRNA-target modules identified by the CleaveLand4/miR167e-5p_evm.model.LG02.237_102_TPlot.pdf]

**T=evm.model.LG05.2295\_Q=miR167e-5p\_S=1994**

category=2\_p=0.0260209548157995

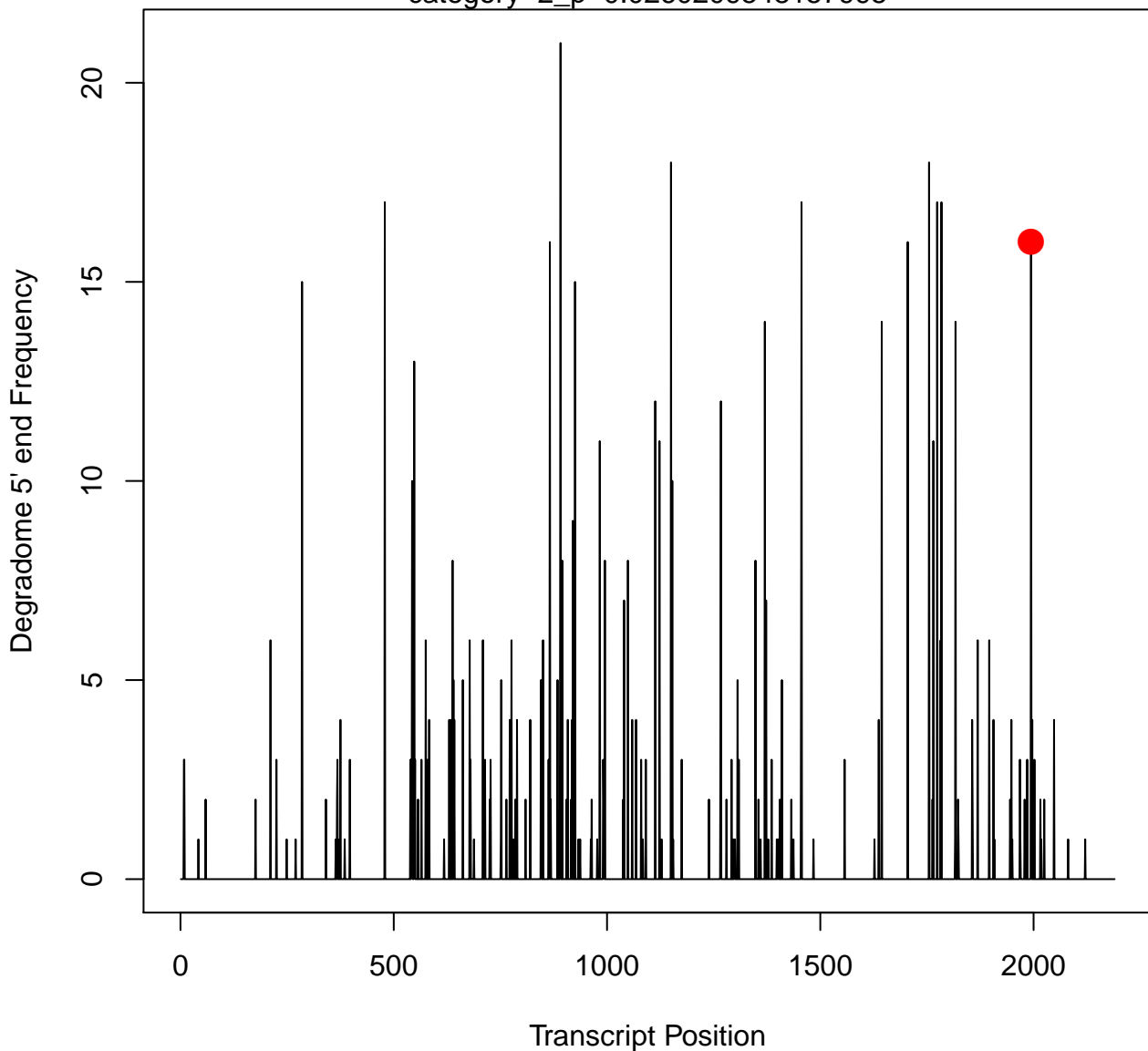

Supplement: Supplementary file 1 [file DataSheet_1.zip › The miRNA-target modules identified by the CleaveLand4/miR167e-5p_evm.model.LG05.2295_1994_TPlot.pdf]

**T=evm.model.LG06.3980\_Q=miR167e-5p\_S=2468**

category=3\_p=0.0241449679652586

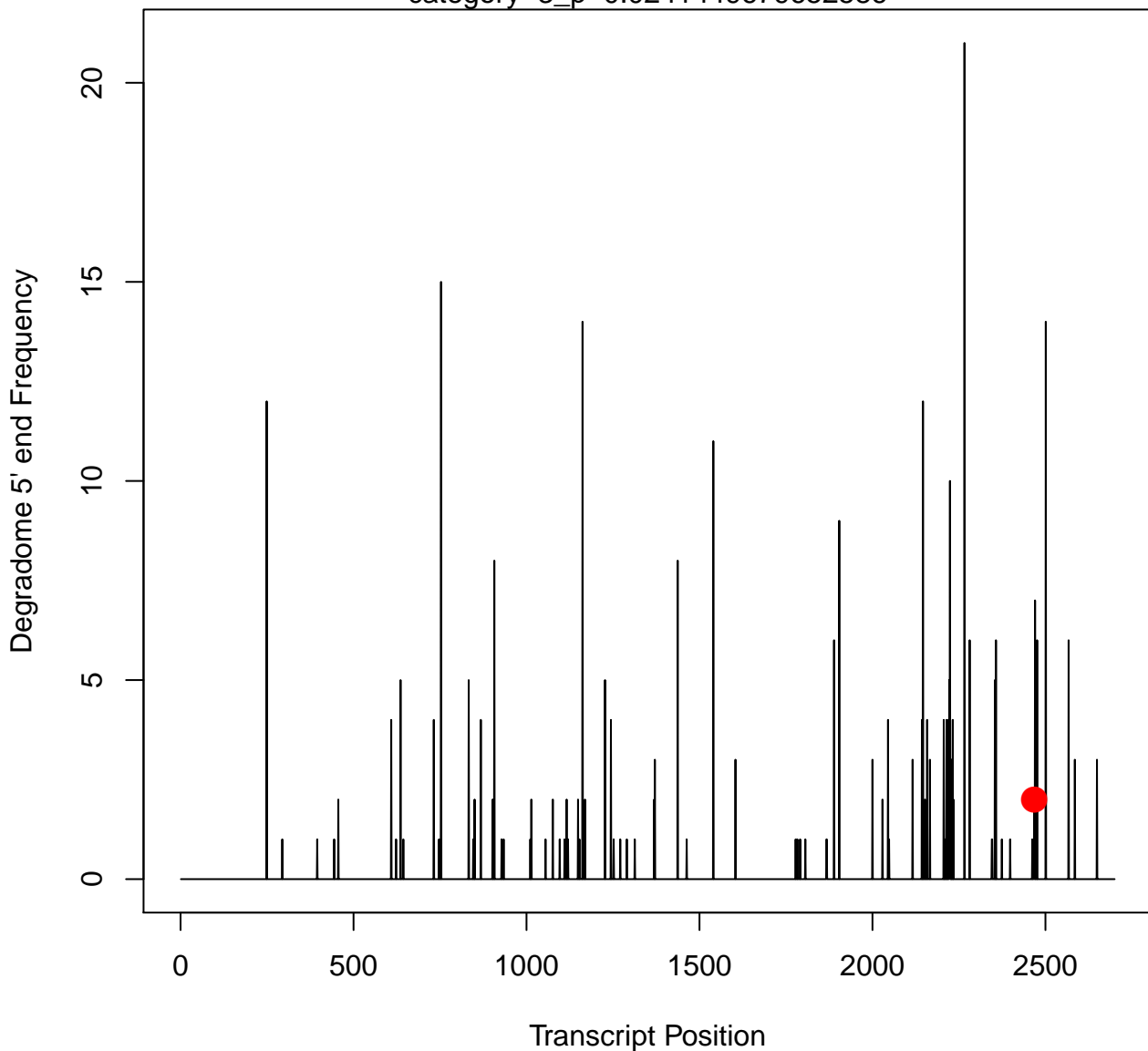

Supplement: Supplementary file 1 [file DataSheet_1.zip › The miRNA-target modules identified by the CleaveLand4/miR167e-5p_evm.model.LG06.3980_2468_TPlot.pdf]

**T=evm.model.LG04.3713\_Q=miR168a-5p\_S=424**

category=2\_p=0.00874999063480097

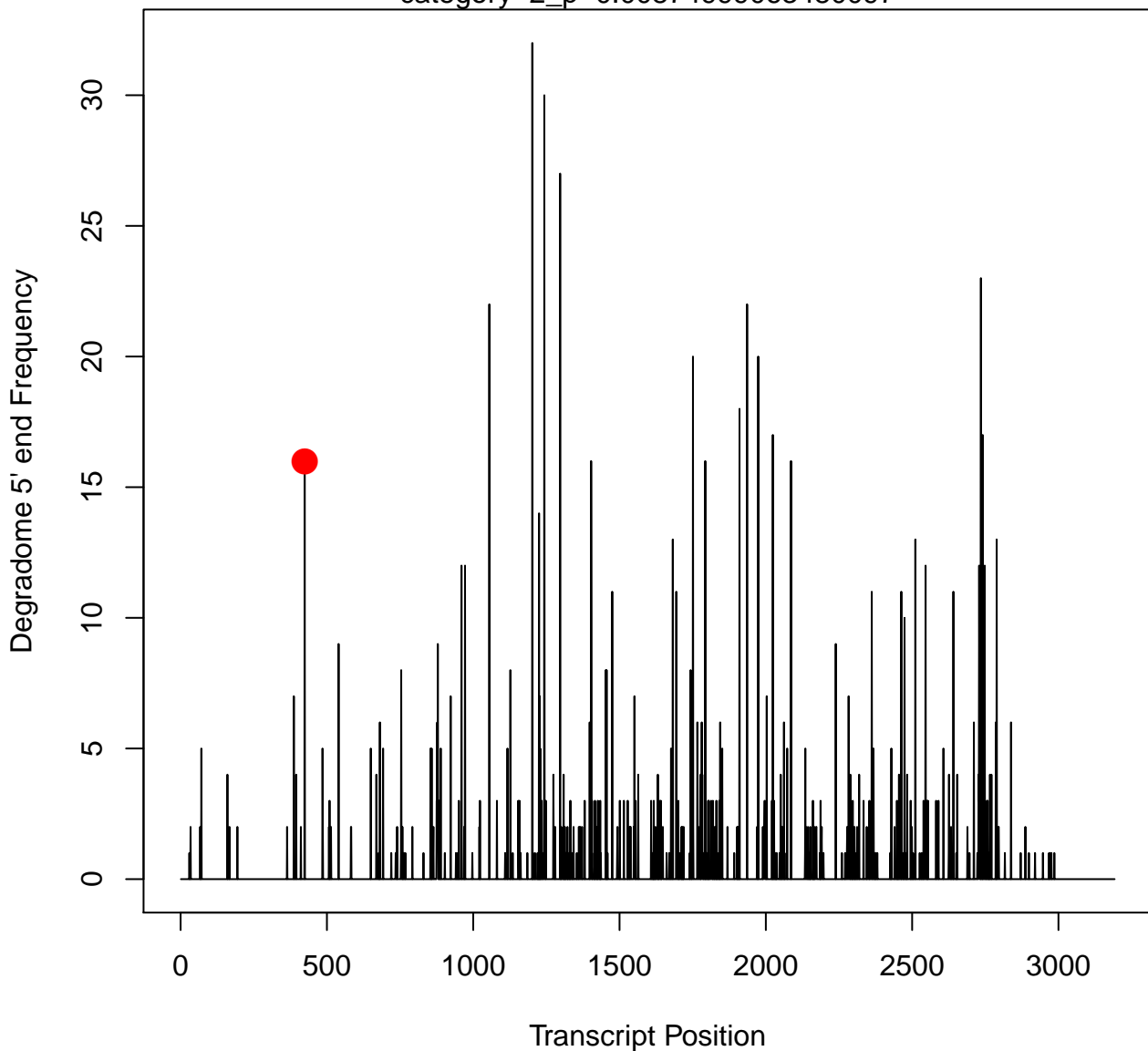

Supplement: Supplementary file 1 [file DataSheet_1.zip › The miRNA-target modules identified by the CleaveLand4/miR168a-5p_evm.model.LG04.3713_424_TPlot.pdf]

**T=evm.model.LG01.8136\_Q=miR168b-5p\_S=811**

category=2\_p=0.00874999063480097

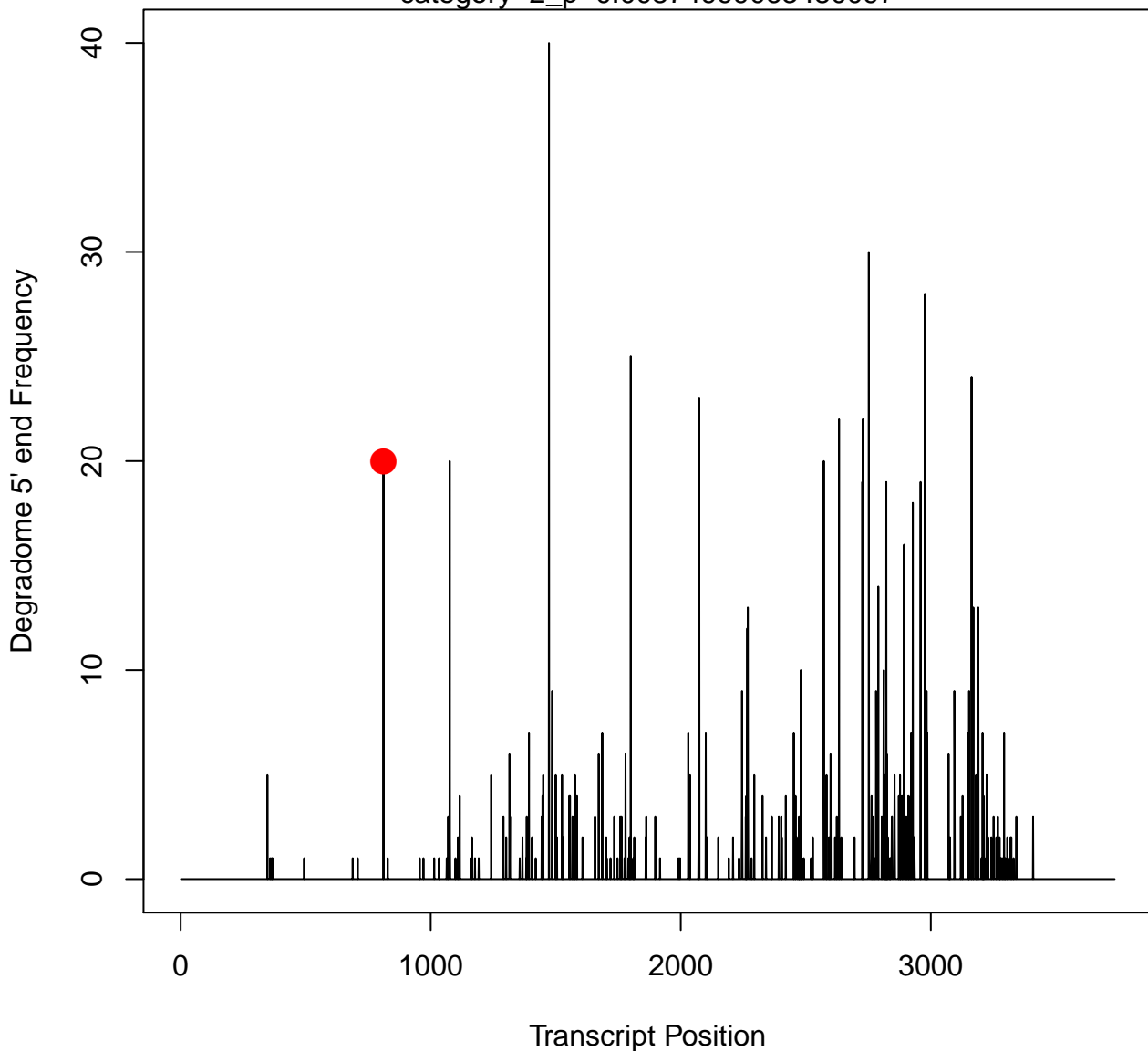

Supplement: Supplementary file 1 [file DataSheet_1.zip › The miRNA-target modules identified by the CleaveLand4/miR168b-5p_evm.model.LG01.8136_811_TPlot.pdf]

**T=evm.model.LG06.170\_Q=miR169b-3p\_S=466**

category=2\_p=0.0345432623396535

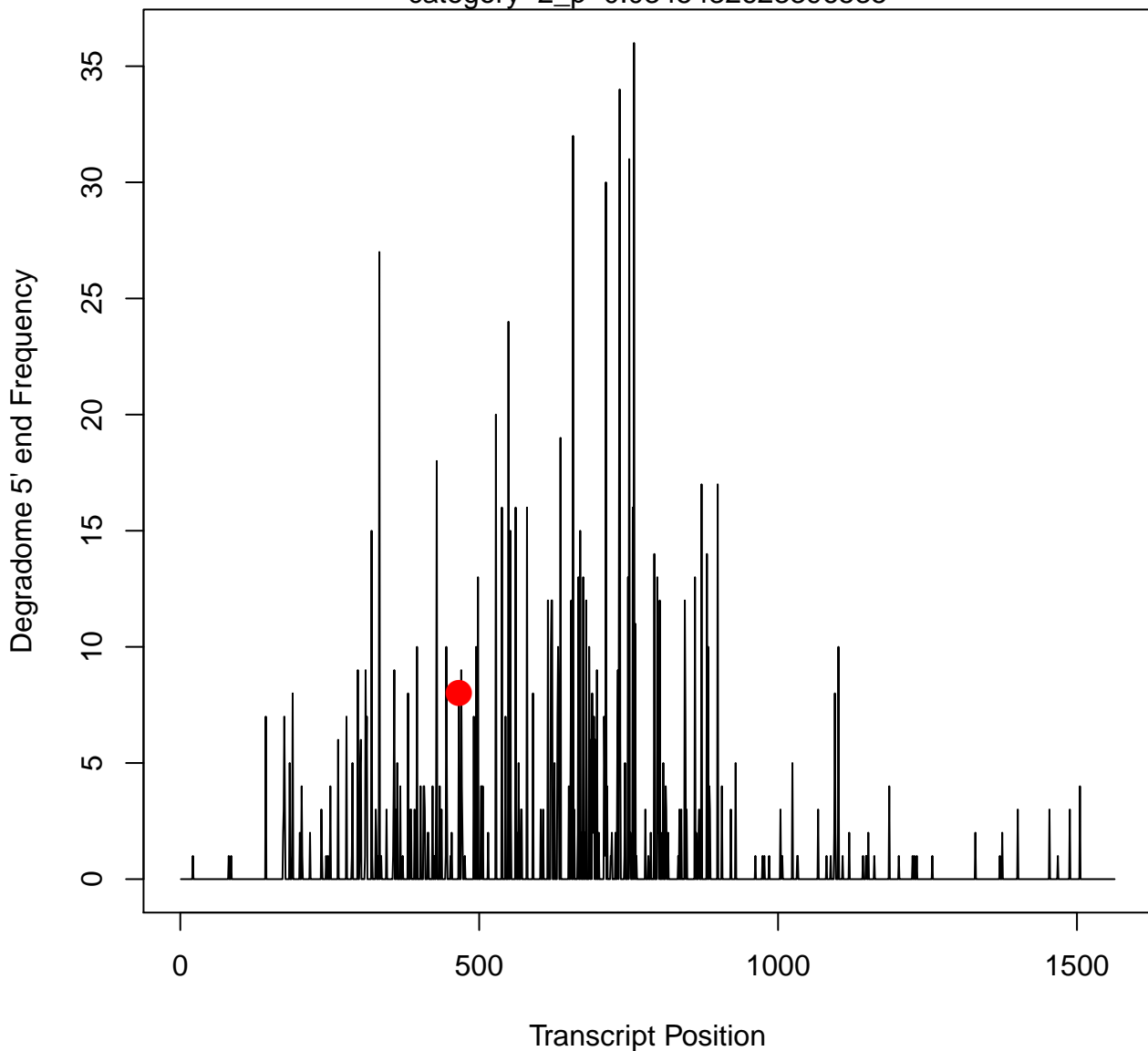

Supplement: Supplementary file 1 [file DataSheet_1.zip › The miRNA-target modules identified by the CleaveLand4/miR169b-3p_evm.model.LG06.170_466_TPlot.pdf]

**T=evm.model.LG02.5698\_Q=miR171a-3p\_S=1061**

category=0\_p=0.00051912393111464

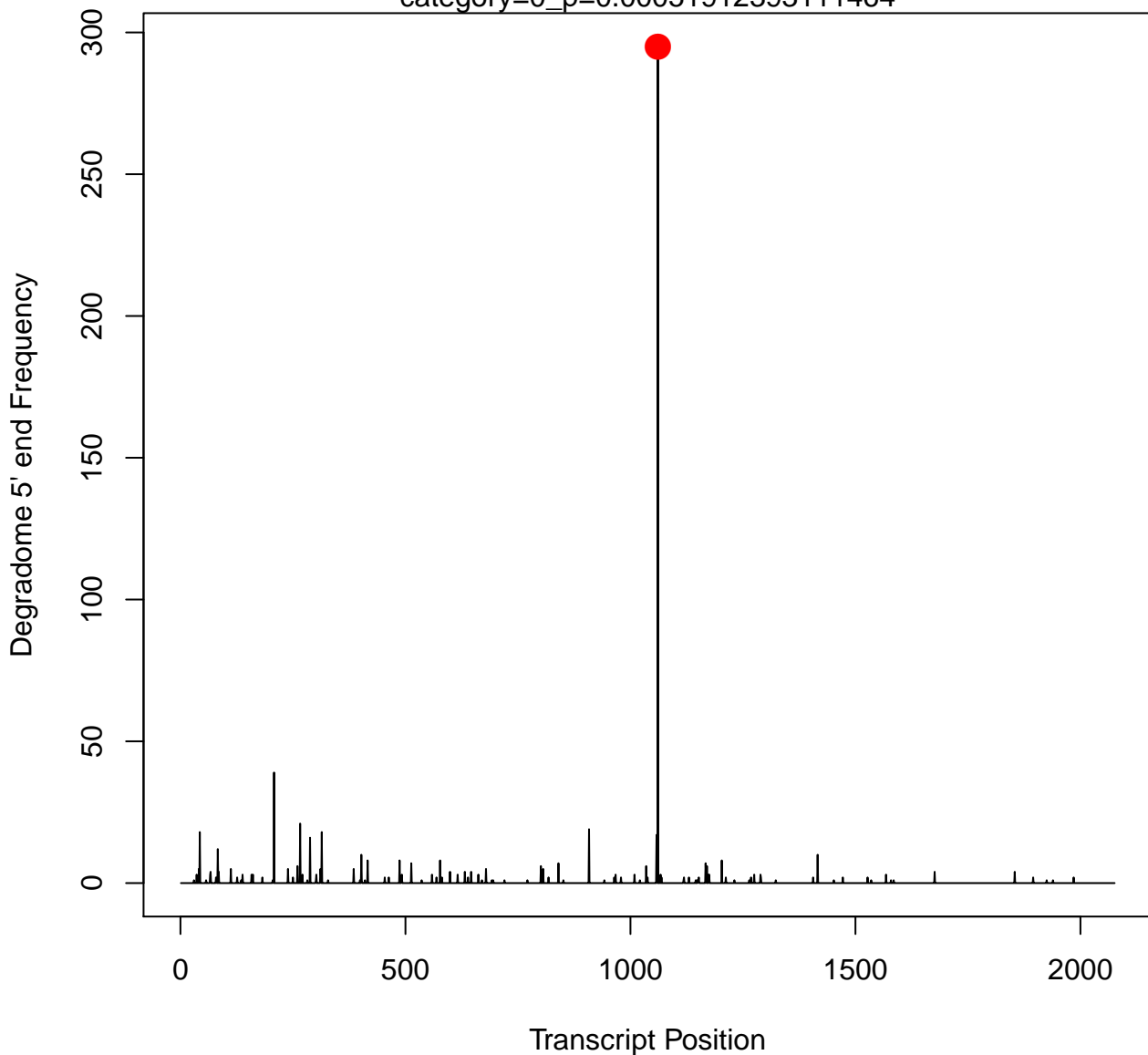

Supplement: Supplementary file 1 [file DataSheet_1.zip › The miRNA-target modules identified by the CleaveLand4/miR171a-3p_evm.model.LG02.5698_1061_TPlot.pdf]

**T=evm.model.LG03.1086\_Q=miR171a-3p\_S=491**

category=0\_p=0.00207487934604456

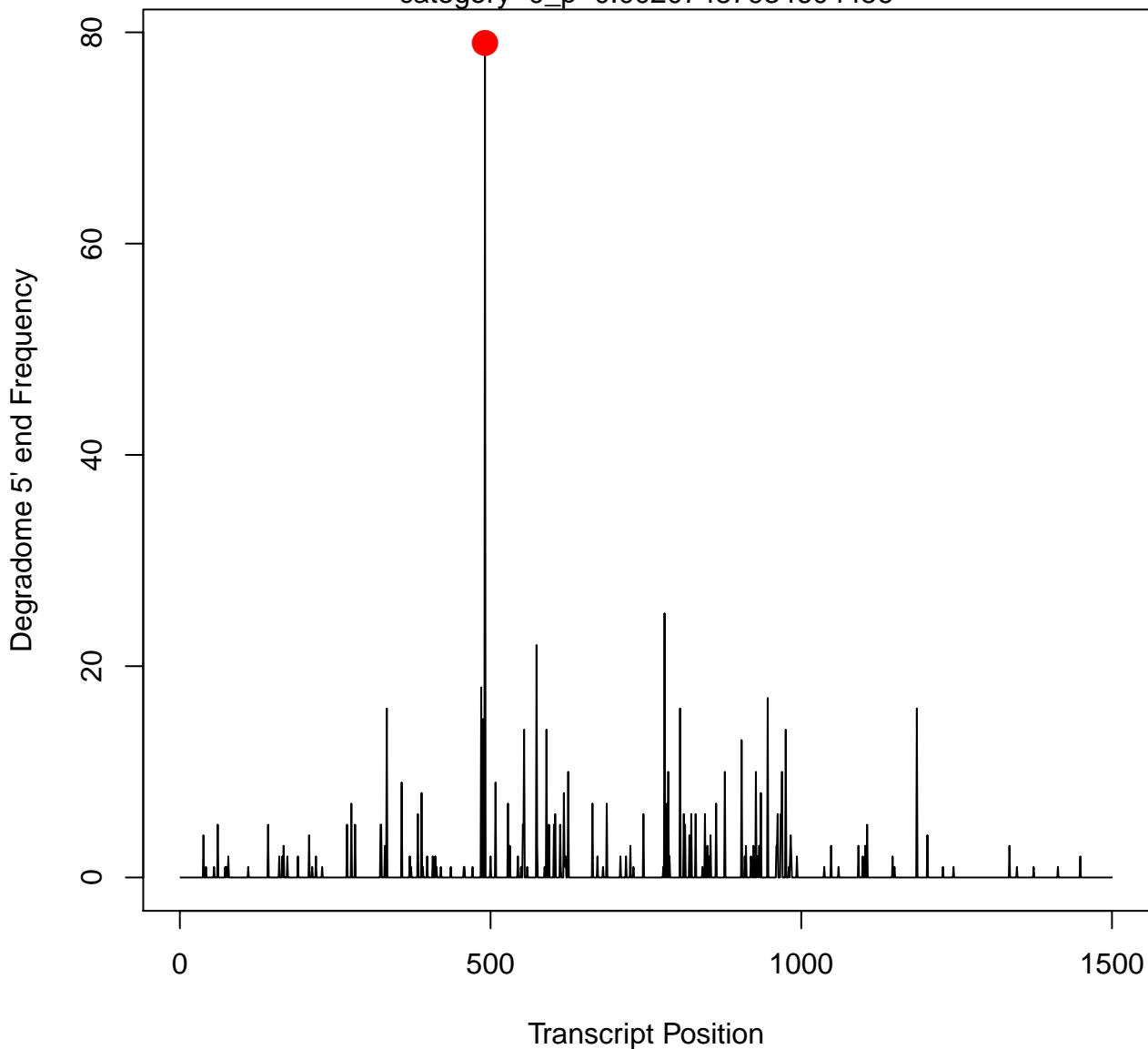

Supplement: Supplementary file 1 [file DataSheet_1.zip › The miRNA-target modules identified by the CleaveLand4/miR171a-3p_evm.model.LG03.1086_491_TPlot.pdf]

**T=evm.model.LG05.2085\_Q=miR171a-3p\_S=551**

category=0\_p=0.00155656346427469

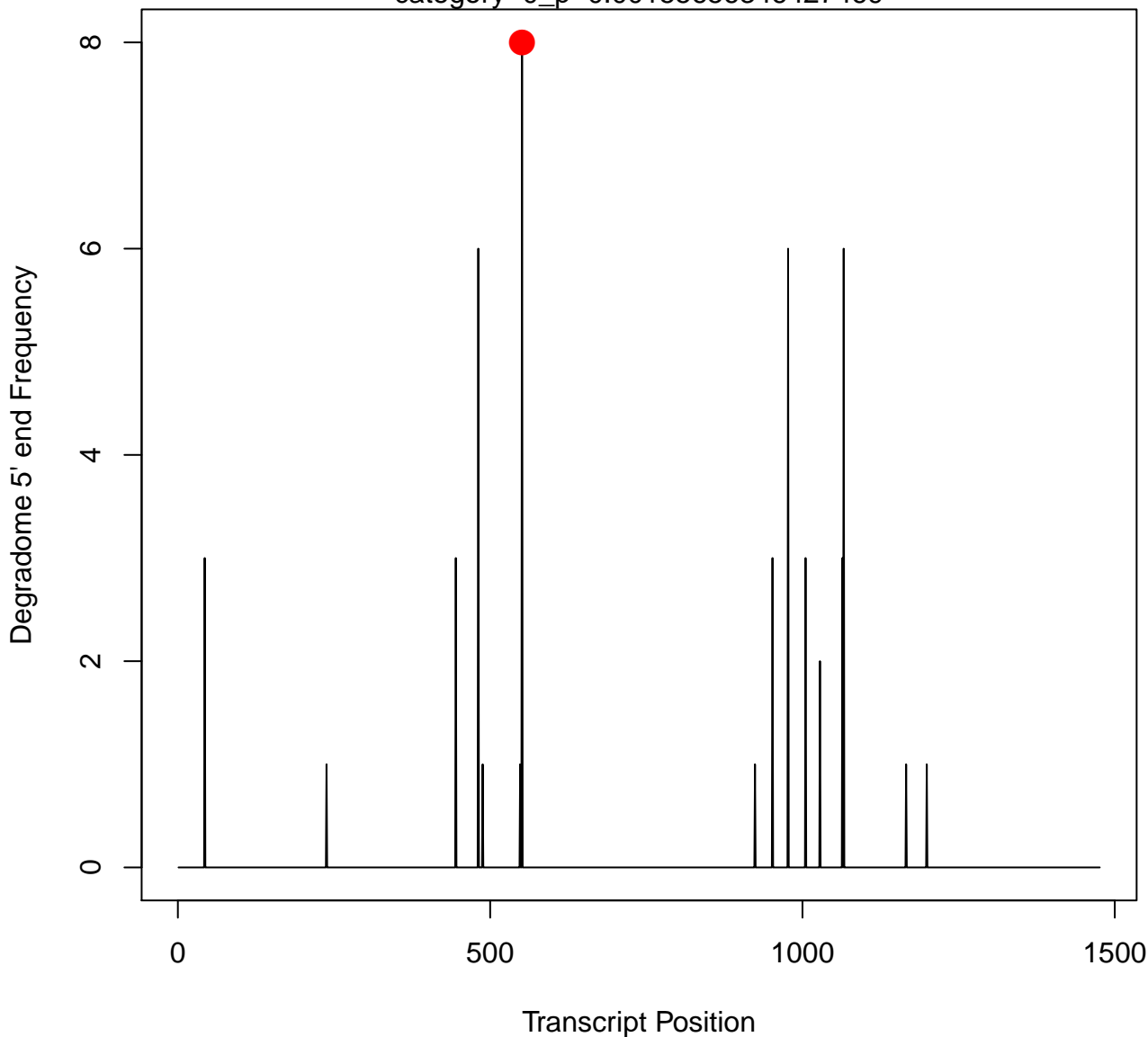

Supplement: Supplementary file 1 [file DataSheet_1.zip › The miRNA-target modules identified by the CleaveLand4/miR171a-3p_evm.model.LG05.2085_551_TPlot.pdf]

**T=evm.model.LG06.4588\_Q=miR171a-3p\_S=998**

category=0\_p=0.00103797837257324

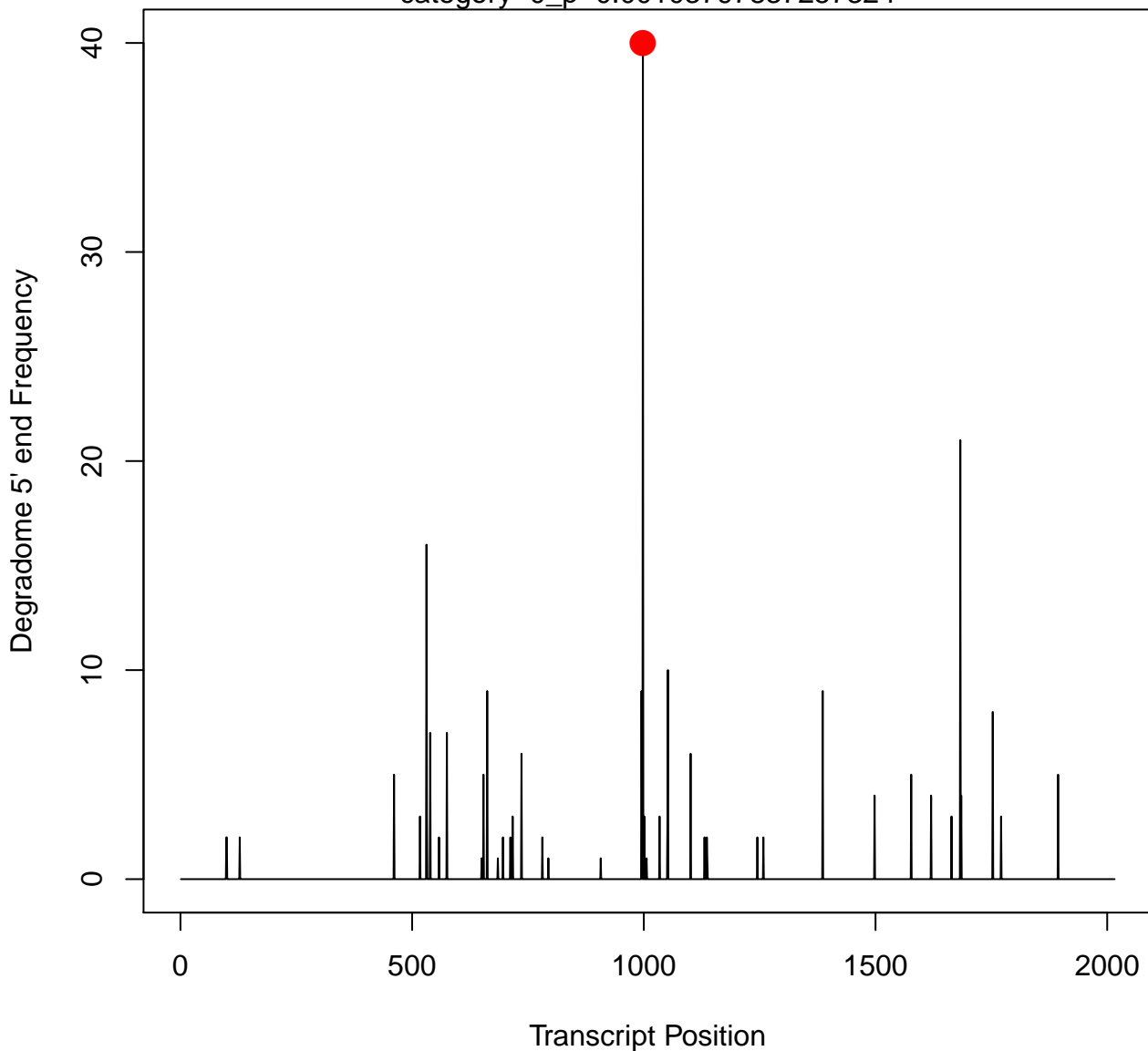

Supplement: Supplementary file 1 [file DataSheet_1.zip › The miRNA-target modules identified by the CleaveLand4/miR171a-3p_evm.model.LG06.4588_998_TPlot.pdf]

**T=evm.model.LG02.5698\_Q=miR171c-3p\_S=1058**

category=2\_p=0.0260209548157995

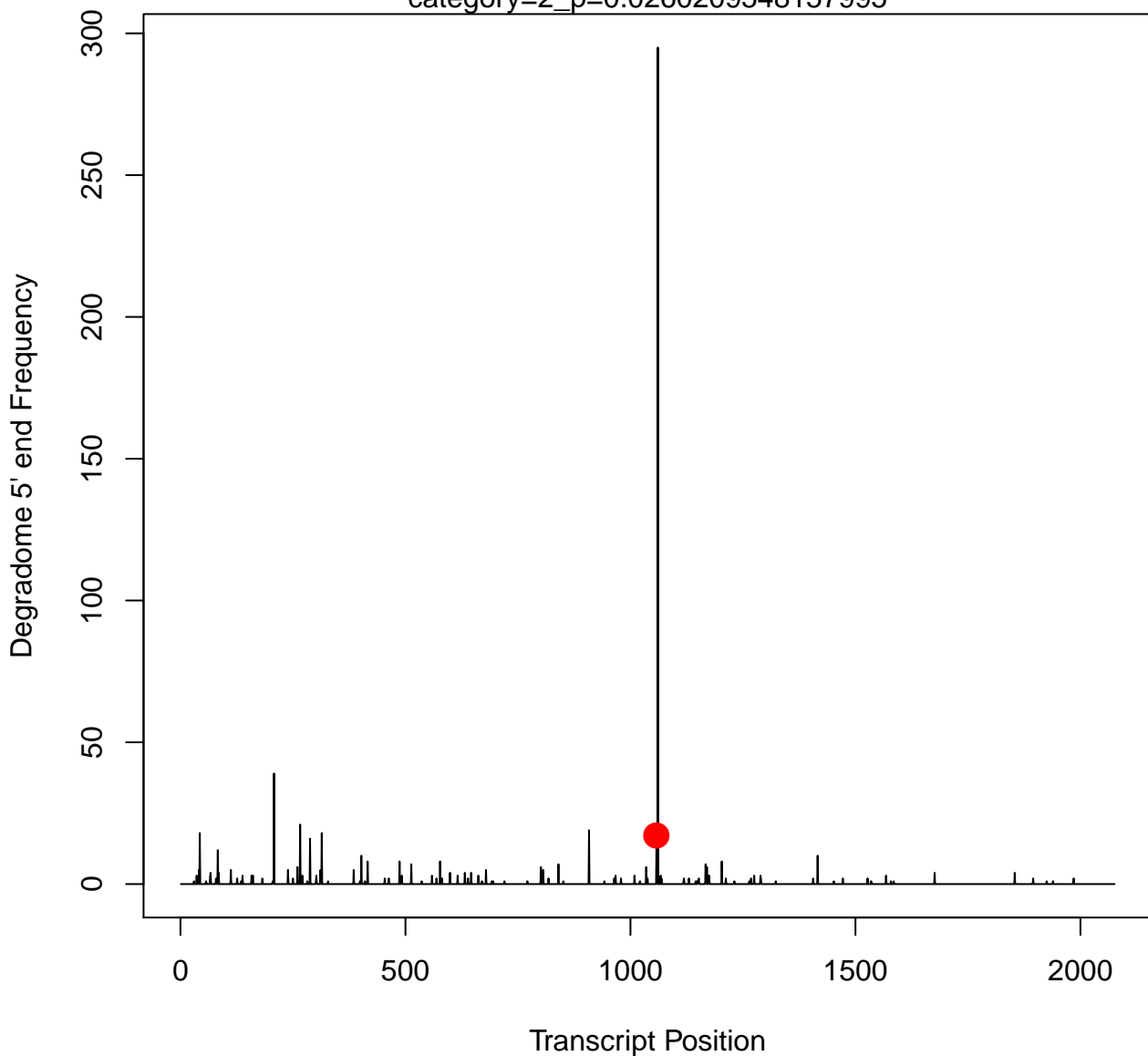

Supplement: Supplementary file 1 [file DataSheet_1.zip › The miRNA-target modules identified by the CleaveLand4/miR171c-3p_evm.model.LG02.5698_1058_TPlot.pdf]

**T=evm.model.LG03.1086\_Q=miR171c-3p\_S=488**

category=2\_p=0.0174234189334928

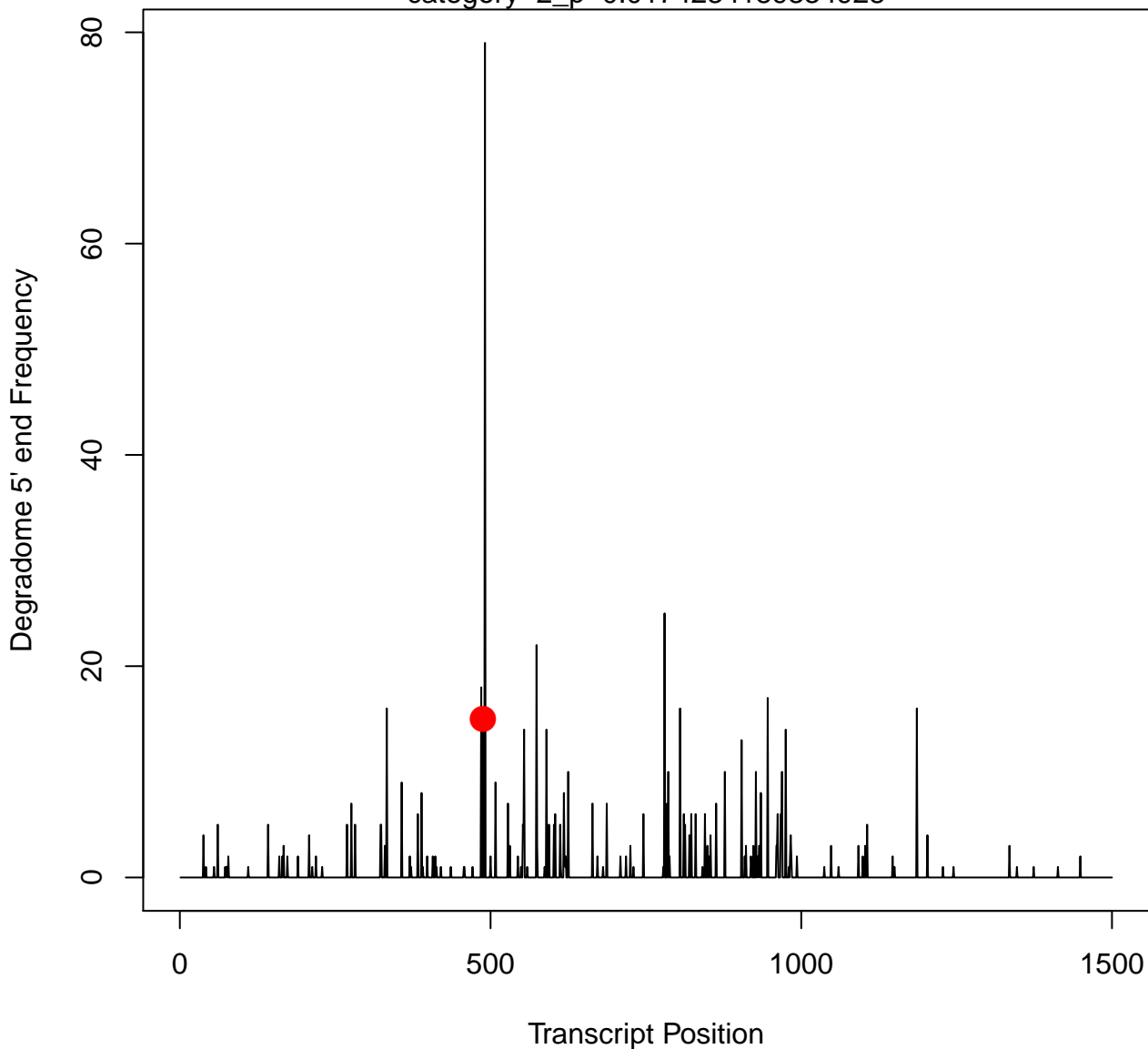

Supplement: Supplementary file 1 [file DataSheet_1.zip › The miRNA-target modules identified by the CleaveLand4/miR171c-3p_evm.model.LG03.1086_488_TPlot.pdf]

**T=evm.model.LG05.2085\_Q=miR171c-3p\_S=548**

category=4\_p=0.00862274555333054

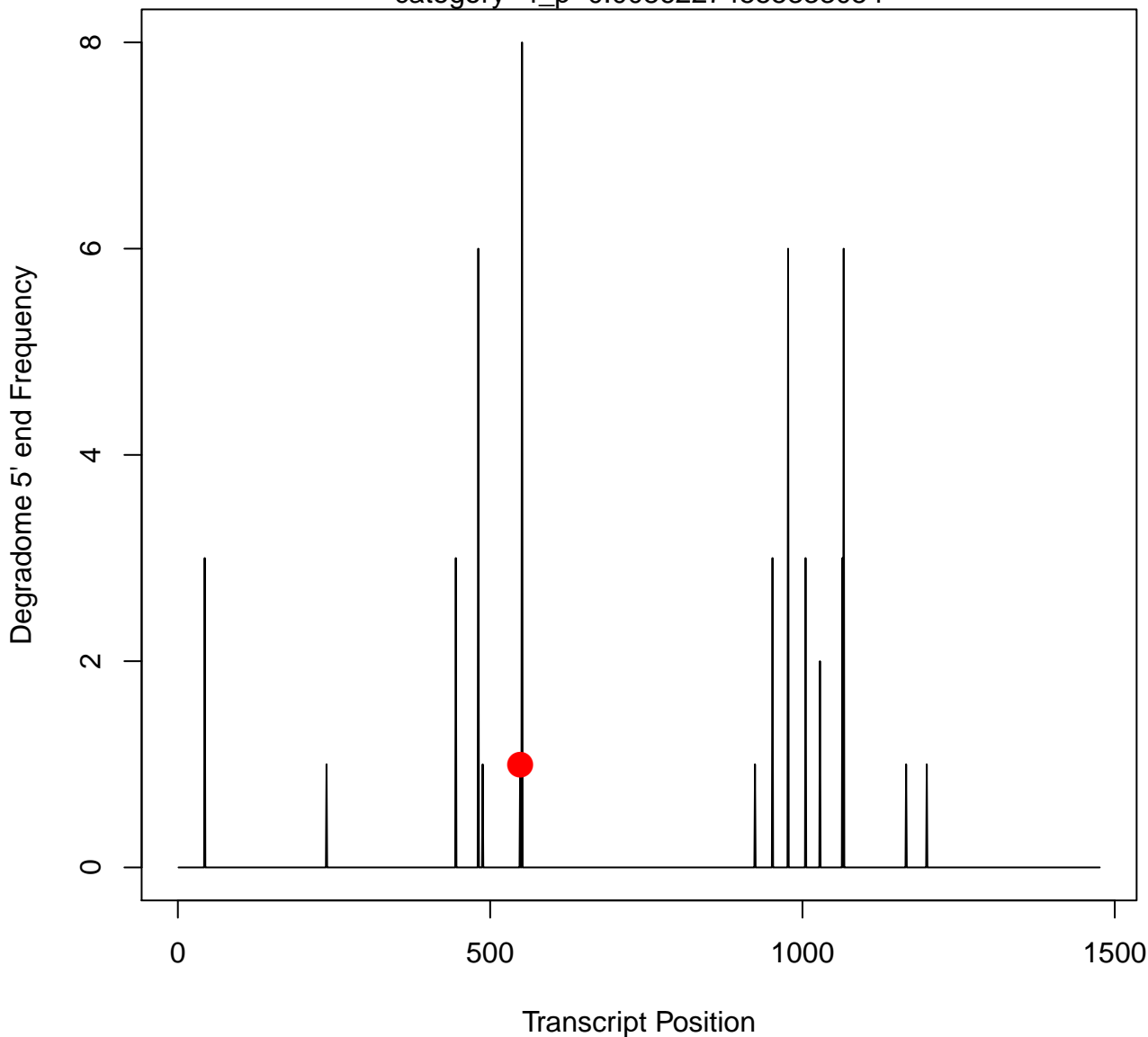

Supplement: Supplementary file 1 [file DataSheet_1.zip › The miRNA-target modules identified by the CleaveLand4/miR171c-3p_evm.model.LG05.2085_548_TPlot.pdf]

**T=evm.model.LG06.4588\_Q=miR171c-3p\_S=995**

category=2\_p=0.0345432623396535

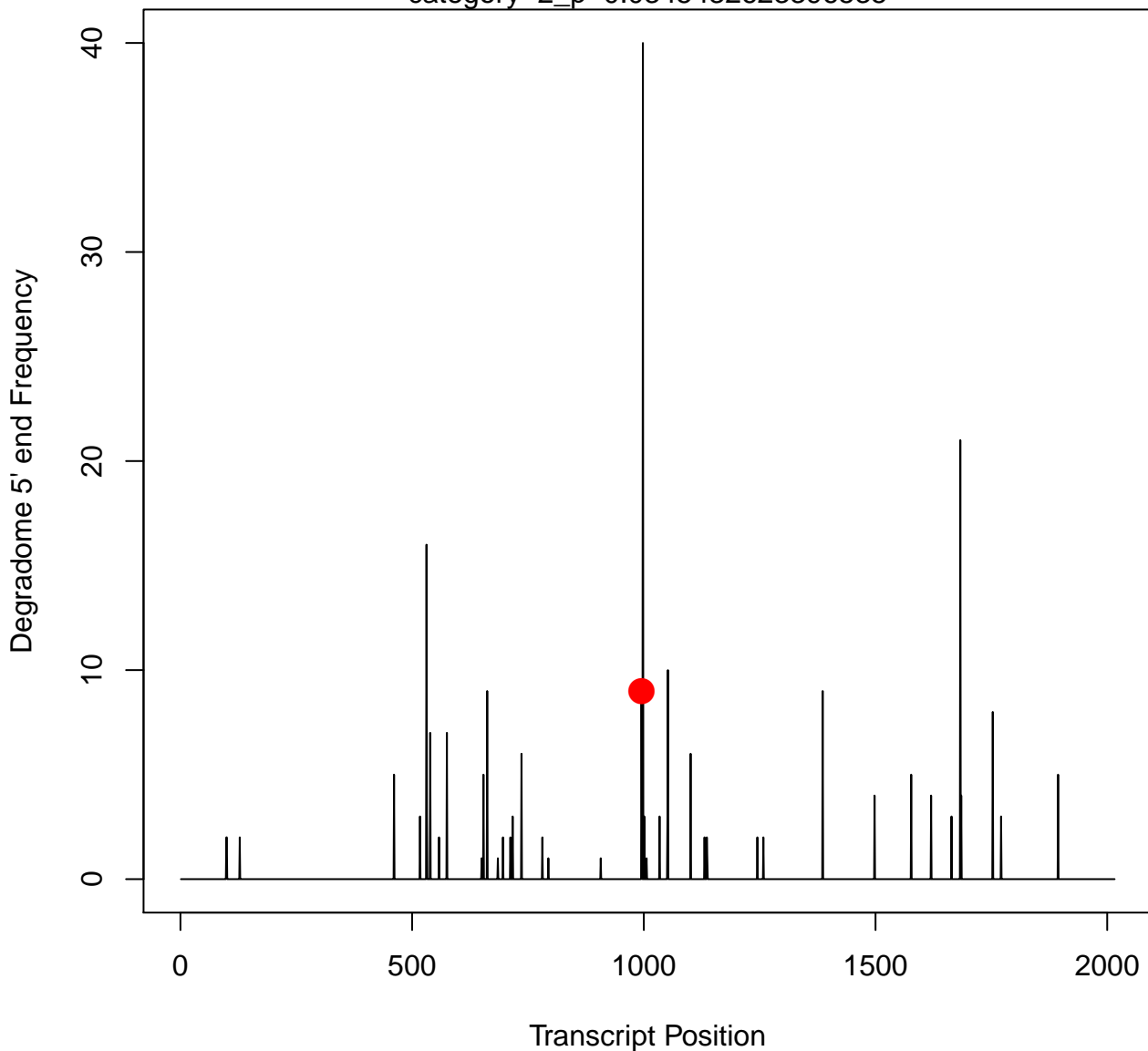

Supplement: Supplementary file 1 [file DataSheet_1.zip › The miRNA-target modules identified by the CleaveLand4/miR171c-3p_evm.model.LG06.4588_995_TPlot.pdf]

**T=evm.model.LG05.2085\_Q=miR171d-3p\_S=551**

category=0\_p=0.00051912393111464

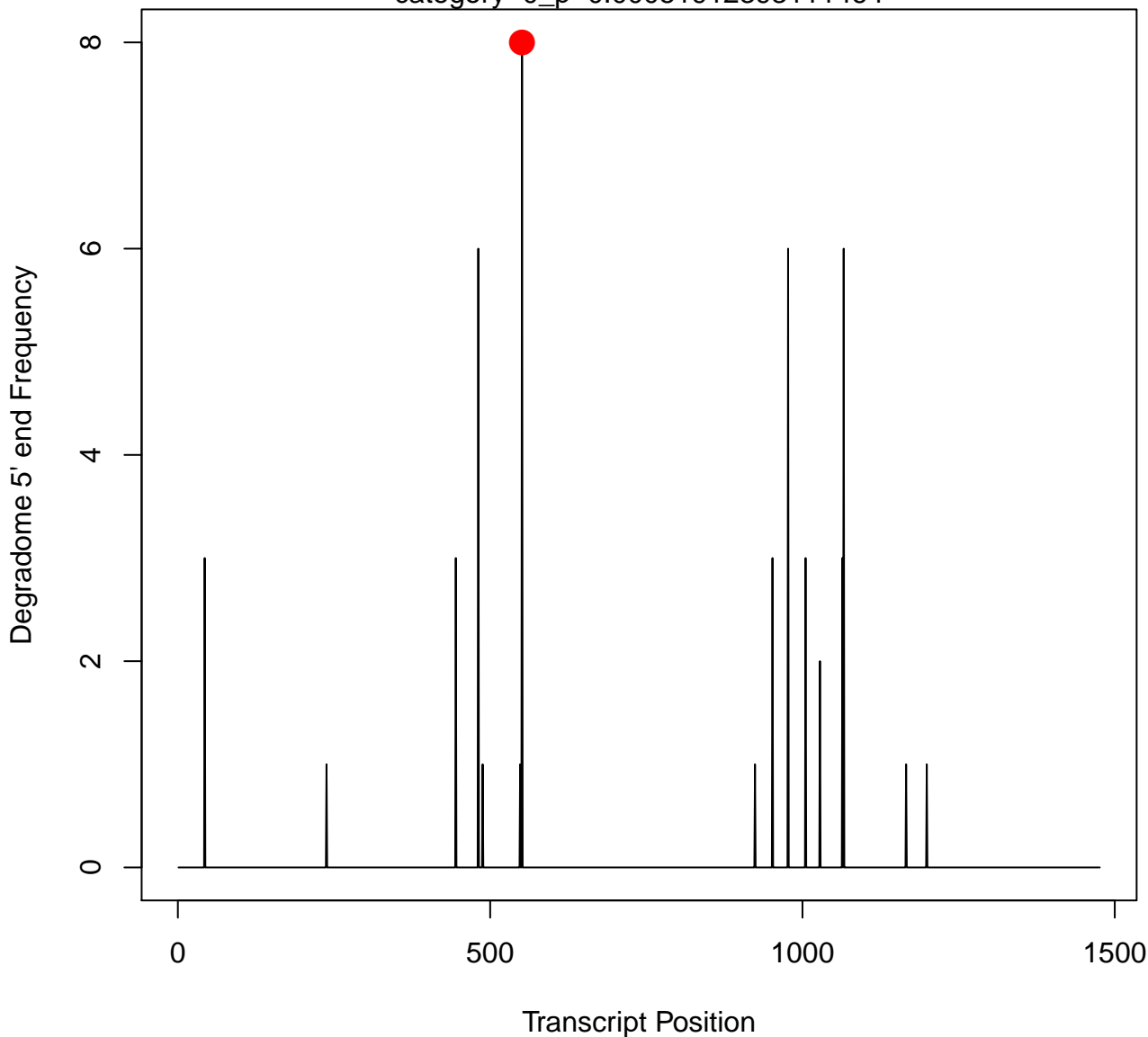

Supplement: Supplementary file 1 [file DataSheet_1.zip › The miRNA-target modules identified by the CleaveLand4/miR171d-3p_evm.model.LG05.2085_551_TPlot.pdf]

**T=evm.model.LG02.5698\_Q=miR171f-3p\_S=1058**

category=2\_p=0.0260209548157995

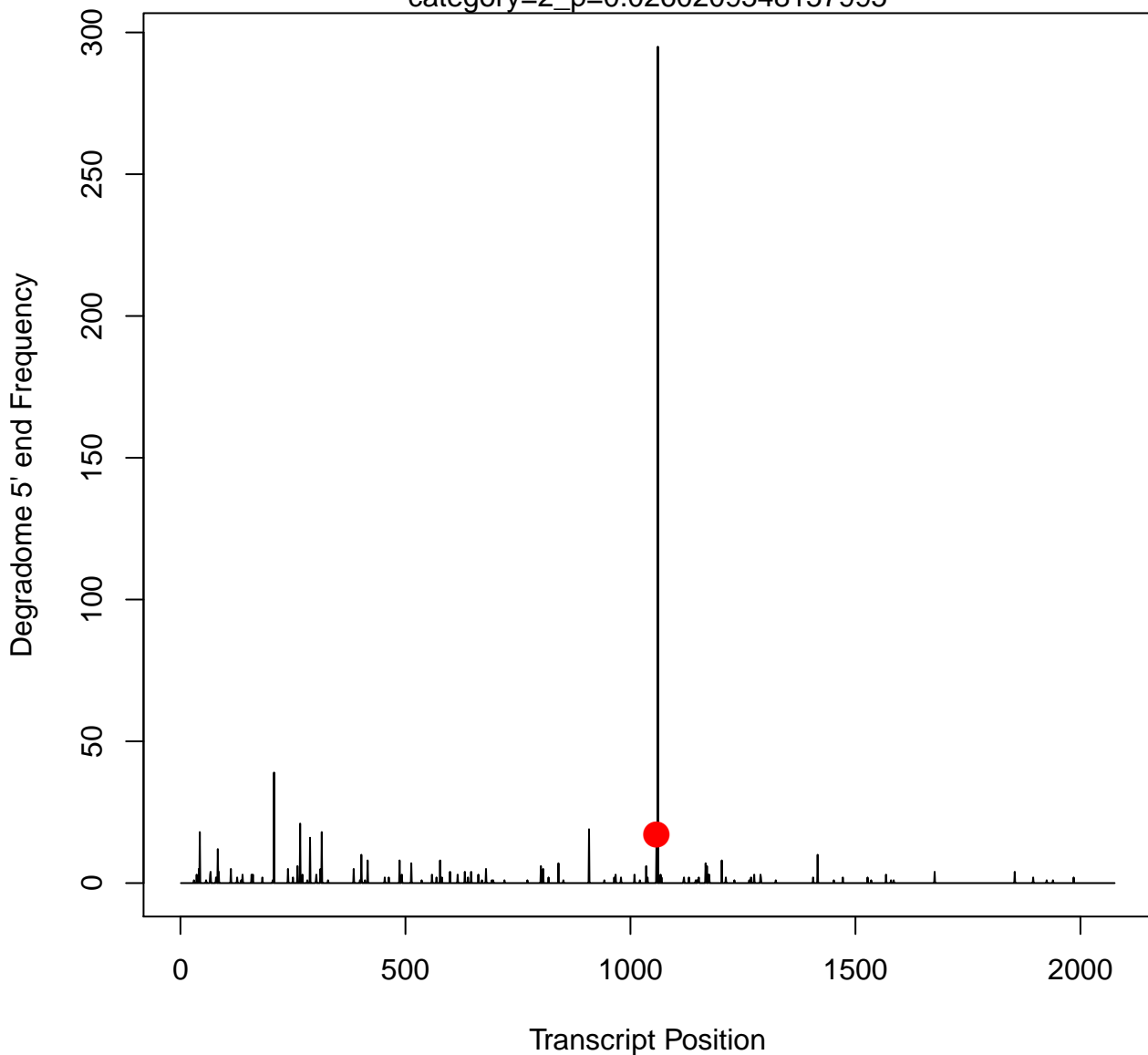

Supplement: Supplementary file 1 [file DataSheet_1.zip › The miRNA-target modules identified by the CleaveLand4/miR171f-3p_evm.model.LG02.5698_1058_TPlot.pdf]

**T=evm.model.LG03.1086\_Q=miR171h-3p\_S=488**

category=2\_p=0.0174234189334928

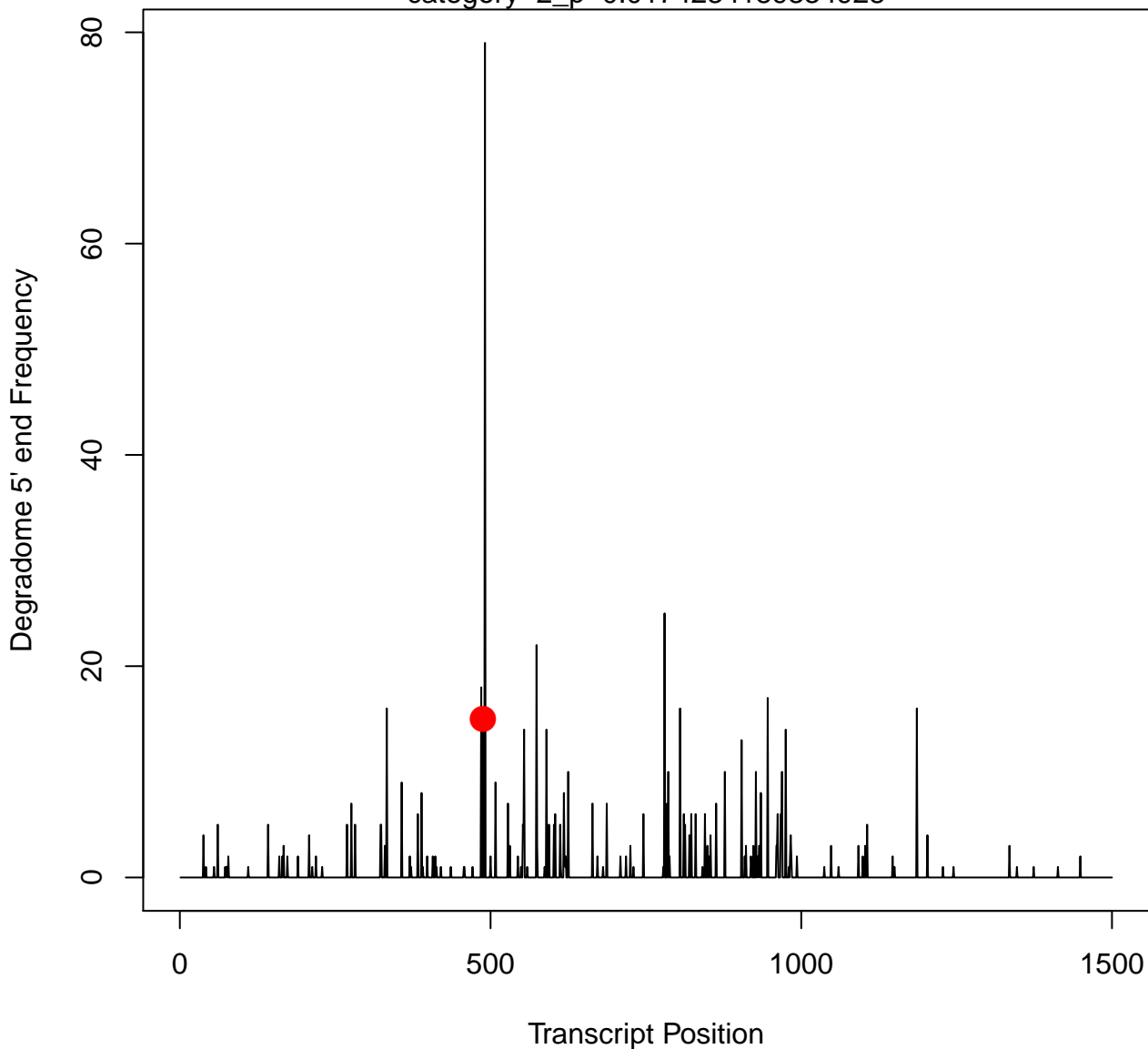

Supplement: Supplementary file 1 [file DataSheet_1.zip › The miRNA-target modules identified by the CleaveLand4/miR171h-3p_evm.model.LG03.1086_488_TPlot.pdf]

**T=evm.model.LG05.2085\_Q=miR171h-3p\_S=548**

category=4\_p=0.00862274555333054

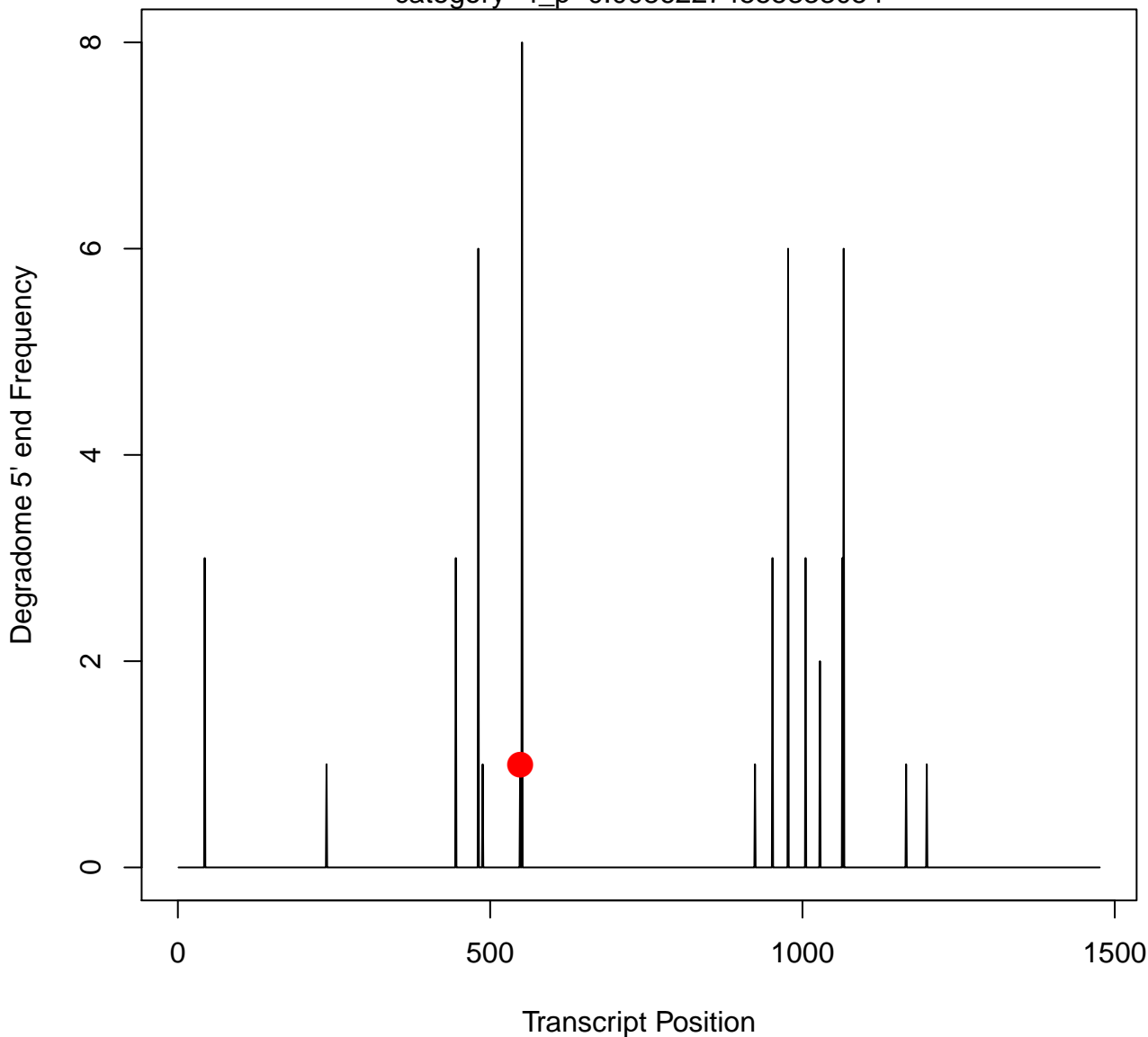

Supplement: Supplementary file 1 [file DataSheet_1.zip › The miRNA-target modules identified by the CleaveLand4/miR171h-3p_evm.model.LG05.2085_548_TPlot.pdf]

**T=evm.model.LG06.4588\_Q=miR171h-3p\_S=995**

category=2\_p=0.0260209548157995

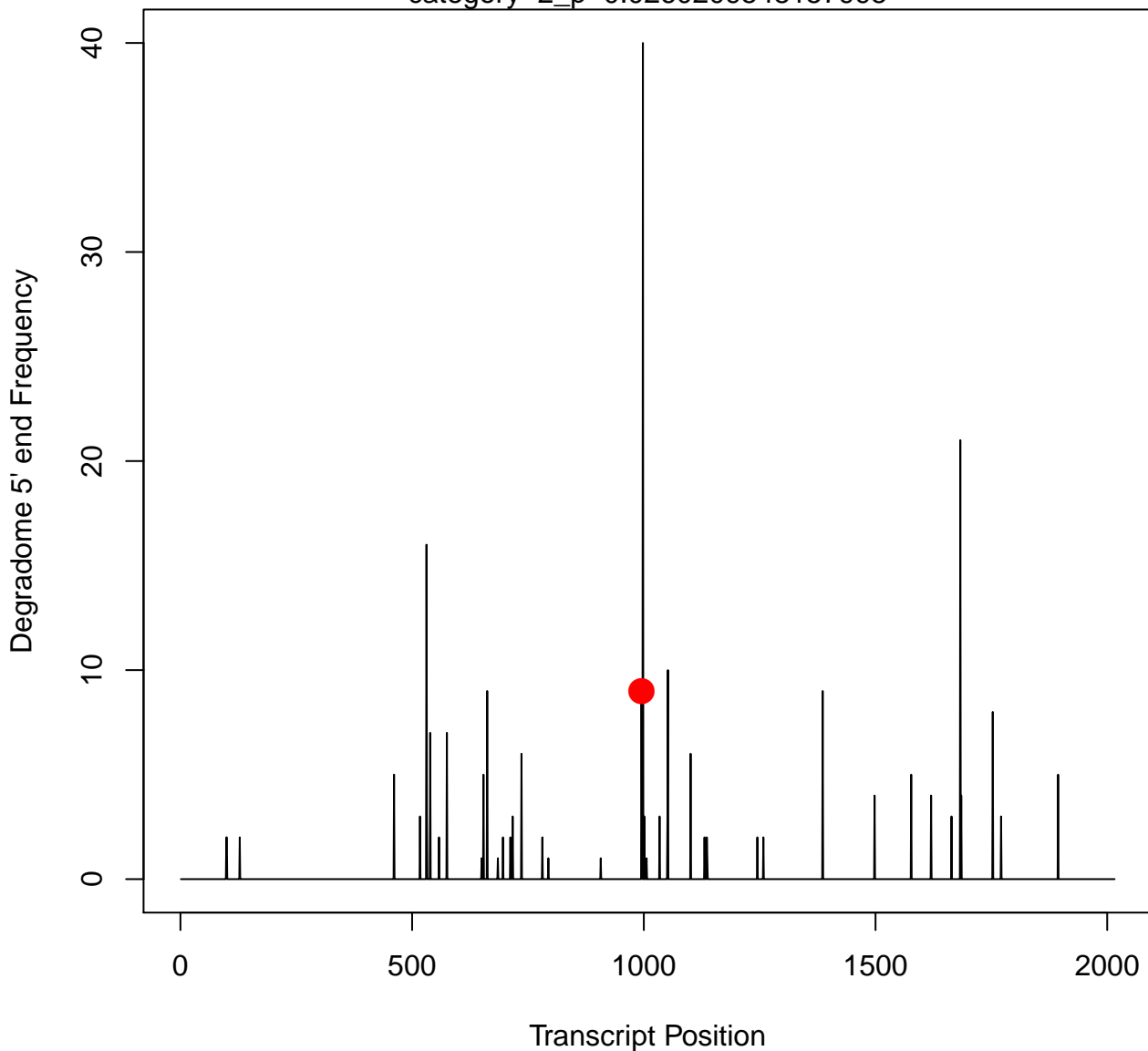

Supplement: Supplementary file 1 [file DataSheet_1.zip › The miRNA-target modules identified by the CleaveLand4/miR171h-3p_evm.model.LG06.4588_995_TPlot.pdf]

**T=evm.model.LG03.1086\_Q=miR171i-3p\_S=491**

category=0\_p=0.00207487934604456

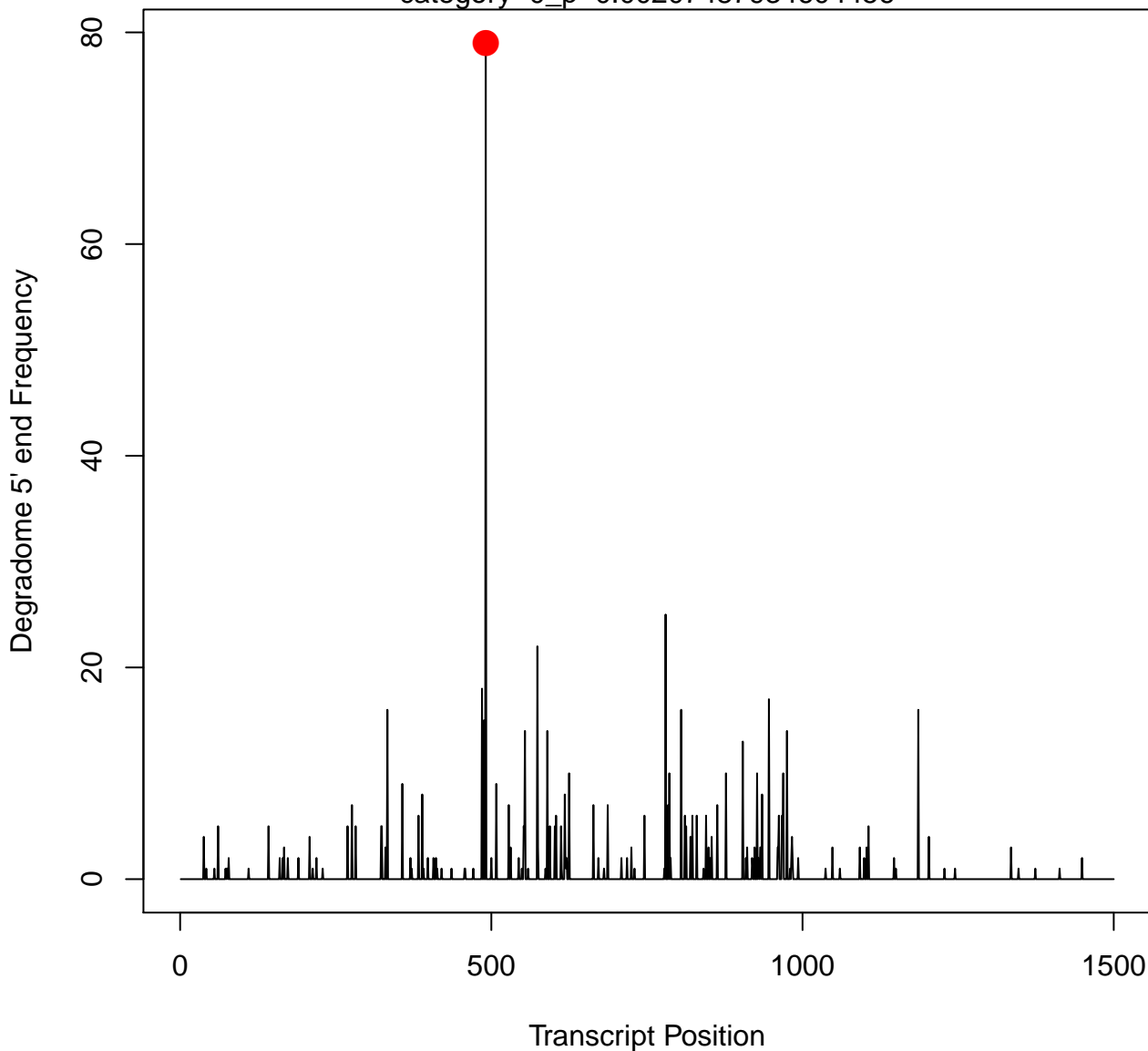

Supplement: Supplementary file 1 [file DataSheet_1.zip › The miRNA-target modules identified by the CleaveLand4/miR171i-3p_evm.model.LG03.1086_491_TPlot.pdf]

**T=evm.model.LG01.5728\_Q=miR172a-3p\_S=1243**

category=2\_p=0.00874999063480097

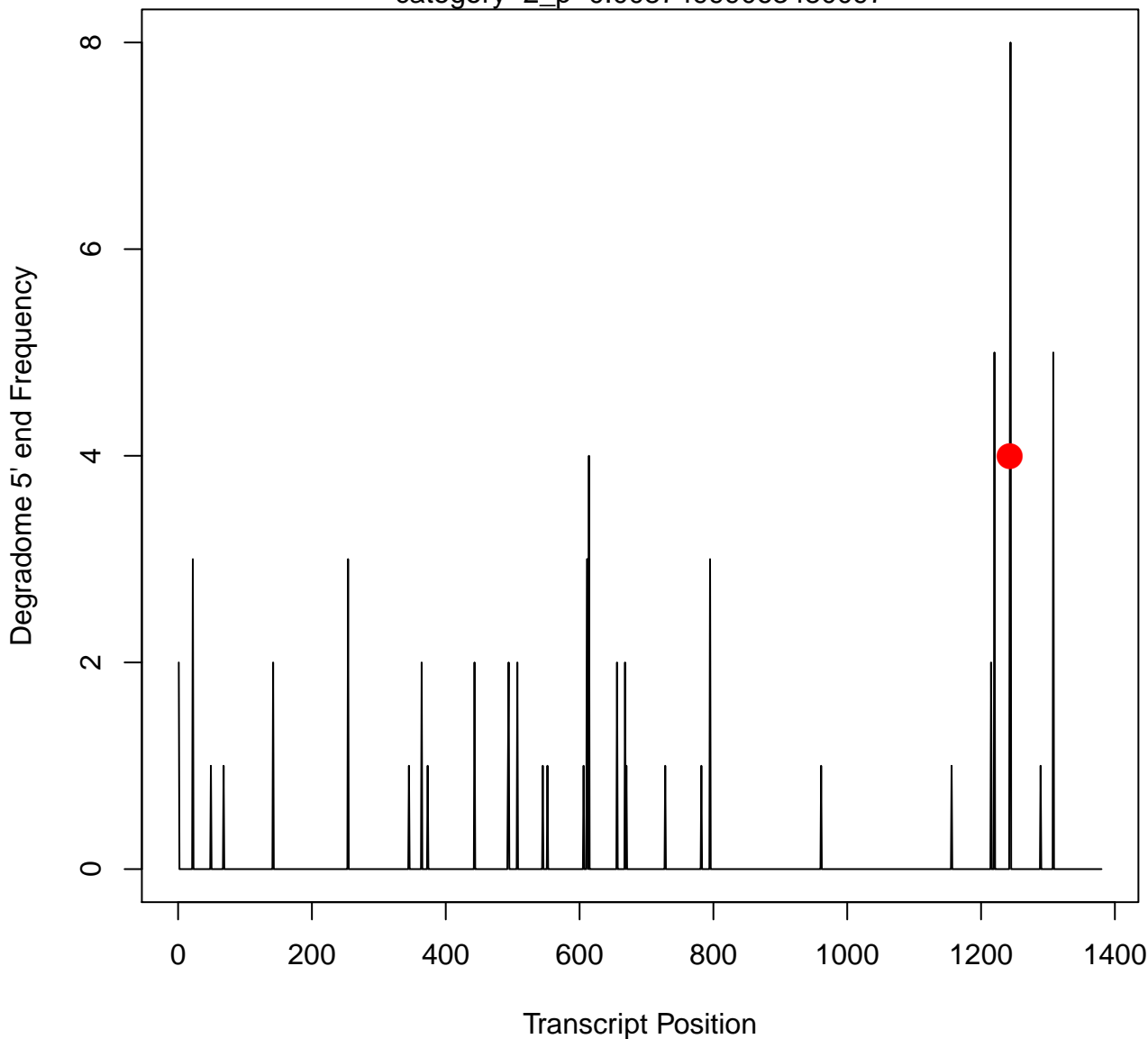

Supplement: Supplementary file 1 [file DataSheet_1.zip › The miRNA-target modules identified by the CleaveLand4/miR172a-3p_evm.model.LG01.5728_1243_TPlot.pdf]

**T=evm.model.LG01.6312\_Q=miR2111-5p\_S=128**

category=4\_p=0.00862274555333054

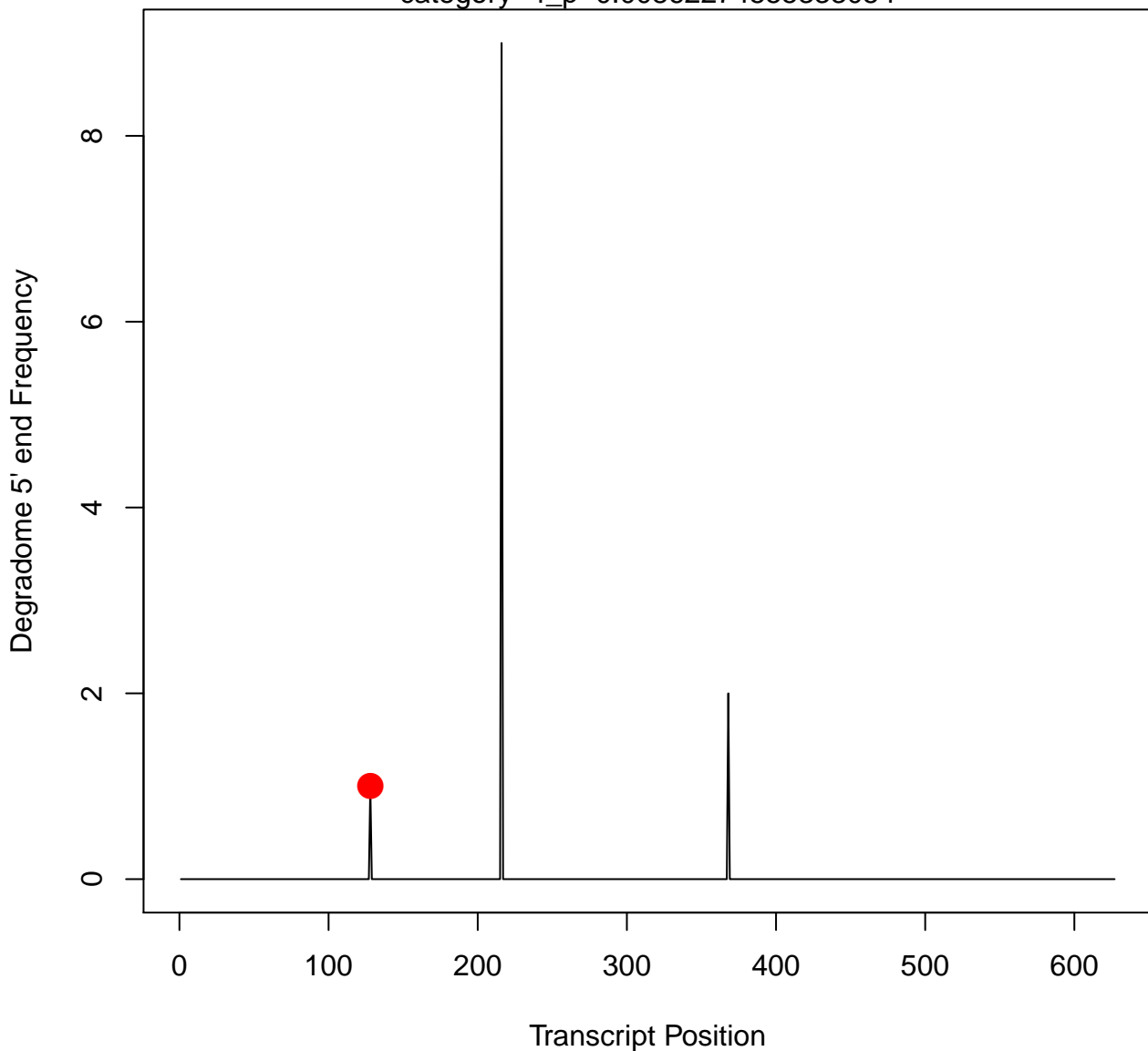

Supplement: Supplementary file 1 [file DataSheet_1.zip › The miRNA-target modules identified by the CleaveLand4/miR2111-5p_evm.model.LG01.6312_128_TPlot.pdf]

**T=evm.model.LG02.3374\_Q=miR319a-3p\_S=859**

category=0\_p=0.0169895595119902

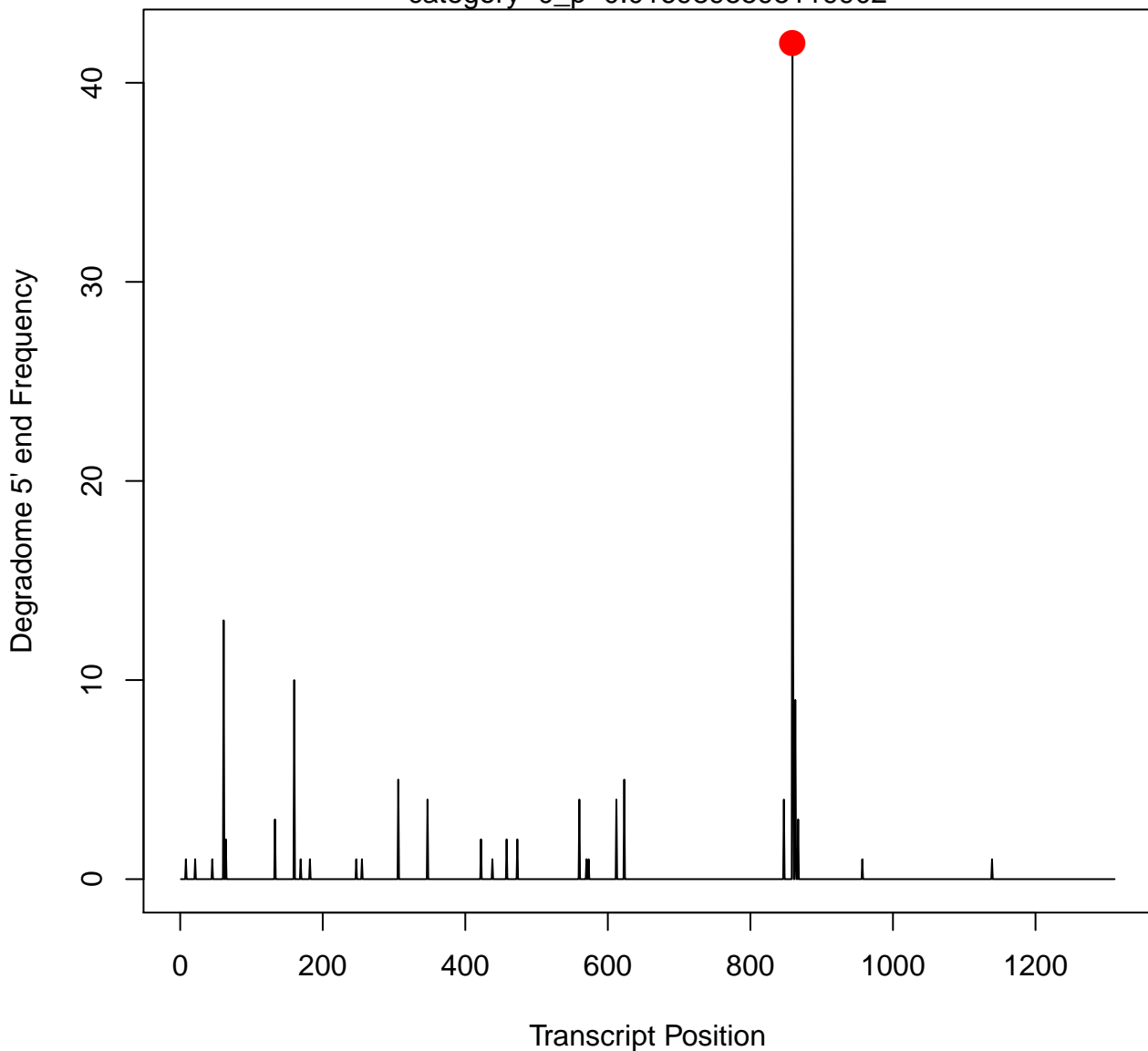

Supplement: Supplementary file 1 [file DataSheet_1.zip › The miRNA-target modules identified by the CleaveLand4/miR319a-3p_evm.model.LG02.3374_859_TPlot.pdf]

**T=evm.model.LG02.1320\_Q=miR319b-5p\_S=142**

category=1\_p=0.0289849666398476

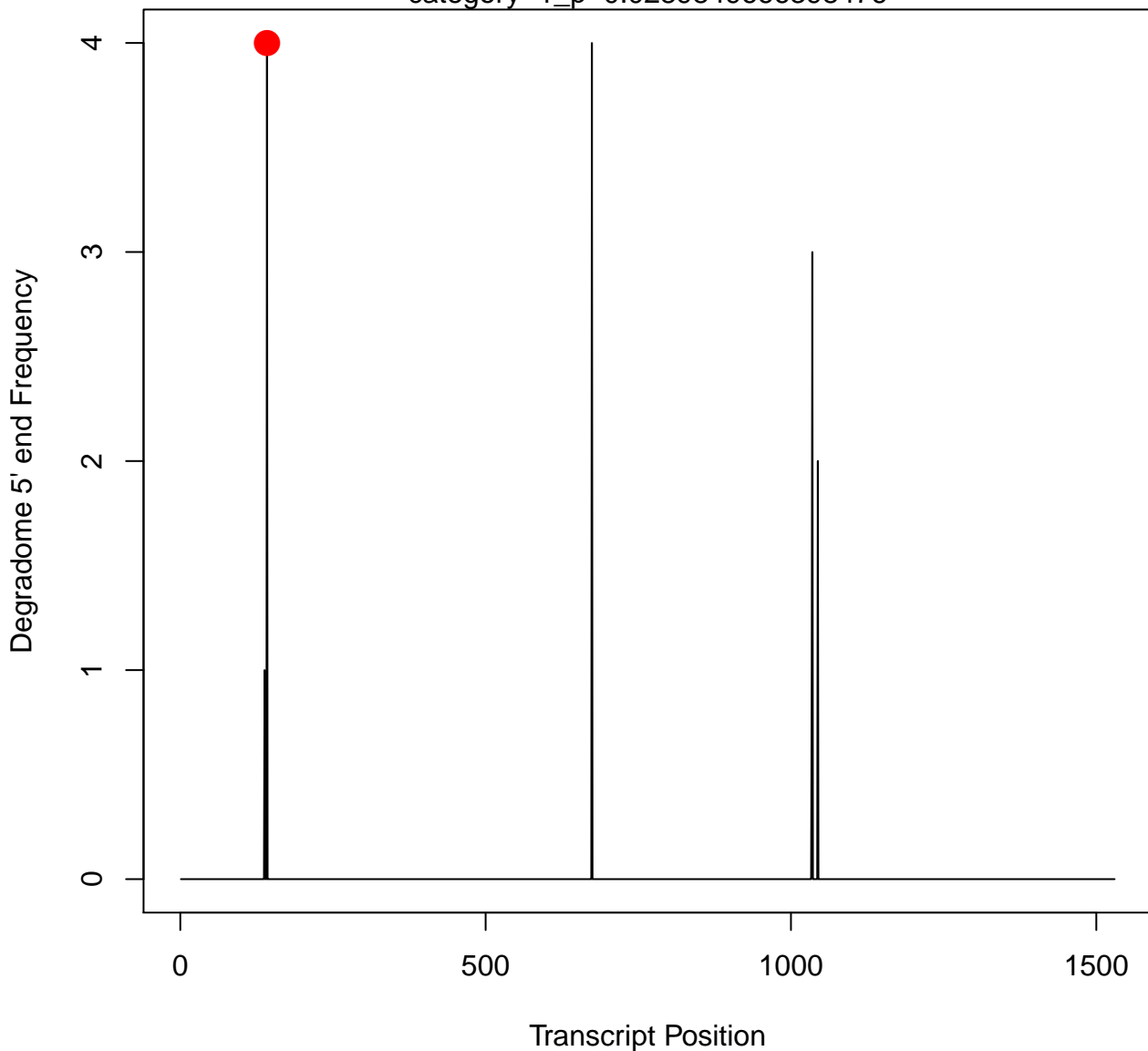

Supplement: Supplementary file 1 [file DataSheet_1.zip › The miRNA-target modules identified by the CleaveLand4/miR319b-5p_evm.model.LG02.1320_142_TPlot.pdf]

**T=evm.model.LG03.4917\_Q=miR319d-3p\_S=1118**

category=4\_p=0.0423765939260693

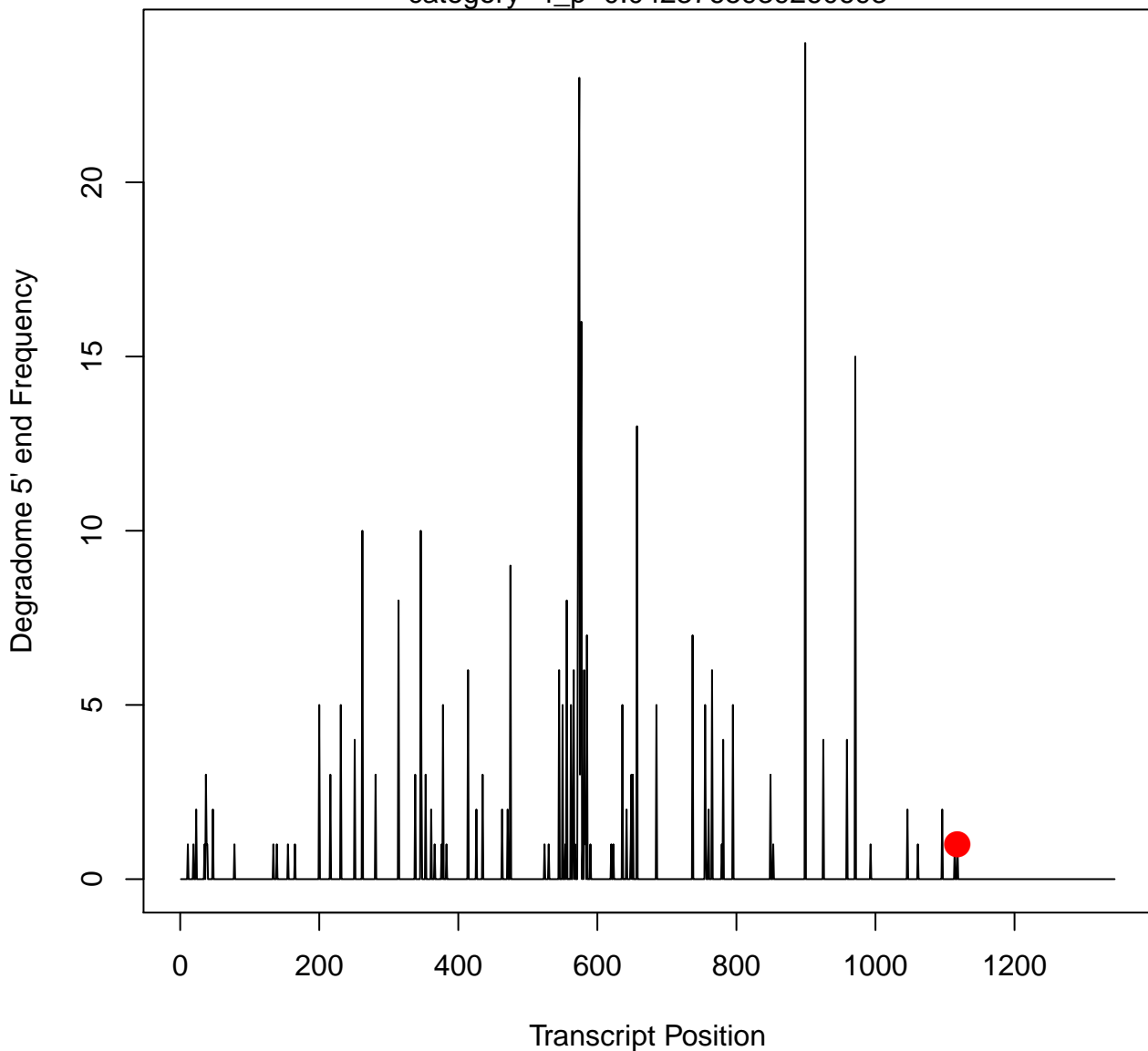

Supplement: Supplementary file 1 [file DataSheet_1.zip › The miRNA-target modules identified by the CleaveLand4/miR319d-3p_evm.model.LG03.4917_1118_TPlot.pdf]

**T=evm.model.LG06.3953\_Q=miR319d-3p\_S=1139**

category=2\_p=0.0174234189334928

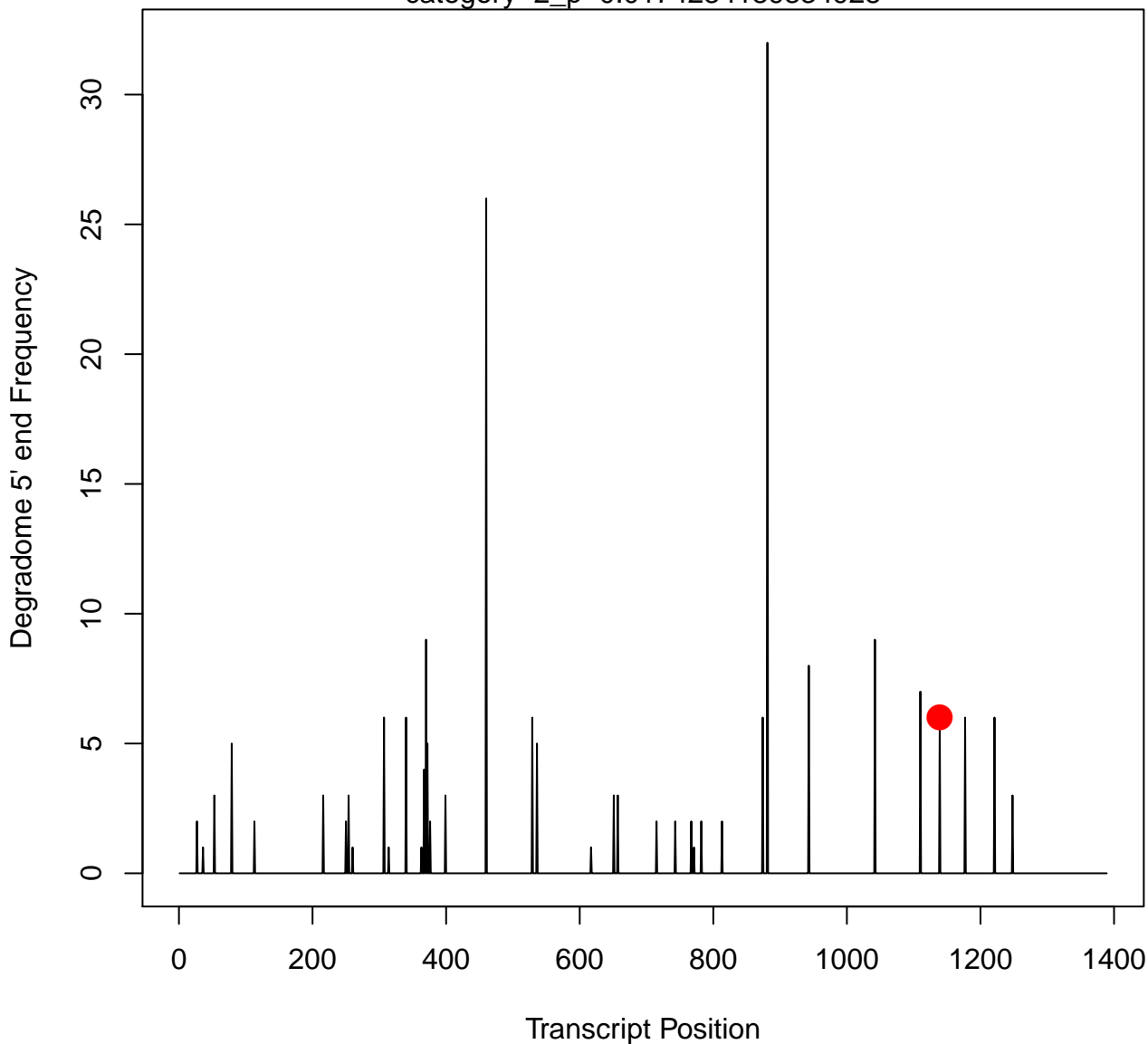

Supplement: Supplementary file 1 [file DataSheet_1.zip › The miRNA-target modules identified by the CleaveLand4/miR319d-3p_evm.model.LG06.3953_1139_TPlot.pdf]

**T=evm.model.LG08.1351\_Q=miR319d-3p\_S=911**

category=1\_p=0.00133003455890757

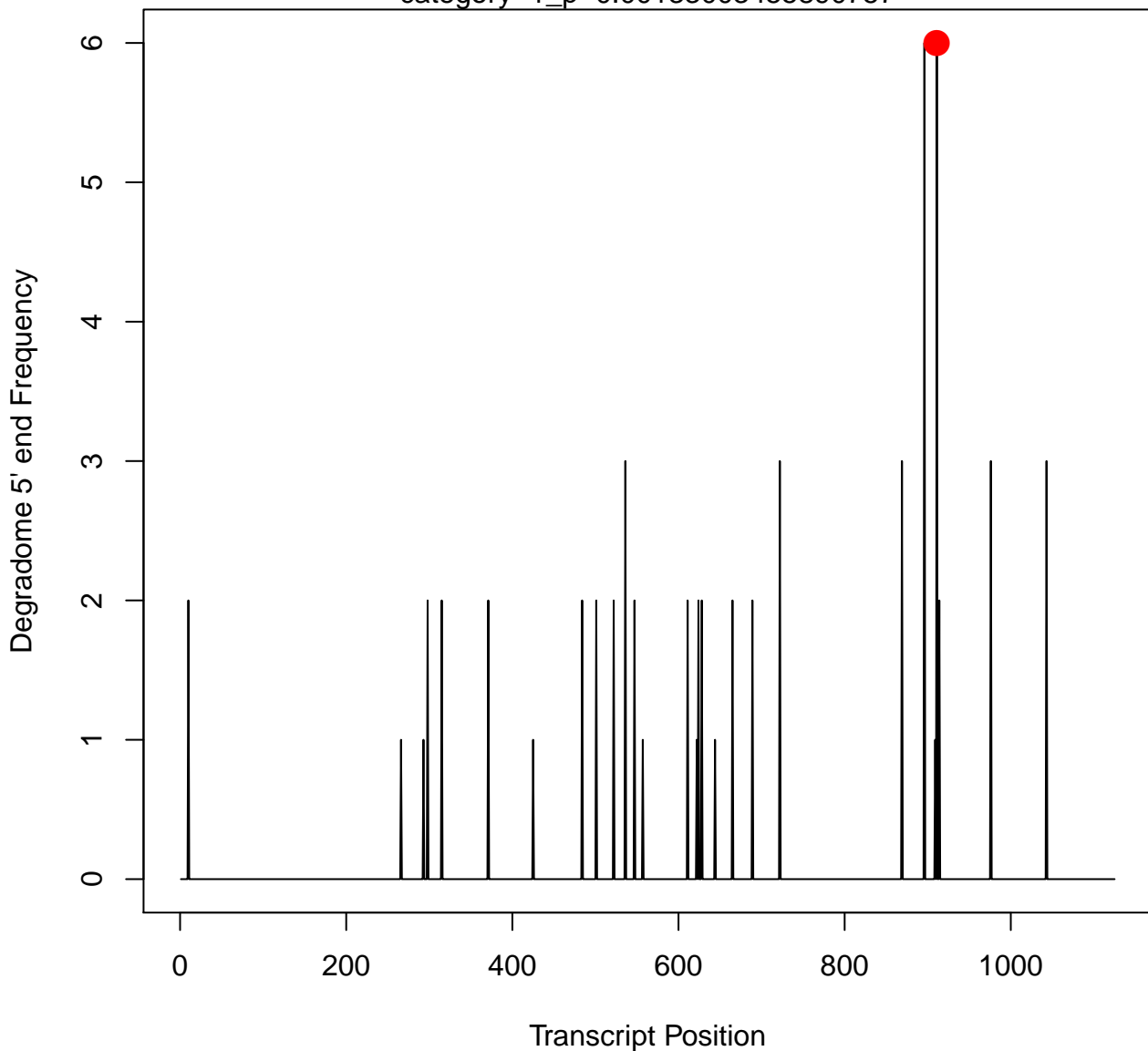

Supplement: Supplementary file 1 [file DataSheet_1.zip › The miRNA-target modules identified by the CleaveLand4/miR319d-3p_evm.model.LG08.1351_911_TPlot.pdf]

**T=evm.model.LG06.353\_Q=miR3623a-5p\_S=287**

category=2\_p=0.00874999063480097

Degradome 5' end Frequency

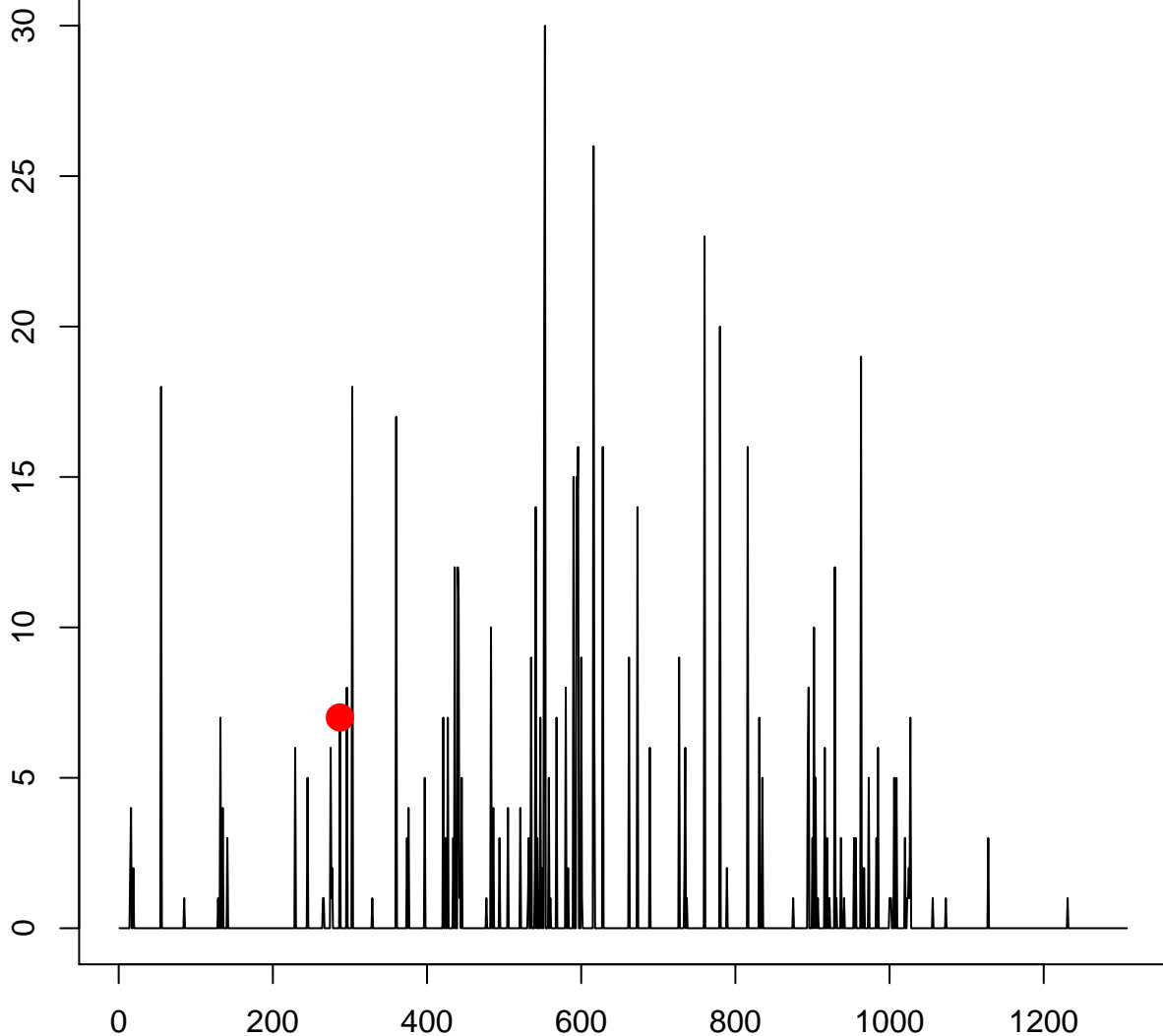

Transcript Position

Supplement: Supplementary file 1 [file DataSheet_1.zip › The miRNA-target modules identified by the CleaveLand4/miR3623a-5p_evm.model.LG06.353_287_TPlot.pdf]

**T=evm.model.LG06.4121\_Q=miR390b-5p\_S=909**

category=2\_p=0.042990999752487

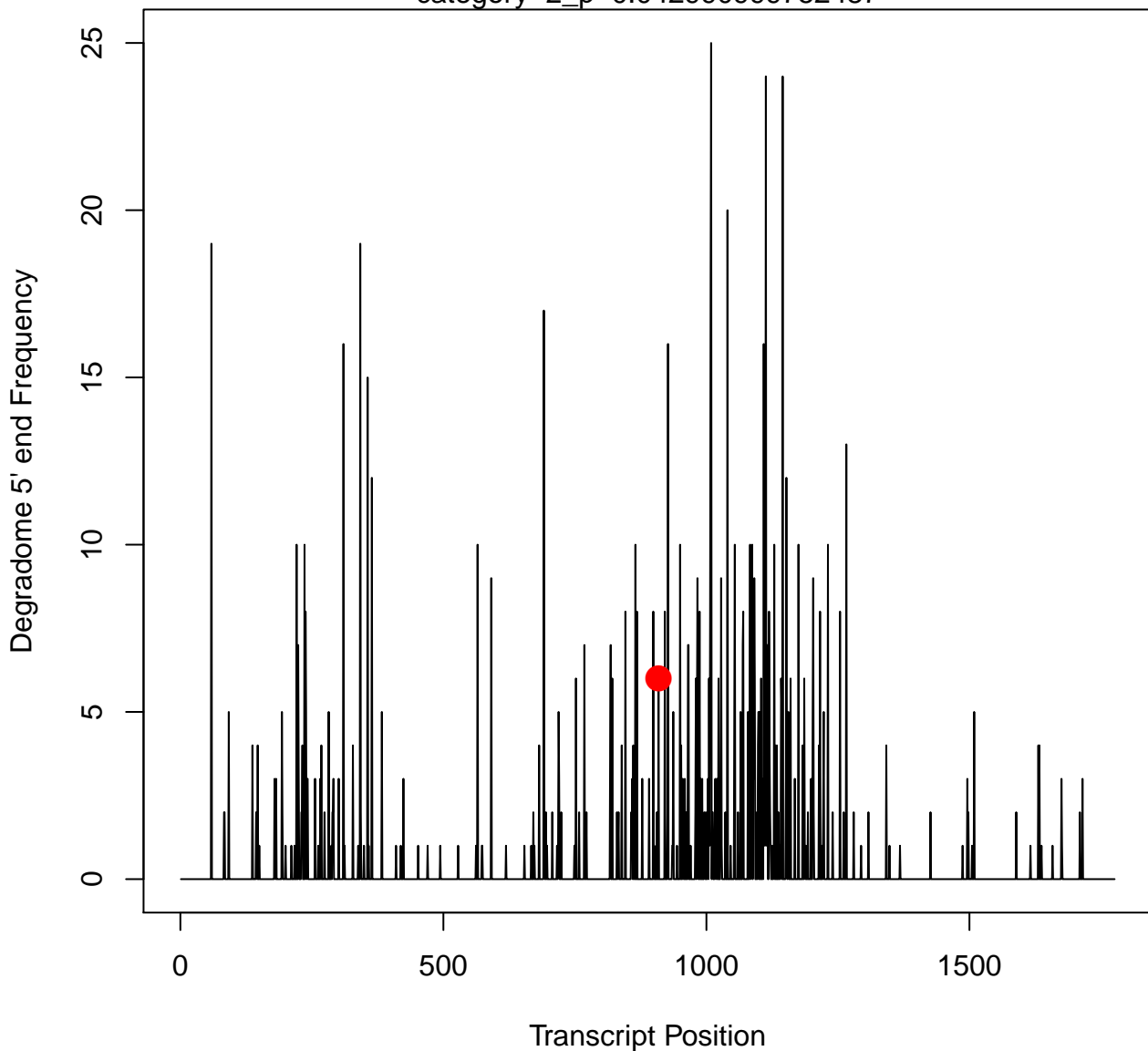

Supplement: Supplementary file 1 [file DataSheet_1.zip › The miRNA-target modules identified by the CleaveLand4/miR390b-5p_evm.model.LG06.4121_909_TPlot.pdf]

**T=evm.model.LG01.4654\_Q=miR393a-5p\_S=1516**

category=0\_p=0.00103797837257324

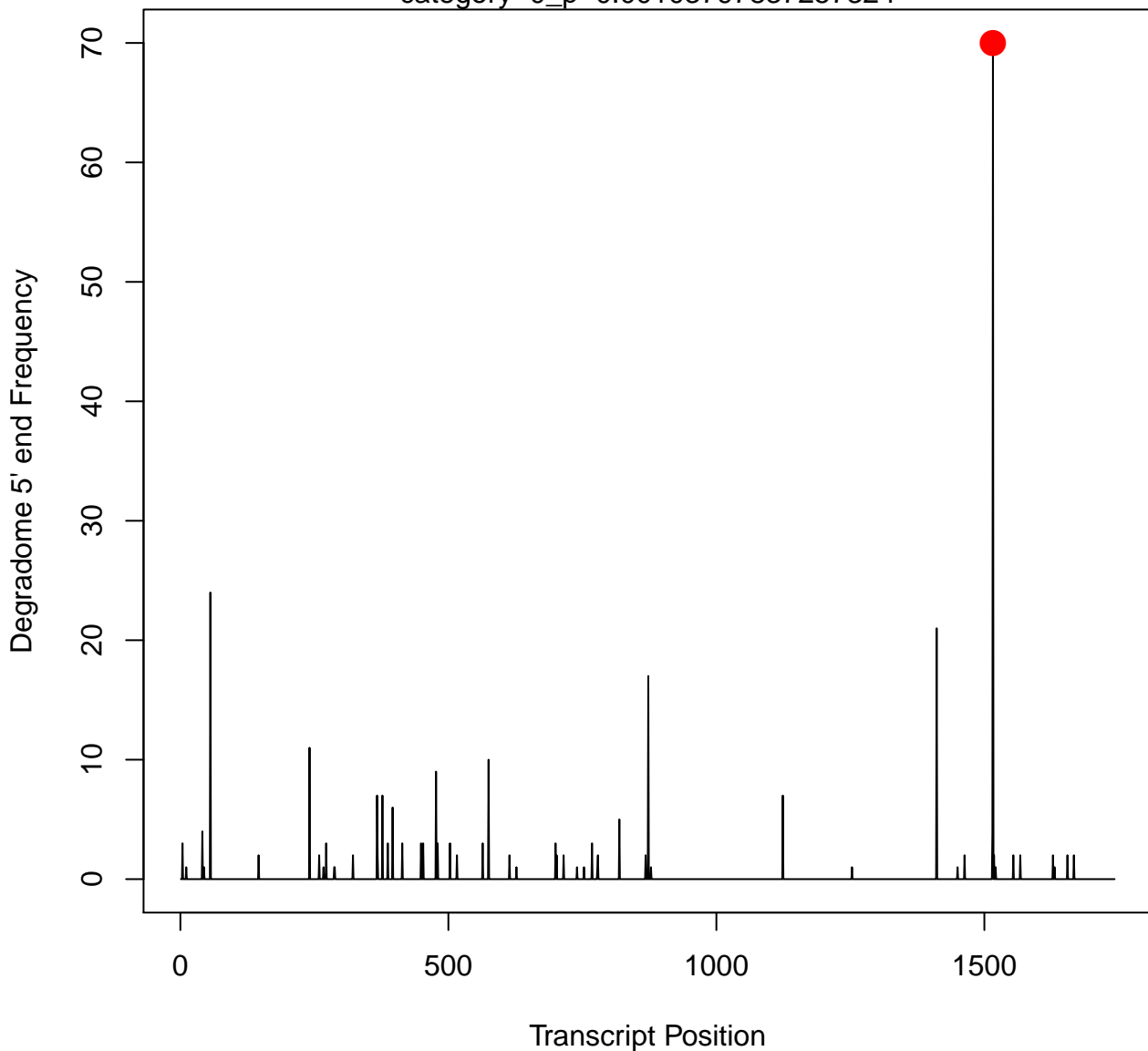

Supplement: Supplementary file 1 [file DataSheet_1.zip › The miRNA-target modules identified by the CleaveLand4/miR393a-5p_evm.model.LG01.4654_1516_TPlot.pdf]

**T=evm.model.LG01.5083\_Q=miR393a-5p\_S=770**

category=0\_p=0.0144340598055845

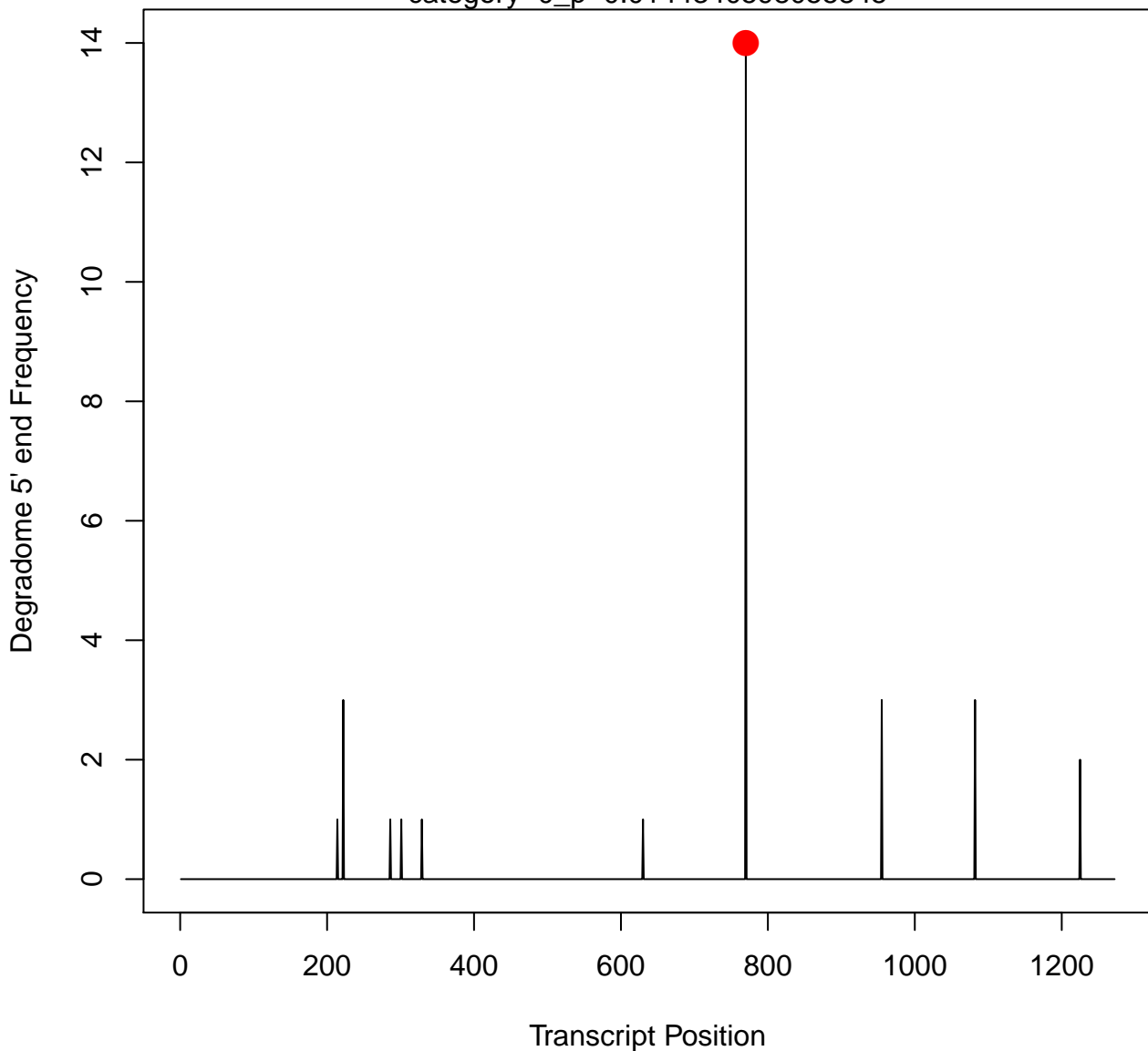

Supplement: Supplementary file 1 [file DataSheet_1.zip › The miRNA-target modules identified by the CleaveLand4/miR393a-5p_evm.model.LG01.5083_770_TPlot.pdf]

**T=evm.model.LG02.1135\_Q=miR393a-5p\_S=1516**

category=2\_p=0.00874999063480097

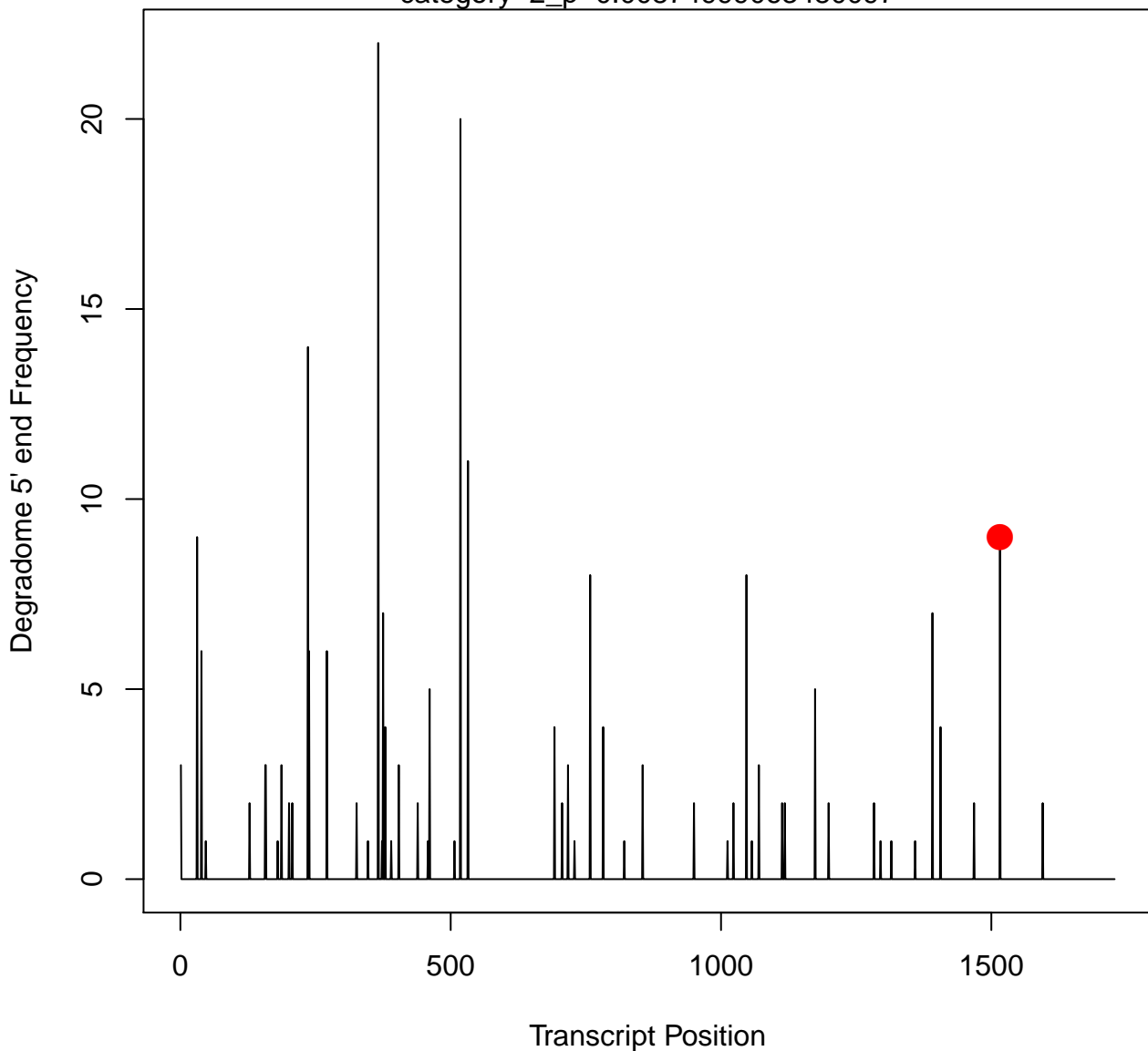

Supplement: Supplementary file 1 [file DataSheet_1.zip › The miRNA-target modules identified by the CleaveLand4/miR393a-5p_evm.model.LG02.1135_1516_TPlot.pdf]

**T=evm.model.LG07.185\_Q=miR393a-5p\_S=1576**

category=0\_p=0.0256287970925908

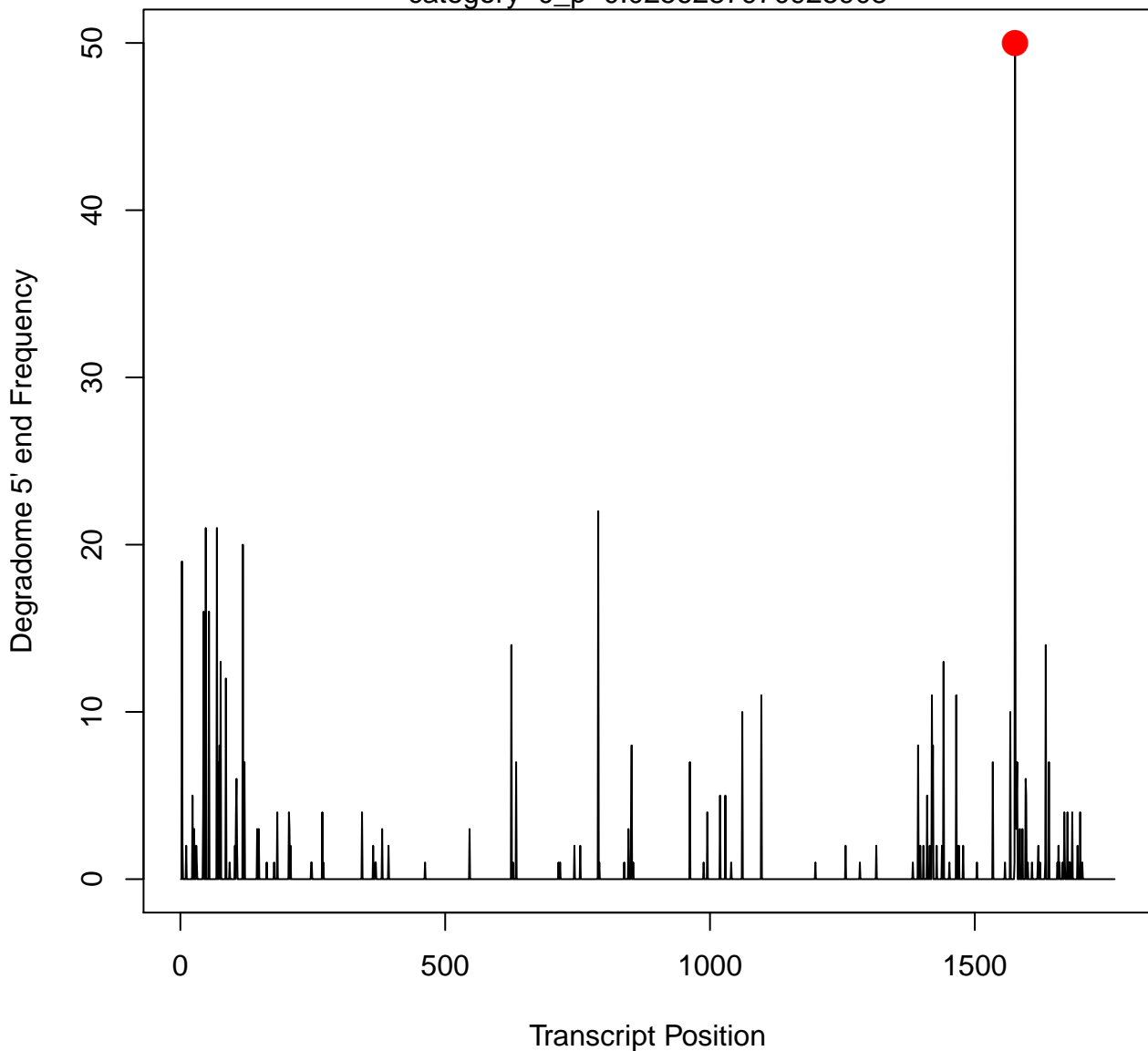

Supplement: Supplementary file 1 [file DataSheet_1.zip › The miRNA-target modules identified by the CleaveLand4/miR393a-5p_evm.model.LG07.185_1576_TPlot.pdf]

**T=evm.model.LG04.1183\_Q=miR393b-5p\_S=1519**

category=0\_p=0.00207487934604456

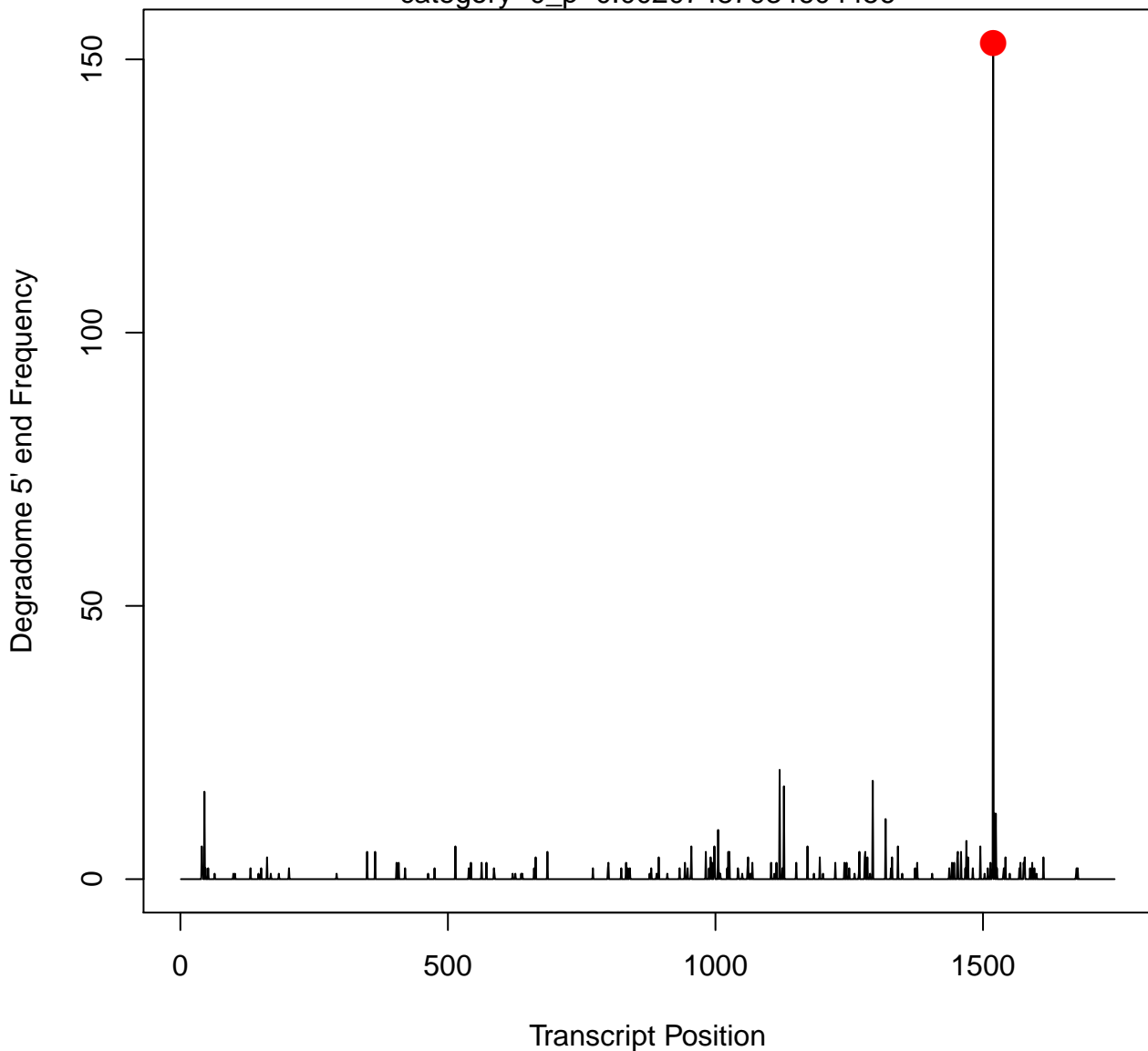

Supplement: Supplementary file 1 [file DataSheet_1.zip › The miRNA-target modules identified by the CleaveLand4/miR393b-5p_evm.model.LG04.1183_1519_TPlot.pdf]

**T=evm.model.LG04.958\_Q=miR393b-5p\_S=36**

category=4\_p=0.0423765939260693

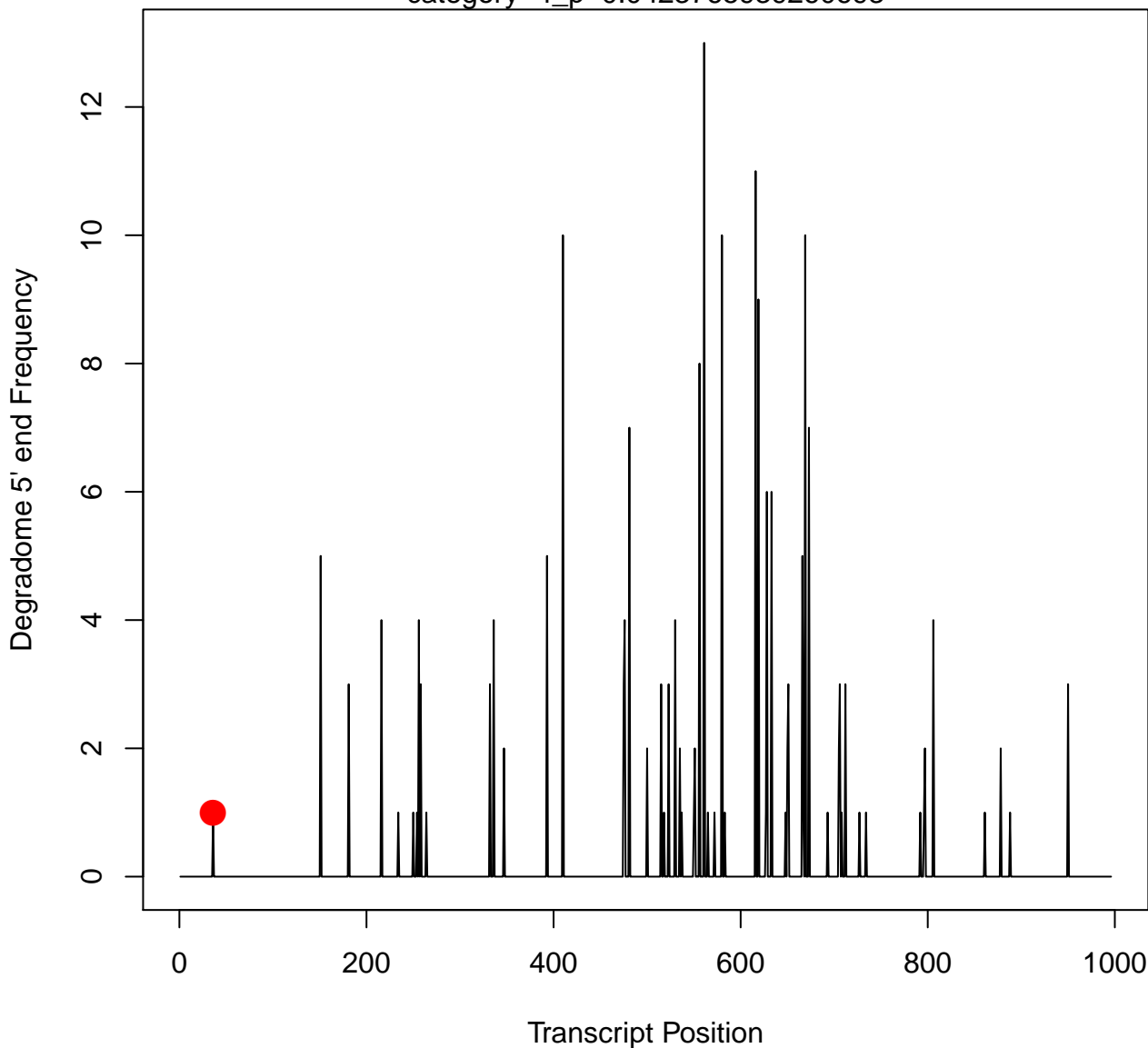

Supplement: Supplementary file 1 [file DataSheet_1.zip › The miRNA-target modules identified by the CleaveLand4/miR393b-5p_evm.model.LG04.958_36_TPlot.pdf]

**T=evm.model.LG07.1621\_Q=miR393b-5p\_S=171**

category=2\_p=0.0260209548157995

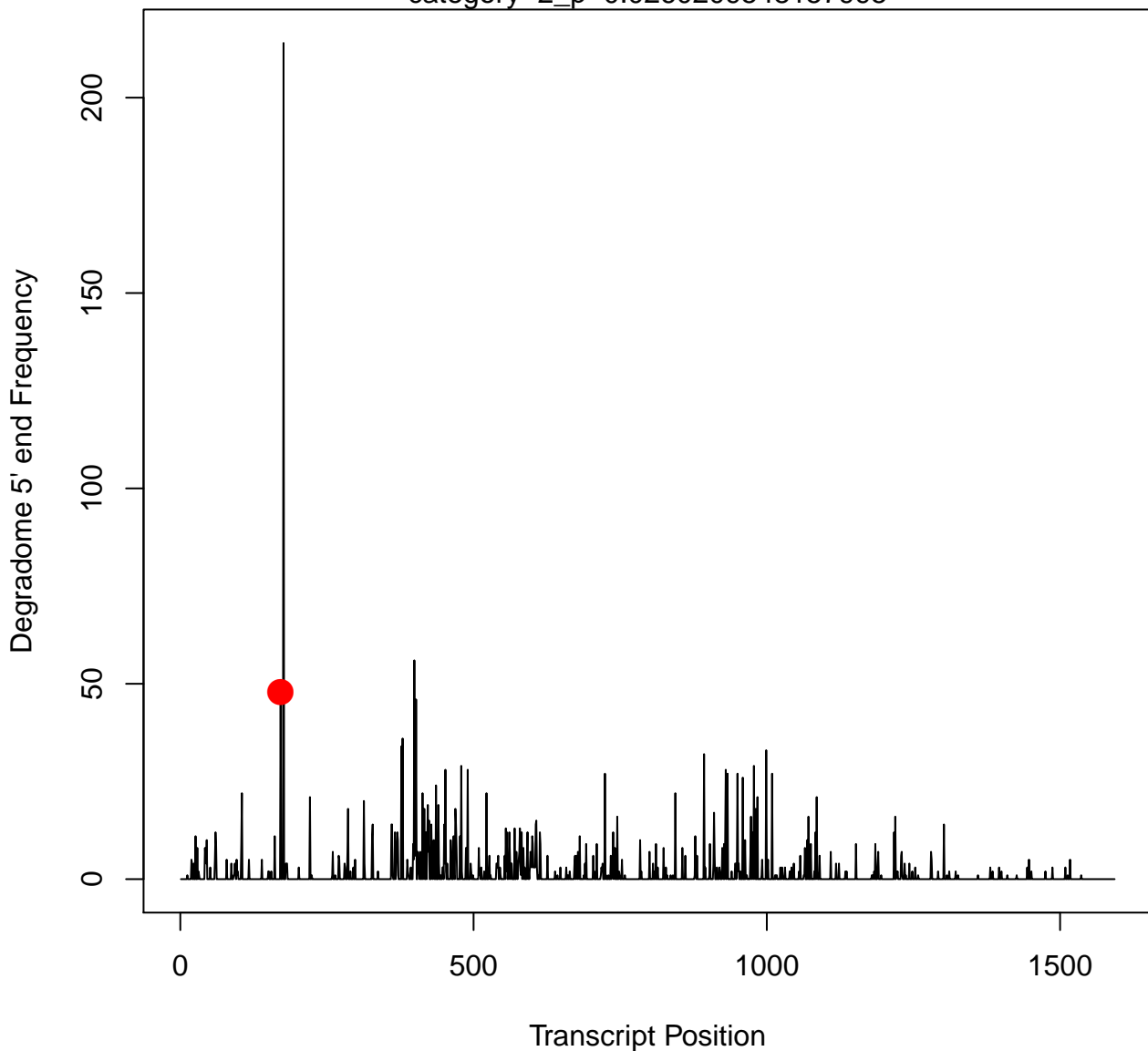

Supplement: Supplementary file 1 [file DataSheet_1.zip › The miRNA-target modules identified by the CleaveLand4/miR393b-5p_evm.model.LG07.1621_171_TPlot.pdf]

**T=evm.model.LG03.4927\_Q=miR394a-5p\_S=1537**

category=1\_p=0.000266148544176392

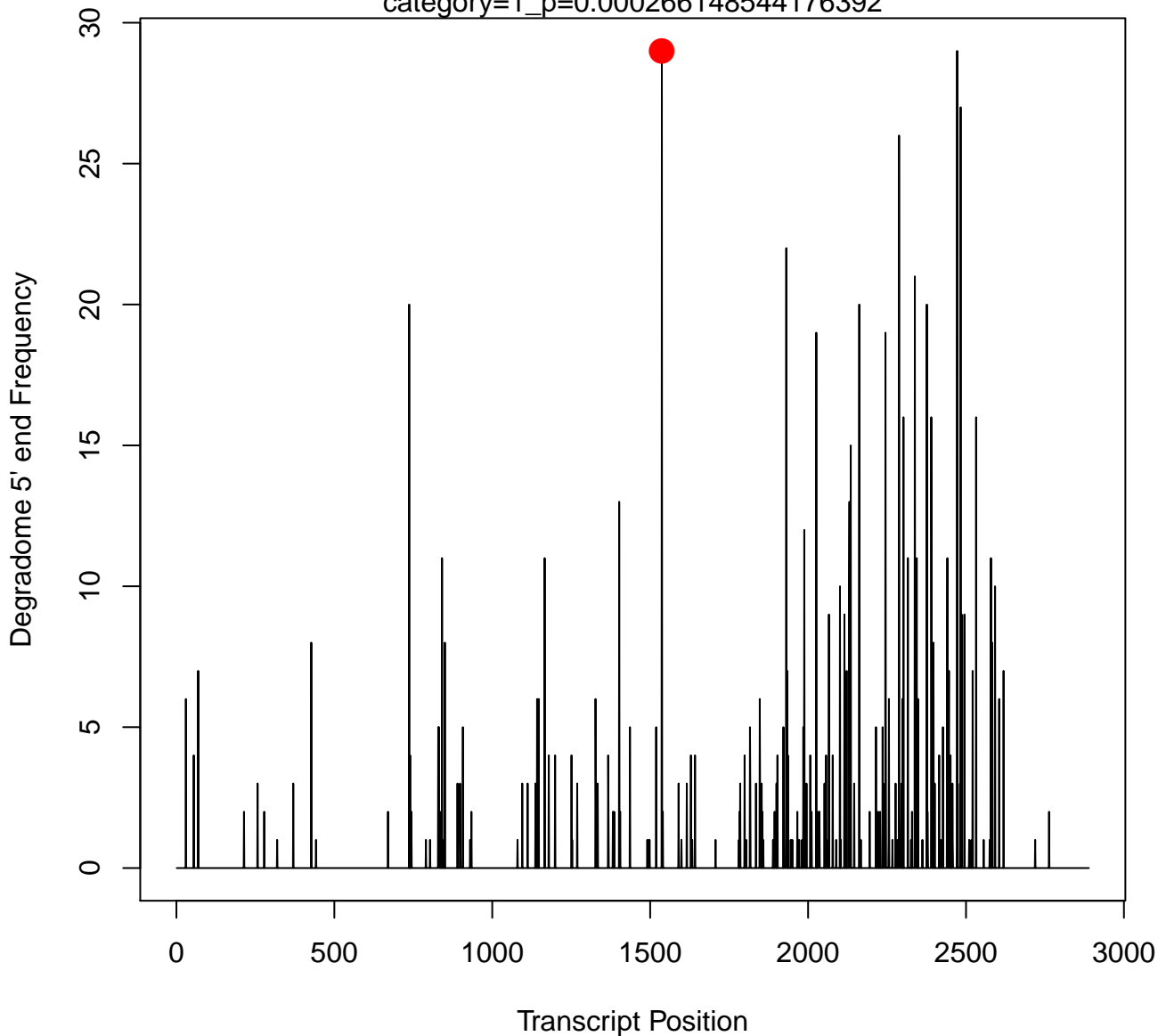

Supplement: Supplementary file 1 [file DataSheet_1.zip › The miRNA-target modules identified by the CleaveLand4/miR394a-5p_evm.model.LG03.4927_1537_TPlot.pdf]

**T=evm.model.LG05.3270\_Q=miR394a-5p\_S=1045**

category=2\_p=0.042990999752487

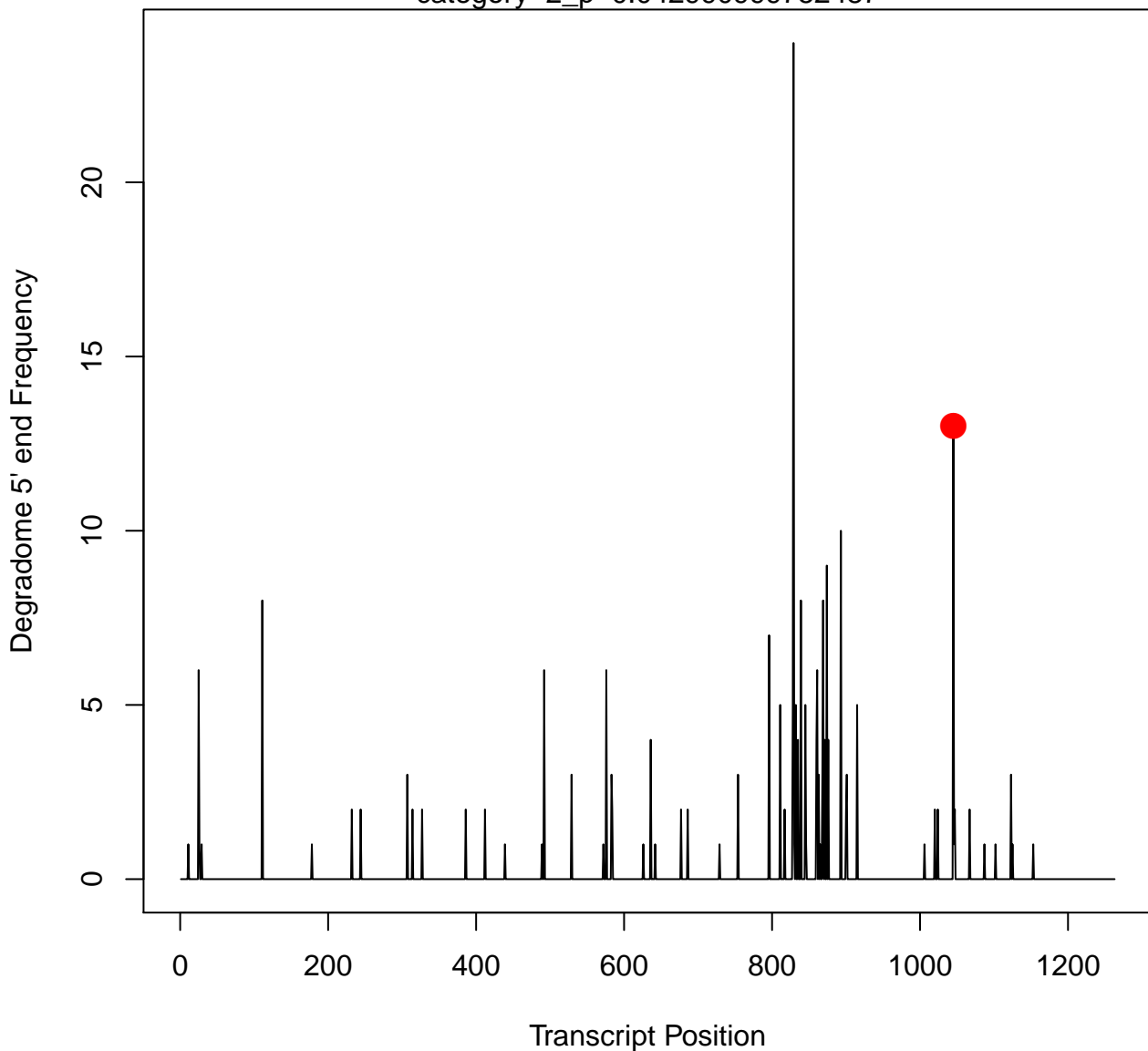

Supplement: Supplementary file 1 [file DataSheet_1.zip › The miRNA-target modules identified by the CleaveLand4/miR394a-5p_evm.model.LG05.3270_1045_TPlot.pdf]

**T=evm.model.LG04.2726\_Q=miR394c-5p\_S=1123**

category=2\_p=0.0174234189334928

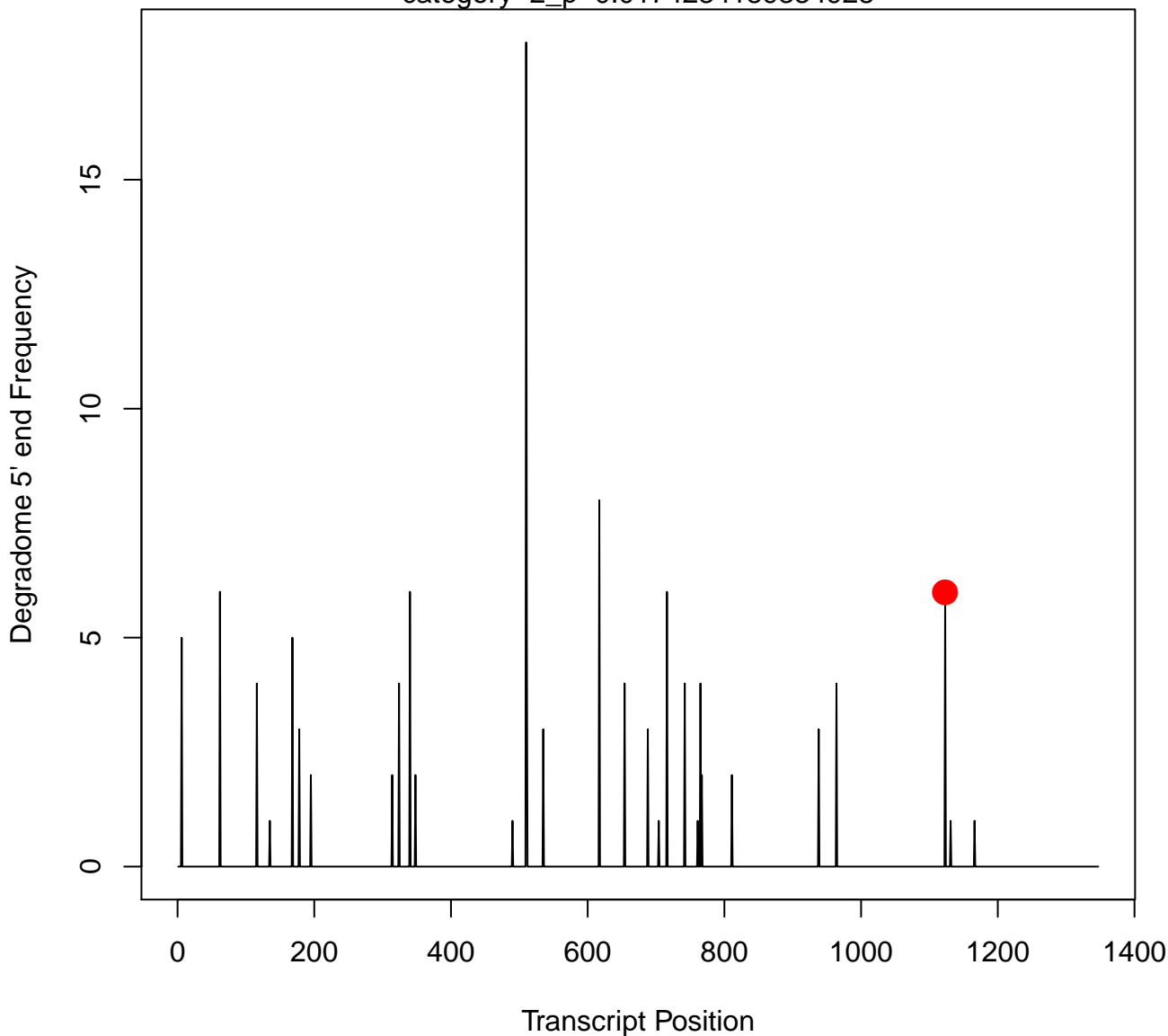

Supplement: Supplementary file 1 [file DataSheet_1.zip › The miRNA-target modules identified by the CleaveLand4/miR394c-5p_evm.model.LG04.2726_1123_TPlot.pdf]

**T=evm.model.LG05.3270\_Q=miR394c-5p\_S=1045**

category=2\_p=0.0260209548157995

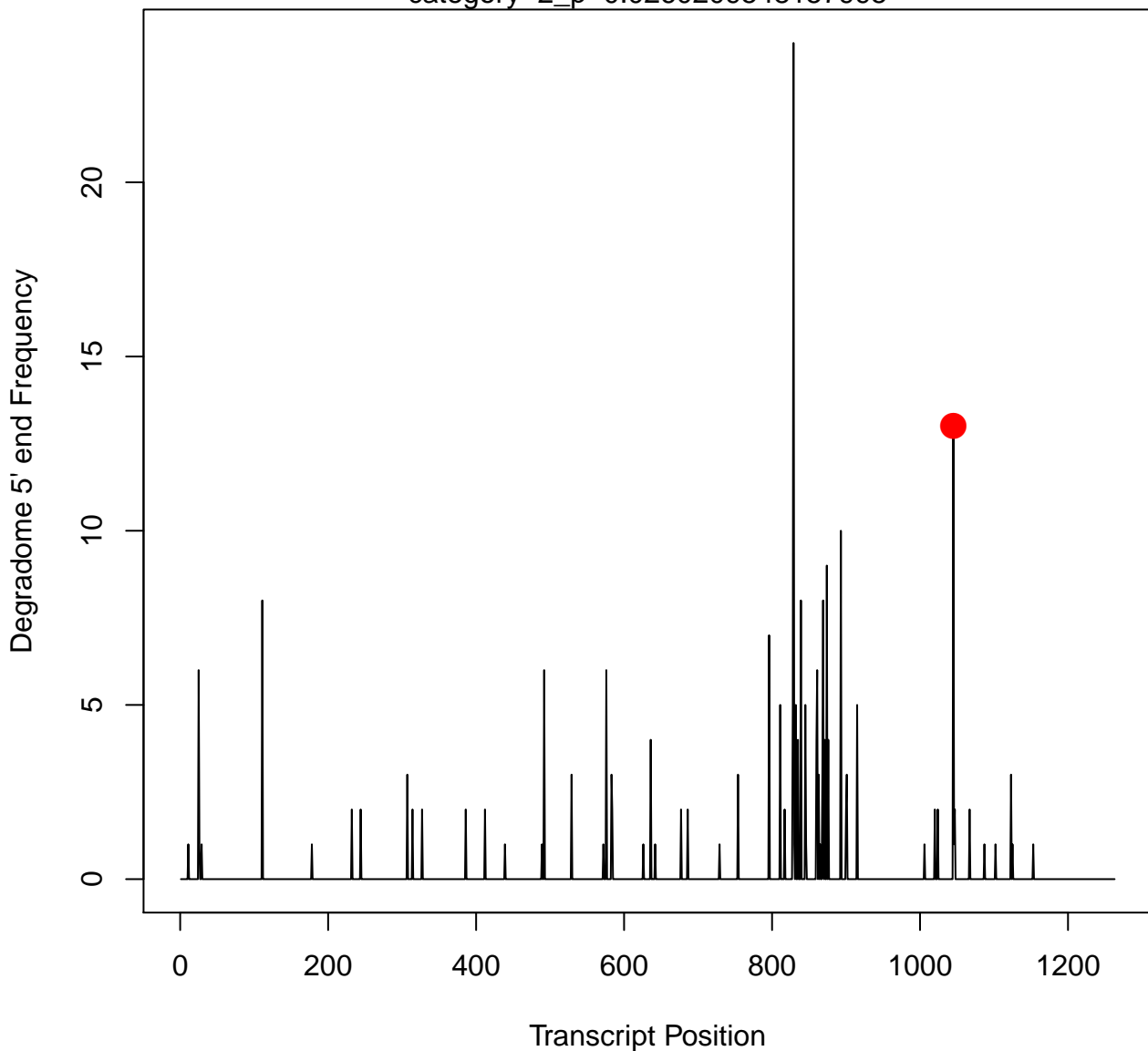

Supplement: Supplementary file 1 [file DataSheet_1.zip › The miRNA-target modules identified by the CleaveLand4/miR394c-5p_evm.model.LG05.3270_1045_TPlot.pdf]

**T=evm.model.LG02.1267\_Q=miR395a-3p\_S=342**

category=2\_p=0.00874999063480097

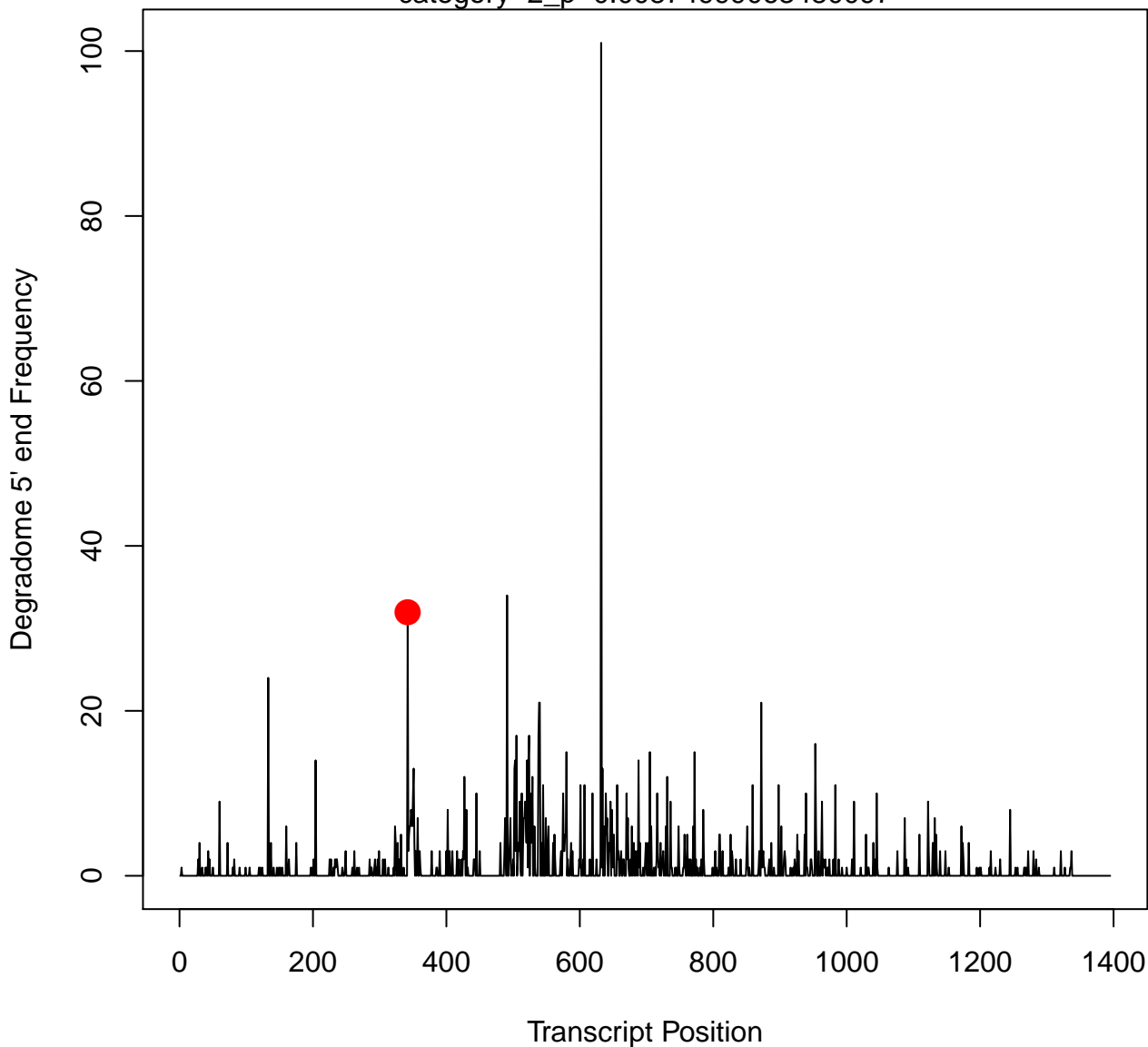

Supplement: Supplementary file 1 [file DataSheet_1.zip › The miRNA-target modules identified by the CleaveLand4/miR395a-3p_evm.model.LG02.1267_342_TPlot.pdf]

**T=evm.model.LG01.574\_Q=miR395c-5p\_S=225**

category=2\_p=0.0174234189334928

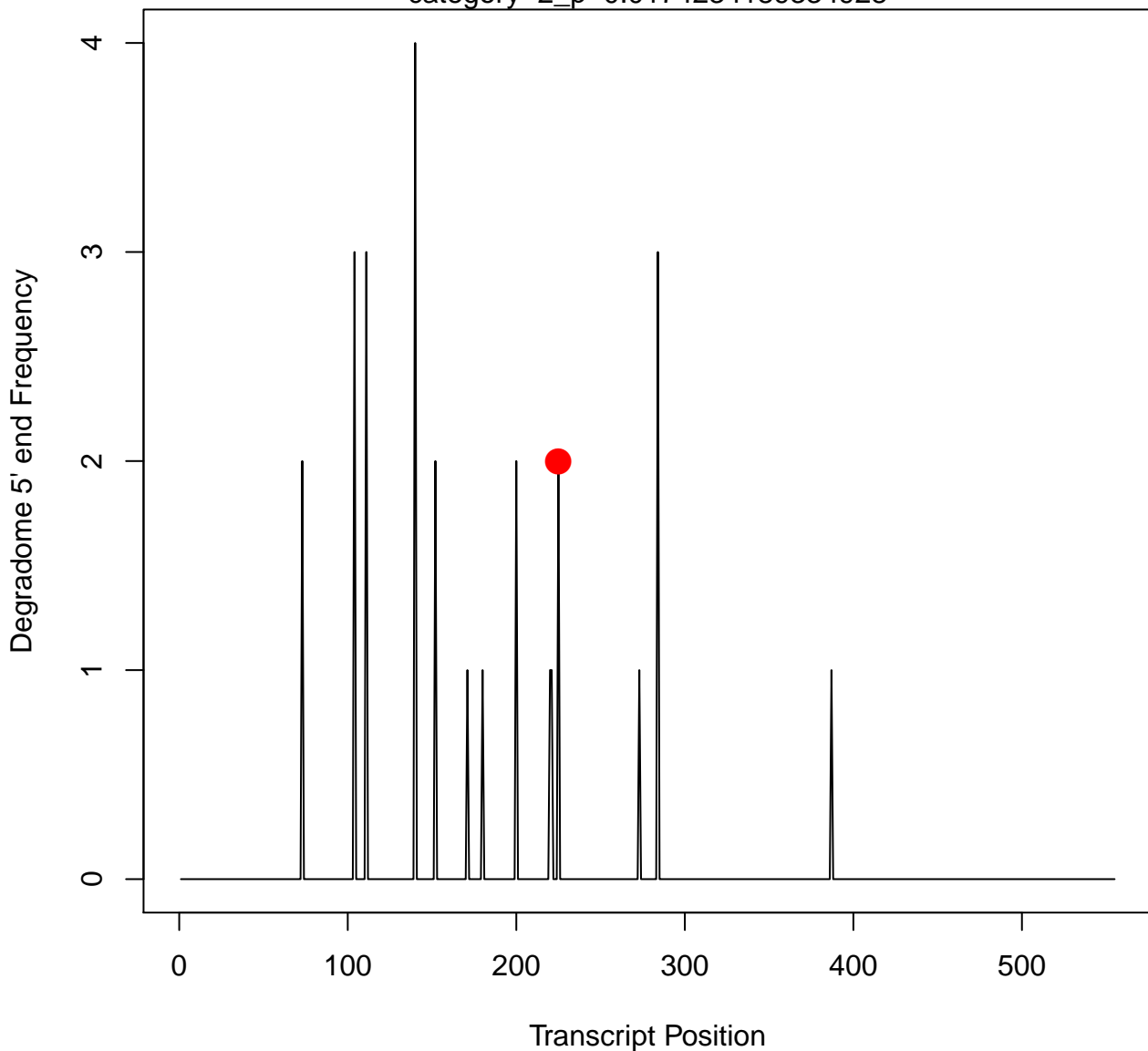

Supplement: Supplementary file 1 [file DataSheet_1.zip › The miRNA-target modules identified by the CleaveLand4/miR395c-5p_evm.model.LG01.574_225_TPlot.pdf]

**T=evm.model.LG03.5350\_Q=miR395g-5p\_S=395**

category=2\_p=0.0174234189334928

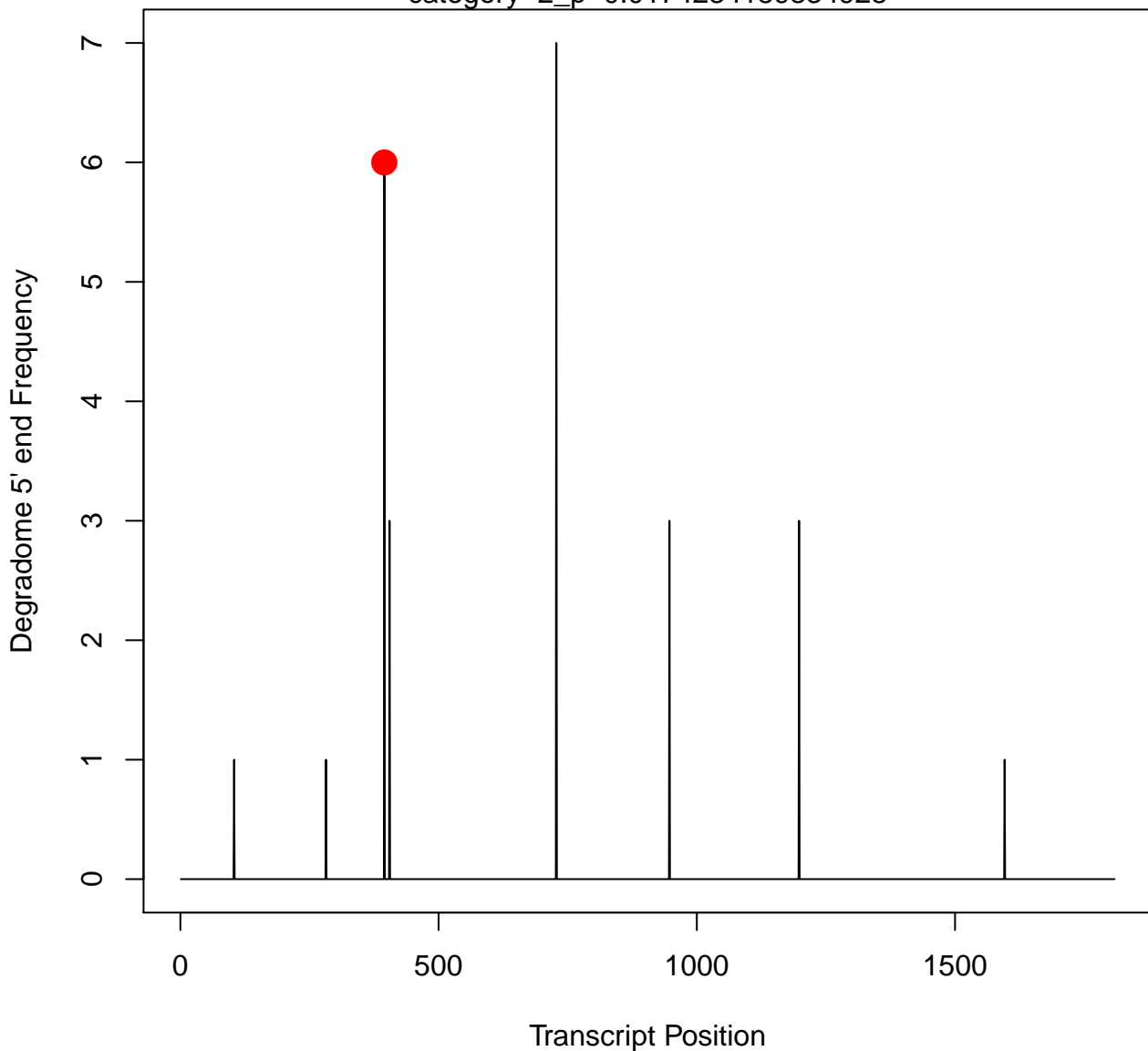

Supplement: Supplementary file 1 [file DataSheet_1.zip › The miRNA-target modules identified by the CleaveLand4/miR395g-5p_evm.model.LG03.5350_395_TPlot.pdf]

**T=evm.model.LG01.294\_Q=miR396a-5p\_S=1091**

category=0\_p=0.00259292615763651

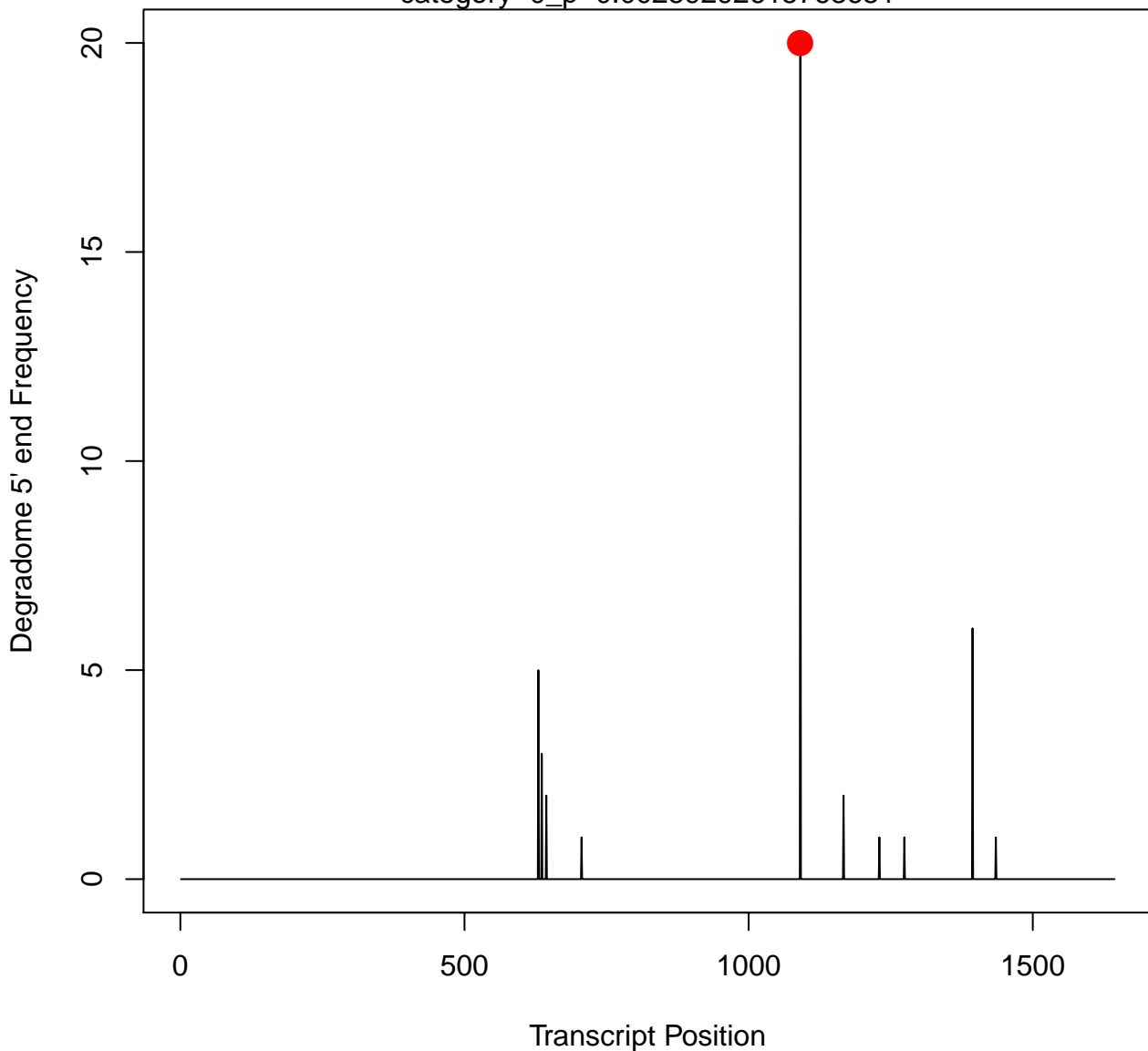

Supplement: Supplementary file 1 [file DataSheet_1.zip › The miRNA-target modules identified by the CleaveLand4/miR396a-5p_evm.model.LG01.294_1091_TPlot.pdf]

**T=evm.model.LG02.1585\_Q=miR396a-5p\_S=1671**

category=0\_p=0.016478990219059

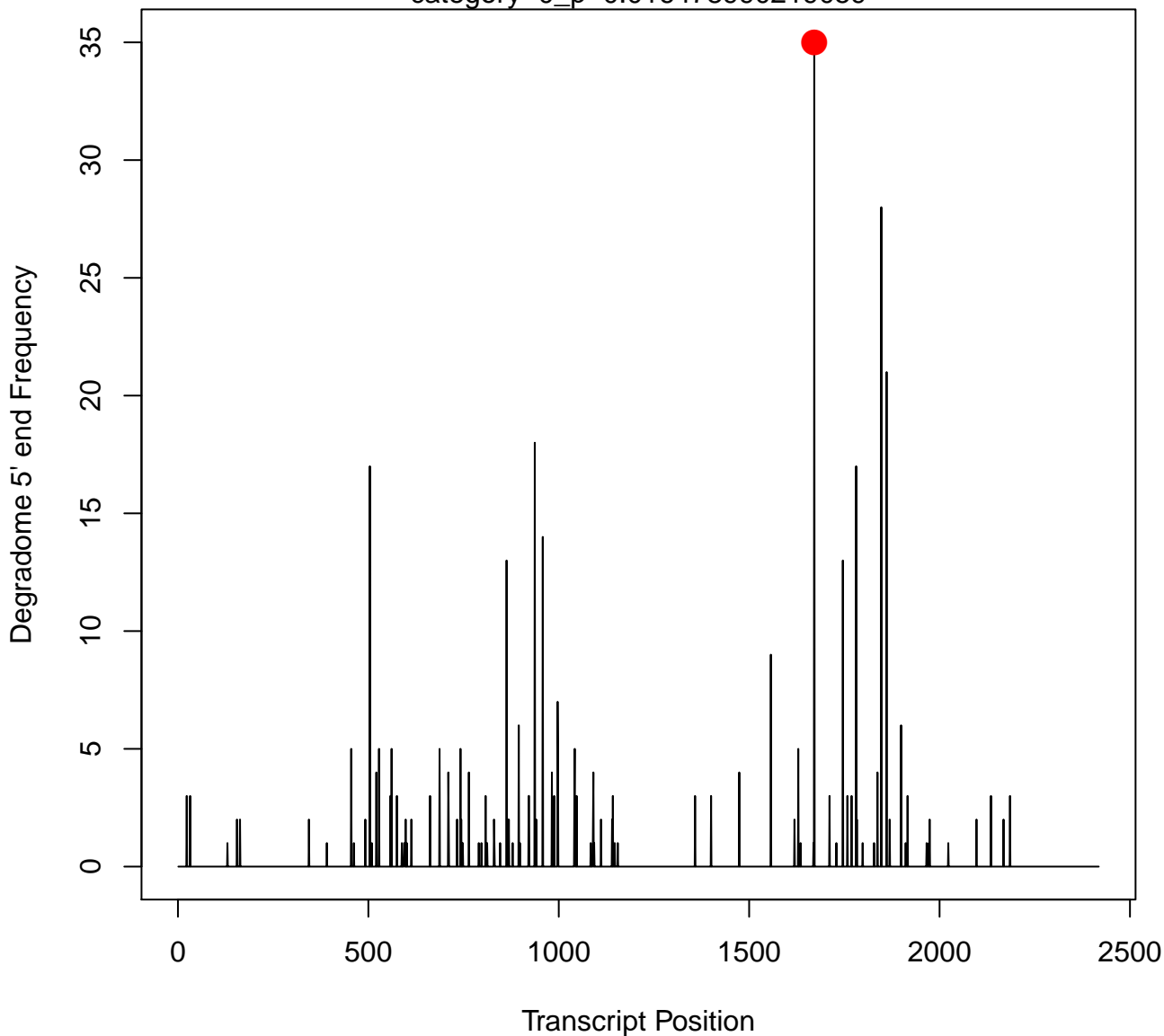

Supplement: Supplementary file 1 [file DataSheet_1.zip › The miRNA-target modules identified by the CleaveLand4/miR396a-5p_evm.model.LG02.1585_1671_TPlot.pdf]

**T=evm.model.LG02.2447\_Q=miR396a-5p\_S=383**

category=0\_p=0.00517912904921392

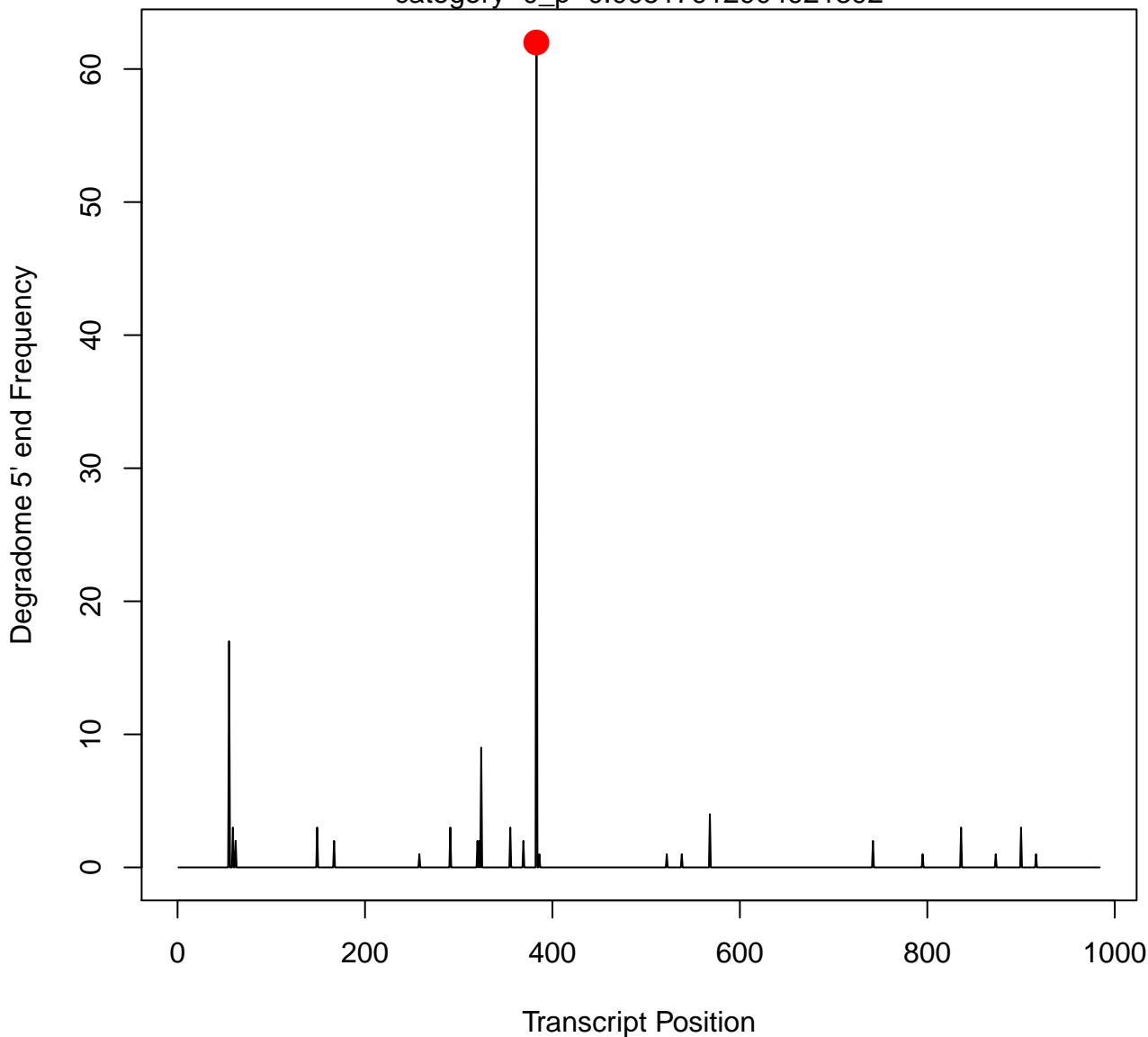

Supplement: Supplementary file 1 [file DataSheet_1.zip › The miRNA-target modules identified by the CleaveLand4/miR396a-5p_evm.model.LG02.2447_383_TPlot.pdf]

**T=evm.model.LG02.6265\_Q=miR396a-5p\_S=644**

category=0\_p=0.00724326232736372

Degradome 5' end Frequency

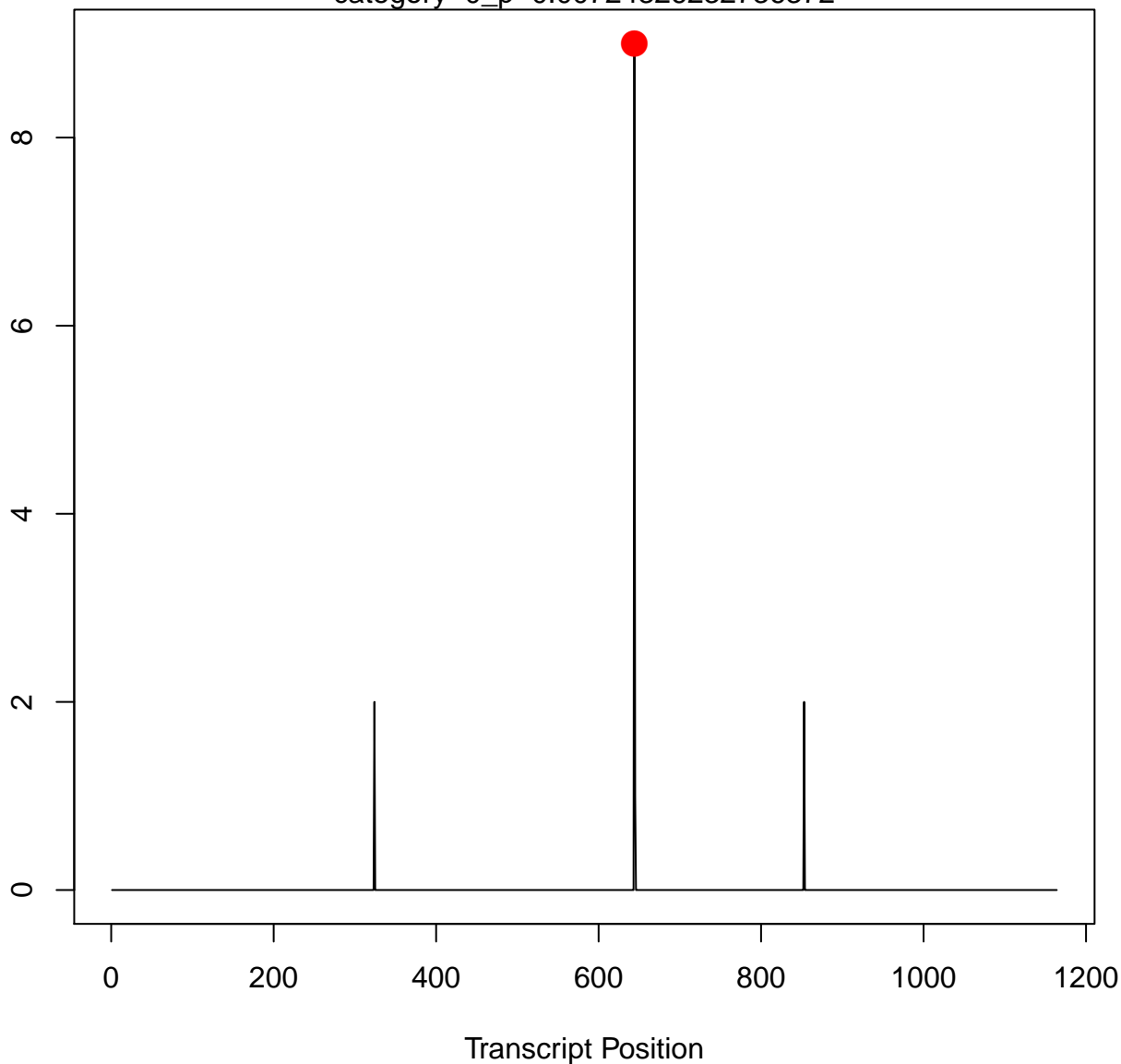

Supplement: Supplementary file 1 [file DataSheet_1.zip › The miRNA-target modules identified by the CleaveLand4/miR396a-5p_evm.model.LG02.6265_644_TPlot.pdf]

**T=evm.model.LG03.2927\_Q=miR396a-5p\_S=377**

category=0\_p=0.00775862610766487

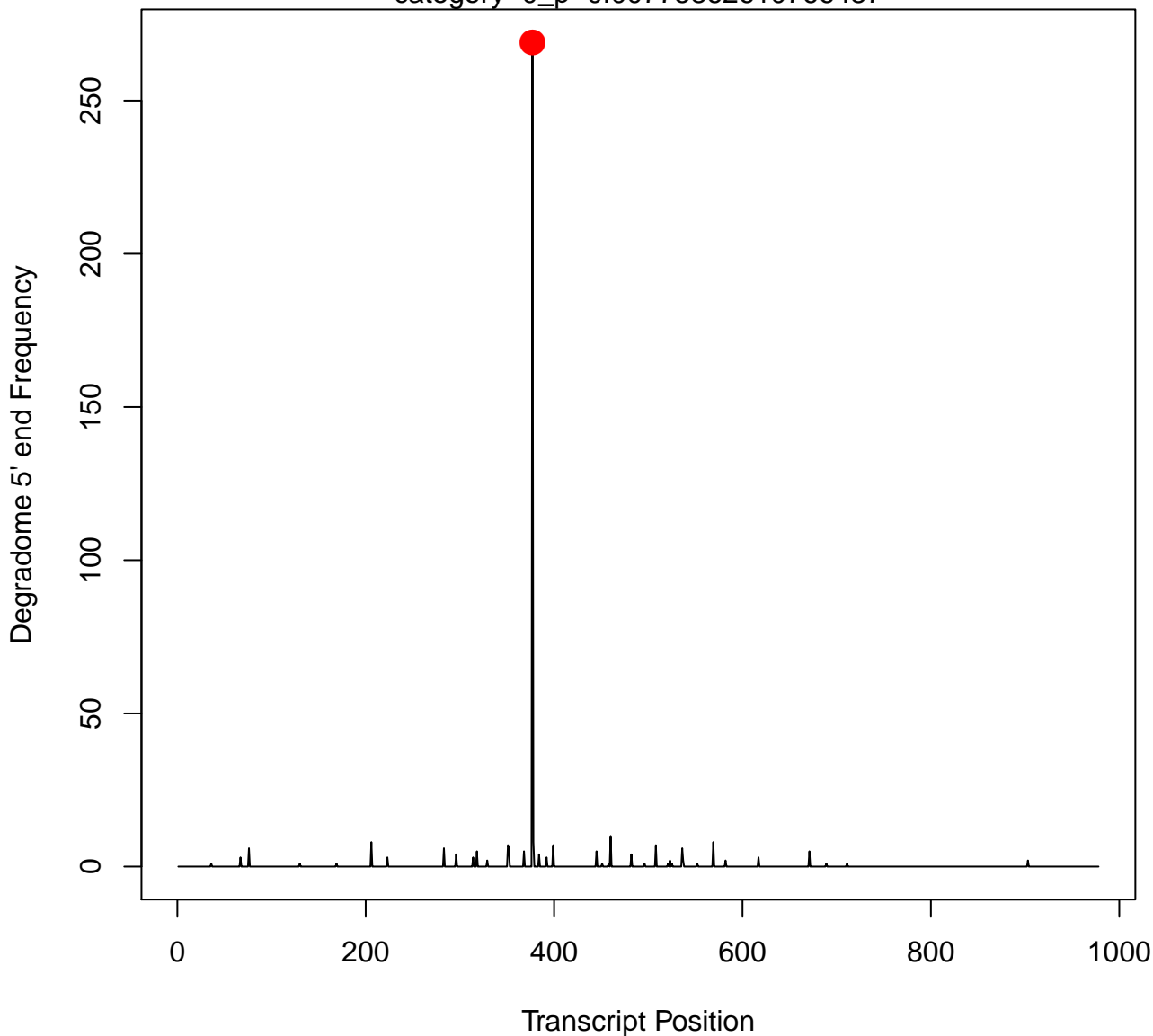

Supplement: Supplementary file 1 [file DataSheet_1.zip › The miRNA-target modules identified by the CleaveLand4/miR396a-5p_evm.model.LG03.2927_377_TPlot.pdf]

**T=evm.model.LG03.637\_Q=miR396a-5p\_S=728**

category=0\_p=0.00466242549475071

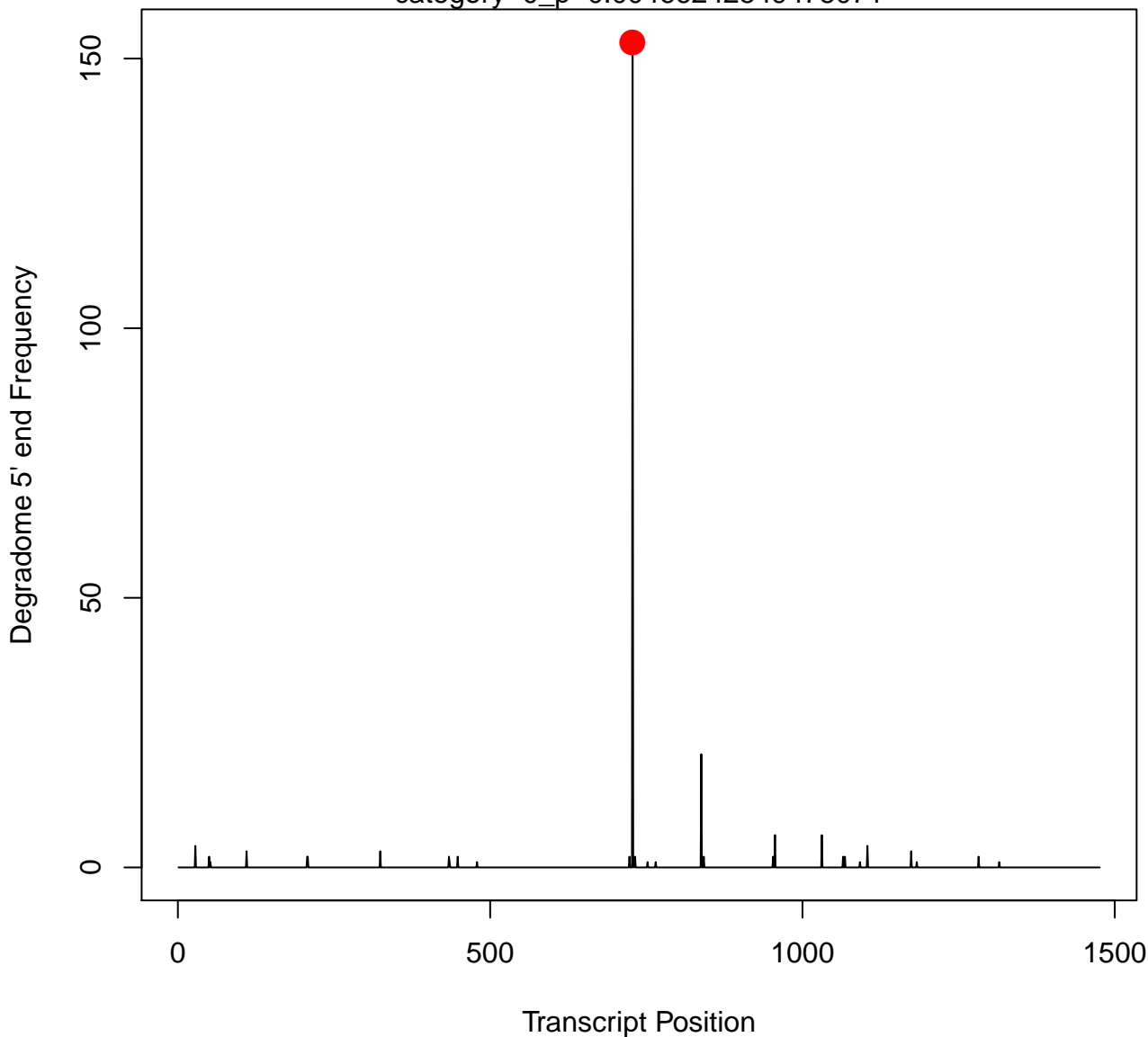

Supplement: Supplementary file 1 [file DataSheet_1.zip › The miRNA-target modules identified by the CleaveLand4/miR396a-5p_evm.model.LG03.637_728_TPlot.pdf]

**T=evm.model.LG04.662\_Q=miR396a-5p\_S=642**

category=0\_p=0.00103797837257324

Degradsome 5' end Frequency

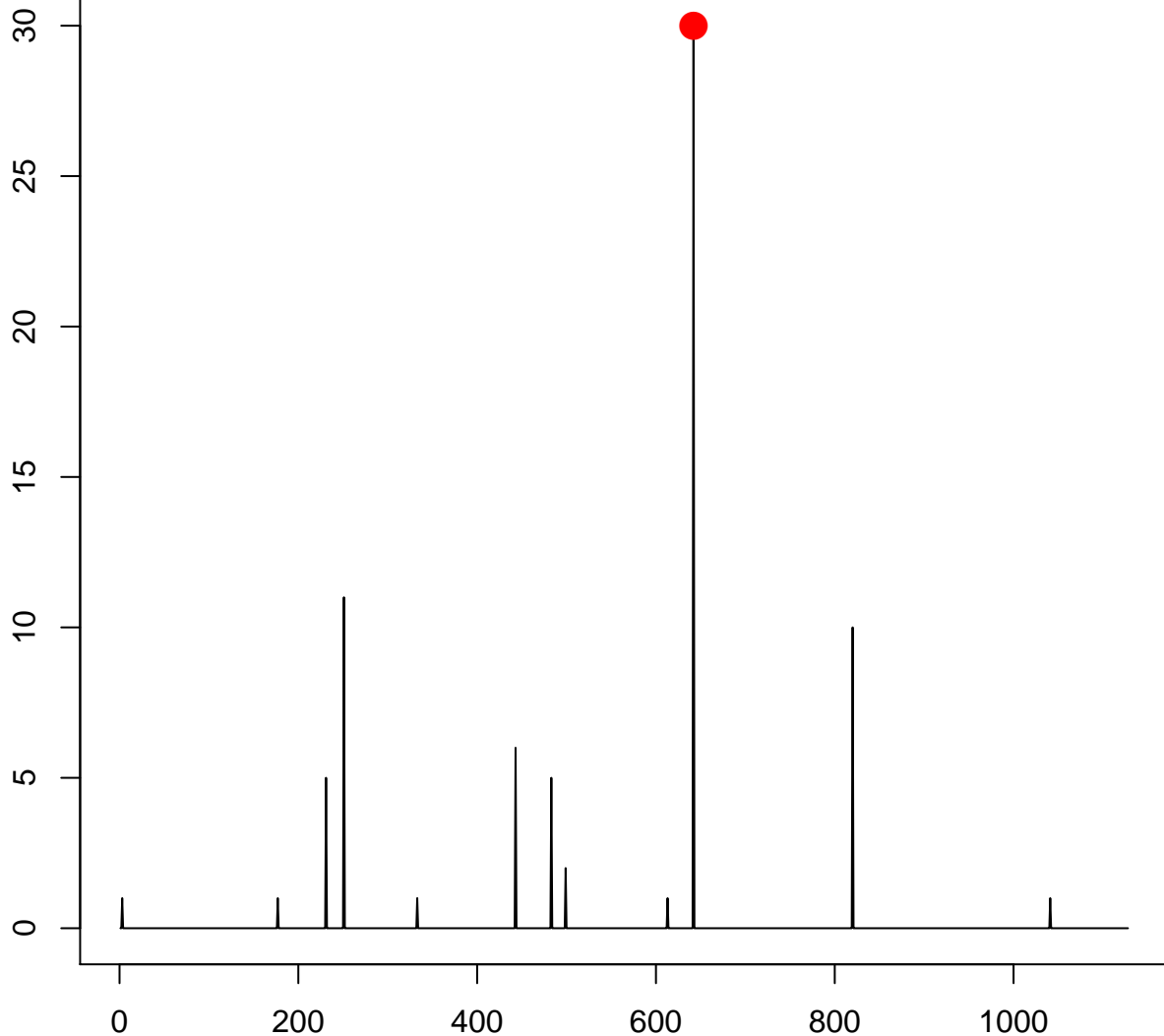

Transcript Position

Supplement: Supplementary file 1 [file DataSheet_1.zip › The miRNA-target modules identified by the CleaveLand4/miR396a-5p_evm.model.LG04.662_642_TPlot.pdf]

**T=evm.model.LG04.896\_Q=miR396a-5p\_S=374**

category=0\_p=0.00362821312893646

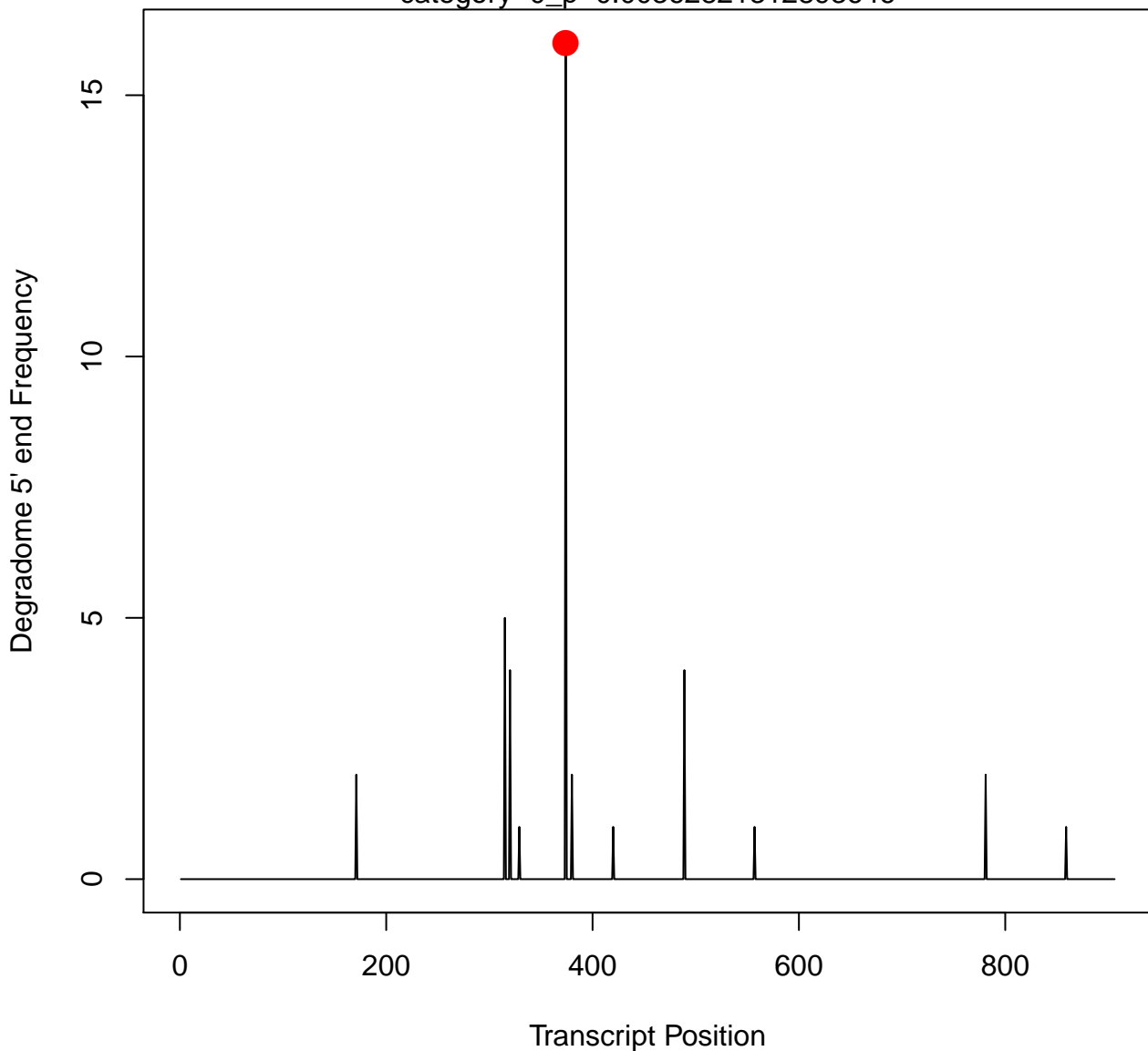

Supplement: Supplementary file 1 [file DataSheet_1.zip › The miRNA-target modules identified by the CleaveLand4/miR396a-5p_evm.model.LG04.896_374_TPlot.pdf]

**T=evm.model.LG05.234\_Q=miR396a-5p\_S=125**

category=1\_p=0.000532226253305179

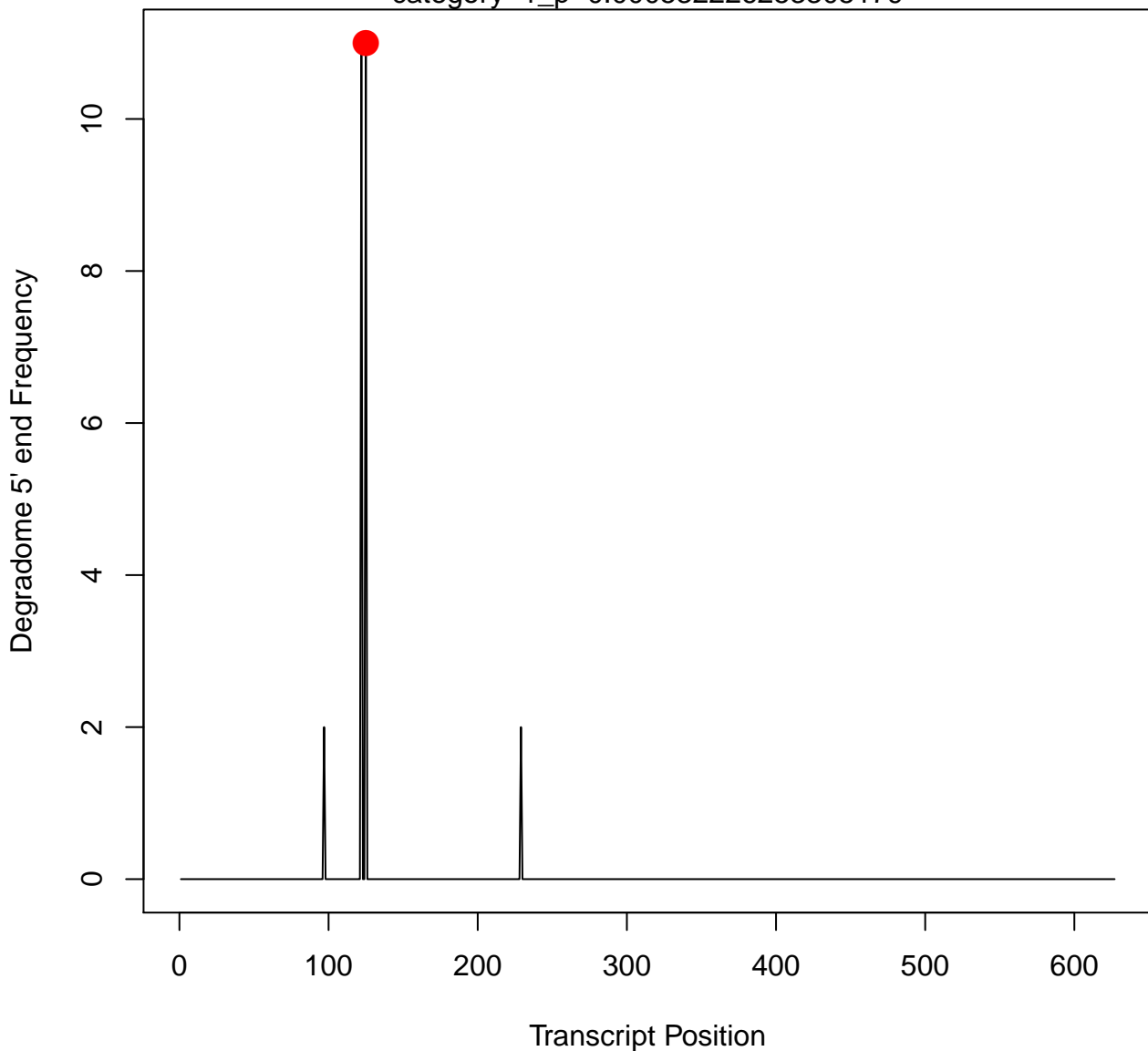

Supplement: Supplementary file 1 [file DataSheet_1.zip › The miRNA-target modules identified by the CleaveLand4/miR396a-5p_evm.model.LG05.234_125_TPlot.pdf]

**T=evm.model.LG06.2004\_Q=miR396a-5p\_S=572**

category=0\_p=0.00878855119413746

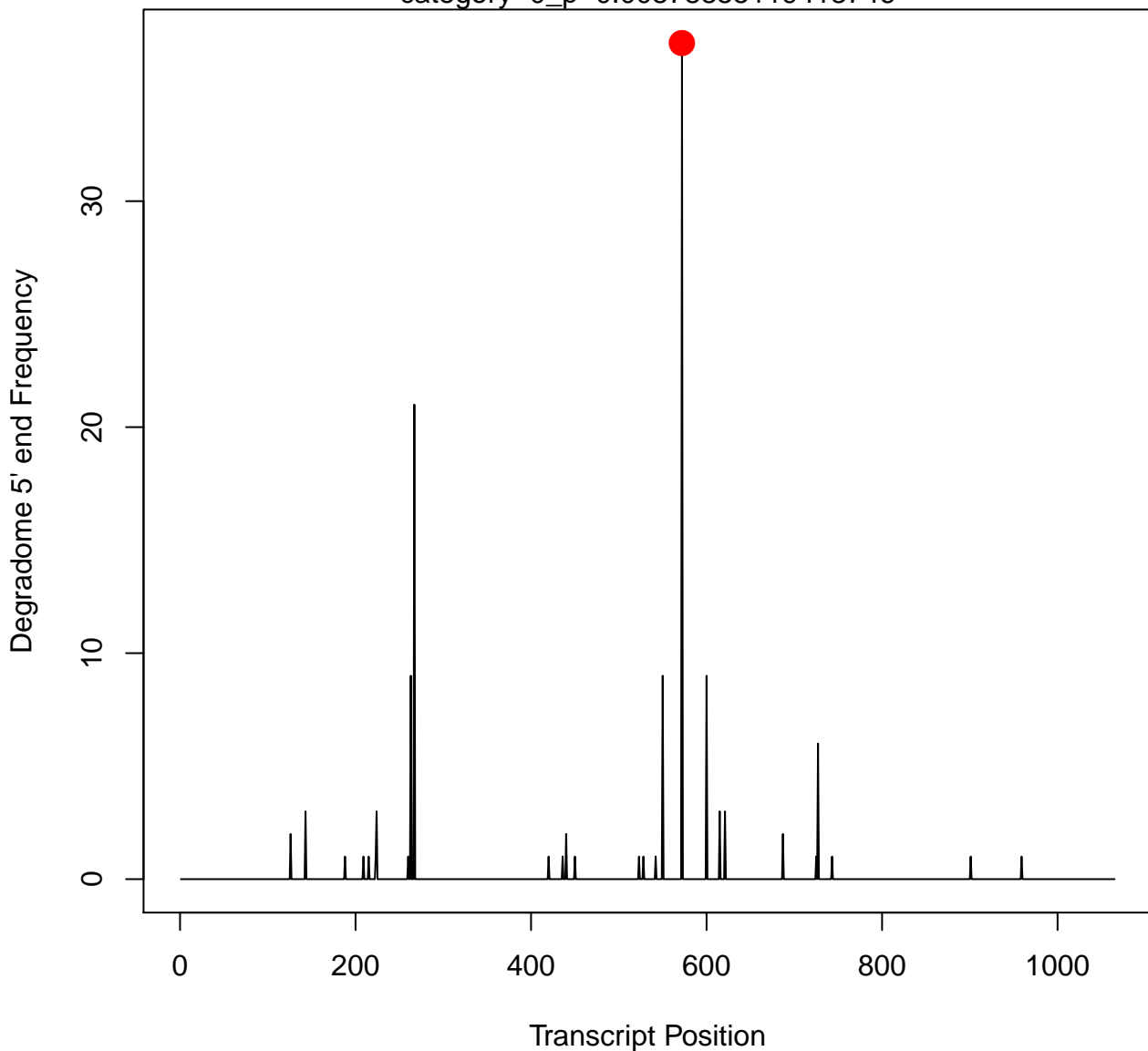

Supplement: Supplementary file 1 [file DataSheet_1.zip › The miRNA-target modules identified by the CleaveLand4/miR396a-5p_evm.model.LG06.2004_572_TPlot.pdf]

**T=evm.model.LG02.2447\_Q=miR396b-5p\_S=383**

category=0\_p=0.00466242549475071

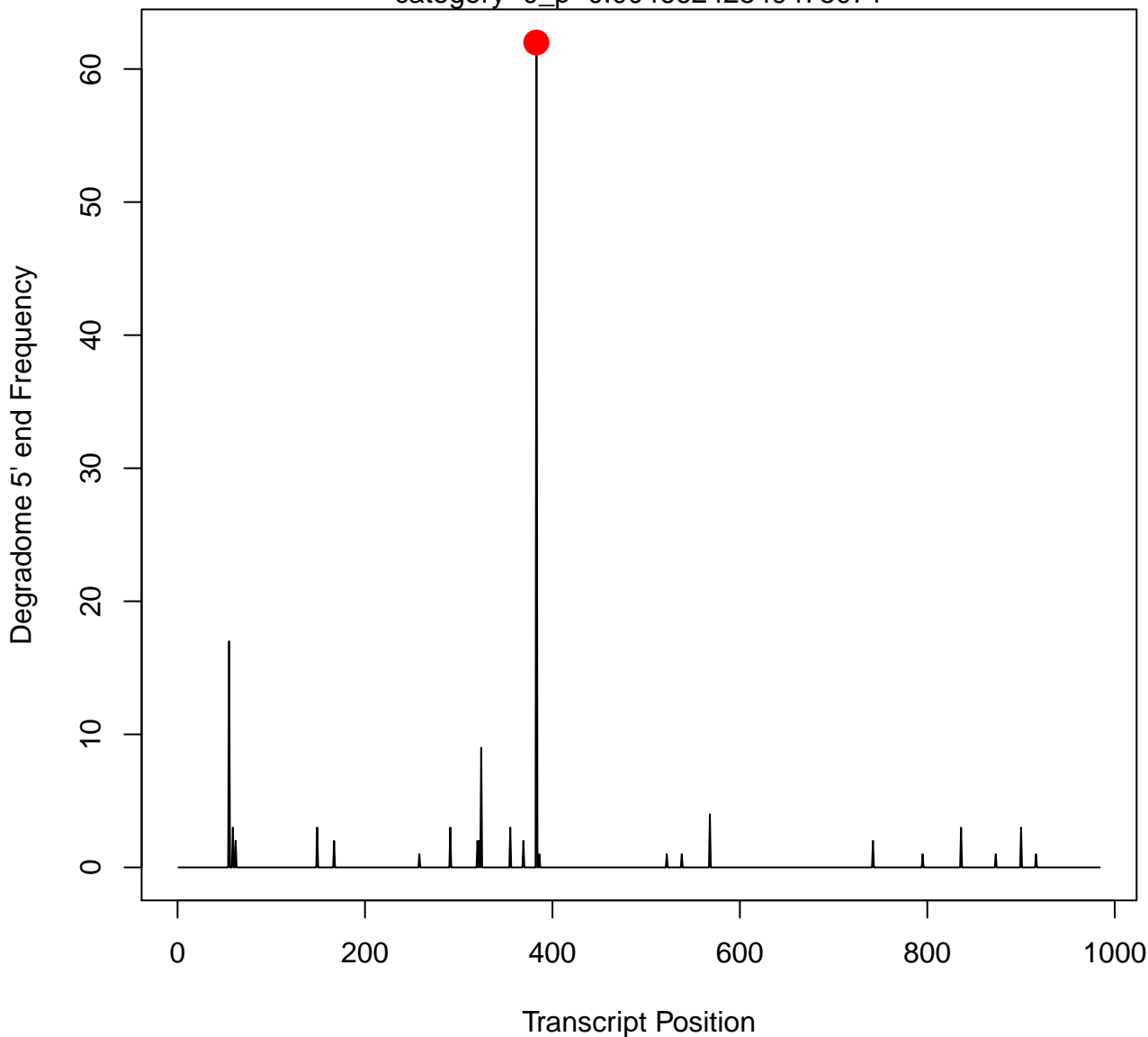

Supplement: Supplementary file 1 [file DataSheet_1.zip › The miRNA-target modules identified by the CleaveLand4/miR396b-5p_evm.model.LG02.2447_383_TPlot.pdf]

**T=evm.model.LG02.6265\_Q=miR396b-5p\_S=644**

category=0\_p=0.00672763087043382

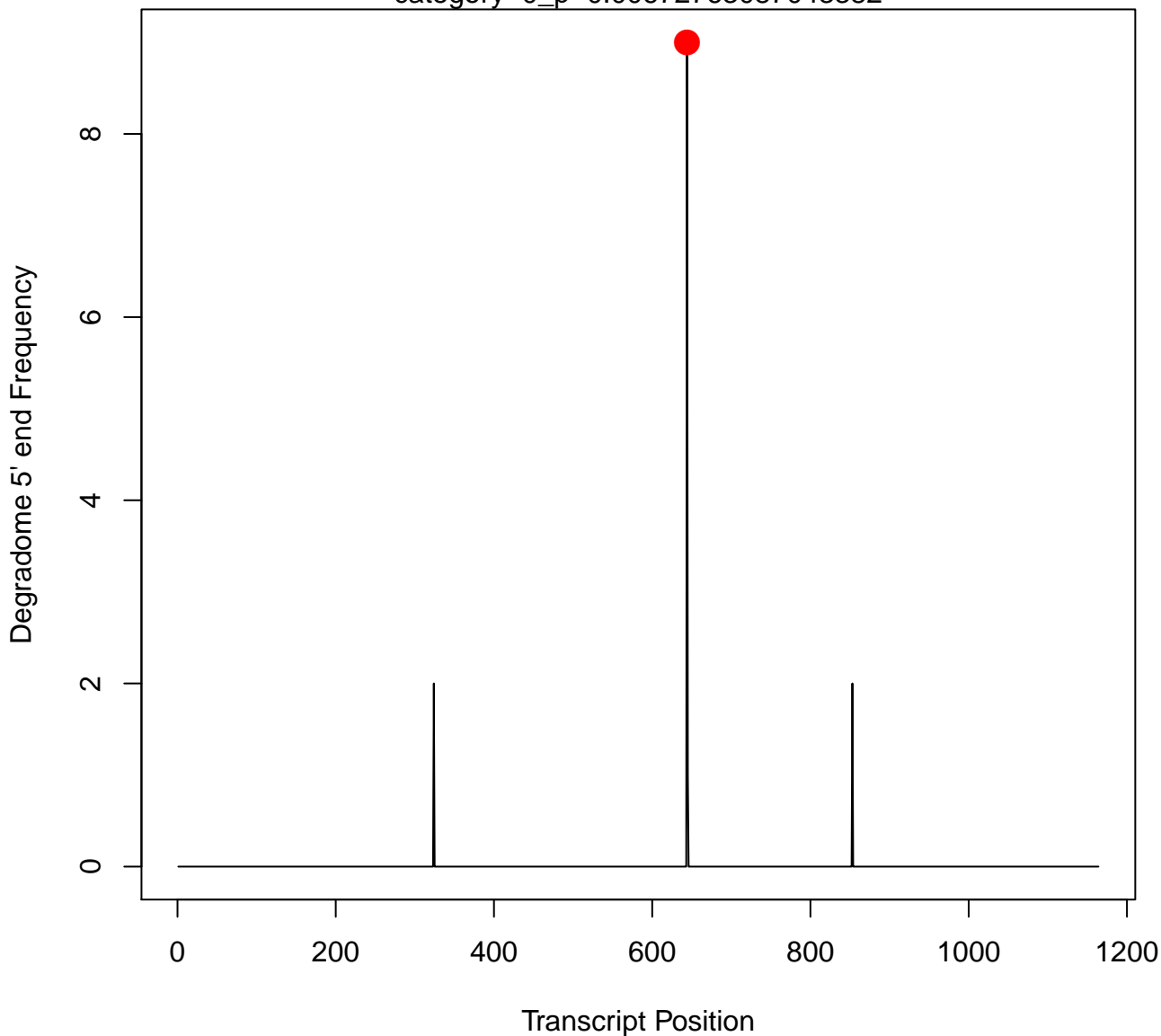

Supplement: Supplementary file 1 [file DataSheet_1.zip › The miRNA-target modules identified by the CleaveLand4/miR396b-5p_evm.model.LG02.6265_644_TPlot.pdf]

**T=evm.model.LG04.896\_Q=miR396b-5p\_S=374**

category=0\_p=0.00311070403873104

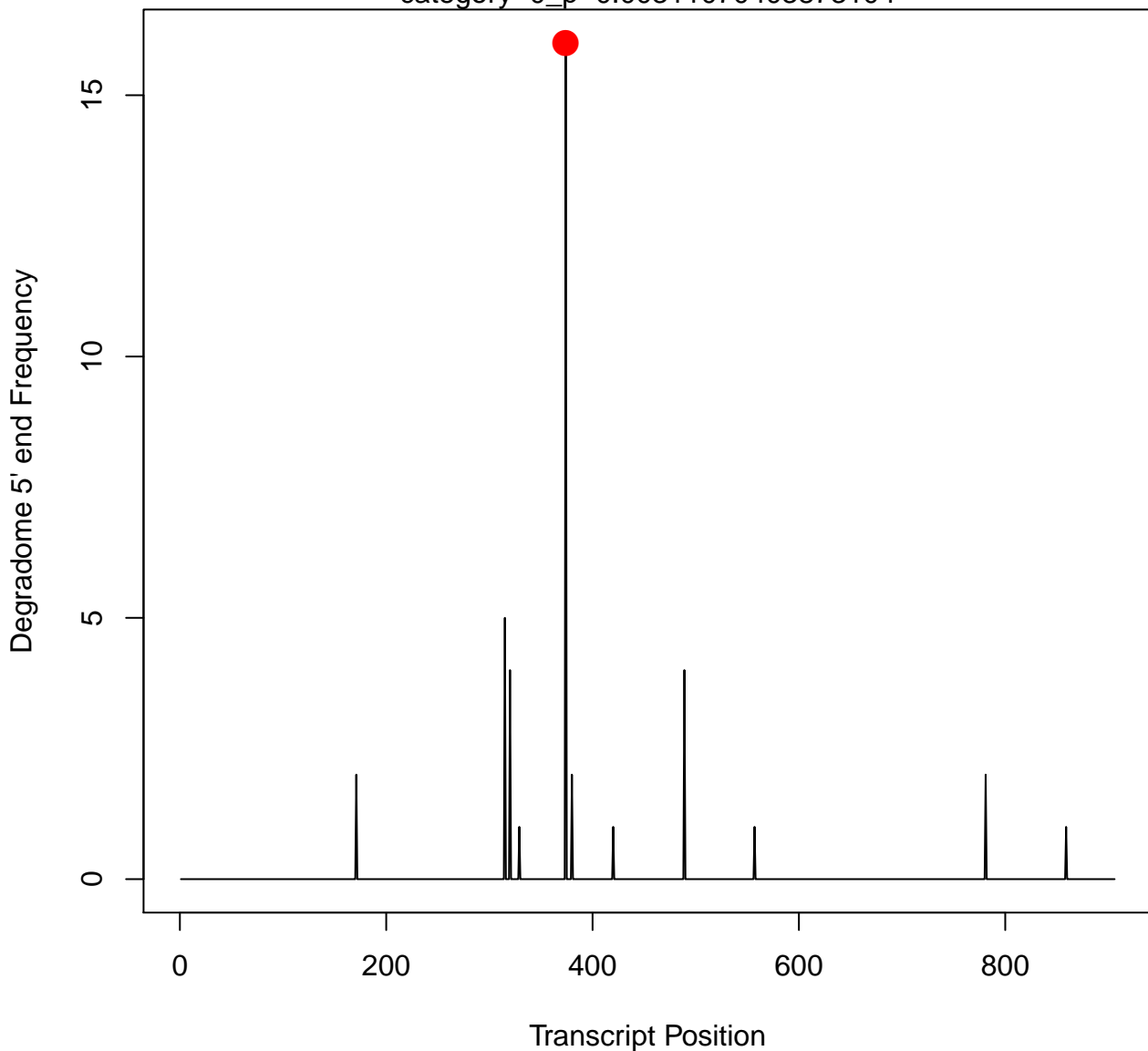

Supplement: Supplementary file 1 [file DataSheet_1.zip › The miRNA-target modules identified by the CleaveLand4/miR396b-5p_evm.model.LG04.896_374_TPlot.pdf]

**T=evm.model.LG05.234\_Q=miR396b-5p\_S=125**

category=1\_p=0.00039919625194329

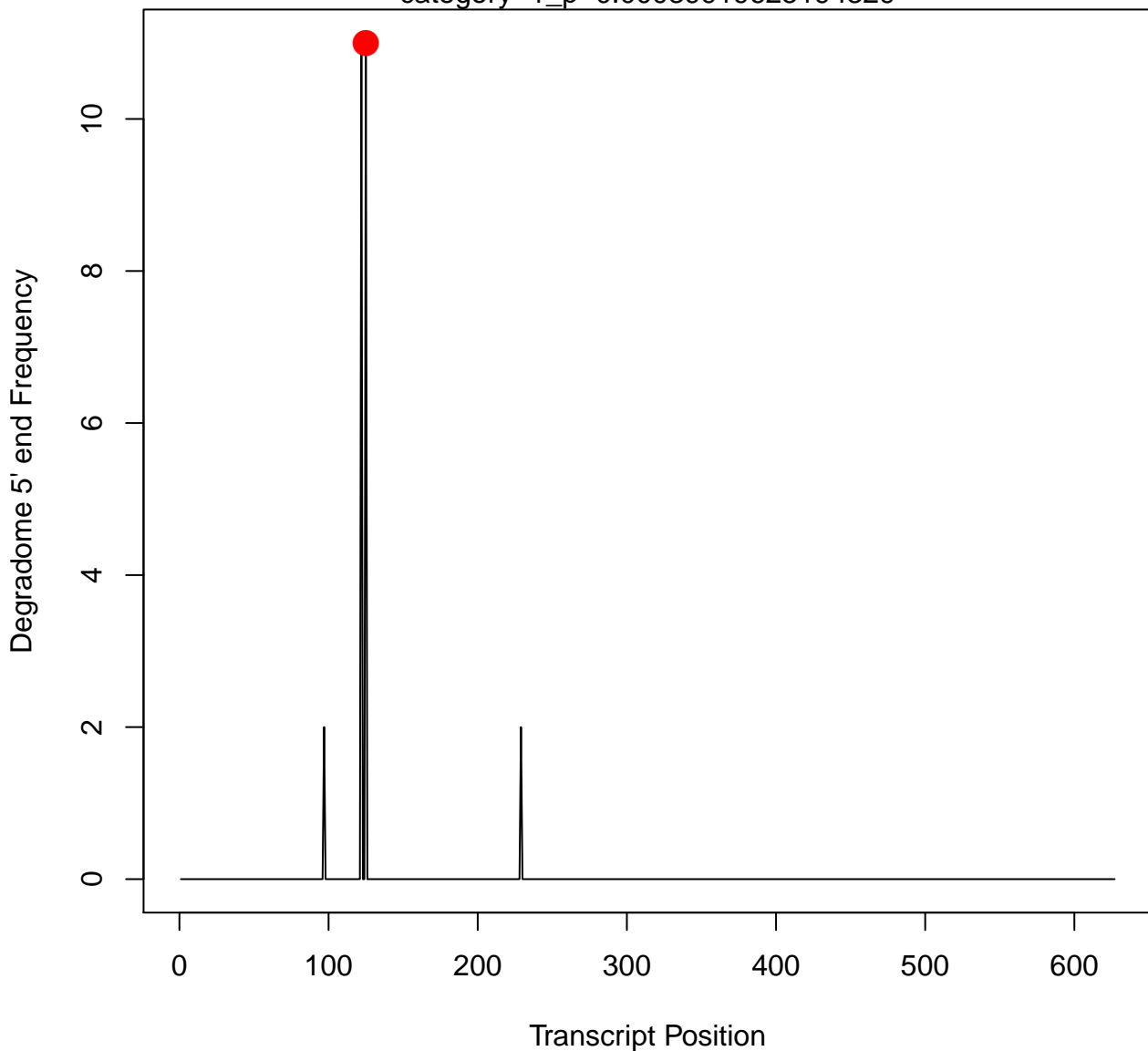

Supplement: Supplementary file 1 [file DataSheet_1.zip › The miRNA-target modules identified by the CleaveLand4/miR396b-5p_evm.model.LG05.234_125_TPlot.pdf]

**T=evm.model.LG01.294\_Q=miR396d-5p\_S=1091**

category=0\_p=0.00207487934604456

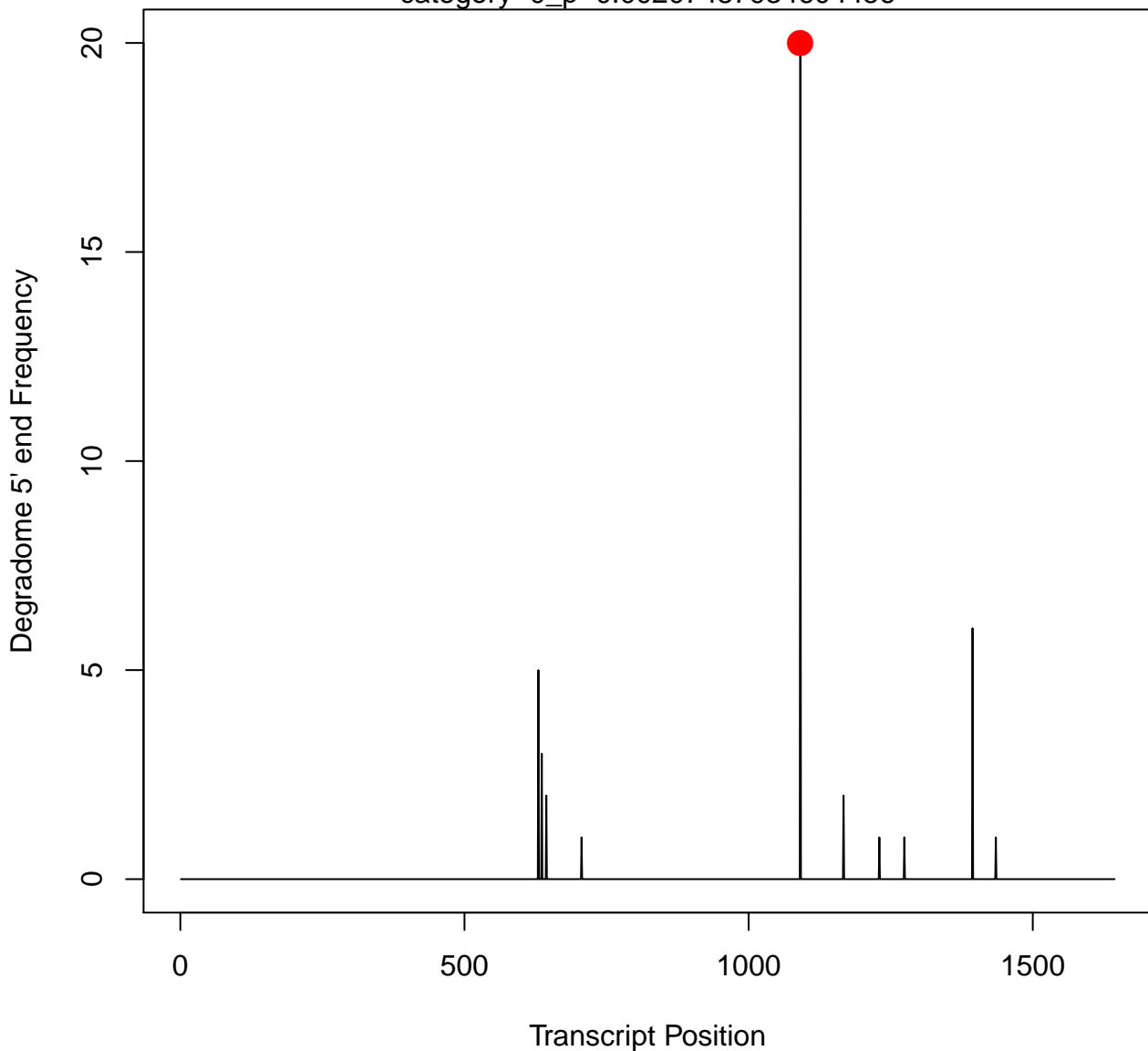

Supplement: Supplementary file 1 [file DataSheet_1.zip › The miRNA-target modules identified by the CleaveLand4/miR396d-5p_evm.model.LG01.294_1091_TPlot.pdf]

**T=evm.model.LG02.1585\_Q=miR396d-5p\_S=1671**

category=0\_p=0.0159681557397247

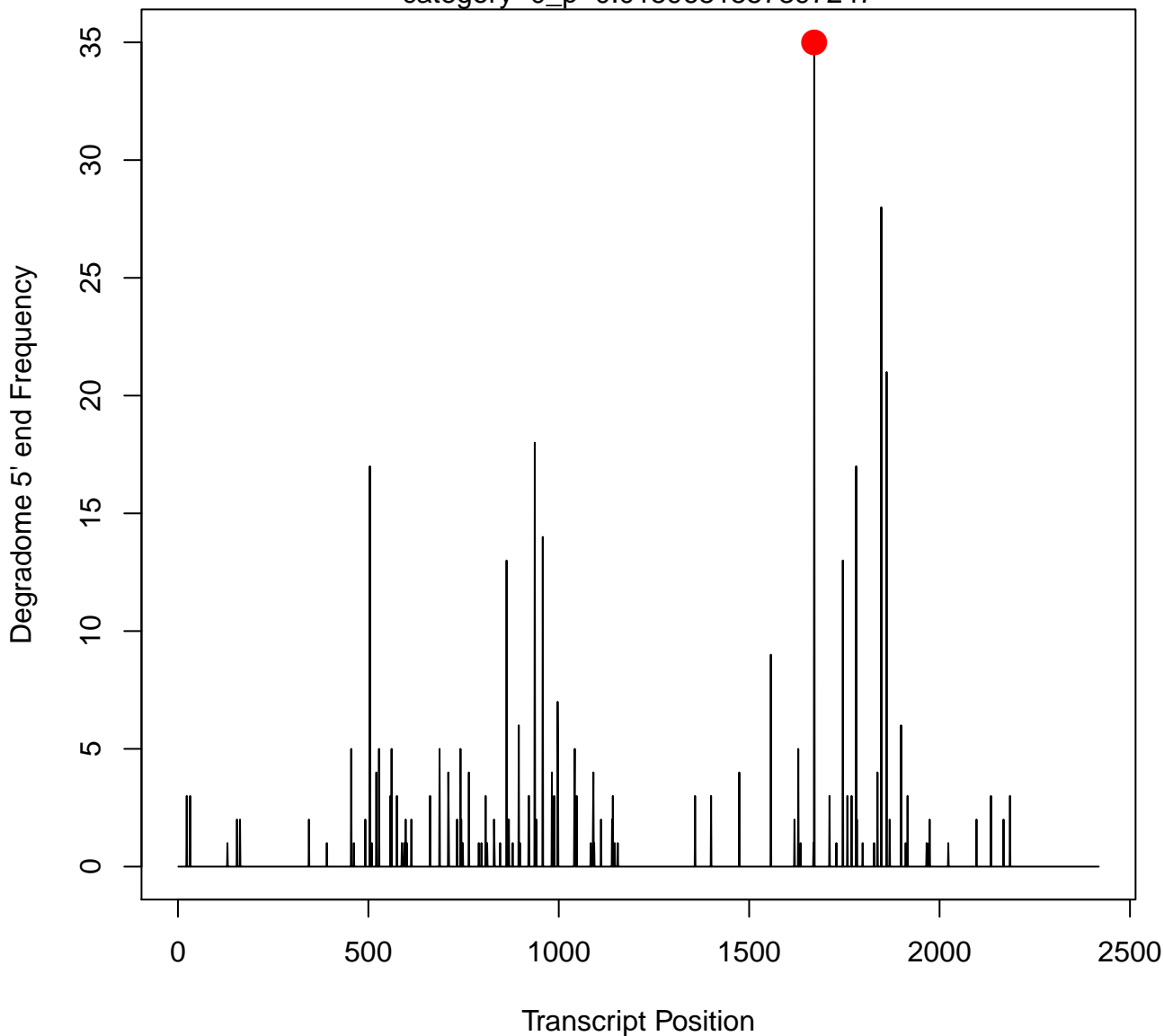

Supplement: Supplementary file 1 [file DataSheet_1.zip › The miRNA-target modules identified by the CleaveLand4/miR396d-5p_evm.model.LG02.1585_1671_TPlot.pdf]

**T=evm.model.LG03.2927\_Q=miR396d-5p\_S=377**

category=0\_p=0.00672763087043382

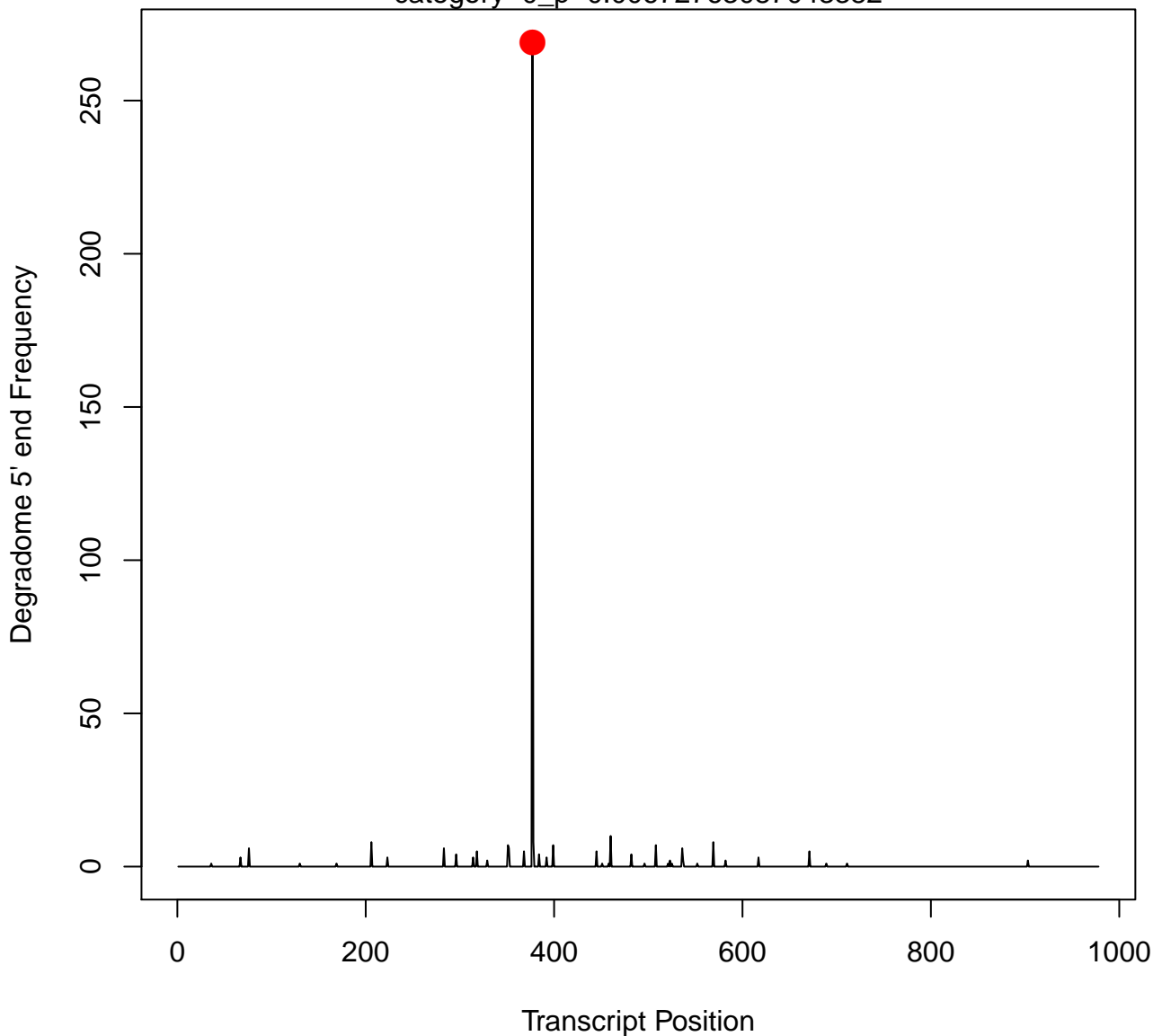

Supplement: Supplementary file 1 [file DataSheet_1.zip › The miRNA-target modules identified by the CleaveLand4/miR396d-5p_evm.model.LG03.2927_377_TPlot.pdf]

**T=evm.model.LG03.637\_Q=miR396d-5p\_S=728**

category=0\_p=0.00311070403873104

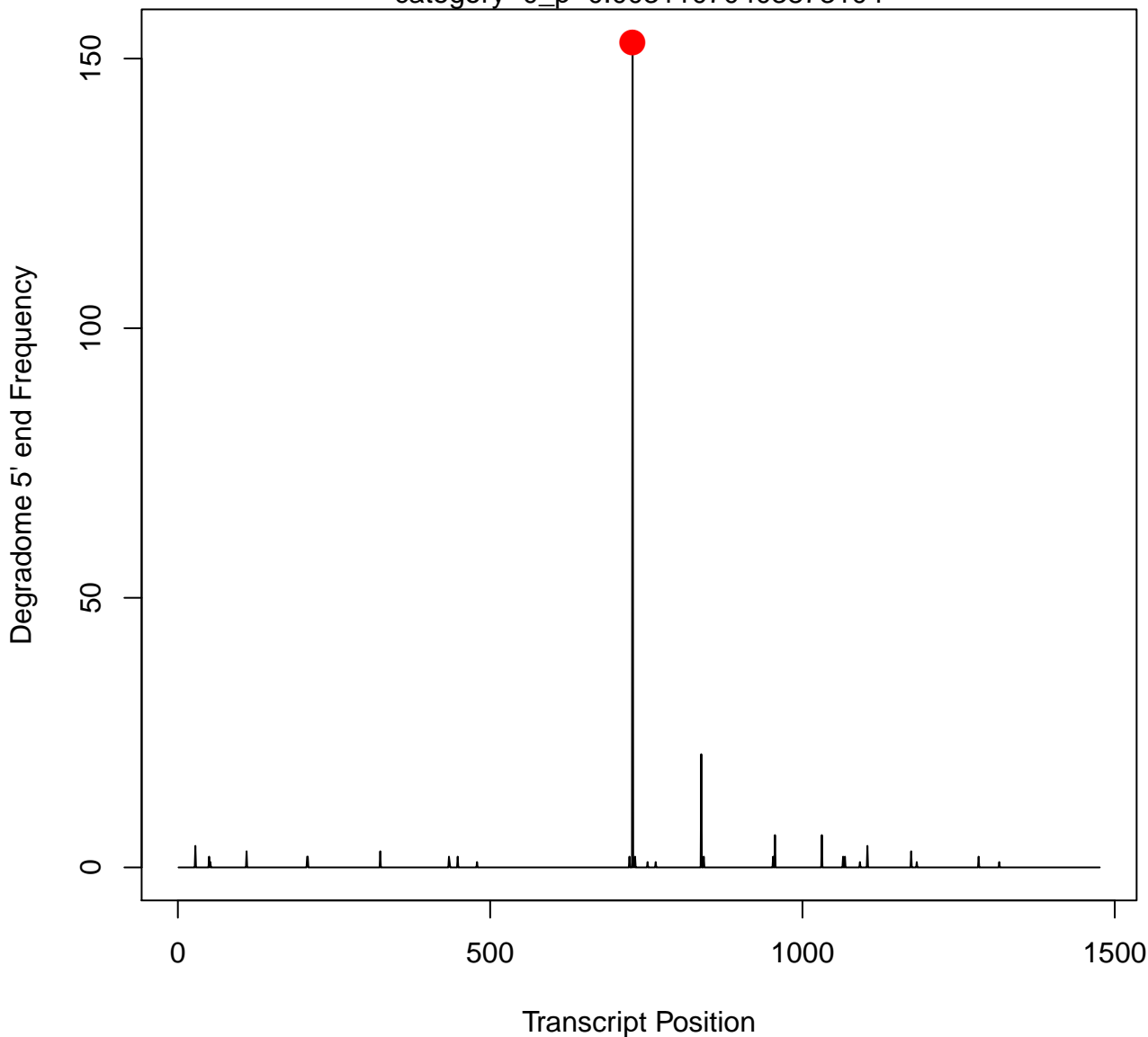

Supplement: Supplementary file 1 [file DataSheet_1.zip › The miRNA-target modules identified by the CleaveLand4/miR396d-5p_evm.model.LG03.637_728_TPlot.pdf]

**T=evm.model.LG05.3790\_Q=miR396d-5p\_S=527**

category=4\_p=0.0423765939260693

Degradome 5' end Frequency

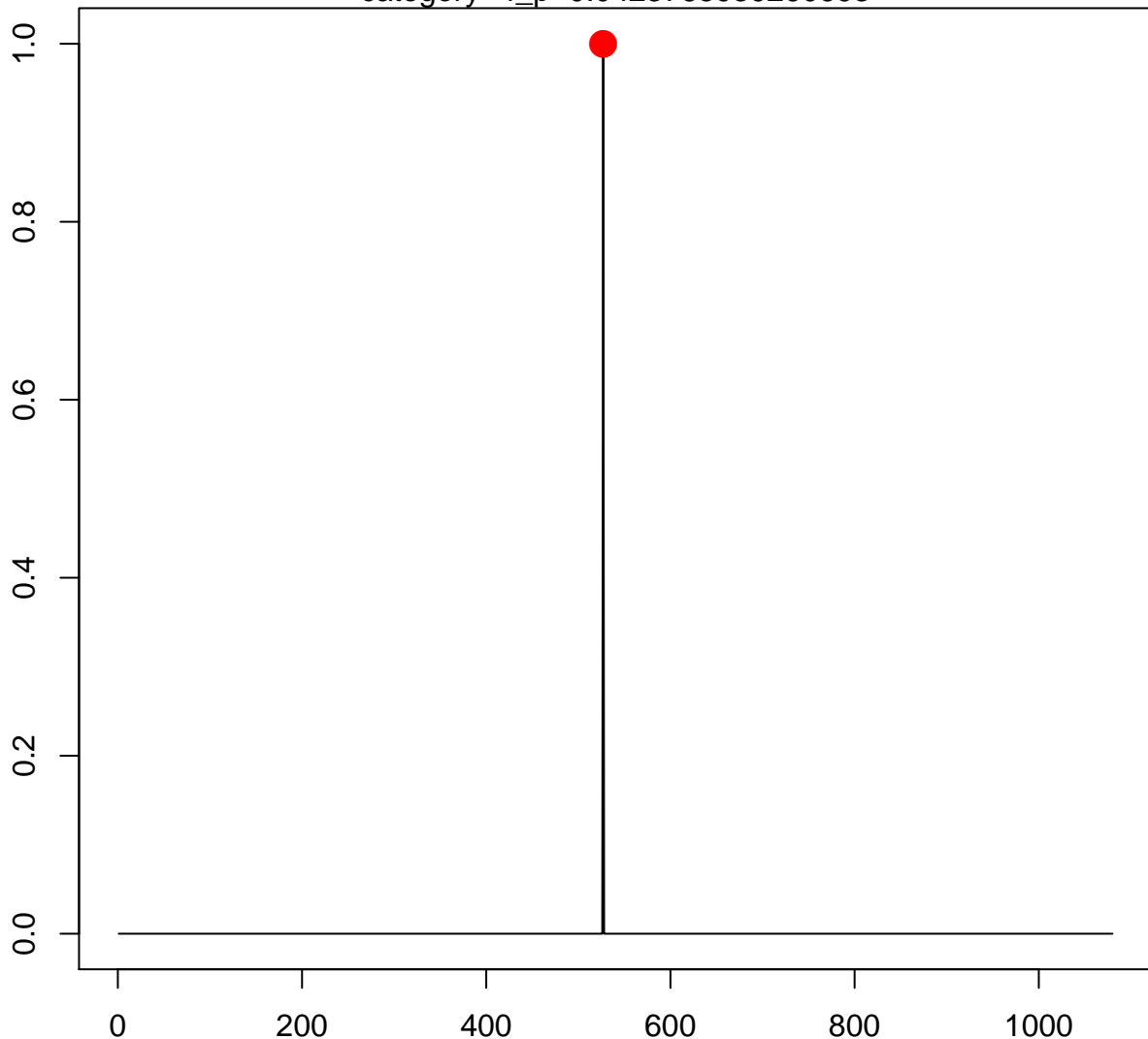

Transcript Position

Supplement: Supplementary file 1 [file DataSheet_1.zip › The miRNA-target modules identified by the CleaveLand4/miR396d-5p_evm.model.LG05.3790_527_TPlot.pdf]

**T=evm.model.LG06.2004\_Q=miR396d-5p\_S=572**

category=0\_p=0.00775862610766487

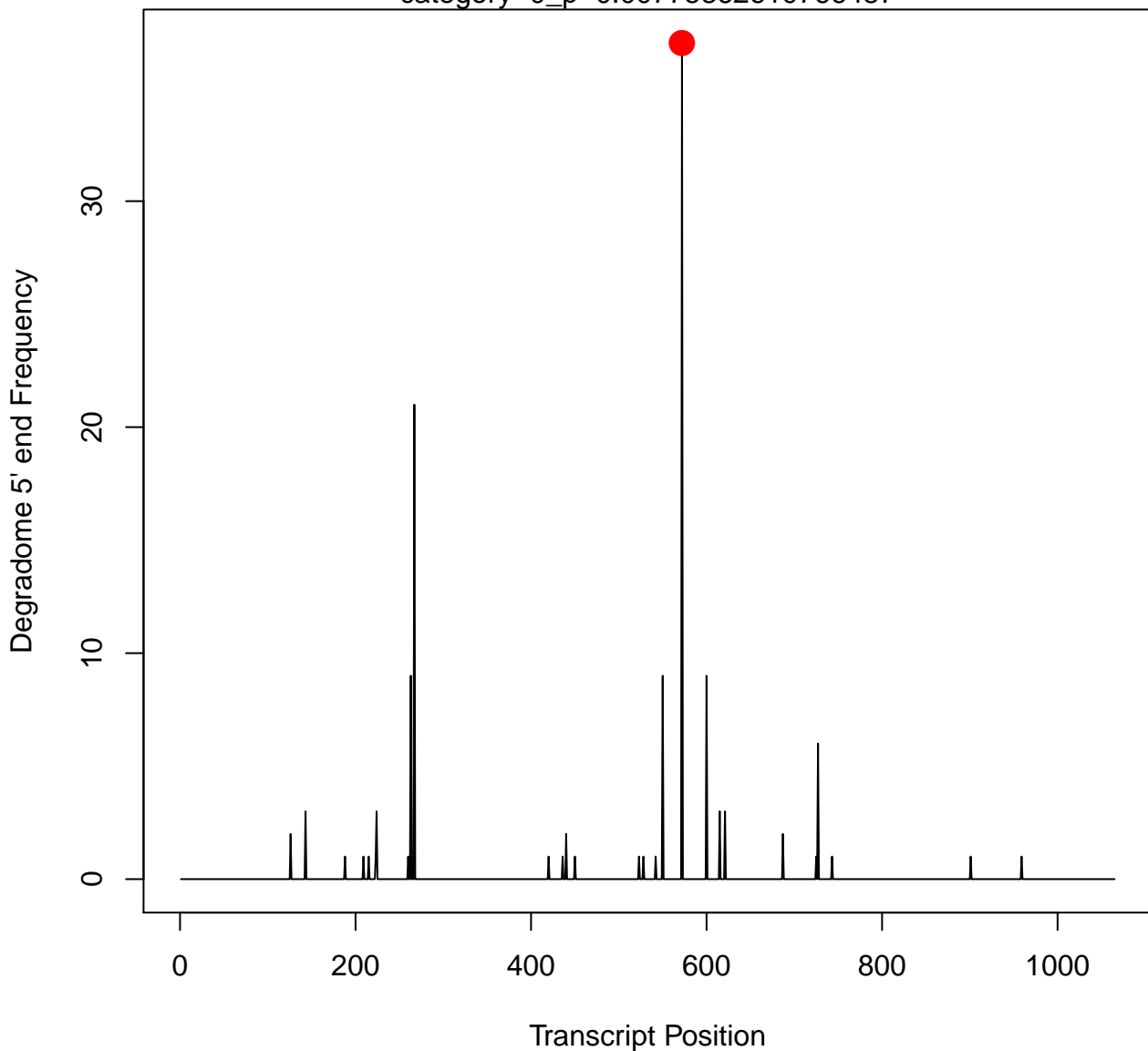

Supplement: Supplementary file 1 [file DataSheet_1.zip › The miRNA-target modules identified by the CleaveLand4/miR396d-5p_evm.model.LG06.2004_572_TPlot.pdf]

**T=evm.model.LG04.831\_Q=miR414-5p\_S=2593**

category=2\_p=0.00874999063480097

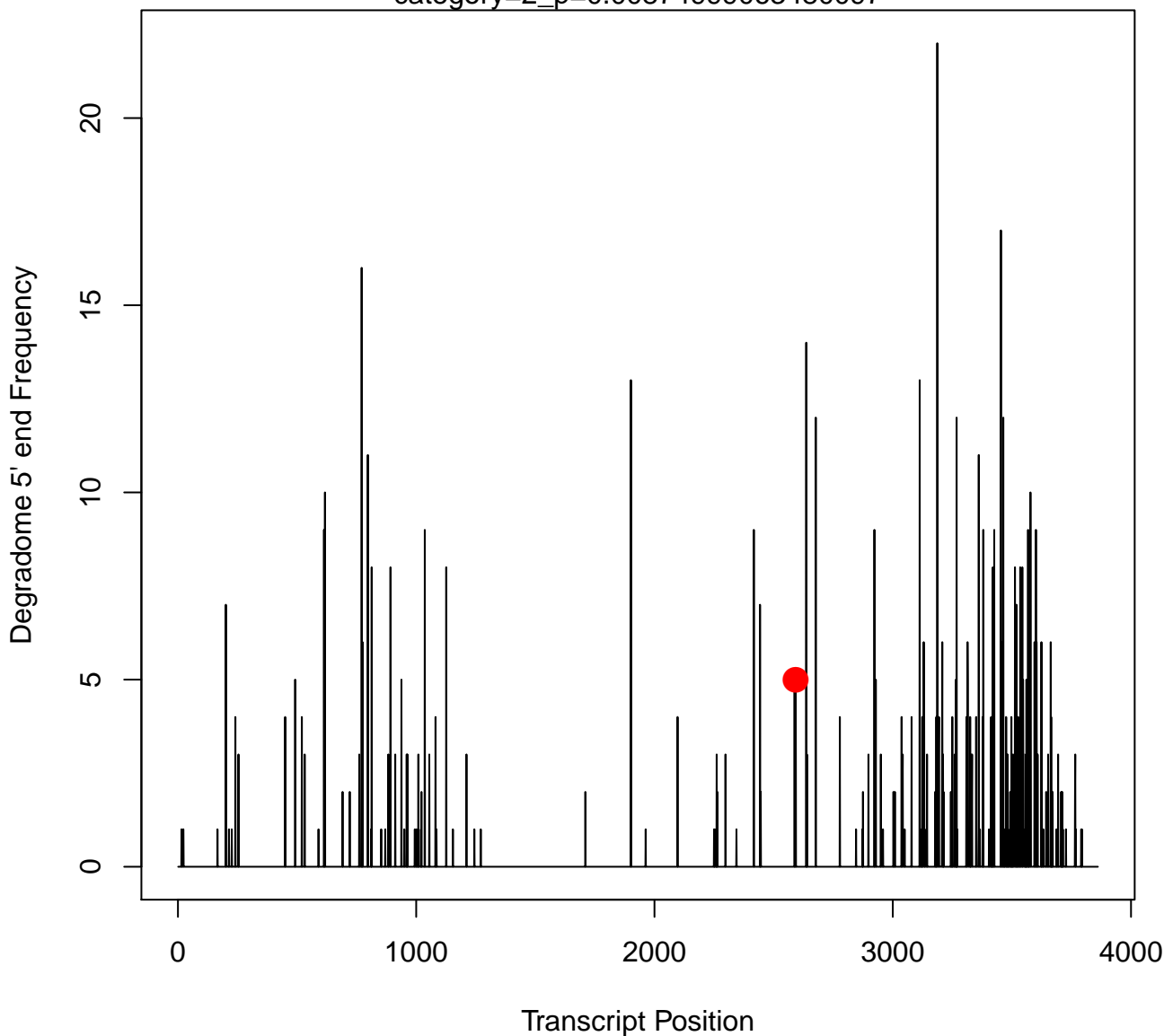

Supplement: Supplementary file 1 [file DataSheet_1.zip › The miRNA-target modules identified by the CleaveLand4/miR414-5p_evm.model.LG04.831_2593_TPlot.pdf]

**T=evm.model.LG02.3364\_Q=miR482-3p\_S=49**

category=0\_p=0.00103797837257324

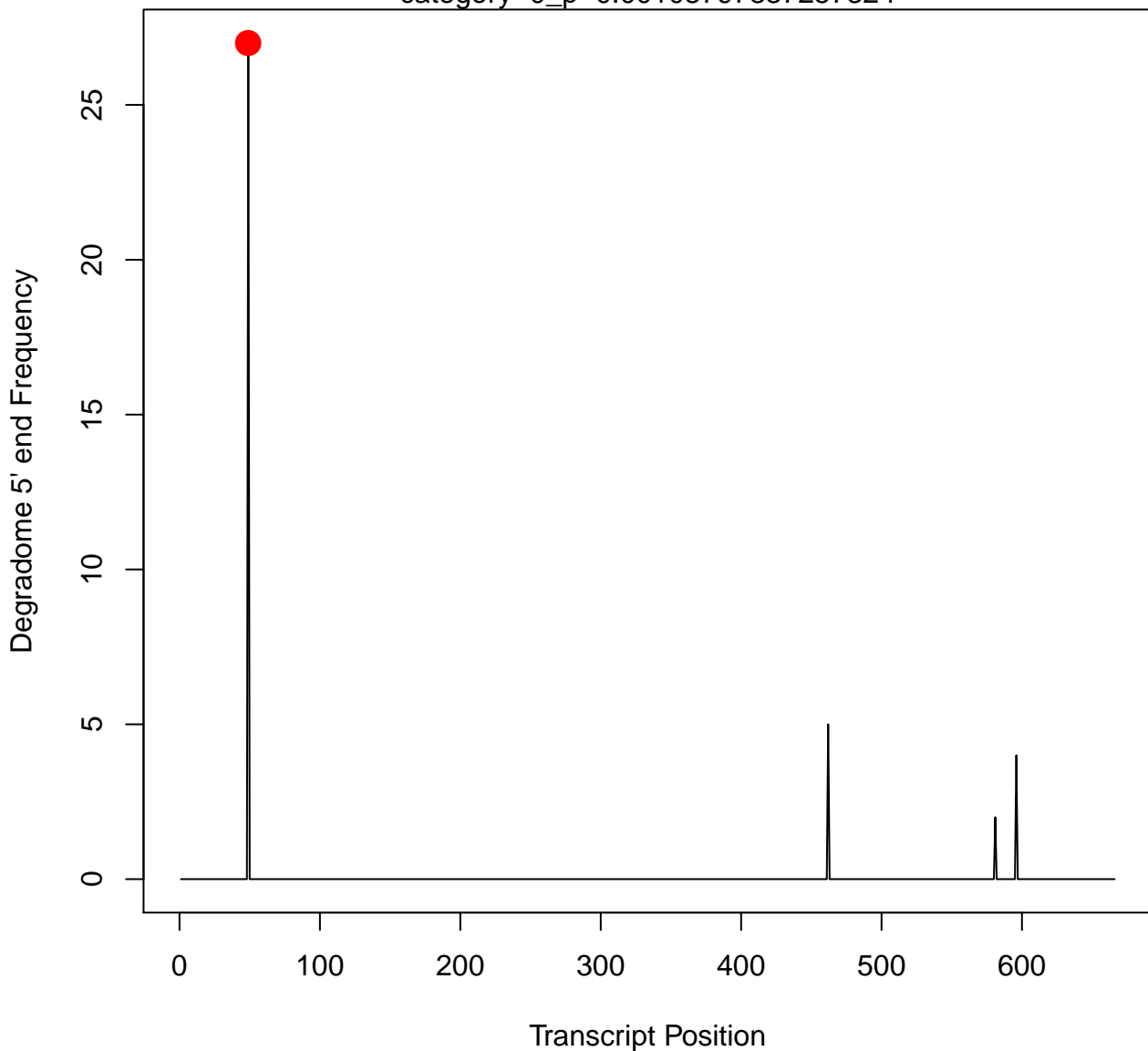

Supplement: Supplementary file 1 [file DataSheet_1.zip › The miRNA-target modules identified by the CleaveLand4/miR482-3p_evm.model.LG02.3364_49_TPlot.pdf]

**T=evm.model.LG02.6040\_Q=miR482-3p\_S=108**

category=0\_p=0.00362821312893646

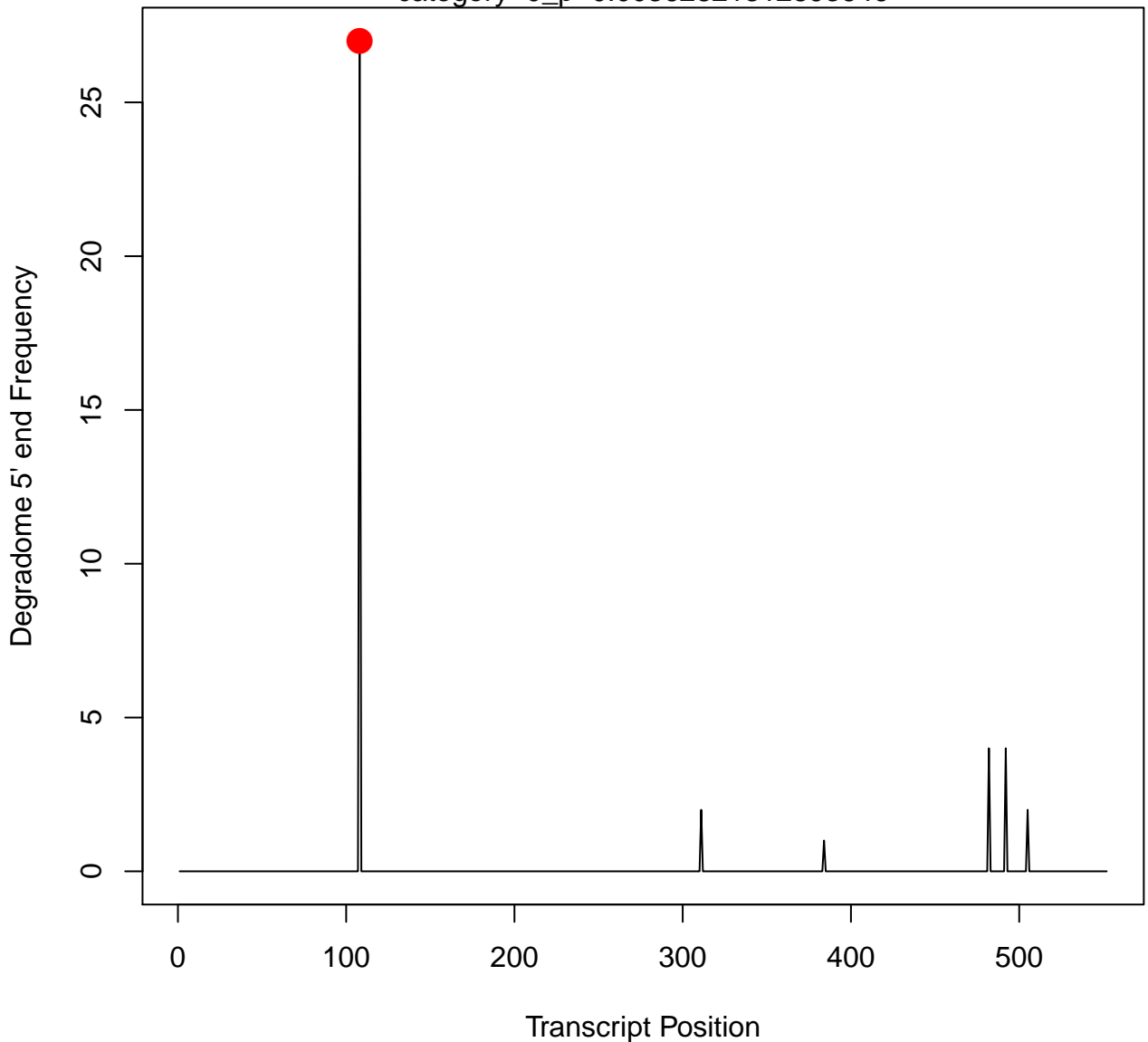

Supplement: Supplementary file 1 [file DataSheet_1.zip › The miRNA-target modules identified by the CleaveLand4/miR482-3p_evm.model.LG02.6040_108_TPlot.pdf]

**T=evm.model.LG02.985\_Q=miR482-3p\_S=46**

category=0\_p=0.00051912393111464

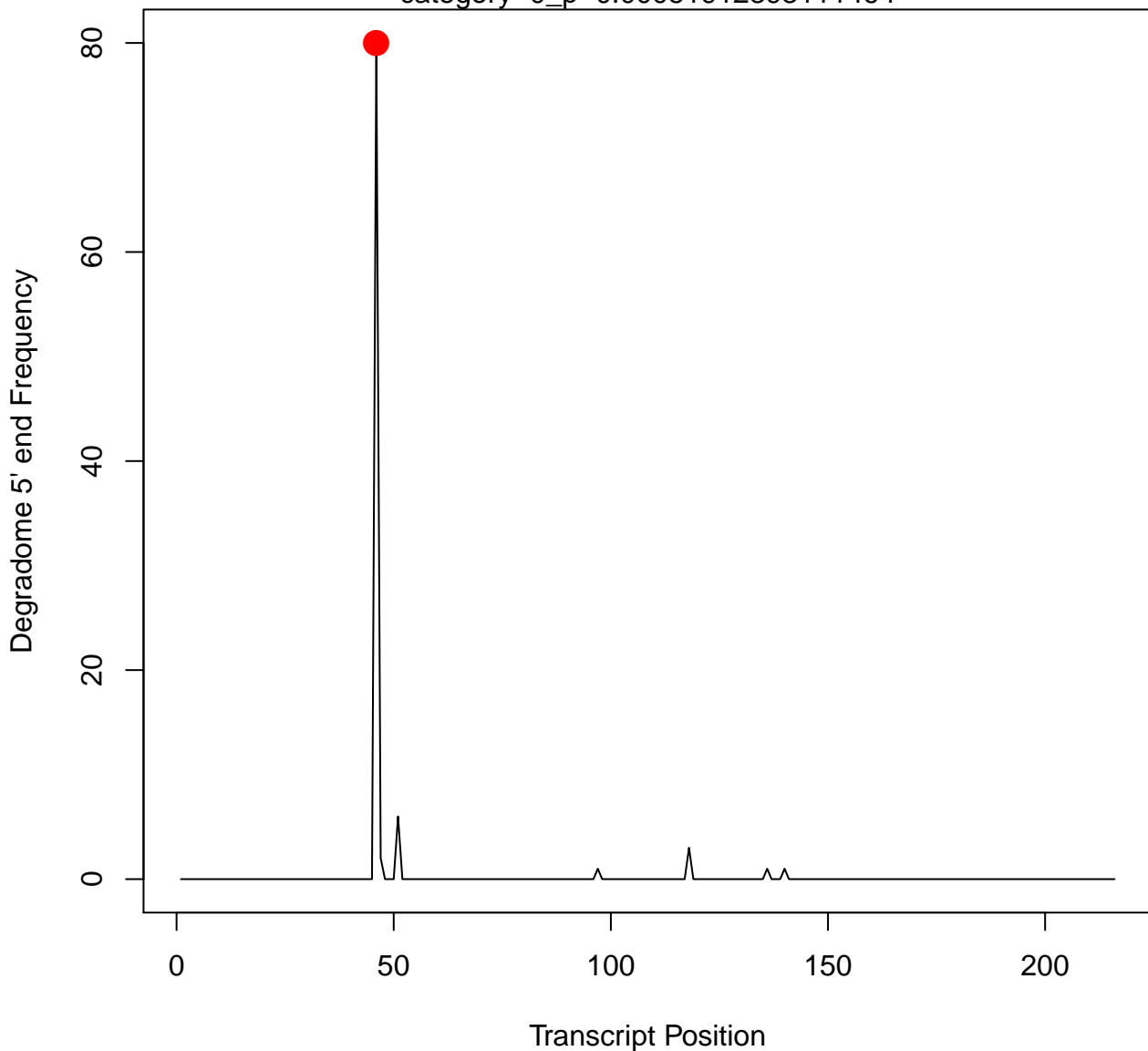

Supplement: Supplementary file 1 [file DataSheet_1.zip › The miRNA-target modules identified by the CleaveLand4/miR482-3p_evm.model.LG02.985_46_TPlot.pdf]

**T=evm.model.LG06.4441\_Q=miR482-3p\_S=565**

category=2\_p=0.0260209548157995

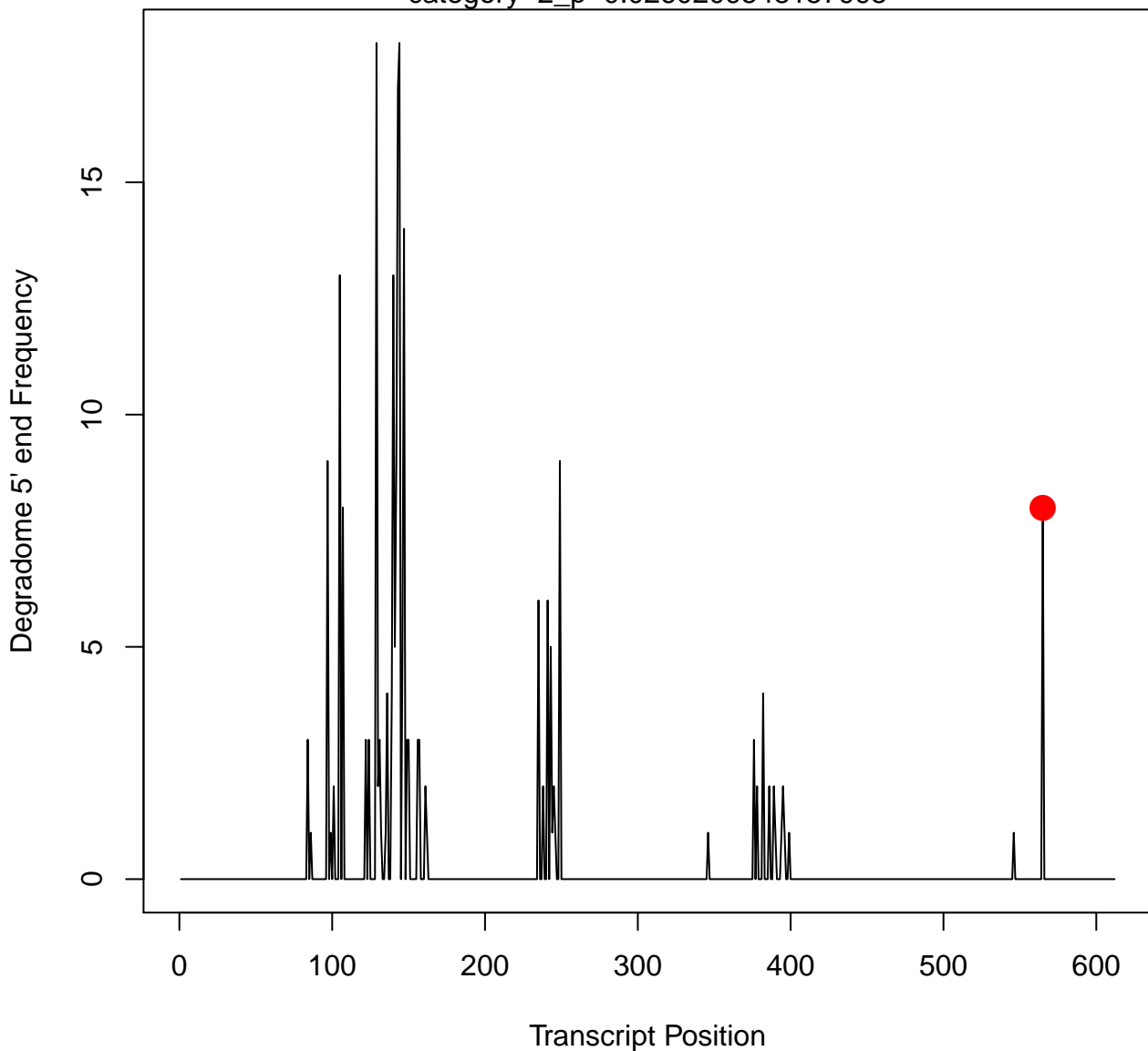

Supplement: Supplementary file 1 [file DataSheet_1.zip › The miRNA-target modules identified by the CleaveLand4/miR482-3p_evm.model.LG06.4441_565_TPlot.pdf]

**T=evm.model.LG02.1185\_Q=miR5041-3p\_S=685**

category=0\_p=0.0230957565261816

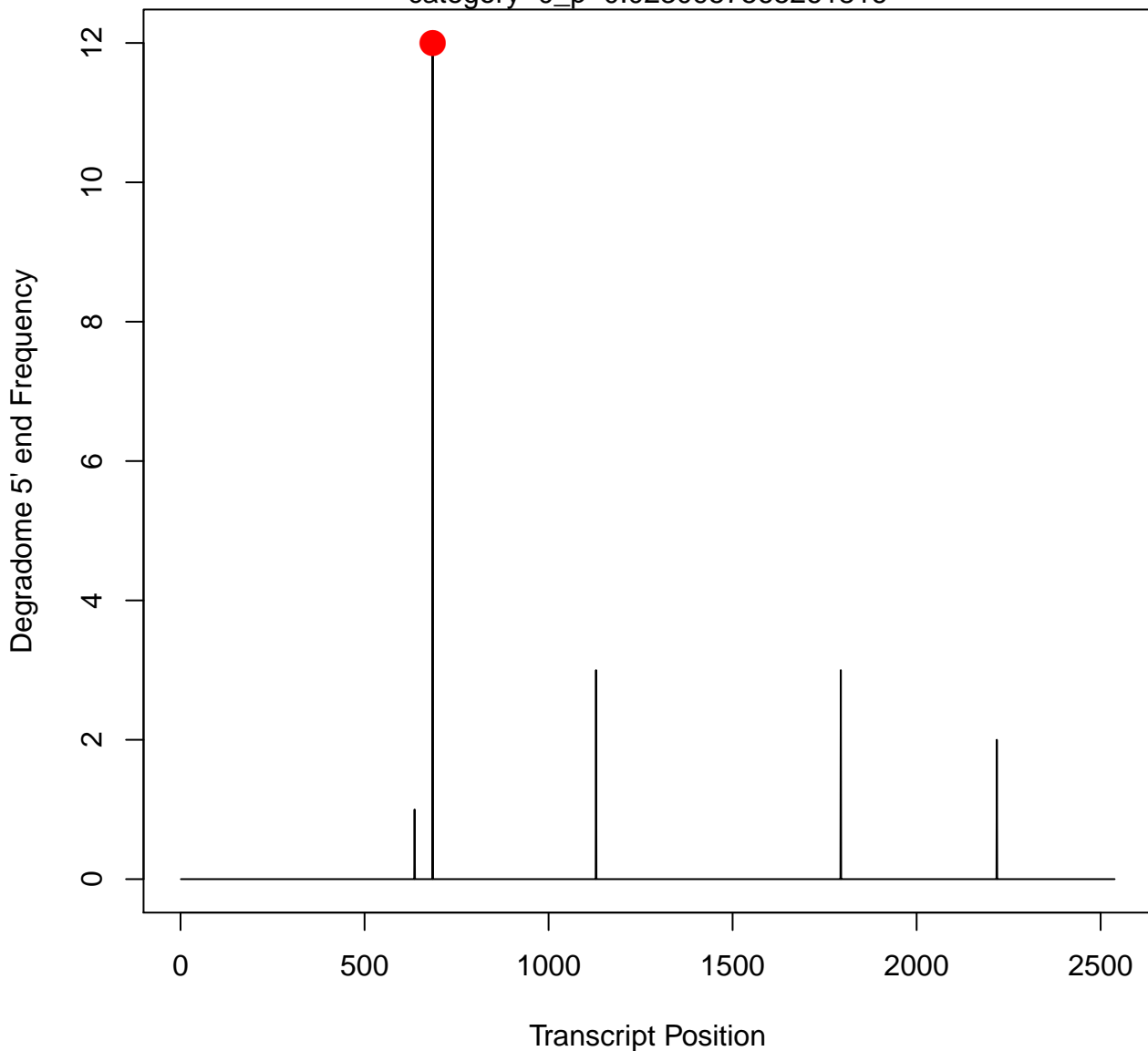

Supplement: Supplementary file 1 [file DataSheet_1.zip › The miRNA-target modules identified by the CleaveLand4/miR5041-3p_evm.model.LG02.1185_685_TPlot.pdf]
